# An un-forgotten classic: the nitro-Mannich reaction between nitrones and silyl nitronates catalysed by B(C_6_F_5_)_3_†

**DOI:** 10.1039/d3sc05672d

**Published:** 2024-01-12

**Authors:** Michael G. Guerzoni, Yara van Ingen, Rasool Babaahmadi, Thomas Wirth, Emma Richards, Rebecca L. Melen

**Affiliations:** a Cardiff Catalysis Institute, School of Chemistry, Cardiff University, Translational Research Hub Maindy Road, Cathays Cardiff CF24 4HQ Cymru/Wales UK RichardsE10@cardiff.ac.uk MelenR@cardiff.ac.uk; b School of Chemistry, Cardiff University, Main Building Park Place Cardiff CF10 3AT Cymru/Wales UK

## Abstract

Herein we report the B(C_6_F_5_)_3_-catalysed nitro-Mannich reaction between nitrones and silyl nitronates, affording silyl-protected α-nitro hydroxylamines with yields up to 99% and diastereoselectivities up to 99 : 1. Crucially, the obtained products can be converted into 1,2-diamines under simple reductive conditions. This work provides a new orthogonal method to the existing routes for the instalment of a nitro moiety under Lewis acid catalysed conditions, and expands the state-of-the-art substrate scope with respect to the silyl nitronates.

## Abstract 2

Herein we report the B(C_6_F_5_)_3_-catalysed nitro-Mannich reaction between nitrones and silyl nitronates, affording silyl-protected α-nitro hydroxylamines with yields up to 99% and diastereoselectivities up to 99 : 1.

## Graphical abstract

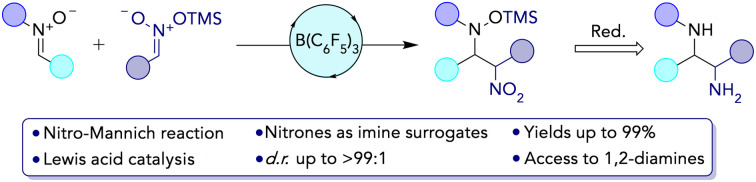

## Introduction

**Scheme 1:**
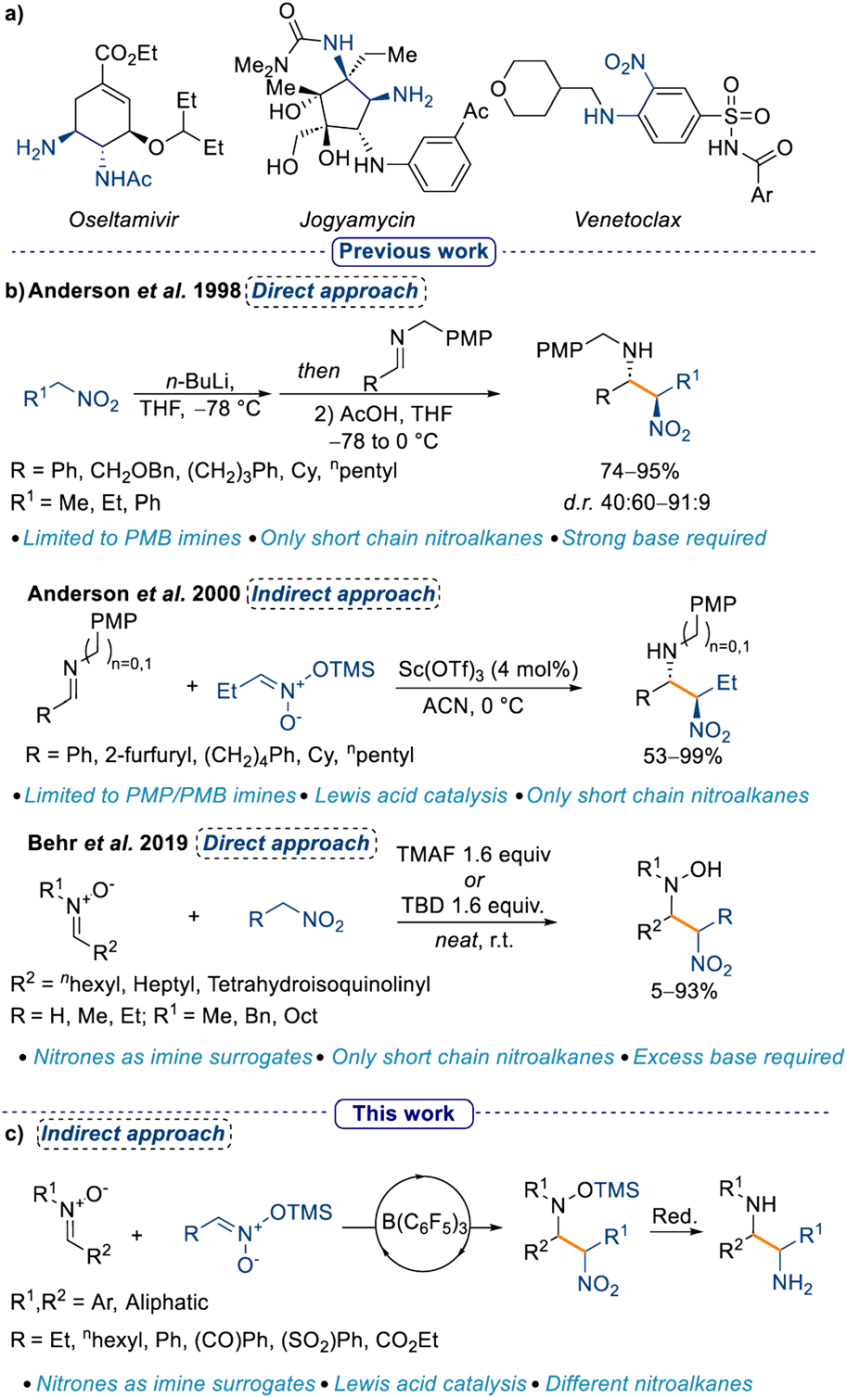
(a) 1,2-Diamine and 1,2-nitroamine containing drugs and natural products, (b) previous examples of the nitro-Mannich reaction, and (c) this work.

Amongst all small molecule drug candidates approved from the US FDA until 2014, 84% of them possessed at least one nitrogen atom.^1^ The frequent occurrence of nitrogen-containing moieties in drug candidates necessitates continuous development of new methods to install them.^2,3^ Nitrogen in drug scaffolds can take on several different oxidation states (*e.g.* as an amine or as a nitro group), and may be present in an open chain or embedded in a heterocycle. Skeletal editing as an approach for the synthesis of nitrogen-containing heterocycles has recently gained traction,^4–6^ however, the classical nucleophilic addition to activated electrophiles remains the most viable and exploited method for aliphatic substrates. The Mannich reaction is a well-established method to synthesise α-functionalised amines starting from an imine (or iminium ion) and a suitable nucleophile, generally an enolate.^7,8^ A direct evolution from the Mannich reaction is the nitro-Mannich reaction (also known as Aza-Henry reaction), where the nucleophile is a nitronate, the “enol” form of a nitro group.^9–11^ The importance of the nitro-Mannich reaction lies in its utility to synthesise 1,2-nitroamine products, where two nitrogen atoms in different oxidation states are in close proximity (Scheme 1a, see Venetoclax). This motif can be leveraged for the selective manipulation of one of the two nitrogen centers,^12–14^ and provides an accessible platform to the 1,2-diamine functionality (Scheme 1a).^9,15–19^ Furthermore, despite the genotoxicity associated with the nitro group,^20^ the possibility to introduce it as a masked carbonyl (*cf.* Nef reaction^21^) makes it a valuable handle for the synthesis of drug candidate libraries.

However, the nitro-Mannich reaction possesses some intrinsic limitations such as the instability of some of the nitroamine products and the unfavorable thermodynamics associated with the addition of a nitronate anion to an imine without an acidic catalyst,^10^ and it has thus been defined as a “forgotten classic” of chemistry.^9^ In one of the earliest reports of the nitro-Mannich reaction in 1998 Anderson *et al.* described the use of stoichiometric amounts of *n*-BuLi in a direct approach to the synthesis of the 1,2-nitroamine moiety which, upon reduction with SmI_2_ affords the 1,2-diamine derivative in good yields and diastereoselectivities.^10^ Later, the same group developed the first Lewis acid catalysed indirect nitro-Mannich reaction with pre-formed silyl nitronates and PMB/PMP-protected imines (PMB = *p*-methoxybenzyl; PMP = *p*-methoxyphenyl; Scheme 1b).^22^ The authors noticed that the PMP protection was crucial for obtaining good diastereoselectivities, hence the reaction was limited to only a few imines. Moreover, the scope with respect to different silyl nitronates had not been explored. This protocol was further improved in 2005 using a Cu catalyst and a chiral ligand, which allowed the formation of nitroamines in yields up to 91% with almost full enantiocontrol (up to 94% ee).^23^ However, the scope was again limited to the simple 1-nitropropane. A direct approach for the nitro-Mannich reaction, that is, making the nitronate *in situ*, would be more appealing, yet the only reports of this used an excess of nitro alkane and it was restricted to specifically designed imines.^24–28^ A turning point occurred in 2019, when Behr *et al.* showed the possibility to use nitrones as imine surrogates, however, the substrate scope was limited to short chain nitro alkanes and required excess base, which in turn limited the functional group tolerability (Scheme 1b).^29^ In addition, a large excess of the nitro nucleophile was required, as the authorsobserved that the process was reversible and following formation of the first nitro-Mannich product, a retro-nitro-Mannich addition occured, redelivering the starting material. The use of nitrones as imine surrogates is a longstanding approach in a variety of transformations in organic chemistry.^30^ Moreover, nucleophilic addition to nitrones affords hydroxylamine products, which have recently been used as nitrogen radical precursors, which can increase the synthetic utility of the products.^31^ Additionally, the use of catalytic amounts of a Lewis acid instead of stoichiometric amounts of base is desirable in many regards, ranging from a higher functional group tolerance to less downstream waste. Finally, the possibility to use chiral Lewis acids can open up an avenue to establish an enantioselective synthesis of the 1,2-nitroamine products.^32–35^

Tris(pentafluorophenyl)borane [B(C_6_F_5_)_3_] is a well-established Lewis acid that has previously been shown to activate a wide range of substrates. Recently, we have shown that B(C_6_F_5_)_3_ can catalyse the Mukaiyama-Mannich addition of nitrones with silylenol diazo esters,^36^ and we envisioned that we could similarly employ it to catalyse the nitro-Mannich reaction, providing an orthogonal method to existing procedures. Based on this premise, we decided to expand the applicability of the nitro-Mannich reaction by using nitrones as imine surrogates, and by investigating different silyl nitronates under Lewis acid catalysis conditions. Crucially, our work would deliver silyl protected hydroxylamines, which in turn would be more stable to oxidation in comparison to the hydroxylamine products described in Behr's work. To the best of our knowledge, this is the first example of a Lewis acid catalysed indirect nitro-Mannich reaction using nitrones as imine surrogates.

## Results and discussion

**Fig. 1:**
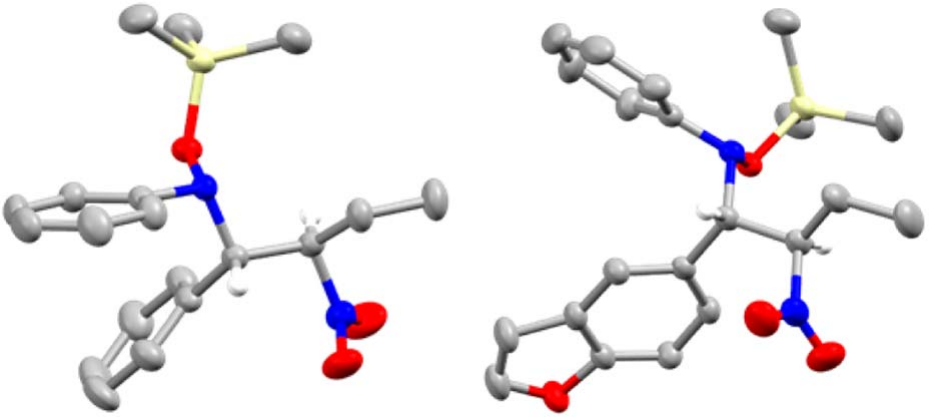
Solid state structure of compound 3a′ (left) and compound 3l′ (right). Ellipsoids shown at 50% probability. Carbon: grey; hydrogen: white; nitrogen: blue; oxygen: red; boron: pink; silicon: yellow. Hydrogens omitted for clarity (except for the α-hydrogen atom).

We began our investigation by reacting nitrone 1aa and silyl nitronate 2a in toluene at room temperature for 6 hours using 20 mol% of B(C_6_F_5_)_3_ (Table S1, see ESI† for full optimisation table). NMR analysis of the crude reaction mixture using 1 equivalent of 1,3,5-trimethoxybenzene as an internal standard showed clean formation of compound (±)-3 as mixture of diastereoisomers ((±)-3a and (±)-3a′) in 80% NMR spectroscopic yield and with a d.r. of 85 : 15. The diastereoisomers were separated, allowing the growth of crystals of the minor diastereoisomer suitable for single crystal X-ray diffraction, which revealed an (±)-(*R*,*S*) configuration of the two chiral centers (Fig. 1, left). This led us to assign by extension the absolute configuration of the major diastereoisomer as (±)-(*R*,*R*).

We then set out to optimise the conditions for the nitro-Mannich reaction giving the best conditions as: 1 equivalent of nitrone (1), 2 equivalents of silyl nitronate (2), 10 mol% of B(C_6_F_5_)_3_, dichloromethane as the solvent (0.1 M), at room temperature for 3 hours (Table 1).

**Table 1:** Reaction scheme and optimisation. Reactions carried out under a nitrogen atmosphere on a 0.1 mmol scale using 1 equiv. of 1aa and 2 equiv. of 2a, unless otherwise stated

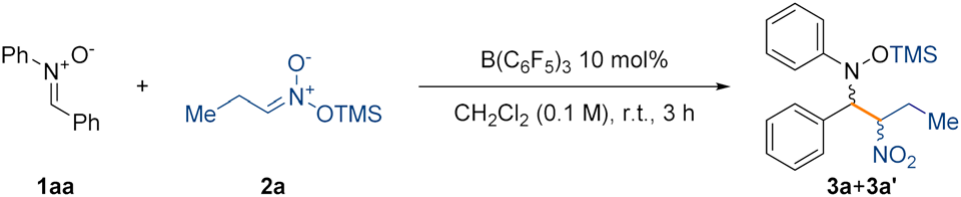			
Entry	Variation from standard conditions	NMR yield^a^ (%)	d.r.^b^ (±)-(*R*,*R* : *R*,*S*)
1	None	90	86 : 14
2	BPh_3_ instead of B(C_6_F_5_)_3_	n.r.	n.d.
3	BF_3_·Et_2_O instead of B(C_6_F_5_)_3_	n.r.	n.d.
4	1 equiv. of TFA	Decomp.	n.d.
5	B(C_6_F_5_)_3_ 5 mol%	71	86 : 14
6	B(C_6_F_5_)_3_ 20 mol%, 1.5 equiv. of 2a	77	86 : 14
7	B(C_6_F_5_)_3_ 20 mol% at 0 °C	79	88 : 12
8	B(C_6_F_5_)_3_ 20 mol% at –41 °C	54	74 : 26
9	B(C_6_F_5_)_3_ 20 mol% at –78 °C	44	76 : 24

a NMR yield calculated from the crude reaction mixture using 0.1 mmol of 1,3,5-trimethoxybenzene as an internal standard.

b Diastereomeric ratio calculated from the ^1^H NMR of the crude reaction mixture.

Weaker Lewis acids such as BPh_3_ did not promote the reaction (Table 1, entry 2) presumably due to the inability to increase the electrophilicity of the carbon of the nitrone upon coordination with the Lewis basic oxygen atom. Surprisingly, Lewis acids of similar strength^37,38^ to B(C_6_F_5_)_3_ such as BF_3_·Et_2_O did not promote the reaction either (Table 1, entry 3), giving only unreacted starting material. Using Brønsted acids such as TFA (trifluoroacetic acid) instead afforded only decomposition products (Table 1, entry 4). Lowering the catalyst loading had a detrimental effect on the yield (Table 1, entry 5), similarly to when the equivalents of the silyl nitronate were decreased (Table 1, entry 6). We also attempted to improve the overall diastereoselectivity of the process by lowering the temperature, but unfortunately such improvement did not occur (Table 1, entries 7–9) (see the ESI† for the full optimisation table).

**Scheme 2:**
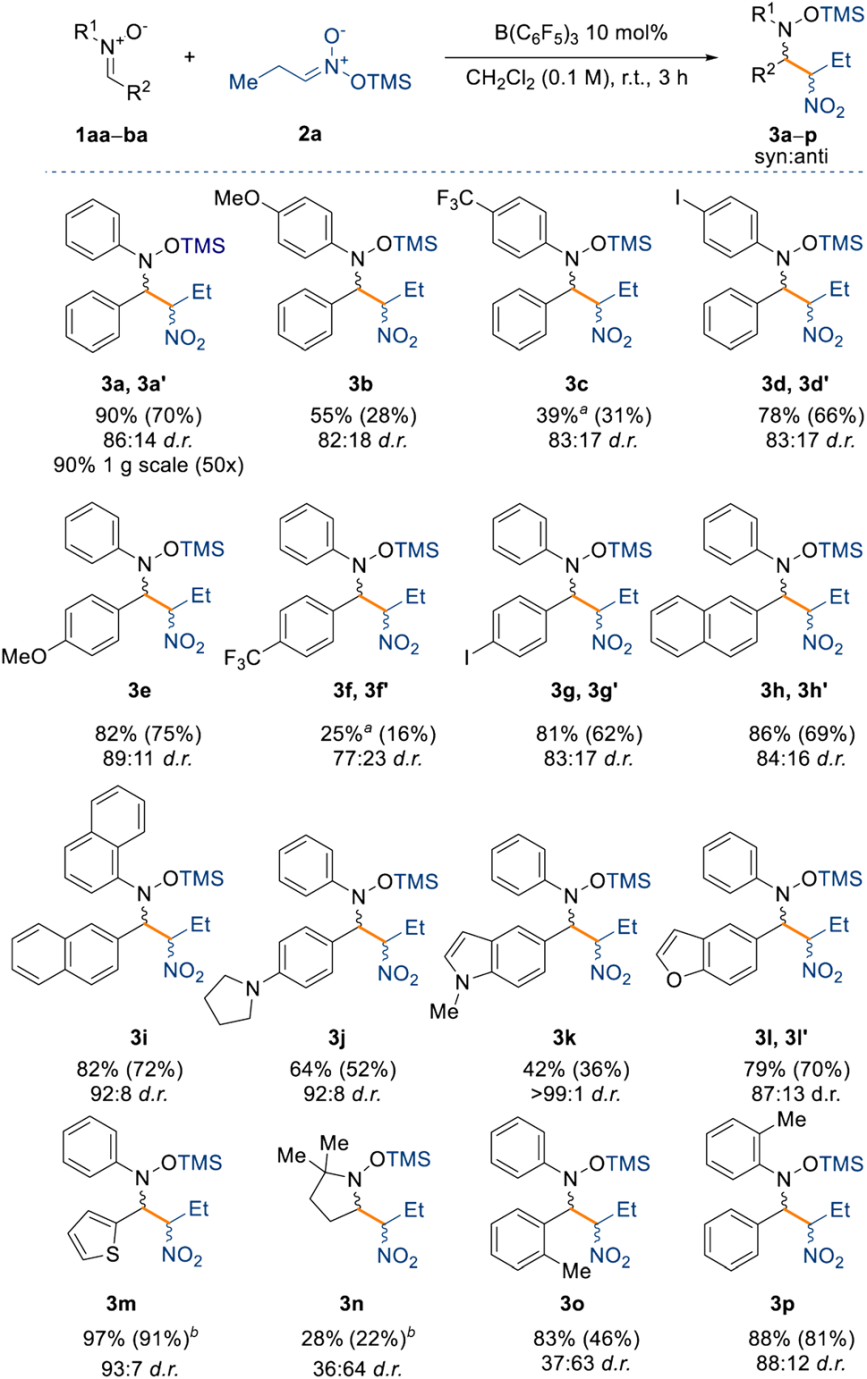
Substrate scope with respect to nitrones 1. All reactions were carried out on a 0.1 mmol scale under the optimised conditions. Yields refer to the NMR spectroscopic yield of both the major and minor diastereoisomer, ratio calculated from the crude reaction using 1 equiv. of 1,3,5-trimethoxybenzene as an internal standard. Isolated yield of the major diastereoisomer in parentheses. ^*a*^Reaction carried out for 24 h. ^*b*^Obtained as mixture of diastereoisomers.

With the optimised reaction conditions in hand, we set out to explore the substrate scope for the reaction (Scheme 2). First, we assessed the effect of electron donating and withdrawing groups on the nitrone (1aa–ba, see ESI†) on the yield and diastereoselectivity of the reaction, whilst keeping the silyl nitronate as 2a. Electron donating groups (*e.g.* –OMe, –NR_2_) were well tolerated affording products 3b, 3e, and 3j in NMR spectroscopic yields between 55% and 82% and up to 89 : 11 d.r. For the methoxy-substituted product 3b the isolated yield was low (28%) merely due to the instability of the product, which readily decomposed.

On the other hand, electron withdrawing groups such as CF_3_ slowed the reaction down requiring longer reaction times (24 h) to obtain an appreciable amount of product 3c and 3f (39% and 25% NMR spectroscopic yields, respectively) albeit not changing the d.r. significantly (83 : 17 and 77 : 23, respectively). Indeed, strongly deactivated nitrones 1ar (R^1^ = Ph; R^2^ = C_6_F_5_), 1aw (R^1^ = Ph; R^2^ = Py), or 1ay (R^1^ = ^*t*^Bu; R^2^ = pyridine 1-oxide) did not react at all under the optimised reaction conditions (see unsuccessful product list in the ESI†). Halogens in the para position, such as iodine, were well tolerated giving 3d (78% yield) and 3g (81% yield), making these compounds useful for possible further manipulation under cross-coupling conditions.^39^ Larger aromatic systems such as naphthyl did not have a negative impact on the yield of the products (3h and 3i), however, we observed an improved diastereoselectivity with compound 3i (92 : 8 d.r.), perhaps hinting at a π⋯π interaction between the catalyst and the nitrone being responsible for the diastereocontrol of the reaction. The reaction was also tolerant to heterocycles generating 3j–3m, which all gave improved diastereoselectivities relative to compound 3a. Indeed, for compound 3k the process reached almost full diastereocontrol (>99 : 1 d.r.).

Next, we were interested in understanding the effect of aliphatic moieties on the nitrone. Nitrones bearing an aliphatic group on the β-position are usually unstable and hence are synthesised *in situ* and used immediately,^40^ however this produces a stoichiometric amount of water which could in turn poison our catalyst. For this reason, we synthesised nitrone 1au (R^1^ = Ph; R^2^ = Cy), which can be isolated but unfortunately the reaction did not occur, even under longer reaction times and higher temperatures (up to 40 °C). The same result was observed when we used nitrones bearing an aliphatic moiety on the α-position as in the case of 1as (R^1^ = Me; R^2^ = Ph) and 1at (R^1^ = ^*t*^Bu; R^2^ = Ph). Surprisingly, with benzyl protected nitrone 1av (R^1^ = Bn; R^2^ = Ph) the reaction did not occur either.

However, in the case of compound 3n, derived from the commercially available nitrone DMPO (5,5-dimethyl-1-pyrroline *N*-oxide), the reaction did occur, albeit in lower yield (28%) and with the opposite diastereoselectivity (36 : 64 d.r.). This result prompted us to further investigate the effect on the diastereoselectivity with sterically demanding groups on the nitrone. To this end, increasing the steric bulk around the electrophilic carbon instead shuts down the reactivity completely, as observed for penta-methyl substituted 1aq (R^1^ = Ph; R^2^ = C_6_Me_5_) and 1ba (R^1^ = Ph; R^2^ = 2-BrC_6_H_4_). Interestingly, we observed that an *o*-tolyl group at R^2^ of the nitrone also inverted the diastereoselectivity in the product (3o) (*vide infra*) and drastically decreased it (36 : 63), but this was not the case in the formation of product 3p where the *o*-tolyl group was at R^1^ position of the nitrone. We propose that the reversal of diastereoselectivity observed for compound 3o might be controlled by the large steric demand of the borane catalyst.

**Fig. 2:**
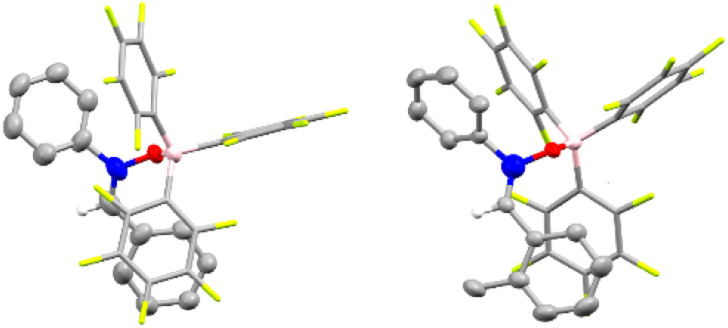
Solid state structure of nitrone 1aa·B(C_6_F_5_)_3_ (left), and nitrone 1ao·B(C_6_F_5_)_3_ (right). Ellipsoids shown at 50% probability, except for C_6_F_5_ groups for clarity. Carbon: grey; hydrogen: white; nitrogen: blue; oxygen: red; boron: pink; fluorine: light green. Hydrogens omitted for clarity except for the α-hydrogen atom.

**Scheme 3:**
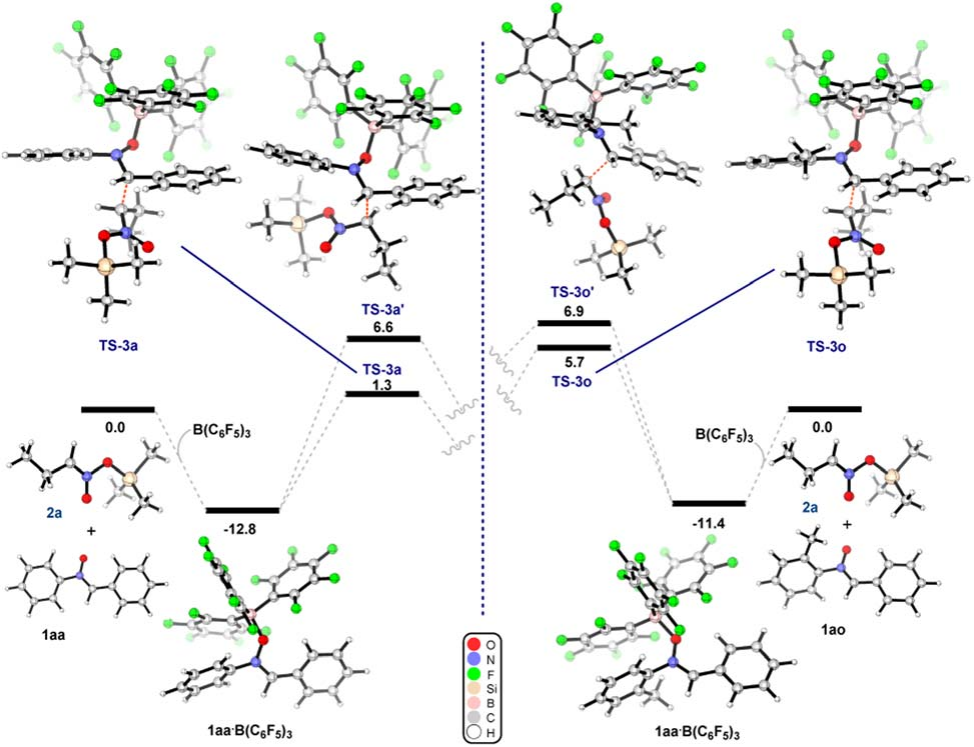
(Left) Energy diagram for the formation of product 3a. (Right) Energy diagram for the formation of product 3o. The relative free energies obtained from SMD/BP86-D3/def2-TZVP//BP86/6-31G* in dichloromethane (see ESI† for full details). Energies are given in kcal mol^−1^.

To gain further insight into this process, we undertook DFT calculations to explore the transition state structures. As expected, the reactions between 1aa (R^1^ = R^2^ = Ph) and 1ao (R^1^ = Ph, R^2^ = *o*-tol) with B(C_6_F_5_)_3_ lead to very stable adducts, in which the Lewis acid catalyst is bound to the Lewis basic oxygen of the nitrone, thus increasing the electrophilicity of the carbon and thereby facilitating the subsequent nucleophilic addition. Indeed, the reaction of equimolar amounts of B(C_6_F_5_)_3_ and nitrone 1aa or 1ao in dichloromethane led to the formation of 1aa·B(C_6_F_5_)_3_ and 1ao·B(C_6_F_5_)_3_. Slow evaporation of the reaction mixture led to the formation of crystals suitable for single crystal X-ray diffraction analysis (Fig. 2). In the subsequent transition state detailing the reaction between the adduct and the nitronate, two possible conformations arise which would subsequently lead to the observed *syn* (or (±)-(*R*,*R*)) and *anti* (or (±)-(*R*,*S*)) products (Scheme 3). Consistent with the experimental results, the transition state TS-3a is 5.3 kcal mol^−1^ lower in energy than TS-3a′, accounting for the preferential formation of the *syn* product over the *anti* with 86 : 14 d.r. The corresponding calculations for 3o still indicated a slight preference for the *syn* isomer over the *anti*, albeit with a much smaller energy gap between TS-3o and TS-3o′ (1.2 kcal mol^−1^).

Hence, whilst this computational result does not fully account for the opposite diastereoselectivity, within computational error it clearly indicates that the presence of an *o*-CH_3_ group on the nitrone greatly affects the stability of the conformers in the TS, and this agrees with the reduced diastereoselectivity for compound 3o (37 : 63 d.r.). Further studies into the effects governing the diastereoselectivity of reactions catalysed by sterically encumbered Lewis acids are still ongoing in our laboratory.

**Scheme 4:**
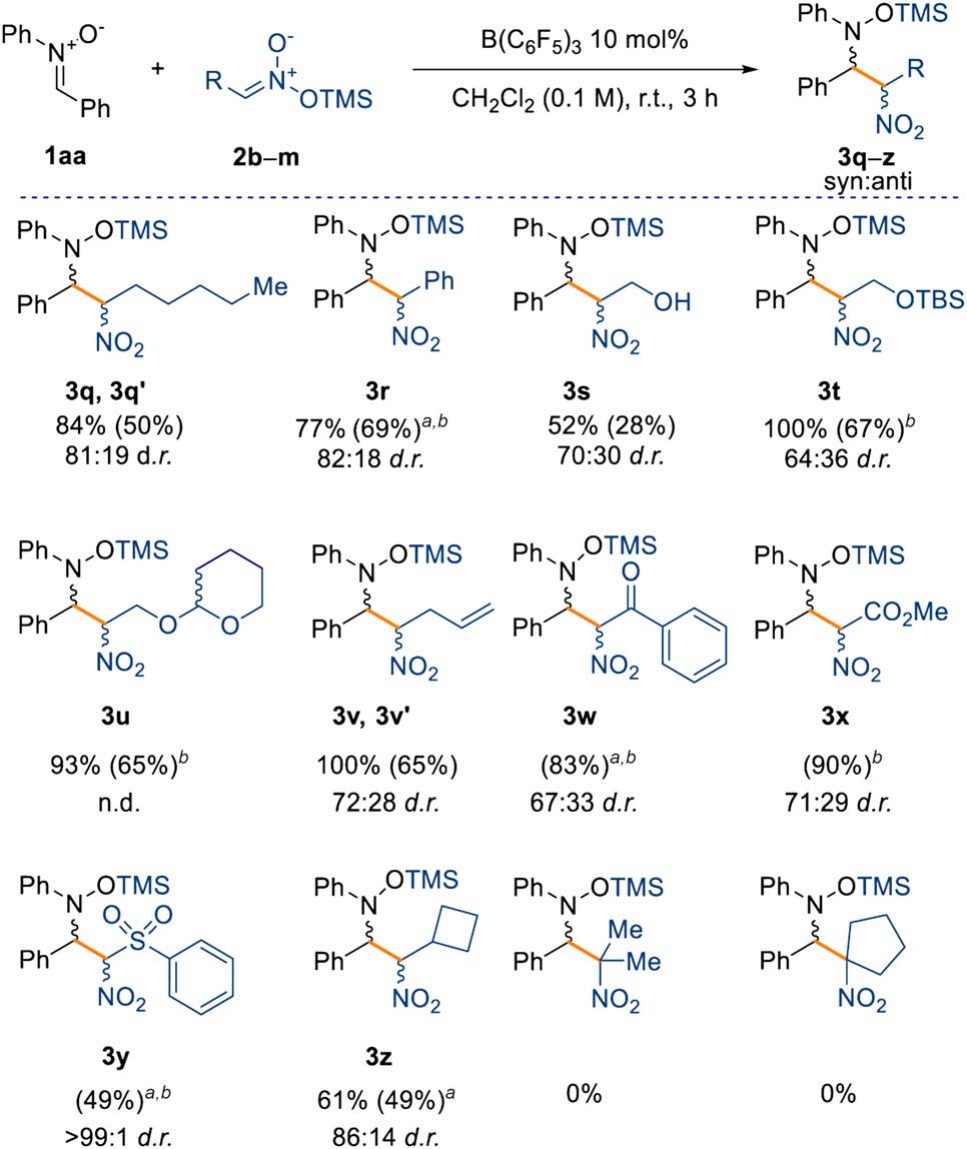
Substrate scope with respect to silyl nitronates 2. Yields refer to the NMR spectroscopic yield of both the major and minor diastereoisomers calculated from the crude reaction using 1 equiv. of 1,3,5-trimethoxybenzene as an internal standard. Isolated yield of the major diastereoisomer in parentheses. All reactions were carried out on a 0.1 mmol scale under the optimised conditions. ^*a*^Reaction carried out for 24 h. ^*b*^Obtained as mixture of diastereoisomers.

Finally, we have also observed that the reaction is scalable, as evidenced from the synthesis of compound 3a, obtained in 90% isolated yield on a gram scale. Having assessed a variety of nitrones for the nitro-Mannich reaction, we then explored the substrate scope with respect to different silyl nitronates (2b–m) with nitrone 1aa (Scheme 4). Increasing the chain length of the silyl nitronate did not alter the reactivity nor the diastereoselectivity as 3q was obtained in 84% NMR yield and in 81 : 19 d.r. Nitromethyl aromatics are also amenable to the B(C_6_F_5_)_3_-catalysed nitro-Mannich reaction, as observed for compound 3r (77% yield, 82 : 18 d.r.). Starting from the TMS (TMS = trimethylsilyl) protected commercially available 2-nitroethanol (2d), we successfully synthesised the alcohol 3s in 52% NMR spectroscopic yield. In this case, partial cleavage of the TMS group occurred during purification on silica, and only the major isomer of 3s was obtained in an unsatisfactory 28% isolated yield. Consequently, we repeated the reaction with a more acid-stable protecting group to better validate the protocol. Pleasingly, the reaction proceeded smoothly affording the –OTBS (TBS = *tert*-butyldimethylsilyl) and –OTHP (THP = 2-tetrahydropyranyl) protected nitro groups, 3t and 3u respectively, in 67% and 65% isolated yield.

**Scheme 5:**
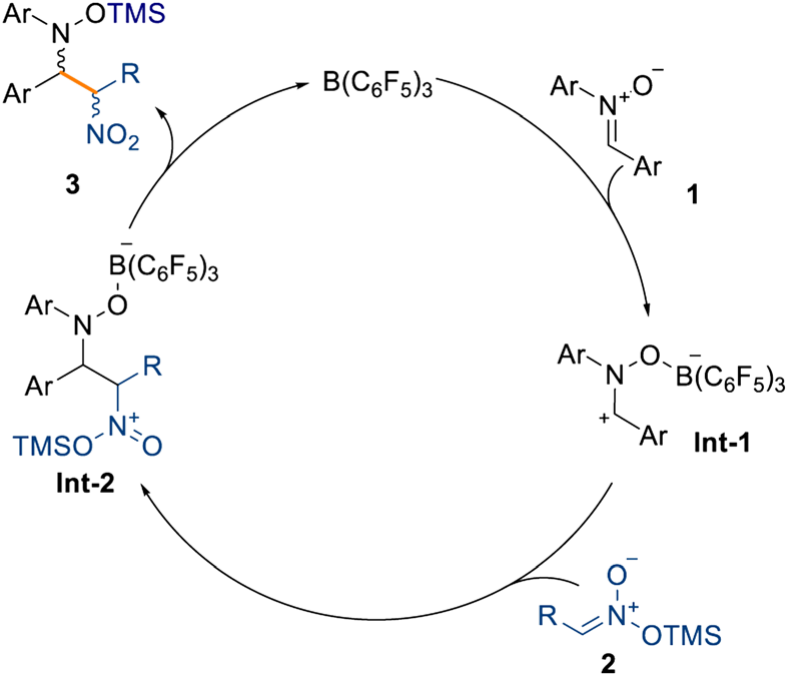
Proposed catalytic cycle for the reaction.

Moreover, the reaction tolerates unsaturated moieties at peripheral positions of the silyl nitronate, as evidenced by compound 3v obtained in quantitative NMR spectroscopic yield. This substrate could potentially engage in a Heck-type coupling in a downstream process. Crucially, we also obtained the α-nitro ketone, ester, and sulfone derivatives (3w–3y) in very good yields (up to 90% isolated yield) and moderate diastereoselectivities (up to 71 : 29). Interestingly, sulfone 3y was obtained without any detectable formation of the minor diastereoisomer. The synthesis of cyclobutyl compound 3z in moderate NMR spectroscopic yields and good diastereoselectivity (61% yield and 86 : 14 d.r.) showed that sp^3^-rich fragments can also be incorporated. The formation of a quaternary carbon center, starting from silyl nitronates 2l and 2m (see ESI†), prevents any reactivity and is the main limitation in this scope. Based on these results and the computational details highlighted in Scheme 3, we propose that the mechanism takes place through initial coordination between the catalyst B(C_6_F_5_)_3_ and the prochiral nitrone 1, affording the zwitterionic intermediate Int-1 (Scheme 5). This possesses enhanced electrophilicity at the α-carbon and can then undergo nucleophilic addition with the silyl nitronate 2, affording Int-2. Lastly, intramolecular silyl migration occurs affording product 3 and reinstating the active catalyst.

**Scheme 6:**
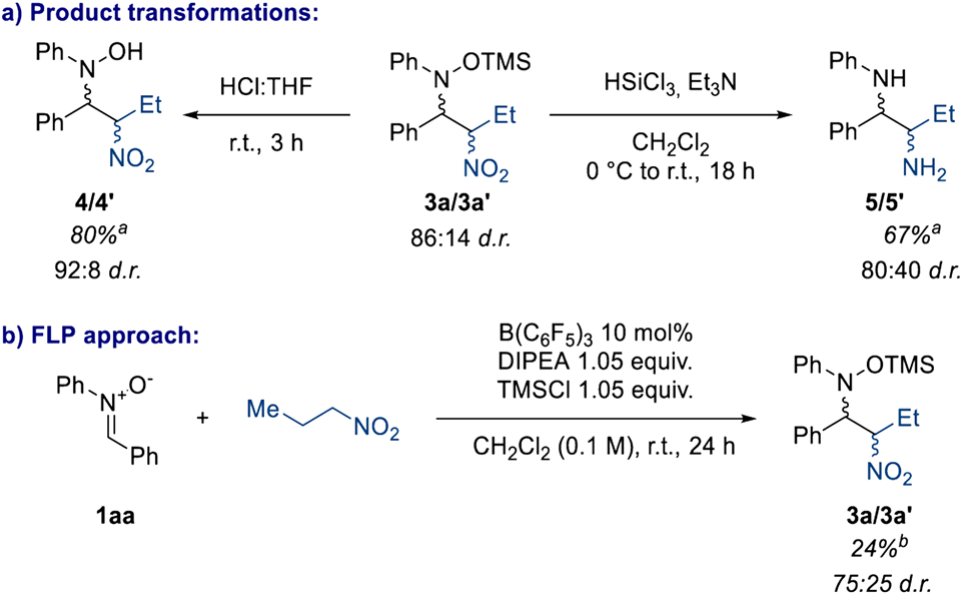
(a) Product manipulation carried out on a 0.2 mmol scale. (b) FLP-type direct nitro-Mannich reaction carried out on a 0.1 mmol scale. ^*a*^Isolated yield. ^*b*^Yield refers to the NMR spectroscopic yield of both the diastereoisomers, calculated from the crude reaction mixture using 1 equiv. of 1,3,5-trimethoxybenzene as an internal standard.

The nitro group possesses an ambiguous position in medicinal chemistry: although it has been proven to have increased genotoxicity and mutagenicity properties,^20^ its synthetic utility is undeniable. The most well-known reactions of the nitro group are the Nef reaction which converts the NO_2_ group to a carbonyl functionality, and NO_2_ reduction to the corresponding amine under several conditions, such as the Béchamp reaction.^12,13,41,42^ Therefore, we screened the product 3a towards different conditions to transform the NO_2_ group to other useful functional groups (Scheme 6a). Firstly, we observed that under very mild conditions the TMS group can be cleaved affording the corresponding nitro hydroxylamine (4/4′) in quantitative yield (80% isolated yield) (Scheme 6a).^43^3a can also be reduced to the corresponding 1,2-diamine (5/5′) and the product can be obtained by a simple aqueous work-up, showing that this methodology could be a useful way to make unsymmetrical 1,2-diamines.

In this way we show that the nitrones employed in this work are readily accessible imine surrogates, which provides an alternative method to the classic nitro-Mannich reaction. Unfortunately, attempts at the Nef reaction under different conditions, such as acidic, reducing, and oxidative were unsuccessful.^44^ We propose that the presence of a nucleophilic group in close proximity to the nitronate intermediate interrupts the Nef reaction affording several side products.^45^

Finally, we were interested in a direct nitro-Mannich reaction, where the silyl nitronate starting material 2 is synthesised *in situ*, as the instability of these compounds pose a limitation to the work. With this in mind, we sought to apply the concept of cooperative catalysis derived from Frustrated Lewis Pair (FLP) chemistry.^46,47^ By mixing nitrone 1aa, 1-nitropropane, B(C_6_F_5_)_3_ and a suitable base we could in principle activate the nitrone with the Lewis acid, and form the nitronate with the base, whilst preventing acid–base inhibition due to the sterics of the catalysts (*cf.* FLP). In this case the FLP system chosen was B(C_6_F_5_)_3_/DIPEA (DIPEA = *N*,*N*-diisopropylethylamine). Pleasingly, preliminary results show that this protocol is possible, generating 3a in 24% yield, and this is currently under exploration in our lab (Scheme 4b).

## Conclusions

In conclusion, this work highlights the ability of B(C_6_F_5_)_3_ to catalyse the nitro-Mannich reaction between nitrones and silyl nitronates in good to high yields (up to 91%) and with good control over the diastereoselectivity (up to >99 : 1) with 10 mol% catalyst loading and under very mild conditions. The reaction is also scalable up to 50 times without any loss in reactivity or diastereoselectivity. The substrate scope (26 examples) shows a high degree of variability of both the nitrone and the silyl nitronate, rendering this protocol amenable to the synthesis of a wide range of products. Crucially, this methodology allows the formation of silyl-protected α-nitro hydroxylamines, which can be easily converted into the corresponding 1,2-diamine or hydroxylamine in high yields. This approach also overcomes previous limitations with the use of imines for the classic nitro-Mannich reaction by replacing them with nitrones. Moreover, it significantly expands the scope of silyl nitronates, which has never been applied to this transformation before. Finally, we have shown that by leveraging the concept of FLPs in cooperative catalysis, we can carry out the direct nitro-Mannich of which we will describe our future endeavours in due course.

## Data availability

The datasets supporting this article have been uploaded as part of the ESI.†

## Author contributions

Conceptualisation: M. G. G.; data curation: M. G. G.; formal analysis: M. G. G.; funding acquisition: T. W., E. R., R. L. M.; investigation: M. G. G.; methodology: M. G. G.; crystal structure analysis: Y. v. I.; DFT calculations: R. B.; writing – original draft: M. G. G.; writing – review and editing: all authors.

## Conflicts of interest

There are no conflicts to declare.

## Supplementary Material

SC-015-D3SC05672D-s001

SC-015-D3SC05672D-s002
