# Correction: An un-forgotten classic: the nitro-Mannich reaction between nitrones and silyl nitronates catalysed by B(C_6_F_5_)_3_

**DOI:** 10.1039/d4sc90113d

**Published:** 2024-06-18

**Authors:** Michael G. Guerzoni, Yara van Ingen, Rasool Babaahmadi, Thomas Wirth, Emma Richards, Rebecca L. Melen

**Affiliations:** a Cardiff Catalysis Institute, School of Chemistry, Cardiff University Translational Research Hub, Maindy Road, Cathays Cardiff CF24 4HQ Cymru/Wales UK RichardsE10@cardiff.ac.uk MelenR@cardiff.ac.uk; b School of Chemistry, Cardiff University Main Building, Park Place Cardiff CF10 3AT Cymru/Wales UK

## Abstract

Correction for ‘An un-forgotten classic: the nitro-Mannich reaction between nitrones and silyl nitronates catalysed by B(C_6_F_5_)_3_’ by Michael G. Guerzoni *et al.*, *Chem. Sci.*, 2024, **15**, 2648–2654, https://doi.org/10.1039/D3SC05672D.

The authors regret that a citation related to a previous publication on the activation of nitrones by boranes is missing in the original article. The full reference is presented herein as ref. 1.

The Royal Society of Chemistry apologises for these errors and any consequent inconvenience to authors and readers.
