# Supplementary material for: An un-forgotten classic: the nitro-Mannich reaction between nitrones and silyl nitronates catalysed by B(C6F5)3
Source: Chem Sci. 2024 Jan 12;15(7):2648–54. doi: 10.1039/d3sc05672d (PMC10866342; doi:10.1039/d3sc05672d)
Supplement: SC-015-D3SC05672D-s001 [file SC-015-D3SC05672D-s001.pdf]

Electronic Supplementary Material (ESI) for Chemical Science.
This journal is © The Royal Society of Chemistry 2024

Electronic Supporting Information

An un-forgotten classic: the nitro-Mannich reaction between nitrones and silyl nitronates catalysed by $B(C_6F_5)_3$

Michael G. Guerzoni,[a] Yara van Ingen,[a] Rasool Babaahmadi,[a] Thomas Wirth,[b] Emma Richards,*[a,b] Rebecca L. Melena,*[b]

[a]Cardiff Catalysis Institute, School of Chemistry, Cardiff University, Translational Research Hub, Maindy Road, Cathays, Cardiff, CF24 4HQ, Cymru/Wales, UK.
[b] School of Chemistry, Cardiff University, Main Building, Park Place, Cardiff, CF10 3AT, Cymru/Wales, UK.
E-mail: RichardsE10@cardiff.ac.uk , MelenR@cardiff.ac.uk

Table of contents

1. General experimental 2
2. Substrate scope table 3
3. Synthesis of starting materials 4
3.1 Nitrones used in this study (GP1): 4
3.2 Synthesis of non-commercial nitro compounds (GP2): 7
3.3 Nitronates used in this study (GP3): 7
4. Optimisation table 12
5. Synthesis of products (GP4): 13
5.1. Structural determination of the minor diastereoisomer: 13
6. Further reactivity: 31
7. NMR Spectra 34
8. Crystallographic data 93
9. Computational details 104
10. References 118

1. General experimental

Except for the nitrone starting materials, all reactions and manipulations were carried out under an atmosphere of dry, O_2-free nitrogen using standard double-manifold techniques with a rotary oil pump. A nitrogen-filled glove box (MBraun) was used to manipulate solids including the storage of starting materials. All solvents (dichloromethane, pentane, acetonitrile, toluene) were dried by employing a Grubbs-type column system (Innovative Technology) or a solvent purification system MB SPS-800 and stored under a nitrogen atmosphere. Dry 1,2-dichloroethane was purchased from Sigma and used as received. Deuterated solvents were distilled and/or dried over molecular sieves before use. Chemicals were purchased from commercial suppliers and used as received. $B(C_6F_5)_3$ was prepared as per the standard literature report.[1] Thin-layer chromatography (TLC) was performed on pre-coated aluminium sheets of Merck silica gel 60 F254 (0.20 mm). ^{1}H, ^{13}C, and ^{19}F NMR spectra were recorded on a Bruker Avance II 400 spectrometer. All coupling constants are absolute values and are expressed in Hertz (Hz). ^{13}C NMR spectra were measured as ^{1}H decoupled. Yields are given as isolated yields of both the diastereoisomers, unless stated otherwise. Chemical shifts are expressed as parts per million (ppm, δ) downfield of tetramethylsilane (TMS) and are referenced to $CDCl_3$ (7.26/77.16 ppm) as internal standard. The description of signals includes s = singlet, d = doublet, t

= triplet, q = quartet, and m = multiplet, br. = broad, dt = doublet of triplets, td = triplet of doublets, dd = doublet of doublets. Proton assignment (determined by 2D NMR experiments: COSY, HSQC and HMBC) where possible. All spectra were analysed assuming a first order approximation. IR-Spectra were measured on a Shimadzu IRAffinity-1 photo-spectrometer. Mass spectra were measured on a Waters LCT Premier/XE or a Waters GCT Premier spectrometer. Ions were generated by Electrospray (ES) or Chemical Ionisation (CI). The molecular ion peaks values are quoted for molecular ion plus hydrogen ($M+H^+$).

2. Substrate scope table

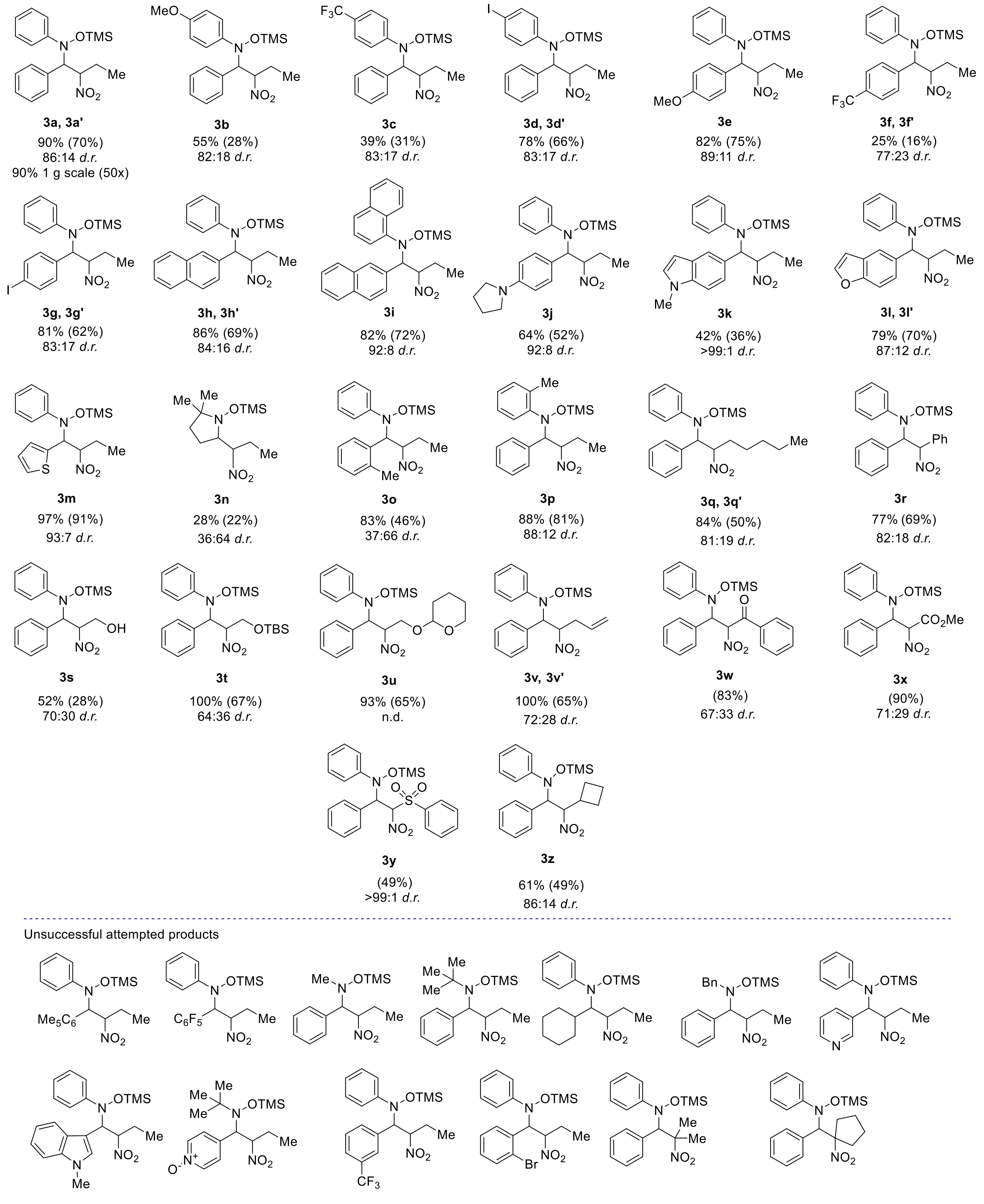

Scheme S1. Products synthesised and attempted products.

3. Synthesis of starting materials

3.1 Nitrones used in this study (GP1):

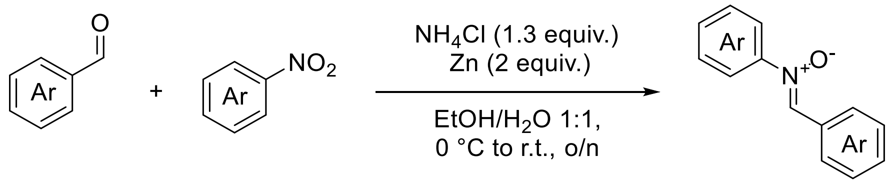

General procedure 1 (GP1): nitroaryl (1 equiv.), aldehyde (1.0 equiv.), and NH_4Cl (1.3 equiv.) were added to a 1:1 mixture of ethanol:water (2 mL/mmol) and the resulting mixture was stirred for 5 minutes at room temperature. The mixture was then cooled to 0 °C, and Zn dust (2 equiv.) was added portion-wise over 10 minutes. Subsequently, the reaction was slowly warmed to room temperature and stirred overnight. The resulting mixture was then filtered through cotton and the organics were extracted using EtOAc (3 × 40 mL), washed with brine (1 × 40 mL), dried over $MgSO_4$, and concentrated *in vacuo*. The crude compound was purified by recrystallisation using ethanol or ethyl acetate. Except for nitrones **1af**, **1aj**, **1ak**, **1aw**, which are described below, all other nitrones are literature-known and their spectra match those reported in the literature.[2–8] Nitrones **1al**, **1as** and **1ax** are commercially available.

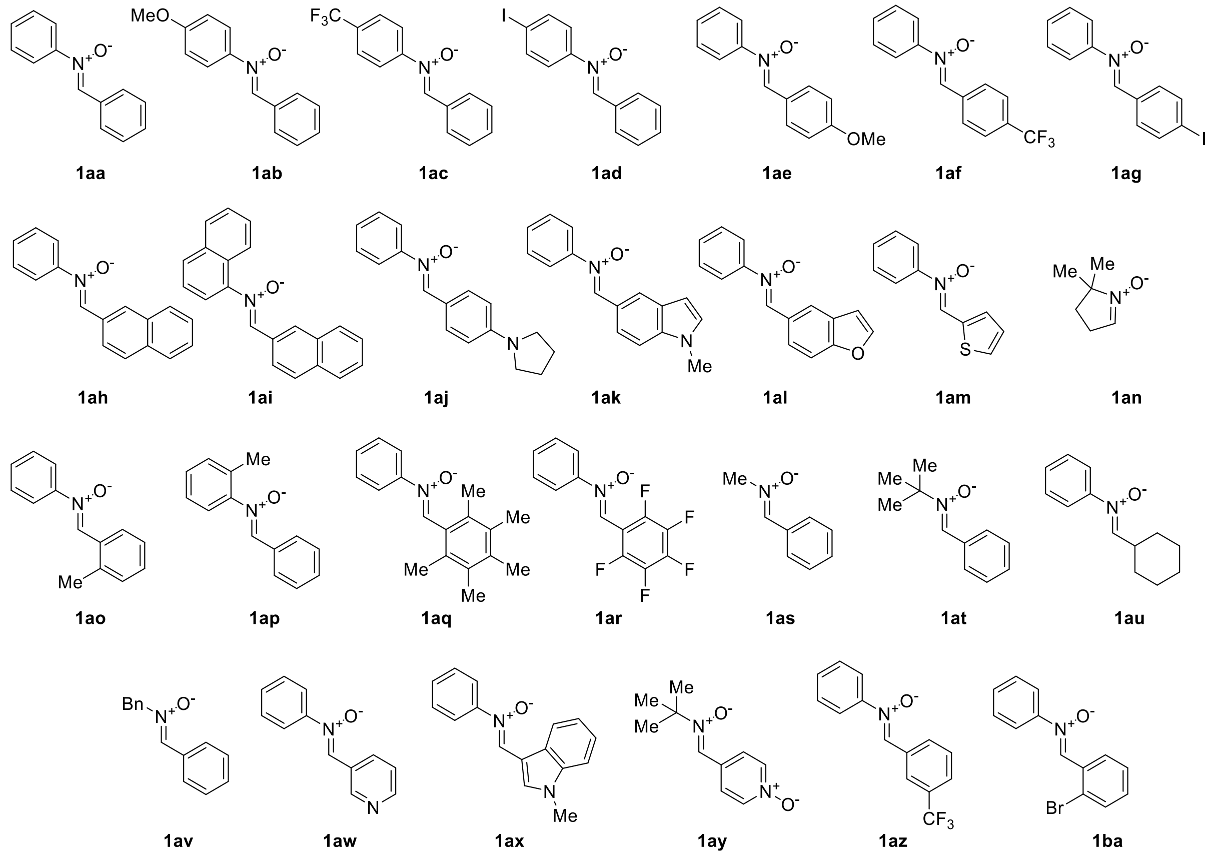

Scheme S2. Starting material nitrones (**1**) used in this study. All nitrones possess (Z)-configuration, although a certain degree of isomerisation might occur in solution for the ones bearing an EWG at the electrophilic carbon.[9]

Synthesis of (Z)-N-phenyl-1-(4-(pyrrolidin-1-yl)phenyl)methanimine oxide **1aj**.

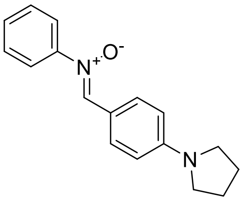

Synthesised according to **GP1** using 4-(pyrrolidin-1-yl)benzaldehyde (2.00 g, 1 equiv.), nitrobenzene (1.16 mL, 1 equiv.), NH_4Cl (0.79 g, 1.3 equiv.) and Zn powder (1.48 g, 2 equiv.) in ethanol:water for 18 hours. Purification of the crude reaction by recrystallisation from EtOH gave nitrone **1aj** as a yellow powder (0.72 g, 24%).

^{1}H-NMR (400 MHz, $CDCl_3$, 298 K) δ: 8.33 (d, *J*=8.9, 1H, Ar–C**H**), 7.78 (m, 2H, Ar–C**H**), 7.45 (m, 1H, Ar–C**H**), 7.40 (m, 1H, Ar–C**H**), 6.60 (d, *J*=9.2, 1H, Ar–C**H**), 3.38 (m, 4H, N–C**H**$_2$–CH_2), 2.04 (m, 4H, N–CH_2–C**H**$_2$).

^{13}C-NMR (101 MHz, $CDCl_3$, 298 K) δ: 149.6 (Ar–**C**), 149.1 (Ar–**C**), 135.2 (**C**H), 131.6 (Ar–**C**), 129.1 (Ar–**C**), 121.6 (Ar–**C**), 118.5 (Ar–**C**), 111.4 (Ar–**C**), 47.7 (N–CH_2–CH_2), 25.6 (N–CH_2–**C**H$_2$).

IR ν_{max} (cm^{-1}): 3042, 2965, 2866, 2837, 1678, 1609, 1593, 1518, 1489, 1456, 1377, 1337, 1300, 1263, 1159, 1136, 1028.

HRMS (ES+): $[M+H]^+$ calculated for $[C_{17}H_{19}N_2O]^+$: 267.1497, found 267.1496.

Synthesis of (Z)-1-(1-methyl-1H-indol-5-yl)-N-phenylmethanimine oxide **1ak**.

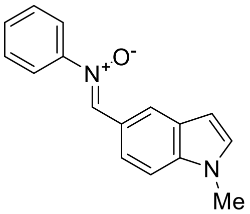

Synthesised according to **GP1** using 1-methyl-1H-indole-5-carbaldehyde (0.5 g, 1 equiv.), nitrobenzene (0.39 mL, 1 equiv.), NH_4Cl (0.22 g, 1.3 equiv.) and Zn powder (0.41 g, 2 equiv.) in ethanol:water for 2 hours. Purification of the crude reaction by recrystallisation from EtOH gave nitrone **1ak** as an orange powder (0.42 g, 54%).

^{1}H-NMR (400 MHz, $CDCl_3$, 298 K) δ: 9.06 (d, *J*=1.6, 1H, Ar–C**H**), 8.10 (d, *J*=1.6, 1H, Ar–C**H**), 8.03 (s, 1H, C**H**), 7.88–7.78 (m, 2H, Ar–C**H**), 7.54–7.44 (m, 3H, Ar–C**H**), 7.40 (dt, *J*=8.7, 0.8, 1H, Ar–C**H**), 7.11 (d, *J*=3.1, 1H, C2**H**), 6.61 (dd, *J*=3.1, 0.9, 1H, C3**H**), 3.84 (s, 3H).

^{13}C-NMR (101 MHz, $CDCl_3$, 298 K) δ: 149.4 (Ar–**C**), 138.1 (Ar–**C**), 136.2 (**C**H), 130.2 (**C**1), 129.5 (Ar–**C**), 129.2 (Ar–**C**), 128.5(Ar–**C**), 123.8 (Ar–**C**), 123.3(Ar–**C**), 122.6 (Ar–**C**), 121.9 (Ar–**C**), 109.4 (Ar–**C**), 102.9 (**C**2), 33.1 (**C**H$_3$).

IR ν_{max} (cm^{-1}): 3096, 3061, 2941, 1672, 1607, 1555, 1489, 1451, 1424, 1400, 1368, 1344, 1304, 1244, 1190, 1148, 1103, 1063, 1024.

HRMS (ES+): $[M+H]^+$ calculated for $[C_{16}H_{15}N_2O]^+$: 251.1184, found 251.1189.

Synthesis of (Z)-1-(benzofuran-5-yl)-N-phenylmethanimine oxide **1al**.

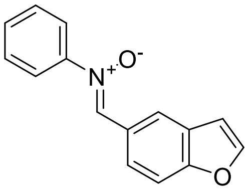

Synthesised according to **GP1** using 1-benzofuran-5-carbaldehyde (0.73 g, 1 equiv.), nitrobenzene (0.51 mL, 1 equiv.), NH_4Cl (0.35 g, 1.3 equiv.) and Zn powder (0.65 g, 2 equiv.) in ethanol:water for 18 hours. Purification of the crude reaction by recrystallisation from EtOAc gave nitrone **1al** as a light orange solid (0.36 g, 30%).

^{1}H-NMR (400 MHz, $CDCl_3$, 298 K) δ: 9.18 (d, *J*=1.7, 1H, Ar–C**H**), 8.04–7.98 (m, 2H, Ar–C**H**+ C**H**), 7.85–7.76 (m, 2H, Ar–C**H**), 7.68 (d, *J*=2.2, 1H, C1**H**), 7.57 (m, 1H, Ar–C**H**), 7.54–7.45 (m, 3H, Ar–C**H**), 6.86 (dd, *J*=2.2, 1.0, 1H, C2**H**).

^{13}C-NMR (101 MHz, CDCl$_3$, 298 K) δ: 156.1 (Ar–**C**), 149.2 (Ar–**C**), 146.2 (Ar–**C**), 135.0 (**C**H), 129.9 (Ar–**C**), 129.3 (Ar–**C**), 127.9 (Ar–**C**), 126.6 (**C**1), 126.0 (Ar–**C**), 122.5 (Ar–**C**), 121.9 (Ar–**C**), 111.8 (Ar–**C**), 107.4 (**C**2).

IR ν$_{max}$ (cm^{-1}): 3104, 3057, 1591, 1557, 1487, 1458, 1439, 1397, 1344, 1325, 1269, 1211, 1190, 1146, 1125, 1109, 1065, 1024.

HRMS (ES+): [M+H]$^+$ calculated for [$C_{15}H_{12}NO_2$]$^+$: 238.0868, found 238.0866.

Synthesis of (Z)-1-(2,3,4,5,6-pentamethylphenyl)-N-phenylmethanimine oxide **1aq**.

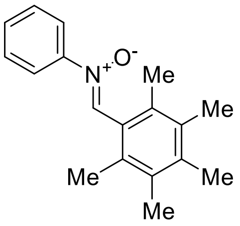

Synthesised according to **GP1** using 2,3,4,5,6-pentamethylbenzaldehyde (0.88 g, 1 equiv.), nitrobenzene (0.51 mL, 1 equiv.), NH_4Cl (0.35 g, 1.3 equiv.) and Zn powder (0.65 g, 2 equiv.) in ethanol:water for 24 hours. Purification of the crude reaction by recrystallisation from EtOH gave nitrone **1aq** as a white powder (0.15 g, 11%).

^{1}H-NMR (400 MHz, CDCl$_3$, 298 K) δ: 8.15 (s, 1H, C**H**), 7.84–7.78 (m, 2H, Ar–C**H**), 7.58–7.43 (m, 2H, Ar–C**H**), 2.27 (s, 6H, C**H**$_3$), 2.26 (s, 3H, C**H**$_3$), 2.24 (s, 6H, C**H**$_3$).

^{13}C-NMR (101 MHz, CDCl$_3$, 298 K) δ: 148.7 (Ar–**C**), 137.1 (**C**H), 133.0 (Ar–**C**), 132.7 (Ar–**C**), 130.2(Ar–**C**), 129.3 (Ar–**C**), 129.3 (Ar–**C**), 126.5(Ar–**C**), 122.2 (Ar–**C**), 17.6 (**C**H$_3$), 17.1 (**C**H$_3$), 16.5 (**C**H$_3$).

IR ν$_{max}$ (cm^{-1}): 3057, 2938, 1587, 1541, 1487, 1458, 1385, 1371, 1296, 1192, 1105, 1057, 1022, 1001.

HRMS (ES+): [M+H]$^+$ calculated for [$C_{18}H_{22}NO$]$^+$: 268.1701, found 268.1706.

Synthesis of (Z)-1-(1-methyl-1H-indol-3-yl)-N-phenylmethanimine oxide **1ax**.

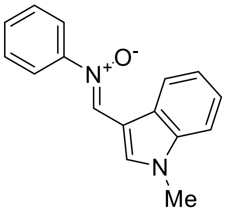

Synthesised according to **GP1** using 1-methyl-1H-indole-3-carbaldehyde (2.00 g, 1 equiv.), nitrobenzene (1.3 mL, 1 equiv.), NH_4Cl (0.87 g, 1.3 equiv.) and Zn powder (1.63 g, 2 equiv.) in ethanol:water for 24 hours. Purification of the crude reaction by recrystallisation from EtOH gave nitrone **1ax** as a white powder (0.61 g, 20%).

^{1}H-NMR (400 MHz, CDCl$_3$, 298 K) δ: 9.17 (s, 1H, C**H**), 8.35 (d, *J*=0.7, 1H, C1**H**), 7.92–7.83 (m, 2H, Ar–C**H**), 7.76 (dt, *J*=7.9, 1.0, 1H, Ar–C**H**), 7.58–7.47 (m, 2H, Ar–C**H**), 7.47–7.40 (m, 2H, Ar–C**H**), 7.35 (ddd, *J*=8.2, 7.0, 1.2, 1H, Ar–C**H**), 7.27 (ddd, *J*=8.0, 7.0, 1.1, 1H, Ar–C**H**), 3.90 (s, 3H, C**H**$_3$).

^{13}C-NMR (101 MHz, CDCl$_3$, 298 K) δ: 148.0 (Ar–**C**), 136.9 (**C**2), 134.4 (**C**H), 129.2 (Ar–**C**), 129.1 (Ar–**C**), 127.7 (**C**1), 127.2 (Ar–**C**), 123.3 (Ar–**C**), 121.3 (Ar–**C**), 121.2 (Ar–**C**), 118.2 (Ar–**C**), 110.3 (Ar–**C**), 107.5 (Ar–**C**), 33.6 (**C**H$_3$).

IR ν$_{max}$ (cm^{-1}): 3121, 3055, 3007, 1676, 1655, 1601, 1570, 1535, 1514, 1474, 1431, 1379, 1346, 1325, 1244, 1192, 1175, 1130, 1121, 1074, 1059, 1028, 1013.

HRMS (ES+): [M+H]$^+$ calculated for [$C_{16}H_{15}N_2O$]$^+$: 251.1184, found 251.1190.

3.2 Synthesis of non-commercial nitro compounds (GP2):

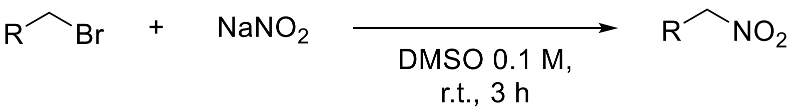

General procedure 2 (GP2): Under air, the bromo starting material (1 equiv.) was dissolved in DMSO (0.1 M). To this solution, $NaNO_2$ (2 equiv.) was added portion wise over the course of 5 minutes and the reaction mixture was left to stir for 3 hours at room temperature. Subsequently, the reaction was quenched with ice and extracted several times with Et_2O. The organic layers were then collected and dried over $MgSO_4$ and the solvent was removed under a stream of compressed air. The remaining oil was then purified with a static vacuum-short path distillation to afford the nitro compound which was then used for the next step without further purification.

Synthesis of 4-nitrobut-1-ene.

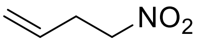

Synthesised according to **GP2** using 4-bromobut-1-ene (1 equiv., 2.00 mL) and $NaNO_2$ (2 equiv., 2.70 g) in DMSO for 3 hours. After distillation, *4-nitrobut-1-ene* was obtained as a colourless oil (0.48 g, 24%).[10]

Synthesis of (nitromethyl)cyclobutane.

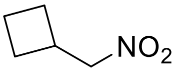

Synthesised according to **GP2** using (bromomethyl)cyclobutane (1 equiv., 2.00 mL) and $NaNO_2$ (2 equiv., 2.50 g) in DMSO for 3 hours. After distillation, 0.41 mg of *(nitromethyl)cyclobutane* were obtained as a yellow oil containing 37% of unreacted (bromomethyl)cyclobutene and DMSO.

^{1}H-NMR (400 MHz, $CDCl_3$, 298 K) δ: 4.38 (d, *J*=7.6, 2H α$C\mathbf{H}_2$), 3.01 (tt, *J*=10.2, 6.6, 1H, C**H**), 2.05–1.64 (m, 6H, $C\mathbf{H}_2$).

^{13}C-NMR (101 MHz, $CDCl_3$, 298 K) δ: 80.2 (αCH_2), 33.5 (**C**H), 27.3 (**C**H_2), 25.8 (**C**H_2), 18.2 (**C**H_2), 17.0 (**C**H_2).

IR ν_{max} (cm^{-1}): 2255, 1638, 1549, 1375, 1319, 1015.

HRMS (EI): $[M]^+$ calculated for $[C_5H_9NO_2]^+$: 115.06278, found 115.0628.

3.3 Nitronates used in this study (GP3):

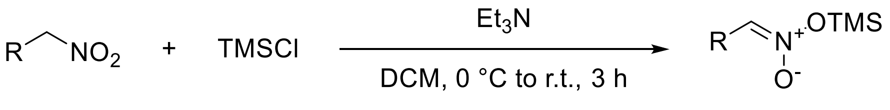

General procedure 3 (GP3): The nitro compound (1 equiv.) and TMSCl (1.05 equiv.) were dissolved in anhydrous CH_2Cl_2 (0.05 M) and cooled to 0 °C. To the resulting mixture freshly distilled Et_3N (1.05 equiv.) was added in one portion and the solution was left to stir at 0 °C for 15 minutes. Then, the ice bath was removed, and the reaction was left to stir for 3 h at room temperature. Subsequently, the solvent was removed using a secondary trap and the remaining white solid was washed three times with anhydrous pentane. Each washing was transferred to a dry vessel by filter cannula. The pentane was subsequently removed using a secondary trap and the leftover yellow oil was then transferred

inside a nitrogen-filled glovebox and stored in a -38 °C freezer in the dark. Due to the reported[11] instability of the silyl nitronates towards light, air and temperature, full characterisation was not possible and only ^{1}H-NMR and ^{13}C-NMR spectra are given. The configuration of all the silyl nitronates has been assigned based on the *J* value which is consistent with a *cis* (or (*E*)) configuration. In the case of **2l** and **2m**, we believe that the TMS group sits between the two negatively charged oxygen atoms, giving rise to a symmetric molecule which yields only 2 peaks for compound **2l** and 3 peaks for compound **2m**.

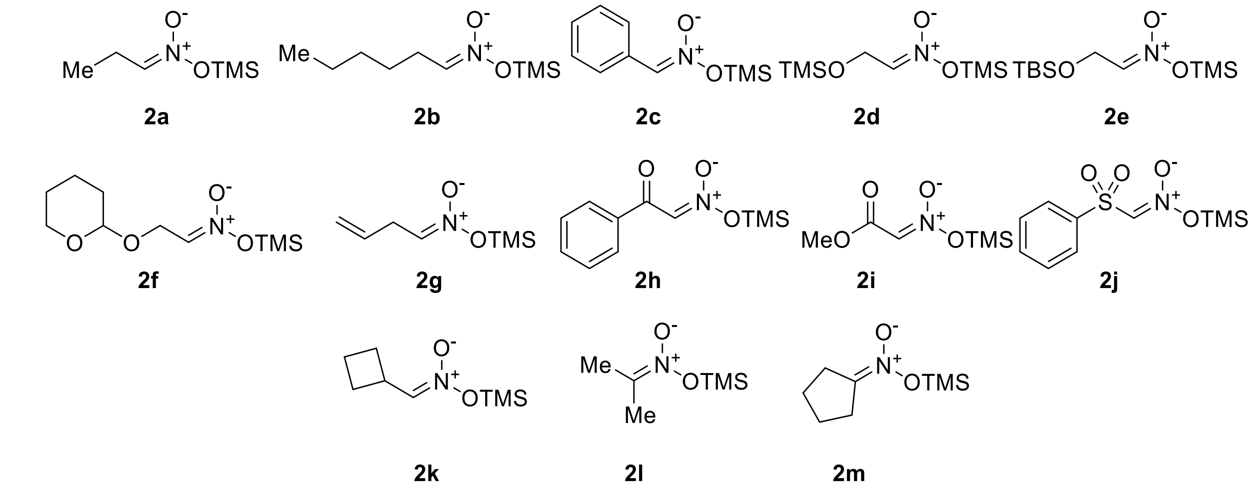

Scheme S3. Nitronates (**2**) used in this study.

Synthesis of trimethylsilyl (E)-propylideneazinate **2a**.

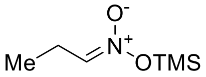

Synthesised according to **GP3** using 1-nitropropane (1 equiv., 1.00 mL), TMSCl (1.05 equiv., 1.63 mL) and Et_3N (1.05 equiv., 1.91 mL) in dichloromethane for 3 hours. After removal of the solvent, compound **2a** was obtained as a pale yellow oil (0.78g, 43%).

^{1}H-NMR (400 MHz, $CDCl_3$, 298 K) δ: 6.09 (t, *J*=6.3, 1H, C**H**), 2.31 (qd, *J*=7.6, 6.3, 2H, C**H**$_2$), 1.08 (t, *J*=7.7, 3H, C**H**$_3$), 0.31 (s, 9H, TMS–C**H**$_3$).

^{13}C-NMR (101 MHz, $CDCl_3$, 298 K) δ: 118.4 (**C**H), 20.1 (**C**H$_2$), 10.4 (**C**H$_3$), 0.1 (TMS–**C**H$_3$).

Synthesis of trimethylsilyl (E)-hexylideneazinate **2b**.

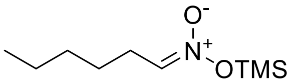

Synthesised according to **GP3** using 1-nitrohexane (1.0 equiv., 1.00 g), TMSCl (1.05 equiv., 1.02 mL) and Et_3N (1.05 equiv., 1.12 mL) in dichloromethane for 3 hours. After removal of the solvent, compound **2b** was obtained as a yellow oil (1.15 g, 74%).

^{1}H-NMR (400 MHz, $CDCl_3$, 298 K) δ: 6.09 (t, *J*=6.4, 1H, C**H**), 2.28 (td, *J*=7.5, 6.5, 2H, C**H**$_2$), 1.48 (m, 2H, C**H**$_2$), 1.36–1.23 (m, 4H, C**H**$_2$), 0.94–0.81 (m, 3H, C**H**$_3$), 0.30 (s, 9H, TMS–C**H**$_3$).

^{13}C-NMR (101 MHz, $CDCl_3$, 298 K) δ: 117.5 (**C**H), 31.6 (**C**H$_2$), 26.5 (**C**H$_2$), 25.7 (**C**H$_2$), 22.5 (**C**H$_2$), 14.1 (**C**H$_2$), 0.1 (TMS–**C**H$_3$).

Synthesis of trimethylsilyl (E)-benzylideneazinate **2c**.

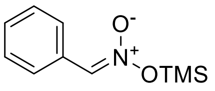

Synthesised according to **GP3** using (nitromethyl)benzene (1 equiv., 0.42 mL), TMSCl (1.05 equiv., 0.54 mL) and Et_3N (1.05 equiv., 0.59 mL) in dichloromethane for 3 hours. After removal of the solvent, compound **2c** was obtained as a yellow oil (0.55 g, 72%).

^{1}H-NMR (400 MHz, CDCl$_3$, 298 K) δ: 7.90–7.83 (m, 2H, Ar–C**H**), 7.43–7.30 (m, 3H, Ar–C**H**), 7.03 (s, 1H, C**H**), 0.38 (s, 9H, TMS–C**H**$_3$).

^{13}C-NMR (101 MHz, CDCl$_3$, 298 K) δ: 129.6 (Ar–CH), 129.3 (Ar–**C**H), 128. (Ar–**C**H)7, 127.5 (Ar–**C**H), 116.4 (**C**H), 0.1 (TMS–**C**H$_3$).

Synthesis of trimethylsilyl (E)-(2-((trimethylsilyl)oxy)ethylidene)azinate **2d**.

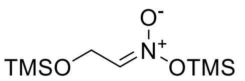

Synthesised according to **GP3** using 2-nitroethan-1-ol (1 equiv., 0.50 mL), TMSCl (2.1 equiv., 1.78 mL) and Et$_3$N (2.1 equiv., 1.94 mL) in dichloromethane for 3 hours. After removal of the solvent, compound **2d** was obtained as a yellow oil (0.96 g, 58%).

^{1}H-NMR (400 MHz, CDCl$_3$, 298 K) δ: 6.23 (t, *J*=5.3, 1H, C**H**), 4.35 (d, *J*=5.3, 2H, C**H**$_2$), 0.29 (s, 9H, TMS–C**H**$_3$), 0.12 (s, 9H, TMS–C**H**$_3$).

^{13}C-NMR (101 MHz, CDCl$_3$, 298 K) δ: 116.7 (**C**H), 57.8 (**C**H$_2$), 0.1 (TMS–**C**H$_3$), -0.5 (TMS–**C**H$_3$).

Synthesis of trimethylsilyl (E)-(2-((tert-butyldimethylsilyl)oxy)ethylidene)azinate **2e**.

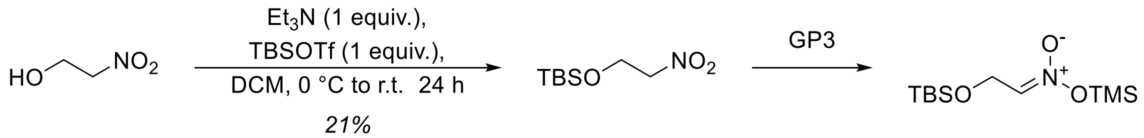

Synthesised according to **GP3** using tert-butyldimethyl(2-nitroethoxy)silane[12] (1 equiv., 0.59 g), TMSCl (1.05 equiv., 0.38 mL) and Et$_3$N (1.05 equiv., 0.42 mL) in dichloromethane for 3 hours. After removal of the solvent, compound **2e** was obtained as a yellow oil (0.61 g, 77%).

^{1}H-NMR (400 MHz, CDCl$_3$, 298 K) δ: 6.24 (t, *J*=5.1, 1H, C**H**), 4.40 (d, *J*=5.1, 3H, C**H**$_2$), 0.89 (s, 9H, TBS–C**H**$_3$), 0.31 (s, 9H, TMS–C**H**$_3$), 0.08 (s, 6H, TBS–C**H**$_3$).

^{13}C-NMR (101 MHz, CDCl$_3$, 298 K) δ: 117.2 (**C**H), 58.5 (**C**H$_2$), 25.9 (TBS–CH$_3$), 18.4 (TBS–**C**), 0.1 (TMS–**C**H$_3$), -5.3 (TBS–**C**H$_3$).

Synthesis of trimethylsilyl (E)-(2-((tert-butyldimethylsilyl)oxy)ethylidene)azinate **2f**.

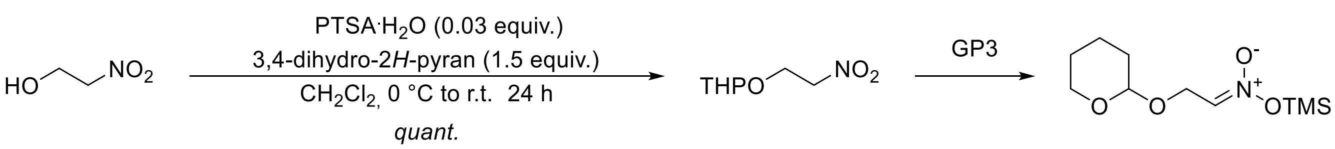

Synthesised according to **GP3** using 2-(2-nitroethoxy)tetrahydro-2*H*-pyran[12] (1 equiv., 0.50 g), TMSCl (1.05 equiv., 0.38 mL) and Et$_3$N (1.05 equiv., 0.42 mL) in dichloromethane for 3 hours. After removal of the solvent, compound **2f** was obtained as a yellow oil (0.47 g, 67%).

^{1}H-NMR (400 MHz, CDCl$_3$, 298 K) δ: 6.31 (t, *J*=5.6, 1H, C**H**), 4.64 (m, 1H, O–C**H**–O), 4.39–4.28 (m, 2H, C**H**$_2$), 3.87–3.81 (m, 1H, O–C**H**$_2$), 3.54–3.49 (m, 1H, O–C**H**$_2$), 1.83–1.48 (m, 6H, C**H**$_2$), 0.30 (s, 9H, TMS–C**H**$_3$).

^{13}C-NMR (101 MHz, CDCl$_3$, 298 K) δ: 114.6 (**C**H), 99.2 (O–CH–O), 62.5 (**C**H$_2$), 61.8 (O–**C**H$_2$), 30.5 (**C**H$_2$), 25.4 (**C**H$_2$), 19.4 (**C**H$_2$), 0.0 (TMS–**C**H$_3$).

Synthesis of trimethylsilyl (E)-but-3-en-1-ylideneazinate **2g**.

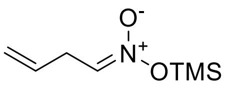

Synthesised according to **GP3** using *4-nitrobut-1-ene* (1 equiv., 0.48 g), TMSCl (1.05 equiv., 0.63 mL) and Et_3N (1.05 equiv., 0.69 mL) in dichloromethane for 3 hours. After removal of the solvent, compound **2g** was obtained as a yellow oil (0.45 g, 94%).

^{1}H-NMR (400 MHz, $CDCl_3$, 298 K) δ: 6.12 (td, *J*=6.3, 1.1, 1H, C**H**), 5.81–5.71 (m, 1H, vinylic C**H**), 5.15–5.07 (m, 2H, vinylic C**H**$_2$), 3.01 (m, 2H, C**H**$_2$), 0.28 (s, 9H, TMS–C**H**$_3$).

^{13}C-NMR (101 MHz, $CDCl_3$, 298 K) δ: 131.3 (**C**H), 117.7 (vinylic **C**H$_2$), 114.6 (vinylic **C**H), 30.7 (**C**H$_2$), 0.0, (TMS–**C**H$_3$).

Synthesis of trimethylsilyl (E)-(2-oxo-2-phenylethylidene)azinate **2h**.

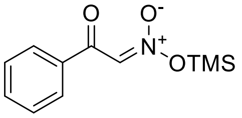

Synthesised according to **GP3** using 2-nitro-1-phenylethan-1-one (1 equiv., 1.00 g), TMSCl (1.05 equiv., 0.81 mL) and Et_3N (1.05 equiv., 0.89 mL) in dichloromethane for 3 hours. After removal of the solvent, compound **2h** was obtained as a yellow oil (0.19 g, 13%).

^{1}H-NMR (400 MHz, $CDCl_3$, 298 K) δ: 7.57–7.38 (m, 6H, Ar–C**H**+C**H**), 0.26 (s, 9H, TMS–C**H**$_3$).

^{13}C-NMR (101 MHz, $CDCl_3$, 298 K) δ: 160.1 (**C**=O), 134.4 (Ar–**C**), 132.0 (Ar–**C**), 129.0 (Ar–**C**), 127.1 (Ar–**C**), 121.9 (**C**H), 0.8 (TMS–**C**H$_3$).

Synthesis of methyl (E)-2-(oxido((trimethylsilyl)oxy)azaneylidene)acetate **2i**.

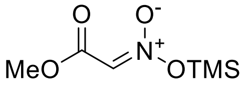

Synthesised according to **GP3** using methyl 2-nitroacetate (1 equiv., 0.39 mL), TMSCl (1.05 equiv., 0.56 mL) and Et_3N (1.05 equiv., 0.61 mL) in dichloromethane for 18 hours. After removal of the solvent, compound **2i** was obtained as a yellow oil (0.32 g, 40%).

^{1}H-NMR (400 MHz, $CDCl_3$, 298 K) δ: 6.69 (s, 1H, C**H**), 3.77 (s, 3H, OC**H**$_3$), 0.32 (s, 9H, TMS–C**H**$_3$).

^{13}C-NMR (101 MHz, $CDCl_3$, 298 K) δ: 161.1 (**C**=O), 107.2 (**C**H), 52.1 (O**C**H$_3$), -0.3 (TMS–**C**H$_3$).

Synthesis of trimethylsilyl (E)-((phenylsulfonyl)methylene)azinate **2j**.

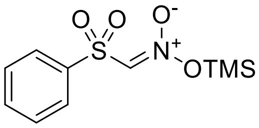

Synthesised according to **GP3** using nitromethyl phenyl sulfone (1.0 equiv., 0.50 g), TMSCl (1.05 equiv., 0.33 mL) and Et_3N (1.05 equiv., 0.36 mL) in dichloromethane for 3 hours. After removal of the solvent, compound **2j** was obtained as a yellow oil (0.12 g) and used as is without further purification.

^{1}H-NMR (400 MHz, $CDCl_3$, 298 K) δ: 8.03–7.97 (m, 2H, Ar–C**H**), 7.64–7.50 (m, 3H, Ar–C**H**), 7.21 (br s, 1H, C**H**), 0.24 (s, 9H, TMS–C**H**$_3$).

^{13}C-NMR (101 MHz, $CDCl_3$, 298 K) δ: 134.9 (Ar–**C**), 129.6 (Ar–**C**), 129.4 (Ar–**C**), 129.0 (Ar–**C**), 124.4 (**C**H), -0.5 (TMS–**C**H$_3$).

Synthesis of trimethylsilyl (E)-(cyclobutylmethylene)azinate **2k**.

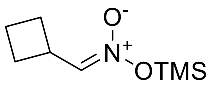

Synthesised according to **GP3** using *(nitromethyl)cyclobutane* (1.0 equiv., 0.42 g), TMSCl (1.05 equiv., 0.49 mL) and Et_3N (1.05 equiv., 0.53 mL) in dichloromethane for 3 hours. After removal of the solvent the yellow oil was also distilled, affording compound **2k** as a yellow oil (0.15 g).

^{1}H-NMR (400 MHz, $CDCl_3$, 298 K) δ: 6.16 (d, *J*=7.0, 1H, C**H**), 3.41–3.31 (m, 1H, C**H**), 2.29–2.21 (m, 2H, C**H**$_2$), 2.01–1.84 (m, 4H, C**H**$_2$), 0.28 (s, 9H, TMS–C**H**$_3$).

^{13}C-NMR (101 MHz, $CDCl_3$, 298 K) δ: 120.8 (**C**H), 32.2 (**C**H), 26.9(**C**H$_2$), 19.3 (**C**H$_2$), 0.1 (TMS–**C**H$_3$).

Synthesis of trimethylsilyl propan-2-ylideneazinate **2l**.

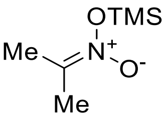

Synthesised according to **GP2** using 2-nitropropane (1equiv., 0.51 mL), TMSCl (1.05 equiv., 0.75 mL) and Et_3N (1.05 equiv., 0.82 mL) in dichloromethane for 18 hours. After removal of the solvent, compound **2l** was obtained as a yellow oil (0.65 g, 87%).

For this compound, only one CH_3 peak could be seen due to the rapid intramolecular exchange of the trimethylsilyl group between the two oxygen atoms.[13]

^{1}H-NMR (400 MHz, $CDCl_3$, 298 K) δ: 1.97 (s, 6H, C**H**$_3$), 0.28 (s, 9H, TMS–C**H**$_3$).

^{13}C-NMR (101 MHz, $CDCl_3$, 298 K) δ: 121.4 (**C**), 18.5 (**C**H$_3$), 0.4 (TMS–**C**H$_3$).

Synthesis of trimethylsilyl cyclopentylideneazinate **2m**.

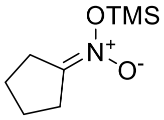

Synthesised according to **GP3** using nitrocyclopentane (1 equiv., 0.25 mL), TMSCl (1.05 equiv., 0.31 mL) and Et_3N (1.05 equiv., 0.35 mL) in dichloromethane for 18 hours. After removal of the solvent, compound **2m** was obtained as a green oil (0.36 g, 82%).

For this compound, only two CH_2 peaks could be seen due to the rapid intramolecular exchange of the trimethylsilyl group between the two oxygen atoms.[13]

^{1}H-NMR (400 MHz, $CDCl_3$, 298 K) δ: 2.49–2.46 (m, 4H, C**H**$_2$), 1.79–1.75 (m, 4H, C**H**$_2$), 0.31–0.02 (m, 9H, TMS–C**H**$_3$).

^{13}C-NMR (101 MHz, $CDCl_3$, 298 K) δ: 132.5 (**C**), 30.0 (**C**H$_2$), 25.6 (**C**H$_2$), 0.4(TMS–**C**H$_3$).

4. Optimisation table

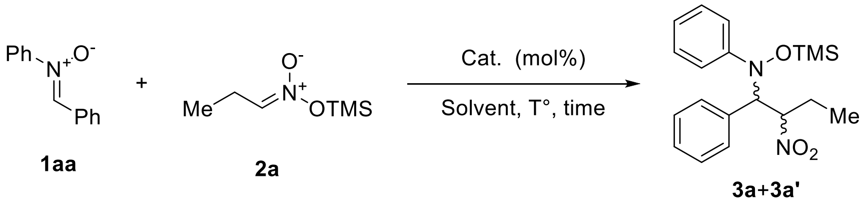

Table S1. Optimisation of the reaction conditions.

Entry	Cat (mol%)	Solvent (0.1 M)	T (°C)	Time (h)	NMR Yield (Isolated)	*d.r.* (syn:anti)	Conversion
1	$B(C_6F_5)_3$ (20)	Toluene	r.t.	5.5	80% (59%)	85:15	94%
2	-	Toluene	r.t.	5.5	0%	n.a.	0%
3	$B(C_6F_5)_3$ (20)	CH_2Cl_2	r.t.	6	73%	85:15	92%
4	$B(C_6F_5)_3$ (20)	$C_2H_4Cl_2$	r.t.	6	44%	86:14	48%
5	$B(C_6F_5)_3$ (20)	Trifluorotoluene	r.t.	6	80% (62%)	85:15	88%
6	$B(C_6F_5)_3$ (20)	THF	r.t.	6	71%	78:22	88%
7	$B(C_6F_5)_3$ (20)	Pentane	r.t.	6	82% (68%)	87:13	82%
8	$B(C_6F_5)_3$ (20)	Et_2O	r.t.	6	45%	83:17	55%
9	$B(C_6F_5)_3$ (20)	MeCN	r.t.	6	decomp.	n.a.	100%
10	$B(C_6F_5)_3$ (20)	neat	r.t.	6	67%	86:14	90%
11	-	Toluene	r.t.	24	traces	n.a.	<5%
12	$B(C_6F_5)_3 \cdot H_2O$ (20)	Pentane	r.t.	6	traces	n.a.	<5%
13[a]	$B(C_6F_5)_3$ (20)	Pentane	r.t.	6	61%	87:13	91%
14	$TsOH \cdot H_2O$ (20)	Pentane	r.t.	6	Decomp.	n.a.	<5%
15	$B(C_6F_5)_3$ (20)	Pentane	r.t.	0.5	34%	78:22	58%
16	$B(C_6F_5)_3$ (20)	CH_2Cl_2	r.t.	3	77%	86:14	94%
17	$B(C_6F_5)_3$ (20)	TFT	r.t.	3	69%	84:16	94%
18	$B(C_6F_5)_3$ (20)	Pentane	r.t.	3	59%	86:14	84%
19[b]	$B(C_6F_5)_3$ (20)	CH_2Cl_2	r.t.	3	84%	86:14	>95%
20[b]	$B(C_6F_5)_3$ (20)	CH_2Cl_2	r.t.	1	79%	86:14	94%
21[b]	$B(C_6F_5)_3$ (20)	CH_2Cl_2	0 °C	3	79%	88:12	89%
22[b]	$B(C_6F_5)_3$ (20)	CH_2Cl_2	-41	3	54%	74:26	82%
23[b]	$B(C_6F_5)_3$ (20)	CH_2Cl_2	-78	3	44%	76:24	67%
24[b]	$B(C_6F_5)_3$ (10)	CH_2Cl_2	r.t.	3	90% (81%)	86:14	100%
25[b]	$B(C_6F_5)_3$ (5)	CH_2Cl_2	r.t.	3	71%	86:14	83%
26[b]	BPh_3 (10)	CH_2Cl_2	r.t.	3	n.r.	n.a.	0%
27[b]	$BF_3 \cdot Et_2O$ (10)	CH_2Cl_2	r.t.	3	n.r.	n.a.	0%
28[b]	TFA (100)	CH_2Cl_2	r.t.	3	Decomp.	n.a.	<5%

All reactions were carried out on a 0.1 mmol scale using 1.5 equiv. of silyl nitronate, except where otherwise stated. NMR spectroscopic yields are calculated using 1 equiv. of 1,3,5- trimethoxybenzene. [a]Reaction carried out under air. [b]2 equiv. of silyl nitronate used. n.r.: no reaction. n.a.: not applicable. "Conversion" refers to how much nitrone starting material has been consumed over the course of the reaction, and it has been calculated based on the remaining nitrone in the crude reaction mixture.

5. Synthesis of products (GP4):

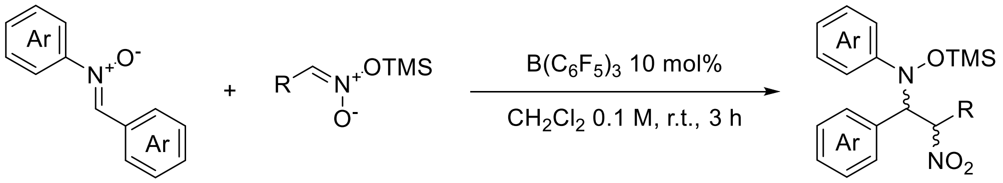

General procedure 4 (GP4): Inside a nitrogen-filled glovebox, nitrone (**1**) (1 equiv.), the $B(C_6F_5)_3$ (0.1 equiv.) and a teflon-coated magnetic stirring bar were added to a microwave vial, which was subsequently closed with a crimp cap. Silyl-nitronate (**2**) (2 equiv.) was added to another microwave vial, which was also closed with a crimp cap. The two vials were taken out from the glovebox, and 0.5 mL of solvent was added to each vessel (1 mL in total). Subsequently, the solution of silyl-nitronate (**2**) was added dropwise at room temperature to the solution of $B(C_6F_5)_3$ and nitrone (**1**) under vigorous stirring (500–1000 rpm) and left to react for the set amount of time. After completion, the crimp cap was removed and the crude reaction mixture was transferred to a 10 mL round bottom flask in order to remove all the volatiles with rotary evaporation. To the crude product, 1 equiv. of 1,3,5-trimethoxybenzene (TMB) and 0.5 mL of $CDCl_3$ were added to calculate the NMR spectroscopic yield. After the NMR spectroscopic measurement, the crude reaction solution was dried under vacuum and purified either with preparative TLC or column chromatography to afford the desired product.

5.1. Structural determination of the minor diastereoisomer:

Following **GP4**, compounds **3a** and **3a’** were separated *via* preparative TLC using a mixture of cyclohexane:ethyl acetate 9:1. After solvent removal, compound **3a** appears as a yellow oil whereas **3a’** appears as a white solid. This observation can be generalised to most of the products obtained in this study. Redissolving compound **3a’** in dichloromethane affords crystals suitable for single crystal X-Ray diffraction after slow evaporation, (see Section 9 for full crystallographic data) which showed a (±)-*(S,R)* configuration. This, by extension, led to the implicit assignment of the major diastereoisomer as (±)-*(R,R)*. The two diastereoisomers had diagnostic signals in the ^{1}H-NMR which allowed us to differentiate the major and minor diastereoisomer throughout this study (Spectra S1). In general, all the major diastereoisomers have the benzylic proton more deshielded than the one of the minor diastereoisomer (cf. doublets at 4.70 ppm and ~4.40 ppm). Moreover, the diastereotopic CH_2 protons of the minor diastereoisomer always appear as two separate spin systems in the range between 2.00 and 3.00 ppm, whereas for the major they always collapse around 1.55 ppm.

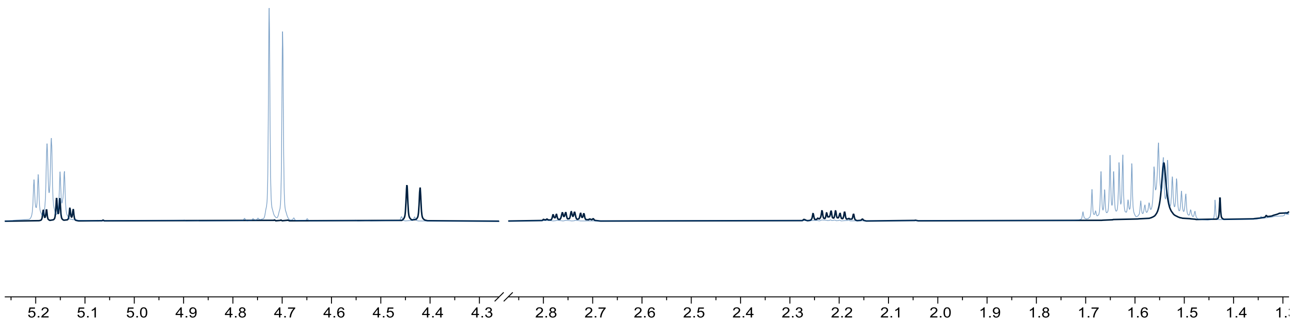

Spectra S1. Stacked ^{1}H NMR spectra of compound **3a** and **3a’**. **3a** is shown in light blue whereas **3a’** is shown in dark blue.

Synthesis of (±)-N-((1R,2R)-2-nitro-1-phenylbutyl)-N-phenyl-O-(trimethylsilyl)hydroxylamine **3a**.

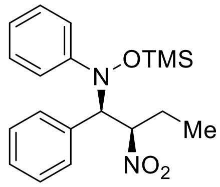

Synthesised according to **GP4** using nitrone **1aa** and nitronate **2a** in dichloromethane for 3 hours. Purification of the crude reaction mixture through preparative TLC with a 9:1 mixture of cyclohexane:ethyl acetate afforded **3a** as a yellow oil (25 mg, 70%). R_f 0.74 (Cy:AcOEt 8:2).

^{1}H-NMR (400 MHz, $CDCl_3$, 298 K) δ: 7.28 (m, 1H, Ar–C**H**), 7.21 (m, 2H, Ar–C**H**), 7.16–7.11 (m, 2H, Ar–C**H**), 7.00 (m, 1H, Ar–C**H**), 6.91 (d, *J*=7.0, 2H, Ar–C**H**), 6.89–6.86 (m, 2H, Ar–C**H**), 5.17 (td, *J*=10.6, 3.5, 1H, C**H**–NO_2), 4.71 (d, *J*=10.9, 1H, C**H**–NOAr), 1.71–1.61 (m, 1H, C**H**$_2$), 1.56–1.50 (m, 1H, C**H**$_2$), 0.89 (t, *J*=7.4, 3H, C**H**$_3$), 0.08 (s, 9H, TMS–C**H**$_3$).

^{13}C-NMR (101 MHz, $CDCl_3$, 298 K) δ: 152.1 (Ar–**C**), 131.9 (Ar–**C**), 130.8 (Ar–**C**), 128.5 (Ar–**C**), 128.2 (Ar–**C**), 127.8 (Ar–**C**), 124.6 (Ar–**C**), 121.1 (Ar–**C**), 91.0 (**C**–NO_2), 76.9 (**C**–NOAr), 25.5 (**C**H_2), 10.1 (**C**H_3), -0.2 (TMS–**C**H_3).

IR ν_{max} (cm^{-1}): 3065, 3032, 2965, 2899, 2160, 1975, 1595, 1580, 1553 (asym. NO_2), 1485, 1452, 1373, 1341, 1323, 1312, 1252 (sym. NO_2), 1206, 1084, 1015.

HRMS (ES+): $[M+H]^+$ calculated for $[C_{19}H_{27}N_2O_3Si]^+$: 359.1791, found 359.1785.

Synthesis of (±)-N-((1S,2R)-2-nitro-1-phenylbutyl)-N-phenyl-O-(trimethylsilyl)hydroxylamine **3a'**.

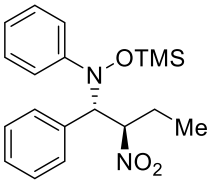

Synthesised according to **GP4** using nitrone **1aa** and nitronate **2a** in dichloromethane for 3 hours. Purification of the crude reaction mixture through preparative TLC with a 9:1 mixture of cyclohexane:ethyl acetate afforded **3a'** as a white solid (4 mg, 11%). R_f 0.56 (Cy:AcOEt 8:2).

^{1}H-NMR (400 MHz, $CDCl_3$, 298 K) δ: 7.22 (m, 1H, Ar–C**H**), 7.19–7.12 (m, 4H, Ar–C**H**), 7.00 (m, 1H, Ar–C**H**), 6.96 (dd, *J*=7.0, 1.6, 2H, Ar–C**H**), 6.84 (dd, *J*=8.6, 1.2, 2H, Ar–C**H**), 5.15 (td, *J*=10.9, 2.7, 1H, C**H**–NO_2), 4.43 (d, *J*=10.7, 1H, C**H**–NOAr), 2.75 (m, 1H, C**H**$_2$), 2.21 (m, 1H, C**H**$_2$), 1.11 (t, *J*=7.4, 3H, C**H**$_3$), 0.07 (s, 9H, TMS–C**H**$_3$).

^{13}C-NMR (101 MHz, $CDCl_3$, 298 K) δ: 152.2 (Ar–**C**), 131.6 (Ar–**C**), 130.4 (Ar–**C**) 128.5 (Ar–**C**), 128.4 (Ar–**C**), 127.4 (Ar–**C**), 124.4 (Ar–**C**), 120.4 (Ar–**C**), 90.9 (**C**–NO_2), 76.6 (**C**–NOAr), 26.7 (**C**H_2), 10.4 (**C**H_3), 1.2 (TMS–**C**H_3).

IR ν_{max} (cm^{-1}): 2963, 2930, 2207, 2156, 2041, 2031, 1952, 1593, 1543 (asym. NO_2), 1485, 1454, 1377, 1302, 1258 (sym. NO_2), 1202, 1076, 1017.

HRMS (ES+): $[M+H]^+$ calculated for $[C_{19}H_{27}N_2O_3Si]^+$: 359.1791, found 359.1778.

Synthesis of (±)-N-(4-methoxyphenyl)-N-((1R,2R)-2-nitro-1-phenylbutyl)-O-(trimethylsilyl)hydroxyl amine **3b**.

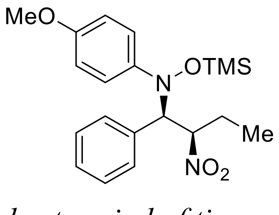

Synthesised according to **GP4** using nitrone **1ab** and nitronate **2a** in dichloromethane for 3 hours. Purification of the crude reaction mixture through preparative TLC with a 9:1 mixture of cyclohexane:ethyl acetate afforded **3b** as a yellow oil (11 mg, 28%). R_f 0.72 (Cy:AcOEt 9:1). *This compound is not stable under ambient conditions, and it decomposes over a short period of time.*

^{1}H-NMR (400 MHz, $CDCl_3$, 298 K) δ: 7.30–7.28 (m, 1H, Ar–C**H**), 7.23–7.19 (m, 2H, Ar–C**H**), 6.91 (m, 2H, Ar–C**H**), 6.80–6.76 (m, 2H, Ar–C**H**), 6.67–6.63 (m, 2H, Ar–C**H**), 5.08 (td, *J*=10.6, 3.4, 1H, C**H**–NO_2), 4.60 (d, *J*=10.8, 1H, C**H**–NOAr), 3.74 (s, 3H, OC**H**$_3$), 1.61 (m, 1H, C**H**$_2$), 1.48 (m, 1H, C**H**$_2$), 0.86 (t, *J*=7.4, 3H, C**H**$_3$), 0.04 (s, 9H, TMS–C**H**$_3$).

^{13}C-NMR (101 MHz, $CDCl_3$, 298 K) δ: 156.9 (Ar–**C**), 144.9 (Ar–**C**), 132.4 (Ar–**C**), 130.9 (Ar–**C**), 128.4 (Ar–**C**), 127.8 (Ar–**C**), 123.3 (Ar–**C**), 113.3 (Ar–**C**), 91.2 (**C**–NO_2), 76.9 (**C**–NOAr), 55.4 (O**C**H_3), 25.5 (**C**H_2), 10.2 (**C**H_3), -0.2 (TMS–**C**H_3).

IR ν_{max} (cm^{-1}): 3032, 2957, 2836, 2033, 2024, 1607, 1587, 1553 (asym. NO_2), 1503, 1456, 1441, 1414, 1373, 1341, 1323, 1298, 1246 (sym. NO_2), 1206, 1180, 1163, 1105, 1084, 1034.

HRMS (ES+): $[M+H]^+$ calculated for $[C_{20}H_{29}N_2O_4Si]^+$: 389.1897, found 389.1893.

Synthesis of (±)-N-((1R,2R)-2-nitro-1-phenylbutyl)-N-(4-(trifluoromethyl)phenyl)-O-(trimethylsilyl) hydroxylamine **3c**.

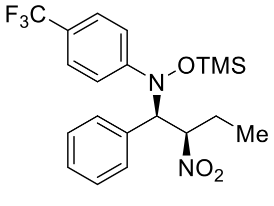

Synthesised according to **GP4** using nitrone **1ac** and nitronate **2a** in dichloromethane for 24 hours. Purification of the crude reaction mixture through preparative TLC with a 9:1 mixture of cyclohexane:ethyl acetate afforded **3ac** as a yellow oil (13 mg, 31%). R_f 0.65 (Cy:AcOEt 9:1).

^{1}H-NMR (400 MHz, $CDCl_3$, 298 K) δ: 7.41 (d, *J*=8.2, 2H, Ar–C**H**), 7.30 (m, 1H, Ar–C**H**), 7.22 (m, 2H, Ar–C**H**), 6.99 (d, *J*=8.1, 2H, Ar–C**H**), 6.91 (d, *J*=7.0, 2H, Ar–C**H**), 5.19 (m, 1H, C**H**–NO_2), 4.77 (d, *J*=11.0, 1H, C**H**–NOAr), 1.70–1.52 (m, 2H, C**H**$_2$), 0.90 (t, *J*=7.4, 3H, C**H**$_3$), 0.10 (s, 9H, TMS–C**H**$_3$).

^{13}C-NMR (101 MHz, $CDCl_3$, 298 K) δ: 155.4 (Ar–**C**), 131.2 (Ar–**C**), 130.6 (Ar–**C**), 128.9 (Ar–**C**), 128.0 (Ar–**C**), 125.6 (m, **C**F_3), 120.4 (Ar–**C**), 90.7 (**C**–NO_2), 76.8 (**C**–NOAr), 25.5 (**C**H_2), 10.1 (**C**H_3), -0.2 (TMS–**C**H_3).

^{19}F-NMR (376 MHz, $CDCl_3$, 298K) δ: -61.93.

IR ν$_{max}$ (cm^{-1}): 3032, 2974, 2940, 2328, 2205, 2182, 2031, 2014, 1614, 1555 (asym. NO_2), 1508, 1454, 1414, 1375, 1323 (sym. NO_2), 254, 1218, 1165, 1121, 1109, 1067, 1011.

HRMS (ES+): $[M+H]^+$ calculated for $[C_{20}H_{26}N_2O_3F_3Si]^+$: 427.1665, found 427.1660.

Synthesis of (±)-N-(4-iodophenyl)-N-((1R,2R)-2-nitro-1-phenylbutyl)-O-(trimethylsilyl)hydroxylamine **3d**.

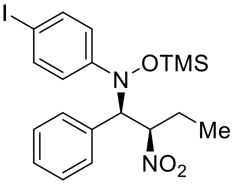

Synthesised according to **GP4** using nitrone **1ad** and nitronate **2a** in dichloromethane for 3 hours. Purification of the crude reaction mixture through preparative TLC with a 95:5 mixture of cyclohexane:ethyl acetate afforded **3d** as a yellow oil (32 mg, 66%). R_f 0.76 (Cy:AcOEt 9:1).

^{1}H-NMR (400 MHz, $CDCl_3$, 298 K) δ: 7.44 (m, 2H, Ar–C**H**), 7.29 (m, 1H, Ar–C**H**), 7.23 (m, 2H, Ar–C**H**), 6.91 (d, *J*=6.9, 2H, Ar–C**H**), 6.64 (m, 2H, Ar–C**H**), 5.13 (td, *J*=10.5, 3.5, 1H, C**H**–NO_2), 4.66 (d, *J*=10.9, 1H, C**H**–NOAr), 1.63 (m, 1H, C**H**$_2$), 1.52 (m, 1H, C**H**$_2$), 0.88 (t, *J*=7.4, 3H, C**H**$_3$), 0.07 (s, 9H, TMS–C**H**$_3$).

^{13}C-NMR (101 MHz, $CDCl_3$, 298 K) δ: 152.1 (Ar–**C**), 137.3 (Ar–**C**), 131.4 (Ar–**C**), 130.7 (Ar–**C**), 128.7 (Ar–**C**), 127.9 (Ar–**C**), 123.1 (Ar–**C**), 90.8 (**C**–NO_2), 88.4 (Ar–**C**), 76.8, (**C**–NOAr), 25.5 (**C**H_2), 10.1 (**C**H_3), -0.2 (TMS–**C**H_3).

IR ν$_{max}$ (cm^{-1}): 3026, 2986, 2326, 2205, 2166, 2033, 2018, 1983, 1721, 1680, 1553 (asym. NO_2), 1478, 1454, 1391, 1373, 1312, 1252 (sym. NO_2), 1209, 1101, 1003.

HRMS (ES+): $[M+H]^+$ calculated for $[C_{19}H_{26}N_2O_3Si^{127}I]^+$: 485.0757, found 485.0757.

Synthesis of (±)-N-(4-iodophenyl)-N-((1S,2R)-2-nitro-1-phenylbutyl)-O-(trimethylsilyl)hydroxylamine **3d'**.

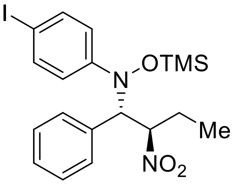

Synthesised according to **GP4** using nitrone **1ad** and nitronate **2a** in dichloromethane for 3 hours. Purification of the crude reaction mixture through preparative TLC with a 95:5 mixture of cyclohexane:ethyl acetate afforded **3d'** as a white solid (6 mg, 12%). R_f 0.70 (Cy:AcOEt 9:1).

^{1}H-NMR (400 MHz, $CDCl_3$, 298 K) δ: 7.46 (m, 2H, Ar–C**H**), 7.23 (m, 1H, Ar–C**H**), 7.16 (m, 2H, Ar–C**H**), 6.98–6.96 (m, 2H, Ar–C**H**), 6.61 (m, 2H, Ar–C**H**), 5.12 (td, *J*=10.9, 2.7, 1H, C**H**–NO_2), 4.38 (d, *J*=10.8, 1H, C**H**–NOAr), 2.69 (m, 1H, C**H**$_2$), 2.19 (m, 1H, C**H**$_2$), 1.10 (t, *J*=7.4, 3H, C**H**$_3$), 0.07 (s, 9H, TMS–C**H**$_3$).

^{13}C-NMR (101 MHz, $CDCl_3$, 298 K) δ: 152.3 (Ar–**C**), 137.5 (Ar–**C**), 131.2 (Ar–**C**), 130.3 (Ar–**C**), 128.8 (Ar–**C**), 127.6 (Ar–**C**), 122.5 (Ar–**C**), 90.7 (**C**–NO_2), 88.0 (Ar–**C**), 76.6 (**C**–NOAr), 26.7 (**C**H_2), 10.4 (**C**H_3), -0.1 (TMS–**C**H_3).

IR ν_{max} (cm^{-1}): 3063, 3030, 2986, 2957, 2851, 2326, 2151, 2016, 1580, 1551 (asym. NO_2), 1495, 1478, 1454, 1391, 1373, 1299, 1252 (sym. NO_2), 1206, 1169, 1101, 1078, 1057, 1003.

HRMS (ES+): $[M+H]^+$ calculated for $[C_{19}H_{26}N_2O_3Si^{127}I]^+$: 485.0757, found 485.0757.

Synthesis of (±)-N-((1R,2R)-1-(4-methoxyphenyl)-2-nitrobutyl)-N-phenyl-O-(trimethylsilyl)hydroxyl amine **3e**.

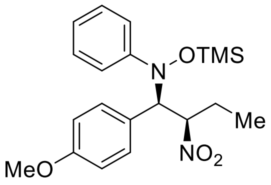

Synthesised according to **GP4** using nitrone **1ae** and nitronate **2a** in dichloromethane for 3 hours. Purification of the crude reaction mixture through preparative TLC with a 9:1 mixture of cyclohexane:ethyl acetate afforded to afford **3e** as a yellow oil (29 mg, 75%). R_f 0.72 (Cy:AcOEt 9:1).

^{1}H-NMR (400 MHz, $CDCl_3$, 298 K) δ: 7.16–7.12 (m, 2H, Ar–C**H**), 6.99 (m, 1H, Ar–C**H**), 6.89–6.87 (m, 2H, Ar–C**H**), 6.83 (d, *J*=8.1, 2H, Ar–C**H**), 6.730 (m, 2H, Ar–C**H**), 5.12 (td, *J*=10.5, 3.6, 1H, C**H**–NO_2), 4.65 (d, *J*=10.9, 1H, C**H**–NOAr), 3.77 (s, 3H, OC**H**3), 1.63 (m, 1H, C**H**2), 1.53 (m, 1H, C**H**2), 0.88 (t, *J*=7.4, 3H, C**H**3), 0.07 (s, 9H, TMS–C**H**3).

^{13}C-NMR (101 MHz, $CDCl_3$, 298 K) δ: 159.6 (Ar–**C**), 152.2 (Ar–**C**), 131.8 (Ar–**C**), 128.2 (Ar–**C**), 124.5 (Ar–**C**), 124.0 (Ar–**C**), 121.1 (Ar–**C**), 113.1 (Ar–**C**), 91.2 (**C**–NO_2), 76.3 (**C**–NOAr), 55.2 (O**C**H3), 25.5 (**C**H2), 10.1 (**C**H3), -0.2 (TMS–**C**H3).

IR ν_{max} (cm^{-1}): 3057, 2957, 2837, 1611, 1595, 1586, 1553 (asym. NO_2), 1512, 1487, 1460, 1443, 1422, 1373, 1341, 1323, 1306, 1285, 1250 (sym. NO_2), 1229, 120, 1180, 1130, 1105, 1078, 1034.

HRMS (ES+): $[M+H]^+$ calculated for $[C_{20}H_{29}N_2O_4Si]^+$: 389.1897, found 389.1885.

Synthesis of (±)-N-((1S,2R)-1-(4-methoxyphenyl)-2-nitrobutyl)-N-phenyl-O-(trimethylsilyl)hydroxyl amine **3e'**.

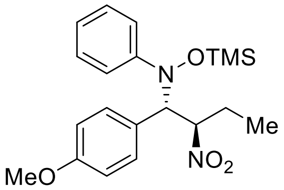

Synthesised according to **GP4** using nitrone **1ae** and nitronate **2a** in dichloromethane for 3 hours. Purification of the crude reaction mixture through preparative TLC with a 9:1 mixture of cyclohexane:ethyl acetate afforded to afford **3e'** as a white solid (2 mg, 5%). R_f 0.66 (Cy:AcOEt 9:1).

^{1}H-NMR (400 MHz, $CDCl_3$, 298 K) δ: 7.16 (m, 2H, Ar–C**H**), 7.0 (m, 1H, Ar–C**H**), 6.89 (m, 2H, Ar–C**H**), 6.85–6.82 (m, 2H, Ar–C**H**), 6.67 (d, *J*=8.9, 2H, Ar–C**H**), 5.10 (td, *J*=10.9, 2.7, 1H, C**H**–NO_2), 4.36 (d, *J*=10.7, 1H, C**H**–NOAr), 3.74 (s, 3H, OC**H**3), 2.74 (m, 1H, C**H**2), 2.20 (m, 1H, C**H**2), 1.10 (t, *J*=7.4, 3H, C**H**3), 0.07 (s, 9H, TMS–C**H**3).

^{13}C-NMR (101 MHz, $CDCl_3$, 298 K) δ: 152.3 (Ar–**C**), 131.6 (Ar–**C**), 128.4 (Ar–**C**), 124.3 (Ar–**C**), 120.5 (Ar–**C**), 112.8 (Ar–**C**), 91.3 (**C**–NO_2), 76.2 (**C**–NOAr), 55.2 (O**C**H3), 26.7 (**C**H2), 10.5 (**C**H3), -0.1 (TMS–**C**H3).

IR ν_{max} (cm^{-1}): 2955, 2924, 2853, 1611, 1593, 1553 (asym. NO_2), 1514, 1487, 1456, 1373, 1252 (asym. NO_2), 1204, 1180, 1034.

HRMS (ES+): $[M+H]^+$ calculated for $[C_{20}H_{29}N_2O_4Si]^+$: 389.1897, found 389.1878.

Synthesis of (±)-N-((1R,2R)-2-nitro-1-(4-(trifluoromethyl)phenyl)butyl)-N-phenyl-O-(trimethylsilyl) hydroxylamine **3f**.

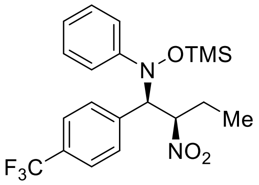

Synthesised according to **GP4** using nitrone **1af** and nitronate **2a** in dichloromethane for 24 hours. Purification of the crude reaction mixture through preparative TLC with a 9:1 mixture of cyclohexane:ethyl acetate afforded to afford **3f** as a yellow oil (7 mg, 16%). R_f 0.76 (Cy:AcOEt 9:1).

^{1}H-NMR (400 MHz, $CDCl_3$, 298 K) δ: 7.47 (d, *J*=8.1, 2H, Ar–C**H**), 7.15 (m, 2H, Ar–C**H**), 7.04–7.00 (m, 3H, Ar–C**H**), 6.85 (dd, *J*=8.6, 1.3, 2H, Ar–C**H**), 5.17 (td, *J*=10.6, 3.4, 1H, C**H**–NO_2), 4.77 (d, *J*=11.0, 1H, C**H**–NOAr), 1.70–1.60 (m, 1H, C**H**$_2$), 1.52–1.45 (m, 1H, C**H**$_2$), 0.90 (t, *J*=7.4, 3H, C**H**$_3$), 0.08 (s, 9H, TMS–C**H**$_3$).

^{13}C-NMR (101 MHz, $CDCl_3$, 298 K) δ: 151.6, (Ar–**C**), 135.6 (Ar–**C**), 131.1 (Ar–**C**), 130.5 (Ar–**C**), 128.5 (Ar–**C**), 124.9 (Ar–**C**), 124.8 (Ar–**C**), 124.7 (q, J_{C-F}=3.6, **C**F_3), 120.8 (Ar–**C**), 90.5 (**C**–NO_2), 76.4 (**C**–NOAr), 25.4 (**C**H_2), 10.1 (**C**H_3), -0.2 (TMS–**C**H_3).

^{19}F-NMR (376 MHz, $CDCl_3$, 298K) δ: -62.64.

IR ν_{max} (cm^{-1}): 2986, 2959, 2855, 2332, 2166, 2160, 2151, 2033, 2012, 2000, 1618, 1595, 1557 (asym. NO_2), 1487, 1420, 1375, 1323 (sym. NO_2), 1254, 1206, 1167, 1126, 1103, 1067, 1018.

HRMS (ES+): $[M+H]^+$ calculated for $[C_{20}H_{26}N_2O_3F_3Si]^+$: 427.1665, found 427.1662.

Synthesis of (±)-N-((1S,2R)-2-nitro-1-(4-(trifluoromethyl)phenyl)butyl)-N-phenyl-O-(trimethylsilyl) hydroxylamine **3f'**.

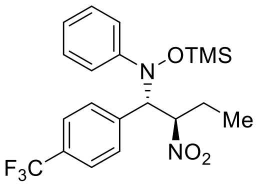

Synthesised according to **GP4** using nitrone **1af** and nitronate **2a** in dichloromethane for 24 hours. Purification of the crude reaction mixture through preparative TLC with a 9:1 mixture of cyclohexane:ethyl acetate afforded to afford **3f'** as a white solid (3 mg, 7%). R_f 0.68 (Cy:AcOEt 9:1).

^{1}H-NMR (400 MHz, $CDCl_3$, 298 K) δ: 7.40 (d, *J*=9.4, 2H, Ar–C**H**), 7.20–7.15 (m, 2H, Ar–C**H**), 7.07–7.01 (m, 3H, Ar–C**H**), 6.83–6.80 (m, 2H, Ar–C**H**), 5.14 (td, *J*=10.9, 2.7, 1H, C**H**–NO_2), 4.47 (d, *J*=10.8, 1H, C**H**–NOAr), 2.78 (m, 1H,, C**H**$_2$), 2.24 (m, C**H**$_2$), 1.13 (t, *J*=7.4, 3H, C**H**$_3$), 0.08 (s, 9H, TMS–C**H**$_3$).

^{13}C-NMR (101 MHz, $CDCl_3$, 298 K) δ: 130.7 (Ar–**C**), 128.6 (Ar–**C**), 124.82 (Ar–**C**), 124.8 (m, **C**F_3), 120.33 (Ar–**C**), 90.64 (**C**–NO_2), 76.23 (**C**–NOAr), 26.67 (**C**H_2), 10.34 (**C**H_3), -0.13 (TMS–**C**H_3). *Due to the small amount of product formed, quaternary carbons could not be detected.*

^{19}F-NMR (376 MHz, $CDCl_3$, 298K) δ: -62.65.

IR ν_{max} (cm^{-1}): 3034, 2959, 2926, 2855, 1595, 1553 (asym. NO_2), 1487,1452, 1422, 1373, 1323 (sym. NO_2), 1256, 1202, 167, 1125, 1099, 1067, 1018.

HRMS (ES+): $[M+H]^+$ calculated for $[C_{20}H_{26}N_2O_3F_3Si]^+$: 427.1665, found 427.1655.

Synthesis of (±)-N-((1R,2R)-1-(4-iodophenyl)-2-nitrobutyl)-N-phenyl-O-(trimethylsilyl)hydroxylamine **3g**.

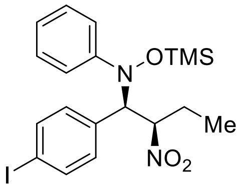

Synthesised according to **GP4** using nitrone **1ag** and nitronate **2a** in dichloromethane for 3 hours. Purification of the crude reaction mixture through preparative TLC with a 9:1 mixture of cyclohexane:ethyl acetate afforded to afford **3g** as a yellow oil (30 mg, 62%). R_f 0.94 (Cy:AcOEt 9:1).

^{1}H-NMR (400 MHz, $CDCl_3$, 298 K) δ: 7.54 (d, *J*=8.5, 2H, Ar–C**H**), 7.15 (td, *J*=7.3, 1.8, 2H, Ar–C**H**), 7.01 (m, 1H, Ar–C**H**), 6.86 (dd, *J*=8.5, 1.2, 2H, Ar–C**H**), 6.64 (d, *J*=7.9, 2H, Ar–C**H**), 5.11 (td, *J*=10.5, 3.4, 1H, C**H**–NO_2), 4.65 (d, *J*=10.9, 1H, C**H**–NOAr), 1.63 (m, 1H, C**H**$_2$), 1.52 (m, 1H, C**H**$_2$), 0.89 (t, *J*=7.4, 3H, C**H**$_3$), 0.07 (s, 9H, TMS–C**H**$_3$). R*f*: 0.94 (Cy:AcOEt 9:1).

^{13}C-NMR (101 MHz, $CDCl_3$, 298 K) δ: 151.7 (Ar–**C**), 136.9 (Ar–**C**), 132.5 (Ar–**C**), 131.3 (Ar–**C**), 128.4 (Ar–**C**), 124.7 (Ar–**C**), 120.9 (Ar–**C**), 94.7 (Ar–**C**), 90.6 (**C**–NO_2), 76.4 (**C**–NOAr), 25.4 (**C**H_2), 10.1 (**C**H_3), -0.2 (TMS–**C**H_3).

IR ν_{max} (cm^{-1}): 3063, 2959, 2035, 1593, 1587, 1553 (asym. NO_2), 1485,1452, 1402, 1373, 1308, 1252 (sym. NO_2), 1206, 1099, 1080, 1063, 1007.

HRMS (ES+): $[M+H]^+$ calculated for $[C_{19}H_{26}N_2O_3Si^{127}I]^+$: 485.0757, found 485.0756.

Synthesis of (±)-N-((1R,2R)-1-(4-iodophenyl)-2-nitrobutyl)-N-phenyl-O-(trimethylsilyl)hydroxylamine **3g'**.

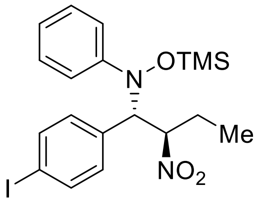

Synthesised according to **GP4** using nitrone **1ag** and nitronate **2a** in dichloromethane for 3 hours. Purification of the crude reaction mixture through preparative TLC with a 9:1 mixture of cyclohexane:ethyl acetate afforded to afford **8a** as a white solid (4 mg, 8%). R_f 0.86 (Cy:AcOEt 9:1).

^{1}H-NMR (400 MHz, $CDCl_3$, 298 K) δ: 7.48 (d, *J*=8.3, 2H, Ar–C**H**), 7.18 (dd, *J*=8.4, 7.3, 2H, Ar–C**H**), 7.02 (m, 1H, Ar–C**H**), 6.83 (dd, *J*=8.6, 1.2, 2H, Ar–C**H**), 6.68 (d, *J*=8.3, 2H, Ar–C**H**), 5.07 (td, *J*=10.9, 2.7, 1H, C**H**–NO_2), 4.35 (d, *J*=10.7, 1H, C**H**–NOAr), 2.75 (m, 1H, C**H**$_2$), 2.27–2.15 (m, 1H, C**H**$_2$), 1.11 (t, *J*=7.4, 3H, C**H**$_3$), 0.07 (s, 9H, TMS–C**H**$_3$).

^{13}C-NMR (101 MHz, $CDCl_3$, 298 K) δ: 151.9 (Ar–**C**), 136.6 (Ar–**C**), 132.1 (Ar–**C**), 131.1 (Ar–**C**), 128.6 (Ar–**C**), 124.7 (Ar–**C**), 120.4 (Ar–**C**), 94.9 (Ar–**C**), 90.7 (**C**–NO_2), 76.3 (**C**–NOAr), 26.6 (**C**H_2), 10.3 (**C**H_3), -0.1 (TMS–**C**H_3).

IR ν_{max} (cm^{-1}): 2957, 2153, 2023, 1998, 1587, 1551 (asym. NO_2), 1508, 1485, 1458, 1406, 1375, 1333, 1298, 1252 (sym. NO_2), 1202, 1072, 1024, 1007.

HRMS (ES+): $[M+H]^+$ calculated for $[C_{19}H_{26}N_2O_3Si^{127}I]^+$: 485.0757, found 485.0752.

Synthesis of (±)-N-((1R,2R)-1-(naphthalen-2-yl)-2-nitrobutyl)-N-phenyl-O-(trimethylsilyl)hydroxyl amine **3h**.

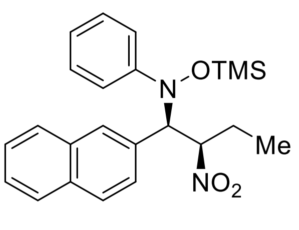

Synthesised according to **GP4** using nitrone **1ah** and nitronate **2a** in dichloromethane for 3 hours. Purification of the crude reaction mixture through preparative TLC with a 95:5 mixture of cyclohexane:ethyl acetate afforded **3h** as a yellow oil (28 mg, 69%). R_f 0.68 (Cy:AcOEt 9:1).

^{1}H-NMR (400 MHz, CDCl$_3$, 298 K) δ: 7.81 (d, *J*=6.7, 1H, Ar–C**H**), 7.71 (t, *J*=7.4, 2H, Ar–C**H**), 7.51–7.43 (m, 2H, Ar–C**H**), 7.32 (s, 1H, Ar–C**H**), 7.15–7.10 (m, 3H, Ar–C**H**), 7.02–6.98 (m, 1H, Ar–C**H**), 6.90 (d, *J*=8.5, 2H, Ar–C**H**), 5.29 (td, *J*=10.6, 3.4, 1H, C**H**–NO$_2$), 4.89 (d, *J*=10.9, 1H, C**H**–NOAr), 1.67 (m, 1H, C**H**$_2$), 1.53 (m, 1H, C**H**$_2$), 0.89 (t, *J*=7.4, 3H, C**H**$_3$), 0.11 (s, 9H, TMS–C**H**$_3$).

^{13}C-NMR (101 MHz, CDCl$_3$, 298 K) δ: 152.0 (Ar–**C**), 133.2 (Ar–**C**), 132.8 (Ar–**C**), 130.5 (Ar–**C**), 129.6 (Ar–**C**), 128.3 (Ar–**C**), 128.2 (Ar–**C**), 128.0 (Ar–**C**), 127.7 (Ar–**C**), 127.2 (Ar–**C**), 126.5 (Ar–**C**), 126.2 (Ar–**C**), 124.7 (Ar–**C**), 121.2 (Ar–**C**), 91.1 (**C**–NO$_2$), 77.0 (**C**–NOAr), 25.6 (**C**H$_2$), 10.1 (**C**H$_3$), -0.2 (TMS–**C**H$_3$).

IR ν$_{max}$ (cm^{-1}): 3059, 2961, 2035, 2023, 2008, 1998, 1595, 1553 (asym. NO$_2$), 1508, 1485, 1373, 1341, 1252 (sym. NO$_2$), 1206, 1080.

HRMS (ES+): [M+H]$^+$ calculated for [C$_{23}$H$_{29}$N$_2$O$_3$Si]$^+$: 409.1947, found 409.1947.

Synthesis of (±)-N-((1S,2R)-1-(naphthalen-2-yl)-2-nitrobutyl)-N-phenyl-O-(trimethylsilyl)hydroxylamine **3h'**.

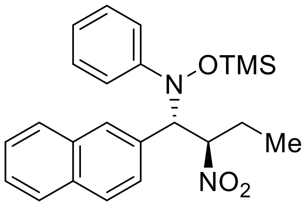

Synthesised according to **GP4** using nitrone **1ah** and nitronate **2a** in dichloromethane for 3 hours. Purification of the crude reaction mixture through preparative TLC with a 95:5 mixture of cyclohexane:ethyl acetate afforded **3h'** as a white solid (3 mg, 7%). R$_f$ 0.62 (Cy:AcOEt 9:1).

^{1}H-NMR (400 MHz, CDCl$_3$, 298 K) δ: 7.77–7.74 (m, 1H, Ar–C**H**), 7.68–7.66 (m, 1H, Ar–C**H**), 7.64–7.62 (m, 1H, Ar–C**H**), 7.44–7.39 (m, 3H, Ar–C**H**), 7.16–7.12 (m, 3H, Ar–C**H**), 7.00 (m, 1H, Ar–C**H**), 6.86–6.84 (m, 2H, Ar–C**H**), 5.28 (td, *J*=10.8, 2.7, 1H, C**H**–NO$_2$), 4.61 (d, *J*=10.6, 1H, C**H**–NOAr), 2.81 (m, 1H, C**H**$_2$), 2.26 (m, 1H, C**H**$_2$), 1.14 (t, *J*=7.4, 3H, C**H**$_3$), 0.08 (s, 9H, TMS–C**H**$_3$).

^{13}C-NMR (101 MHz, CDCl$_3$, 298 K) δ: 152.2 (Ar–**C**), 133.4 (Ar–**C**), 132.6 (Ar–**C**), 130.0 (Ar–**C**), 129.4 (Ar–**C**), 128.5 (Ar–**C**), 128.4 (Ar–**C**), 128.3 (Ar–**C**), 128.0 (Ar–**C**), 127.6 (Ar–**C**), 126.8 (Ar–**C**), 126.3 (Ar–**C**), 125.9 (Ar–**C**), 124.5 (Ar–**C**), 120.5 (Ar–**C**), 91.0 (**C**–NO$_2$), 76.7 (**C**–NOAr), 26.8 (**C**H$_2$), 10.4 (**C**H$_3$), 1.2 (TMS–**C**H$_3$).

IR ν$_{max}$ (cm^{-1}): 3061, 2924, 2351, 2195, 2135, 2008, 1998, 1975, 1593, 1553 (asym. NO$_2$), 1506, 1485, 1373, 1254 (sym. NO$_2$), 1092, 1022.

HRMS (EC): [M]$^+$ calculated for [C$_{23}$H$_{28}$N$_2$O$_3$^{28}Si]$^+$: 408.18637, found 408.1863.

Synthesis of (±)-N-(naphthalen-1-yl)-N-((1R,2R)-1-(naphthalen-2-yl)-2-nitrobutyl)-O-(trimethylsilyl) hydroxylamine **3i**.

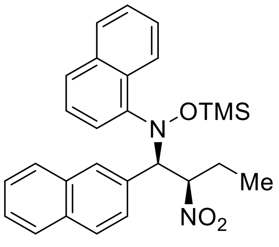

Synthesised according to **GP4** using nitrone **1ai** and nitronate **2a** in dichloromethane for 3 hours. Purification of the crude reaction mixture through preparative TLC with a 95:5 mixture of cyclohexane:ethyl acetate afforded to afford **3ia** as a yellow oil (33 mg, 72%). R$_f$ 0.93 (Cy:AcOEt 9:1).

^{1}H-NMR (400 MHz, CDCl$_3$, 298 K) δ: 8.27 (d, *J*=9.7, 1H, Ar–C**H**), 7.83–7.28 (m, 11H, Ar–C**H**), 6.89 (t, *J*=7.9, 1H, Ar–C**H**), 6.60 (dd, *J*=7.6, 1.2, 1H, Ar–C**H**), 5.59 (td, *J*=10.7, 3.3, 1H, C**H**–NO$_2$), 4.81 (d, *J*=10.6, 1H, C**H**–NOAr), 1.72 (m, 1H, C**H**$_2$), 1.54–1.49 (m, 1H, C**H**$_2$), 0.91 (t, *J*=7.4, 3H, C**H**$_3$), 0.08 (s, 9H, TMS–C**H**$_3$).

^{13}C-NMR (101 MHz, CDCl$_3$, 298 K) δ: 146.7 (Ar–**C**), 134.0 (Ar–**C**), 133.2 (Ar–**C**), 132.7 (Ar–**C**), 130.1 (Ar–**C**), 128.2 (Ar–**C**), 128.2 (Ar–**C**), 127.6 (Ar–**C**), 127.3 (Ar–**C**), 126.5 (Ar–**C**), 126.3 (Ar–

C), 126.1 (Ar–C), 126.0(Ar–C), 125.7 (Ar–C), 124.7 (Ar–C), 123.0 (Ar–C), 120.4 (Ar–C), 90.6 (C–NO_2), 74.2 (C–NOAr), 26.0 (CH_2), 10.2 (CH_3), 0.1 (TMS–CH_3).

IR ν_{max} (cm^{-1}): 3053, 2957, 2195, 1973, 1593, 1553 (asym. NO_2), 1508, 1458, 1439, 1389, 1373, 1341, 1252 (sym. NO_2), 1221, 1132, 1082.

HRMS (ES+): $[M+H]^+$ calculated for $[C_{27}H_{31}N_2O_3Si]^+$: 459.2104, found 459.2101.

Synthesis of (±)-N-((1R,2R)-2-nitro-1-(4-(pyrrolidin-1-yl)phenyl)butyl)-N-phenyl-O-(trimethylsilyl)hydroxylamine **3j**.

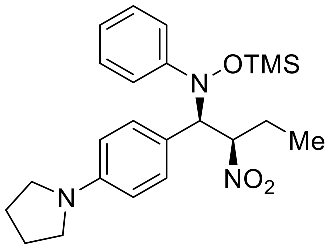

Synthesised according to **GP4** using nitrone **1aj** and nitronate **2a** in dichloromethane for 24 hours. Purification of the crude reaction mixture through preparative TLC with a 9:1 mixture of cyclohexane:ethyl acetate afforded to afford **3j** as a yellow oil (22 mg, 52%). R_f 0.79 (Cy:AcOEt 9:1).

^{1}H-NMR (400 MHz, $CDCl_3$, 298 K) δ: 7.17–7.12 (m, 2H, Ar–CH), 6.99 (m, 1H, Ar–CH), 6.93–6.90 (m, 2H, Ar–CH), 6.74 (d, *J*=8.0, 2H, Ar–CH), 6.38 (d, *J*=8.2, 2H, Ar–CH), 5.11 (td, *J*=10.3, 3.7, 1H, CH–NO_2), 4.60 (d, *J*=10.9, 1H, CH–NOAr), 3.25 (tt, *J*=6.6, 3.0, 4H, Het-CH_2), 1.99 (m, 4H, Het-CH_2), 1.69–1.50 (m, 2H, CH_2), 0.87 (t, *J*=7.4, 3H, CH_3), 0.07 (s, 9H, TMS–CH_3). R_f: 0.80 (Cy:AcOEt 9:1).

^{13}C-NMR (101 MHz, $CDCl_3$, 298 K) δ: 152.5 (Ar–C), 147.6 (Ar–C), 131.7 (Ar–C), 128.1 (Ar–C), 124.2 (Ar–C), 121.2 (Ar–C), 110.7 (Ar–C), 91.6 (C–NO_2), 76.7 (C–NOAr), 47.6 (Het-CH_2), 25.6 (Het-CH_2), 25.6 (CH_2), 10.2 (CH_3), -0.1 (TMS–CH_3).

IR ν_{max} (cm^{-1}): 3038, 2967, 2835, 2160, 2153, 2008, 1975, 1877, 1613, 1595, 1553 (asym. NO_2), 1522, 1487, 1460, 1373, 1341, 1294, 1250 (sym. NO_2), 1207, 1188, 1159, 1078.

HRMS (ES+): $[M+H]^+$ calculated for $[C_{23}H_{34}N_3O_3Si]^+$: 428.2369, found 428.2369.

Synthesis of (±)-N-((1R,2R)-1-(1-methyl-1H-indol-5-yl)-2-nitrobutyl)-N-phenyl-O-(trimethylsilyl) hydroxylamine **3k**.

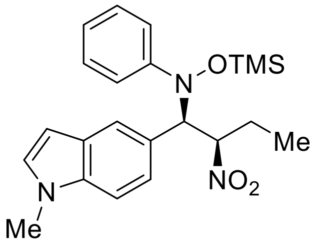

Synthesised according to **GP4** using nitrone **1ak** and nitronate **2a** in dichloromethane for 3 hours. Purification of the crude reaction mixture through preparative TLC with a 9:1 mixture of cyclohexane:ethyl acetate afforded to afford **3k** as a yellow oil (15 mg, 36%). R_f 0.87 (Cy:AcOEt 9:1).

^{1}H-NMR (400 MHz, $CDCl_3$, 298 K) δ: 7.18–7.14 (m, 2H, Ar–CH), 7.13–7.08 (m, 2H, Ar–CH), 7.02 (d, *J*=3.1, 1H, CH=CH–NMe), 6.97 (m, 1H, Ar–CH), 6.91–6.88 (m, 2H, Ar–CH), 6.79 (d, *J*=8.4, 1H, Ar–CH), 6.40 (dd, *J*=3.1, 0.8, 1H, CH=CH–NMe), 5.22 (td, *J*=10.6, 3.4, 1H, CH–NO_2), 4.82 (d, *J*=10.8, 1H, CH–NOAr), 3.77 (s, 3H, N–CH_3), 1.69–1.59 (m, 1H, CH_2), 1.53–1.47 (m, 1H, CH_2), 0.86 (t, *J*=7.4, 3H, CH_3), 0.09 (s, 9H, TMS–CH_3).

^{13}C-NMR (101 MHz, $CDCl_3$, 298 K) δ: 152.5(Ar–C), 136.6 (Ar–C), 129.2 (Ar–C), 128.1 (Ar–C), 127.9 (Ar–C), 124.3 (Ar–C), 124.3 (Ar–C), 123.5 (Ar–C), 123.0 (Ar–C), 121.4 (Ar–C), 108.4 (CH=CH–NMe), 101.4 (CH=CH–NMe), 91.8 (C–NO_2), 77.4 (C–NOAr), 33.0 (N–CH_3), 25.7 (CH_2), 10.2 (CH_3), -0.1 (TMS–CH_3).

IR ν_{max} (cm^{-1}): 2957, 1595, 1551 (asym. NO_2), 1514, 1487, 1451, 1424, 1373, 1341, 1302, 1250 (sym. NO_2), 1206, 1153, 1080.

HRMS (ES+): $[M+H]^+$ calculated for $[C_{22}H_{30}N_3O_3Si]^+$: 412.2056, found 412.2059.

Synthesis of (±)-N-((1R,2R)-1-(benzofuran-5-yl)-2-nitrobutyl)-N-phenyl-O-(trimethylsilyl) hydroxylamine **3l**.

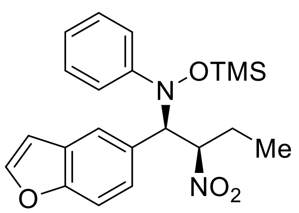

Synthesised according to **GP4** using nitrone **1al** and nitronate **2a** in dichloromethane for 3 hours. Purification of the crude reaction mixture through preparative TLC with a 9:1 mixture of cyclohexane:ethyl acetate afforded **3l** as a yellow oil (28 mg, 70%). R_f 0.73 (Cy:AcOEt 9:1).

^{1}H-NMR (400 MHz, $CDCl_3$, 298 K) δ: 7.59 (d, *J*=2.2, 1H, CH=C**H**–O), 7.34 (d, *J*=8.5, 1H, Ar–C**H**), 7.16 (s, 1H, Ar–C**H**), 7.14–7.09 (m, 2H, Ar–C**H**), 6.98 (m, 1H, Ar–C**H**), 6.87 (dd, *J*=8.5, 1.3, 3H, Ar–C**H**), 6.69 (dd, *J*=2.2, 1.0, 1H, C**H**=CH–O), 5.20 (td, *J*=10.6, 3.4, 1H, C**H**–NO_2), 4.82 (d, *J*=10.9, 1H, C**H**–NOAr), 1.72–1.58 (m, 1H, C**H**$_2$), 1.49 (m, 1H, C**H**$_2$), 0.88 (t, *J*=7.4, 3H, C**H**$_3$), 0.09 (s, 9H, TMS–C**H**$_3$).

^{13}C-NMR (101 MHz, $CDCl_3$, 298 K) δ: 154.86 (Ar–**C**), 152.1 (Ar–**C**), 145.4 (CH=**C**H–O), 128.2 (Ar–**C**), 127.0 (Ar–**C**), 126.9 (Ar–**C**), 126.6 (Ar–**C**), 124.5 (Ar–**C**), 123.5 (Ar–**C**), 121.2 (Ar–**C**), 110.7 (Ar–**C**), 106.8 (**C**H=CH–O), 91.4 (**C**–NO_2), 76.9 (**C**–NOAr), 25.6 (**C**H$_2$), 10.1(**C**H$_3$), -0.2 (TMS–**C**H$_3$).

IR ν_{max} (cm^{-1}): 2959, 2033, 1975, 1595, 1555 (asym. NO_2), 1487, 1468, 1375, 1331, 1316, 1263, 1252 (sym. NO_2), 1207, 1132, 1082, 1032.

HRMS (ES+): $[M+H]^+$ calculated for $[C_{21}H_{27}N_2O_4Si]^+$: 399.1740, found 399.1742.

Synthesis of (±)-N-((1S,2R)-1-(benzofuran-5-yl)-2-nitrobutyl)-N-phenyl-O-(trimethylsilyl)hydroxylamine **3l'**.

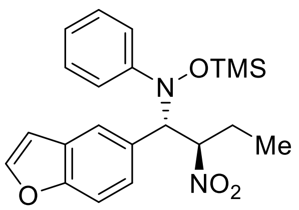

Synthesised according to **GP4** using nitrone **1l** and nitronate **2a** in dichloromethane for 3 hours. Purification of the crude reaction mixture through preparative TLC with a 9:1 mixture of cyclohexane:ethyl acetate afforded to afford **6a** as a white solid (4 mg, 10%). R_f 0.63 (Cy:AcOEt 9:1).

^{1}H-NMR (400 MHz, $CDCl_3$, 298 K) δ: 7.54 (d, *J*=2.2, 1H, CH=C**H**–O), 7.26 (d, *J*=8.5, 2H, Ar–C**H**), 7.23 (d, *J*=1.8, 1H, Ar–C**H**), 7.17–7.12 (m, 2H, Ar–C**H**), 7.00 (m, 1H, Ar–C**H**), 6.84–6.82 (m, 2H, Ar–C**H**), 6.65 (dd, *J*=2.2, 1.0, 1H, C**H**=CH–O), 5.19 (td, *J*=10.8, 2.6, 1H, C**H**–NO_2), 4.54 (d, *J*=10.7, 1H, C**H**–NOAr), 2.78 (m, 1H, C**H**$_2$), 2.25 (m, 1H, C**H**$_2$), 1.12 (t, *J*=7.4, 3H, C**H**$_3$), 0.08 (s, 9H, TMS–C**H**$_3$).

^{13}C-NMR (101 MHz, $CDCl_3$, 298 K) δ: 155.0 (Ar–**C**), 152.2 (Ar–**C**), 145.1(CH=**C**H–O), 128.4 (Ar–**C**), 126.8 (Ar–**C**), 126.7 (Ar–**C**), 126.2 (Ar–**C**), 124.4 (Ar–**C**), 123.2 (Ar–**C**), 120.5 (Ar–**C**), 110.3(Ar–**C**), 106.9 (**C**H=CH–O), 91.4, (**C**–NO_2), 76.7 (**C**–NOAr), 26.8 (**C**H$_2$), 10.5 (**C**H$_3$), -0.1 (TMS–**C**H$_3$).

IR ν_{max} (cm^{-1}): 2957, 2926, 1595, 1551 (asym. NO_2), 1487, 1468, 1373, 1331, 1296, 1252 (sym. NO_2), 1202, 1130, 1078, 1030.

HRMS (ES+): $[M+H]^+$ calculated for $[C_{21}H_{27}N_2O_4Si]^+$: 399.1740, found 399.1742.

Synthesis of (±)-methyl -2-nitro-1-(thiophen-2-yl)butyl)-N-phenyl-O-(trimethylsilyl)hydroxylamine **3m**.

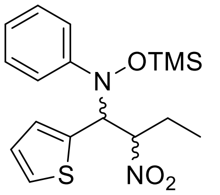

Synthesised according to **GP4** using nitrone **1am** and nitronate **2a** in dichloromethane for 24 hours. Purification of the crude reaction mixture through preparative TLC with a 9:1 mixture of cyclohexane:ethyl acetate afforded **3m** as a pale yellow oil (33 mg, 91%). Obtained as a mixture of diastereoisomers. R_f 0.86 (Cy:AcOEt 9:1).

^{1}H-NMR (400 MHz, $CDCl_3$, 298 K) δ: 7.20 (dd, *J*=4.9, 3.0, 1H, Ar–C**H**), 7.17–7.13 (m, 2H, Ar–C**H**), 7.00 (m, 1H, Ar–C**H**), 6.91–6.88 (m, 2H, Ar–C**H**), 6.84–6.82 (m, 2H, Ar–C**H**), 5.08 (td, *J*=10.5, 3.5, 1H, C**H**–$NO_{2\,major+minor}$), 4.82 (d, *J*=10.8, 1H, C**H**–$NOAr_{major}$), 4.55 (d, *J*=10.6, 1H, C**H**–$NOAr_{minor}$), 2.72 (m, 1H, C$\mathbf{H}_{2minor}$), 2.19 (m, 1H, C$\mathbf{H}_{2minor}$), 1.71–1.59 (m, 1H, C$\mathbf{H}_{2major}$), 1.57–1.47 (m, 1H, C$\mathbf{H}_{2major}$) 1.09 (t, *J*=7.4, 3H, C$\mathbf{H}_{3minor}$), 0.89 (t, *J*=7.4, 3H, C$\mathbf{H}_{3major}$), 0.07 (s, 9H).

^{13}C-NMR (101 MHz, $CDCl_3$, 298 K) δ: 152.2 (Ar–**C**), 132.5 (Ar–**C**), 129.1 (Ar–**C**), 128.8 (Ar–**C**), 128.4(Ar–**C**), 128.3 (Ar–**C**), 126.0 (Ar–**C**), 125.9(Ar–**C**), 124.8 (Ar–**C**), 124.6 (Ar–**C**), 124.0 (Ar–**C**), 120.9 (Ar–$\mathbf{C}_{major}$), 120.3 (Ar–**C**), 91.9(**C**–NO_{2minor}), 91.1 (**C**–NO_{2major}), 72.5 (**C**–$NOAr_{major}$), 72.1 (**C**–$NOAr_{minor}$), 26.4 (**C**H_{2minor}), 25.4 (**C**H_{2major}), 10.4 (**C**H_{3minor}), 10.1 (**C**H_{3major}), -0.2 (TMS–C$\mathbf{H}_{3major+minor}$).

IR $\mathbf{\nu_{max}}$ (cm^{-1}): 2970, 1595, 1553 (asym. NO_2), 1487, 1452, 1373, 1250 (sym. NO_2), 1207, 1080.

HRMS (ES+): $[M+1]^+$ calculated for $[C_{17}H_{25}N_2O_3SiS]^+$: 365.1355, found 365.1359.

Synthesis of (±)-2,2-dimethyl-5-(1-nitropropyl)-1-((trimethylsilyl)oxy)pyrrolidine **3n**.

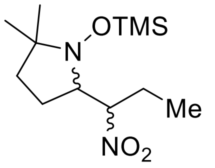

Synthesised according to **GP4** using nitrone **1an** and nitronate **2a** in dichloromethane for 3 hours. Purification of the crude reaction mixture through flash column chromatography with a 9:1 mixture of cyclohexane:ethyl acetate afforded to afford **3n** as a colourless oil (6 mg, 22%). Obtained as a mixture of diastereoisomers. R_f 0.72 (Cy:AcOEt 9:1). [$KMnO_4$ stain]

^{1}H-NMR (400 MHz, $CDCl_3$, 298 K) δ: 4.66 (dt, *J*=10.3, 4.2, 1H, C**H**–NO_{2minor}), 4.49 (m, 1H, C**H**–NO_{2major}), 3.54 (m, 1H, C**H**–$NOTMS_{major}$), 3.20 (m, 1H, C**H**–$NOTMS_{minor}$), 2.13–1.48 (m, 19H, C$\mathbf{H}_{2major+minor}$), 1.12 (s, 3H, C$\mathbf{H}_{3major}$), 1.07 (s, 3H, C$\mathbf{H}_{3minor}$), 1.04 (s, 3H, C$\mathbf{H}_{3major}$), 1.00 (s, 3H, C$\mathbf{H}_{3minor}$), 1.00–0.96 (m, 3H, C$\mathbf{H}_{3major+minor}$), 0.22 (s, 9H, TMS–C$\mathbf{H}_{3minor}$), 0.17 (s, 9H, TMS–C$\mathbf{H}_{3major}$).

^{13}C-NMR (101 MHz, $CDCl_3$, 298 K) δ: δ 91.5 (**C**–NO_{2major}), 90.3(**C**–NO_{2minor}), 68.6 (**C**–$NOTMS_{minor}$), 68.4 (**C**–$NOTMS_{major}$), 65.1 (**C**(Me_2)$NOTMS_{major}$), 64.9 (**C**(Me_2)$NOTMS_{minor}$), 35.1 (**C**H_2), 34.3 (**C**H_2), 27.9 (**C**H_{3major}), 27.7(**C**H_{3minor}), 24.9 (**C**H_2), 20.9 (**C**H_2), 20.4 (**C**H_2), 19.4 (**C**H_2) (**C**H_{3major}), 19.3 (**C**H_2), 18.6 (**C**H_{3minor}), 10.9 (**C**H_{3major}), 10.6 (**C**H_{3minor}), 1.0 (TMS–**C**H_{3minor}), 0.9 (TMS–**C**H_{3major}).

IR $\mathbf{\nu_{max}}$ (cm^{-1}): 2969, 2938, 2882, 2164, 2153, 2000, 1547 (asym. NO_2), 1458, 1364, 1308, 1250 (sym. NO_2), 1173, 1146, 1080.

HRMS (ES+): $[M+H]^+$ calculated for $[C_{12}H_{27}N_2O_3Si]^+$: 275.1791, found 275.1797.

Synthesis of (±)-N-((1R,2R)-2-nitro-1-(o-tolyl)butyl)-N-phenyl-O-(trimethylsilyl)hydroxylamine **3o**.

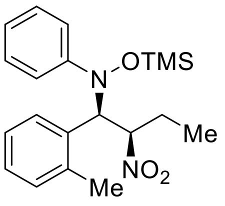

Synthesised according to **GP4** using nitrone **1ao** and nitronate **2a** in dichloromethane for 18 hours. Purification of the crude reaction mixture through preparative TLC with a 95:5 mixture of cyclohexane:ethyl acetate afforded to afford **3o** as a colourless oil (9 mg, 24%). R_f 0.78 (Cy:AcOEt 9:1).

^{1}H-NMR (400 MHz, CDCl$_3$, 298 K) δ: 7.21–7.10 (m, 5H, Ar–C**H**), 7.04–6.99 (m, 2H, Ar–C**H**), 6.93–6.89 (m, 2H, Ar–C**H**), 5.15–5.08 (m, 2H, C**H**–NO$_2$,C**H**–NOAr), 1.72 (s, 3H, Ar-C**H**$_3$), 1.62–1.52 (m, 1H, C**H**$_2$), 1.49–1.41 (m, 1H, C**H**$_2$), 0.85 (t, *J*=7.4, 3H, C**H**$_3$), 0.06 (s, 9H, TMS–C**H**$_3$).

^{13}C-NMR (101 MHz, CDCl$_3$, 298 K) δ: 152.3 (Ar–**C**), 138.5 (Ar–**C**), 130.6 (Ar–**C**), 130.3 (Ar–**C**), 128.5 (Ar–**C**), 128.3 (Ar–**C**), 128.1 (Ar–**C**), 125.2 (Ar–**C**), 125.1 (Ar–**C**), 121.9 (Ar–**C**), 121.0 (Ar–**C**), 92.0 (**C**–NO$_2$), 70.8 (**C**–NOAr), 25.1 (Ar–**C**H$_3$), 19.7 (**C**H$_2$), 10.2 (**C**H$_3$), -0.2 (TMS–**C**H$_3$).

IR ν_{max} (cm^{-1}): 2959, 1595, 1557 (asym. NO$_2$), 1487, 1462, 1373, 1339, 1302, 1252 (sym. NO$_2$), 1207, 1094, 1015.

HRMS (ES+): [M+H]$^+$ calculated for [$C_{20}H_{29}N_2O_3Si$]$^+$: 373.1947, found 373.1948.

Synthesis of (±)-N-((1S,2R)-2-nitro-1-(o-tolyl)butyl)-N-phenyl-O-(trimethylsilyl)hydroxylamine **3o'**.

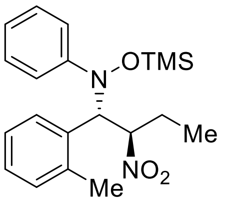

Synthesised according to **GP4** using nitrone **1ao** and nitronate **2a** in dichloromethane for 18 hours. Purification of the crude reaction mixture through preparative TLC with a 9:1 mixture of cyclohexane:ethyl acetate afforded to afford **3o'** as a colourless oil (17 mg, 46%). R_f 0.72 (Cy:AcOEt 9:1).

^{1}H-NMR (400 MHz, CDCl$_3$, 298 K) δ: 7.48–7.45 (m, 1H, Ar–C**H**), 7.19–7.10 (m, 4H, Ar–C**H**), 7.03 (m, 1H, Ar–C**H**), 6.93–6.89 (m, 3H, Ar–C**H**), 5.13 (td, J=10.8, 2.7, 1H, C**H**–NO$_2$), 4.79 (d, *J*=10.6, 1H, C**H**–NOAr), 2.76 (m, 1H, C**H**$_2$), 2.19 (m, 1H, C**H**$_2$), 1.65 (s, 3H, Ar-C**H**$_3$), 1.10 (t, *J*=7.4, 3H, C**H**$_3$), 0.04 (s, 9H, TMS–C**H**$_3$).

^{13}C-NMR (101 MHz, CDCl$_3$, 298 K) δ: 152.6 (Ar–**C**), 138.5 (Ar–**C**), 130.3 (Ar–**C**), 130.1 (Ar–**C**), 129.9 (Ar–**C**), 128.5 (Ar–**C**), 128.3 (Ar–**C**), 124.9 (Ar–**C**), 124.8 (Ar–**C**), 121.0 (Ar–**C**), 91.4 (**C**–NO$_2$), 70.4 (**C**–NOAr), 27.0 (Ar–**C**H$_3$), 19.5 (**C**H$_2$), 10.5 (**C**H$_3$), -0.2 (TMS–**C**H$_3$).

IR ν_{max} (cm^{-1}): 3026, 2961, 1595, 1551 (asym. NO$_2$), 1487, 1458, 1452, 1373, 1341, 1252 (sym. NO$_2$), 1204, 1163, 1076, 1024.

HRMS (ES+): [M+H]$^+$ calculated for [$C_{20}H_{29}N_2O_3Si$]$^+$: 373.1947, found 373.1944.

Synthesis of (±)-N-((1R,2R)-2-nitro-1-phenylbutyl)-N-(o-tolyl)-O-(trimethylsilyl)hydroxylamine **3p**.

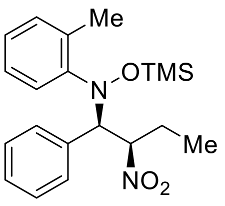

Synthesised according to **GP4** using nitrone **1ap** and nitronate **2a** in dichloromethane for 18 hours. Purification of the crude reaction mixture through preparative TLC with a 95:5 mixture of cyclohexane:ethyl acetate afforded to afford **3p** as a colourless oil (30 mg, 81%). R_f 0.79 (Cy:AcOEt 9:1).

^{1}H-NMR (400 MHz, CDCl$_3$, 298 K) δ: 7.30–7.27 (m, 1H, Ar–C**H**), 7.22–7.21 (m, 2H, Ar–C**H**), 7.07 (d, *J*=7.6, 2H, Ar–C**H**), 6.91 (t, *J*=7.3, 1H, Ar–C**H**), 6.72 (t, *J*=7.7, 1H, Ar–C**H**), 6.42 (d, *J*=8.1, 1H, Ar–C**H**) 5.32 (td, *J*=10.8, 3.2, 1H, C**H**–NO$_2$), 4.30 (d, *J*=10.6, 1H, C**H**–NOAr), 2.28 (s, 3H, Ar-C**H**$_3$), 1.72–1.60 (m, 1H, C**H**$_2$), 1.53–1.42 (m, 1H, C**H**$_2$), 0.88 (t, *J*=7.4, 3H, C**H**$_3$), 0.02 (s, 9H, TMS–C**H**$_3$).

^{13}C-NMR (101 MHz, $CDCl_3$, 298 K) δ: 149.3 (Ar–**C**), 132.4 (Ar–**C**), 131.1 (Ar–**C**), 130.6 (Ar–**C**), 130.3 (Ar–**C**), 128.5 (Ar–**C**), 127.8 (Ar–**C**), 125.4 (Ar–**C**), 125.2 (Ar–**C**), 123.8 (Ar–**C**), 90.6 (**C**–NO_2), 73.7 (**C**–NOAr), 25.7 (Ar–**C**H_3), 17.7 (**C**H_2), 10.2 (**C**H_3), -0.01 (TMS–**C**H_3).

IR ν_{max} (cm^{-1}): 2957, 1555 (asym. NO_2), 1483, 1454, 1373, 1339, 1310, 1250 (sym. NO_2), 1211, 1186, 1109, 1082, 1049, 1034.

HRMS (ES+): $[M+H]^+$ calculated for $[C_{20}H_{29}N_2O_3Si]^+$: 373.1947, found 373.1947.

Synthesis of (±)-N-((1R,2R)-2-nitro-1-phenylheptyl)-N-phenyl-O-(trimethylsilyl)hydroxylamine **3q**.

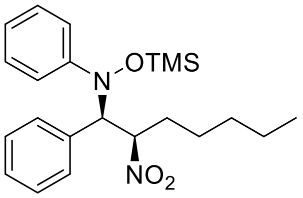

Synthesised according to **GP4** using nitrone **1aa** and nitronate **2b** in dichloromethane for 3 hours. Purification of the crude reaction mixture through preparative TLC with a 95:5 mixture of cyclohexane:ethyl acetate afforded **3q** as a colourless oil (20 mg, 50%). R_f 0.90 (Cy:AcOEt 9:1).

^{1}H-NMR (400 MHz, $CDCl_3$, 298 K) δ: 7.28 (m, 1H, Ar–C**H**), 7.23–7.18 (m, 2H, Ar–C**H**), 7.15–7.11 (m, 2H, Ar–C**H**), 6.99 (m, 1H, Ar–C**H**), 6.90–6.85 (m, 4H, Ar–C**H**), 5.24 (td, J=10.9, 3.0, 1H, C**H**–NO_2), 4.70 (d, J=10.9, 1H, C**H**–NOAr), 1.69–1.60 (m, 1H, C**H**$_2$), 1.43–1.14 (m, 7H, C**H**$_2$), 0.81 (t, J=7.0, 3H, C**H**$_3$), 0.08 (s, 9H, TMS–C**H**$_3$).

^{13}C-NMR (101 MHz, $CDCl_3$, 298 K) δ: 152.1 (Ar–**C**), 131.9 (Ar–**C**), 130.7 (Ar–**C**), 128.5 (Ar–**C**), 128.2 (Ar–**C**), 127.8 (Ar–**C**), 124.5 (Ar–**C**), 121.0 (Ar–**C**), 89.7 (**C**–NO_2), 77.2 (**C**–NOAr), 31.9 (**C**H_2), 30.9 (**C**H_2), 25.3 (**C**H_2), 22.4 (**C**H_2), 14.0 (**C**H_3), -0.2 (TMS–**C**H_3).

IR ν_{max} (cm^{-1}): 2957, 2930, 2860, 1595, 1557 (asym. NO_2), 1485, 1452, 1377, 1252 (sym. NO_2), 1204, 1026.

HRMS (ES+): $[M+1]^+$ calculated for $[C_{22}H_{33}N_2O_3Si]^+$: 401.2260, found 401.2263.

Synthesis of (±)-N-((1S,2R)-2-nitro-1-phenylheptyl)-N-phenyl-O-(trimethylsilyl)hydroxylamine **3q'**.

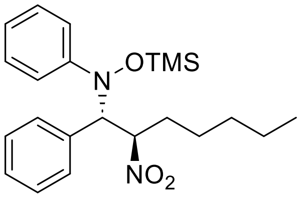

Synthesised according to **GP4** using nitrone **1aa** and nitronate **2b** in dichloromethane for 3 hours. Purification of the crude reaction mixture through preparative TLC with a 95:5 mixture of cyclohexane:ethyl acetate afforded **3q'** as a colourless oil (5 mg, 13%). R_f 0.85 (Cy:AcOEt 9:1).

^{1}H-NMR (400 MHz, $CDCl_3$, 298 K) δ: 7.24–7.20 (m, 1H, Ar–C**H**), 7.19–7.12 (m, 4H, Ar–C**H**), 7.00 (m, 1H, Ar–C**H**), 6.97–6.95 (m, 2H, Ar–C**H**), 6.86–6.83 (m, 2H, Ar–C**H**), 5.21 (td, *J*=10.9, 2.6, 1H, C**H**–NO_2), 4.42 (d, *J*=10.6, 1H, C**H**–NOAr), 2.69–2.62 (m, 1H, C**H**$_2$), 2.24–2.11 (m, 1H, C**H**$_2$), 1.40–1.25 (m, 6H, C**H**$_2$), 0.93 (m, 3H, C**H**$_3$), 0.06 (s, 9H, TMS–C**H**$_3$).

^{13}C-NMR (101 MHz, $CDCl_3$, 298 K) δ: 152.2 (Ar–**C**), 130.4 (Ar–**C**), 128.5 (Ar–**C**), 128.4(Ar–**C**), 127.4 (Ar–**C**), 124.4 (Ar–**C**), 120.5 (Ar–**C**), 89.8 (**C**–NO_2), 77.2 (**C**–NOAr), 33.3 (**C**H_2), 31.6 (**C**H_2), 25.8 (**C**H_2), 22.6 (**C**H_2), 14.1 (**C**H_3), -0.1 (TMS–**C**H_3).

IR ν_{max} (cm^{-1}): 2957, 2928, 2862, 1553 (asym. NO_2), 1485, 1452, 1377, 1252 (sym. NO_2), 1202.

HRMS (ES+): $[M+1]^+$ calculated for $[C_{22}H_{33}N_2O_3Si]^+$: 401.2260, found 401.2265.

Synthesis of (±)-N-(2-nitro-1,2-diphenylethyl)-N-phenyl-O-(trimethylsilyl)hydroxylamine **3r**.

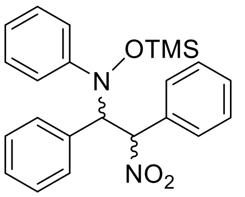

Synthesised according to **GP4** using nitrone **1aa** and nitronate **2c** in dichloromethane for 24 hours. Purification of the crude reaction mixture through preparative TLC with a 95:5 mixture of cyclohexane:ethyl acetate afforded to afford **3r** as a colourless oil (28 mg, 69%). Obtained as a mixture of diastereoisomers. R_f 0.73 (Cy:AcOEt 9:1).

^{1}H-NMR (400 MHz, $CDCl_3$, 298 K) δ: 7.72 (dd, *J*=6.6, 3.0, 1H, Ar–C**H**), 7.41–7.37 (m, 3H, Ar–C**H**), 7.29–7.26 (m, 1H, Ar–C**H**), 7.21–7.16 (m, 5H, Ar–C**H**), 7.09–7.04 (m, 2H, Ar–C**H**), 7.02–6.96 (m, 4H, Ar–C**H**), 6.94–6.89 (m, 1H, Ar–C**H**), 6.84–6.74 (m, 2H, Ar–C**H**), 6.39 (d, *J*=11.2, 1H, C**H**–NO_{2minor}), 6.23 (d, *J*=11.4, 1H, C**H**–NO_{2major}), 5.37 (d, *J*=11.3, 1H, C**H**–$NOAr_{major}$), 5.34 (d, *J*=11.1, 1H, C**H**–$NOAr_{minor}$), 0.14 (s, 9H, TMS–C**H**$_{3major}$), -0.23 (s, 9H TMS–C**H**$_{3minor}$).

^{13}C-NMR (101 MHz, $CDCl_3$, 298 K) δ: 152.1 (Ar–**C**), 151.9 (Ar–**C**), 133.7 (Ar–**C**), 132.4 (Ar–**C**), 131.0 (Ar–**C**), 131.0 (Ar–**C**), 130.1 (Ar–**C**), 130.0 (Ar–**C**), 129.8 (Ar–**C**), 129.44 (Ar–**C**), 128.8 (Ar–**C**), 128.7 (Ar–**C**), 128.3 (Ar–**C**), 128.2 (Ar–**C**), 128.0 (Ar–**C**), 127.9 (Ar–**C**), 127.3 (Ar–**C**), 125.7 (Ar–**C**), 124.7 (Ar–**C**), 124.0(Ar–**C**), 121.1 (Ar–**C**), 120.3 (Ar–**C**), 92.4 (**C**–NO_{2major}), 91.6 (**C**–NO_{2minor}), 76.5 (**C**–$NOAr_{major}$), 74.1 (**C**–$NOAr_{minor}$), -0.2 (TMS–CH_3).

IR ν_{max} (cm^{-1}): 3063, 3034, 2957, 2903, 2166, 2045, 1973, 1802, 1595, 1555 (asym. NO_2), 1485, 1454, 1360, 1296, 1252 (sym. NO_2), 1204, 1076, 1024.

HRMS (ES+): $[M+H]^+$ calculated for $[C_{23}H_{27}N_2O_3Si]^+$: 407.1791, found 407.1794.

Synthesis of (±)- (2S,3R)-2-nitro-3-phenyl-3-(phenyl((trimethylsilyl)oxy)amino)propan-1-ol **3s**.

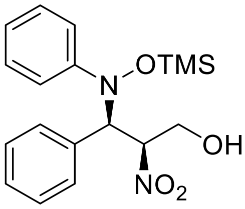

Synthesised according to **GP4** using nitrone **1aa** and nitronate **2d** in dichloromethane for 3 hours. Purification of the crude reaction mixture through preparative TLC with a 8:2 mixture of cyclohexane:ethyl acetate afforded to afford **3s** as a yellow oil (10 mg, 28%). R_f 0.23 (Cy:AcOEt 9:1).

^{1}H-NMR (400 MHz, $CDCl_3$, 298 K) δ: 7.29 (m, 1H, Ar–C**H**), 7.24–7.20 (m, 2H, Ar–C**H**), 7.16–7.11 (m, 2H, Ar–C**H**), 7.02–7.00 (m, 1H, Ar–C**H**), 6.98–6.96 (m, 2H, Ar–C**H**), 6.91–6.88 (m, 2H, Ar–C**H**), 5.38 (m, 1H, C**H**–NO_2), 4.85 (d, *J*=10.9, 1H, C**H**–NOAr), 3.79–3.70 (m, 2H, C**H**$_2$), 1.70 (br s, 1H, O**H**), 0.07 (s, 9H, TMS–C**H**$_3$).

^{13}C-NMR (101 MHz, $CDCl_3$, 298 K) δ: 151.8 (Ar–**C**), 131.4 (Ar–**C**), 130.6 (Ar–**C**), 128.9 (Ar–**C**), 128.3 (Ar–**C**), 128.0 (Ar–**C**), 124.8 (Ar–**C**), 121.3 (Ar–**C**), 90.3 (**C**–NO_2), 74.0 (**C**–NOAr), 62.7 (**C**H_2), -0.2 (TMS–**C**H_3).

IR ν_{max} (cm^{-1}): 3431 (OH), 3063, 3032, 2959, 2033, 2000, 1665, 1595, 1557 (asym. NO_2), 1487, 1452, 1366, 1312, 1252 (sym. NO_2), 1202, 1071.

HRMS (CI): $[M]^+$ calculated for $[C_{18}H_{24}N_2O_4Si]^+$: 360.14999, found 360.1497.

Synthesis of (±)-N-(3-((tert-butyldimethylsilyl)oxy)-2-nitro-1-phenylpropyl)-N-phenyl-O-(trimethylsilyl) hydroxylamine **3t**.

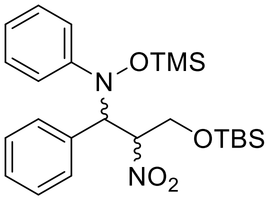

Synthesised according to **GP4** using nitrone **1aa** and nitronate **2e** in dichloromethane for 3 hours. Purification of the crude reaction mixture through preparative TLC with a 95:5 mixture of cyclohexane:ethyl acetate afforded to afford **3t** as a yellow oil (33 mg, 70%). Obtained as a mixture of diastereoisomers. R_f 0.84 (Cy:AcOEt 9:1).

^{1}H-NMR (400 MHz, $CDCl_3$, 298 K) δ: 7.30–7.11 (m, 8H, Ar–C**H**), 7.03–6.97 (m, 3H, Ar–C**H**), 6.95–6.84 (m, 4H, Ar–C**H**), 5.44 (m, 1H, C**H**–NO_{2major}), 5.34 (m, 1H, C**H**–NO_{2minor}), 4.81 (d, *J*=11.0, 1H, C**H**–$NOAr_{minor}$), 4.62–4.55 (m, 2H, C**H**$_{2major}$), 4.38 (dd, *J*=10.9, 8.5, 1H, C**H**–$NOAr_{major}$), 3.72 (dd, *J*=11.3, 7.8, 1H, C**H**$_{2minor}$), 3.64 (dd, *J*=11.3, 3.3, 1H, C**H**$_{2minor}$), 0.91 (s, 9H, TMS–C**H**$_{3major}$), 0.81 (s, 9H TMS–C**H**$_{3minor}$), 0.11 (s, 9H, TBS–C**H**$_{3minor}$), 0.08 (s, 3H, TBS–C**H**$_{3major}$), 0.08 (s, 3H, TBS–C**H**$_{3major}$), 0.07 (s, 9H, TBS–C**H**$_{3major}$), -0.08 (s, 3H, TBS–C**H**$_{3minor}$), -0.14 (s, 3H, TBS–C**H**$_{3minor}$).

^{13}C-NMR (101 MHz, $CDCl_3$, 298 K) δ: 151.9 (Ar–**C**), 151.9 (Ar–**C**), 131.5 (Ar–**C**), 131.5 (Ar–**C**), 130.7 (Ar–**C**), 130.3 (Ar–**C**), 128.7 (Ar–**C**), 128.6 (Ar–**C**), 128.4 (Ar–**C**), 128.2 (Ar–**C**), 127.8 (Ar–**C**), 127.6 (Ar–**C**), 124.6 (Ar–**C**), 124.4 (Ar–**C**), 121.1 (Ar–**C**), 120.2 (Ar–**C**), 90.4 (**C**–NO_{2minor}), 90.2 (**C**–NO_{2major}), 73.9 (**C**–$NOAr_{minor}$), 73.0 (**C**–$NOAr_{major}$), 64.3 (**C**H_{2major}), 63.2 (**C**H_{2minor}), 25.8 (TMS–CH_{3major}), 25.7 (TMS–CH_{3minor}), 18.3 (TBS–**C**$_{major}$), 18.2 (TBS–**C**$_{minor}$), -0.1 (TBS–CH_{3major}), -0.2 (TBS–CH_{3major}), -5.45 (TBS–**C**H_{3major}), -5.48 (TBS–**C**H_{3minor}), -5.6 (TBS–**C**H_{3minor}), -5.8 (TBS–**C**H_{3minor}).

IR ν_{max} (cm^{-1}): 2955, 2930, 2859, 1595, 1557 (asym. NO_2), 1487, 1389, 1362, 1252 (sym. NO_2), 1204, 1119, 1007.

HRMS (ES+): $[M+1]^+$ calculated for $[C_{24}H_{39}N_2O_4Si_2]^+$: 475.2448, found 475.2448.

Synthesis of (±)-N-(2-nitro-1-phenyl-3-((tetrahydro-2H-pyran-2-yl)oxy)propyl)-N-phenyl-O-(trimethylsilyl)hydroxylamine **3u**.

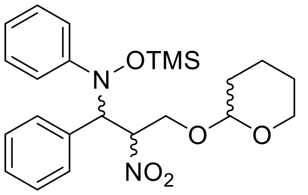

Synthesised according to **GP4** using nitrone **1aa** and nitronate **2f** in dichloromethane for 3 hours. Purification of the crude reaction mixture through preparative TLC with a 95:5 mixture of cyclohexane:ethyl acetate afforded to afford **3u** as yellow oil (30 mg, 68%). Obtained as a mixture of distereoisomers. R_f 0.55 (Cy:AcOEt 9:1).

^{1}H-NMR (400 MHz, $CDCl_3$, 298 K) δ: 7.28 (m, 1H), 7.26–7.10 (m, 10H), 7.03–6.99 (m, 4H), 6.95–6.84 (m, 7H), 5.57–5.46 (m, 3H, C**H**–NO_2), 4.84–4.72 (m, 2H), 4.64–4.50 (m, 3H), 4.43 (dd, *J*=10.9, 3.0, 1H), 4.26 (t, *J*=3.4, 1H), 4.19 (dd, *J*=11.1, 9.1, 1H), 3.91–3.83 (m, 1H), 3.75–3.68 (m, 2H), 3.61–3.53 (m, 2H), 3.44–3.21 (m, 3H), 1.87–1.34 (m, 15H), 0.08 (s, 19H, TMS–C**H**$_3$).

^{13}C-NMR (101 MHz, $CDCl_3$, 298 K) δ: 151.8, 151.7, 131.4, 131.4, 130.7, 130.6, 130.4, 130.4, 128.7 128.7, 128.4, 128.2, 127.8, 127.6, 127.5, 124.7, 124.7, 124.6, 124.5, 121.3, 121.2, 120.5, 120.5, 99.8, 99.6, 98.0, 97.3, 89.0, 88.4, 88.2, 88.1, 74.4, 73.8, 73.5, 68.4, 67.5, 67.3, 65.8, 62.2, 62.2, 61.6, 60.9, 30.4, 30.3, 30.0, 25.5, 25.4, 25.3, 25.2, 19.1, 19.0, 18.6, 18.2, -0.1, -0.12, -0.2.

IR ν_{max} (cm^{-1}): 2949, 1595, 1557 (asym. NO_2), 1485, 1454, 1371, 1252 (sym. NO_2), 1202, 1125, 1069, 1036.

HRMS (ES+): $[M+1]^+$ calculated for $[C_{23}H_{33}N_2O_5Si]^+$: 445.2159, found 445.2158.

Synthesis of (±)-N-((1R,2R)-2-nitro-1-phenylpent-4-en-1-yl)-N-phenyl-O-(trimethylsilyl)hydroxylamine **3v**.

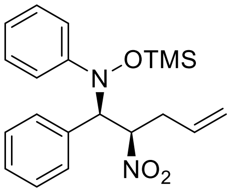

Synthesised according to **GP4** using nitrone **1aa** and nitronate **2g** in dichloromethane for 3 hours. Purification of the crude reaction mixture through preparative TLC with a 95:5 mixture of cyclohexane:ethyl acetate afforded to afford **3v** as a colourless oil (24 mg, 65%). R_f 0.80 (Cy:AcOEt 9:1).

^{1}H-NMR (400 MHz, $CDCl_3$, 298 K) δ: 7.29 (m, 1H, Ar–C**H**), 7.24–7.19 (m, 2H, Ar–C**H**), 7.16–7.11 (m, 2H, Ar–C**H**), 7.00 (m, 1H, Ar–C**H**), 6.92 (d, *J*=6.9, 2H, Ar–C**H**), 6.90–6.86 (m, 2H, Ar–C**H**), 5.65 (m, 1H, C**H**=CH_2), 5.33–5.22 (m, 1H, C**H**–NO_2), 5.07 (dq, *J*=10.2, 1.2, 1H, CH=C**H_2**), 4.97 (dq, *J*=17.0, 1.4, 1H, CH=C**H_2**), 4.72 (d, *J*=10.9, 1H, C**H**–NOAr), 2.36–2.21 (m, 2H, C**H_2**), 0.07 (s, 9H, TMS–C**H_3**).

^{13}C-NMR (101 MHz, $CDCl_3$, 298 K) δ: 152.0 (Ar–**C**), 131.6 (Ar–**C**), 131.1 (Ar–**C**), 130.8 (**C**H=CH_2), 128.6 (Ar–**C**), 128.2 (Ar–**C**), 127.9 (Ar–**C**), 124.6 (Ar–**C**), 121.1 (Ar–**C**), 119.8 (CH=**C**H_2), 89.3 (**C**–NO_2), 76.6 (**C**–NOAr), 36.5 (**C**H_2), -0.2 (TMS–**C**H_3).

IR ν_{max} (cm^{-1}): 2957, 1757, 1645, 1595, 1557 (asym. NO_2), 1485, 1452, 1435, 1371, 1310, 1252 (sym. NO_2), 1204, 1026.

HRMS (ES+): $[M+1]^+$ calculated for $[C_{20}H_{27}N_2O_3Si]^+$: 371.1791, found 371.1793.

Synthesis of (±)-N-((1S,2R)-2-nitro-1-phenylpent-4-en-1-yl)-N-phenyl-O-(trimethylsilyl)hydroxylamine **3v'**.

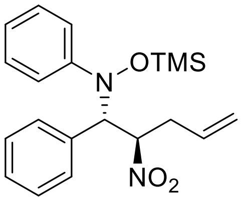

Synthesised according to **GP4** using nitrone **1aa** and nitronate **2g** in dichloromethane for 3 hours. Purification of the crude reaction mixture through preparative TLC with a 95:5 mixture of cyclohexane:ethyl acetate afforded to afford **3v'** as a colourless oil (8 mg, 22%). R_f 0.70 (Cy:AcOEt 9:1).

^{1}H-NMR (400 MHz, $CDCl_3$, 298 K) δ: 7.25–7.18 (m, 2H, Ar–C**H**), 7.18–7.13 (m, 3H, Ar–C**H**), 7.03–6.96 (m, 3H, Ar–C**H**), 6.87–6.84 (m, 2H, Ar–C**H**), 5.87 (m, 1H, C**H**=CH_2), 5.32–5.21 (m, 3H, CH=C**H_2**+C**H**–NO_2), 4.45 (d, *J*=10.7, 1H, C**H**–NOAr), 3.49–3.43 (m, 1H, C**H_2**), 2.96–2.87 (m, 1H, C**H_2**), 0.08 (s, 9H, TMS–C**H_3**).

^{13}C-NMR (101 MHz, $CDCl_3$, 298 K) δ: 152.1 (Ar–**C**), 131.5 (Ar–**C**), 131.3 (Ar–**C**), 130.4 (**C**H=CH_2), 128.6 (Ar–**C**), 128.5 (Ar–**C**), 127.5 (Ar–**C**), 124.5 (Ar–**C**), 120.5 (Ar–**C**), 120.0 (CH=**C**H_2), 88.8 (**C**–NO_2), 76.3 (**C**–NOAr), 37.5 (**C**H_2), 0.0 (TMS–**C**H_3).

IR ν_{max} (cm^{-1}): 2957, 2924, 2853, 1595, 1553 (asym. NO_2), 1485, 1454, 1373, 1252 (sym. NO_2).

HRMS (ES+): $[M+1]^+$ calculated for $[C_{20}H_{27}N_2O_3Si]^+$: 371.1791, found 371.1793.

Synthesis of (±)-2-nitro-1,3-diphenyl-3-(phenyl((trimethylsilyl)oxy)amino)propan-1-one **3w**.

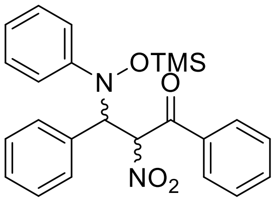

Synthesised according to **GP4** using nitrone **1aa** and nitronate **2h** in dichloromethane for 3 hours. Purification of the crude reaction mixture through flash column chromatography with a 9:1 mixture of cyclohexane:ethyl acetate with 1% Et_3N afforded **3w** as yellow oil (36 mg, 83%). Obtained as a mixture of distereoisomers. R_f 0.76 (Cy:AcOEt 9:1).

This compound was not stable even on deactivated silica, hence we did a flash purification (residence time inside the column: <3 min). During the NMR spectrum acquisition, we could already observe decomposition patterns. We infer this is due to the strong acidity of the proton α to the nitro group.

^{1}H-NMR (400 MHz, CDCl$_3$, 298 K) δ: 8.24–8.22 (m, 1H, Ar–C**H**), 7.89–7.85 (m, 2H, Ar–C**H**), 7.71–7.41 (m, 7H, Ar–C**H**), 7.25–7.05 (m, 7H, Ar–C**H**), 7.04 (d, *J*=4.6, 1H, C**H**–NO$_{2major}$), 7.00 (dd, *J*=8.6, 1.1, 4H, Ar–C**H**), 6.96–6.93 (m, 2H, Ar–C**H**+ C**H**–NO$_{2minor}$), 6.87 (dd, *J*=8.2, 1.3, 2H, Ar–C**H**), 5.56 (d, *J*=10.3, 1H, C**H**–NOAr$_{minor}$), 5.43 (d, *J*=10.7, 1H, C**H**–NOAr$_{major}$), 0.14 (s, 9H, TMS–C**H**$_{3major}$), -0.20 (s, 9H, TMS–C**H**$_{3minor}$).

^{13}C-NMR (101 MHz, CDCl$_3$, 298 K) δ: 187.2 (**C**=O$_{major}$), 185.3 (**C**=O$_{major}$), 151.7 (Ar–**C**), 151.6 (Ar–**C**), 135.3 (Ar–**C**), 134.7 (Ar–**C**), 131.2 (Ar–**C**), 130.7 (Ar–**C**), 130.5 (Ar–**C**), 129.6 (Ar–**C**), 129.3 (Ar–**C**), 129.2 (Ar–**C**), 129.1 (Ar–**C**), 129.0 (Ar–**C**), 128.8 (Ar–**C**), 128.6 (Ar–**C**), 128.4 (Ar–**C**), 128.3 (Ar–**C**), 128.0 (Ar–**C**), 127.7 (Ar–**C**), 125.1 (Ar–**C**), 124.8 (Ar–**C**), 121.5 (Ar–**C**), 121.0 (Ar–**C**), 120.9 (Ar–**C**), 119.6 (Ar–**C**), 88.3 (**C**–NO$_{2major}$), 88.1 (**C**–NO$_{2minor}$), 74.5 (**C**–NOAr$_{major}$), 73.9 (**C**–NOAr$_{minor}$), -0.2 (TMS–**C**H$_{3major}$), -0.4 (TMS–**C**H$_{3minor}$).

IR ν$_{max}$ (cm^{-1}): 3063, 2928, 1697 (C=O), 1636, 1597, 1560 (asym. NO$_2$), 1518, 1449, 1439, 1377, 1341, 1316, 1260 (sym. NO$_2$), 1227, 1069, 1024, 1001.

HRMS (ES+): [M+1]$^+$ calculated for [C$_{24}$H$_{27}$N$_2$O$_4$Si]$^+$: 435.1740, found 435.1747.

Synthesis of (±)-methyl 2-nitro-3-phenyl-3-(phenyl((trimethylsilyl)oxy)amino)propanoate **3x**.

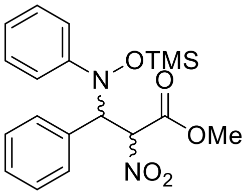

Synthesised according to **GP4** using nitrone **1aa** and nitronate **2i** in dichloromethane for 24 hours. Purification of the crude reaction mixture through flash column chromatography with a 9:1 mixture of cyclohexane:ethyl acetate with 1% Et$_3$N afforded **3x** as a yellow oil (35 mg, 90%). R$_f$ 0.65 (Cy:AcOEt 9:1).

This compound is not stable even on deactivated silica, hence we did a flash purification (residence time inside the column: <3 min). We infer this is due to the strong acidity of the proton α to the nitro group.

^{1}H-NMR (400 MHz, CDCl$_3$, 298 K) δ: 7.29–7.24 (m, 2H, Ar–C**H**), 7.22–7.13 (m, 6H, Ar–C**H**), 7.07–6.95 (m, 8H, Ar–C**H**), 5.89 (d, *J*=10.8, 2H, C**H**–NO$_{2major+minor}$), 5.22–5.17 (m, 2H, C**H**–NOAr $_{major+minor}$), 3.90 (s, 3H, OC**H**$_{3minor}$), 3.45 (s, 3H, OC**H**$_{3major}$), 0.05 (s, 9H, TMS–C**H**$_{3major}$), 0.02 (s, 9H, TMS–C**H**$_{3minor}$).

^{13}C-NMR (101 MHz, CDCl$_3$, 298 K) δ: 163.6 (**C**=O$_{minor}$), 163.0 (**C**=O$_{major}$), 151.5 (Ar–**C**), 151.4 (Ar–**C**), 130.8 (Ar–**C**), 130.3 (Ar–**C**), 130.3 (Ar–**C**), 129.1 (Ar–**C**), 129.0 (Ar–**C**), 128.4 (Ar–**C**), 127.9 (Ar–**C**), 127.7 (Ar–**C**), 125.0 (Ar–**C**), 121.2 (Ar–**C**), 89.4 (**C**–NO$_{2major}$), 88.6 (**C**–NO$_{2minor}$), 74.2 (**C**–NOAr$_{major}$), 73.8 (**C**–NOAr$_{minor}$), 53.9 (O**C**H$_{3minor}$), 53.5 (O**C**H$_{3major}$), -0.38 (TMS–**C**H$_{3major}$), -0.41 (TMS–**C**H$_{3minor}$).

IR ν$_{max}$ (cm^{-1}): 2957, 1755 (C=O), 1562 (asym. NO$_2$), 1485, 1454, 1437, 1364, 1308, 1252 (sym. NO$_2$), 1204, 1167, 1071, 1024.

HRMS (ES+): [M+1]$^+$ calculated for [C$_{19}$H$_{25}$N$_2$O$_5$Si]$^+$: 389.1533, found 389.1542.

Synthesis of (±)-N-((1R,2R)-2-nitro-1-phenyl-2-(phenylsulfonyl)ethyl)-N-phenyl-O-(trimethylsilyl)hydroxylamine **3y**.

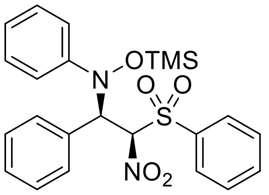

Synthesised according to **GP4** using nitrone **1aa** and nitronate **2j** in dichloromethane for 24 hours. Purification of the crude reaction mixture through flash column chromatography with a 8:2 mixture of cyclohexane:ethyl acetate afforded **3y** as an off white oil (23 mg, 49%). R_f 0.55 (Cy:AcOEt 9:1).

This compound is extremely unstable under basic conditions: 1% of Et_3N in the elution system decomposed the product immediately.

<u>**^{1}H-NMR** (400 MHz, $CDCl_3$, 298 K) δ:</u> 7.60 (tt, *J*=6.9, 1.8, 1H, Ar–C**H**), 7.40–7.34 (m, 4H, Ar–C**H**), 7.29–7.24 (m, 1H, Ar–C**H**), 7.13–7.05 (m, 4H, Ar–C**H**), 7.00 (m, 1H, Ar–C**H**), 6.76–6.74 (m, 4H, Ar–C**H**), 6.33 (d, *J*=11.4, 1H, C**H**–NO_2), 4.88 (d, *J*=11.4, 1H, C**H**–NOAr), 0.02 (s, 9H, TMS–C**H**$_3$).

<u>**^{13}C-NMR** (101 MHz, $CDCl_3$, 298 K) δ:</u> 150.8 (Ar–**C**), 135.7 (Ar–**C**), 135.0 (Ar–**C**), 132.0(Ar–**C**), 129.9 (Ar–**C**), 129.7 (Ar–**C**), 129.5(Ar–**C**), 129.3 (Ar–**C**), 129.2 (Ar–**C**), 128.4 (Ar–**C**), 127.7 (Ar–**C**), 127.3 (Ar–**C**), 125.3 (Ar–**C**), 121.2 (Ar–**C**), 103.6 (**C**–NO_2), 74.2 (**C**–NOAr), -0.1 (TMS–**C**H_3).

<u>**IR** ν_{max} (cm^{-1})</u>: 2957, 2924, 2855, 1568 (asym. NO_2), 1485, 1449, 1346 (asym. SO_2), 1252 (sym. NO_2), 1190, 1157 (sym. SO_2), 1082, 1024.

HRMS (ES+): $[M+1]^+$ calculated for $[C_{23}H_{27}N_2O_5SiS]^+$: 471.1410, found 471.1410.

Synthesis of (±)-N-((1R,2R)-2-cyclobutyl-2-nitro-1-phenylethyl)-N-phenyl-O-(trimethylsilyl) hydroxylamine **3z**.

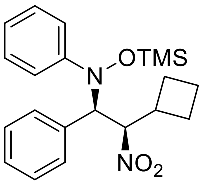

Synthesised according to **GP4** using nitrone **1aa** and nitronate **2k** in dichloromethane for 24 hours. Purification of the crude reaction mixture through flash column chromatography with a 8:2 mixture of cyclohexane:ethyl acetate afforded **3z** as an off white oil (23 mg, 49%). R_f 0.91 (Cy:AcOEt 9:1).

<u>**^{1}H-NMR** (400 MHz, $CDCl_3$, 298 K) δ:</u> 7.26 (dt, *J*=14.7, 1.3, 1H, Ar–C**H**), 7.20–7.16 (m, 2H, Ar–C**H**), 7.14–7.09 (m, 2H, Ar–C**H**), 7.00–6.96 (m, 3H, Ar–C**H**), 6.90–6.88 (m, 2H, Ar–C**H**), 5.17 (dd, *J*=10.8, 7.7, 1H, C**H**–NO_2), 4.65 (d, *J*=10.9, 1H, C**H**–NOAr), 2.49–2.38 (m, 1H, C**H**), 2.05–1.95 (m, 1H, C**H**$_2$), 1.70–1.50 (m, 4H, C**H**$_2$), 1.26–1.22 (m, 1H, C**H**$_2$), 0.07 (s, 9H, TMS–C**H**$_3$).

<u>**^{13}C-NMR** (101 MHz, $CDCl_3$, 298 K) δ:</u> 152.1 (Ar–**C**), 131.8 (Ar–**C**), 131.0 (Ar–**C**), 128.5 (Ar–**C**), 128.2 (Ar–**C**), 127.6 (Ar–**C**), 124.6 (Ar–**C**), 121.2 (Ar–**C**), 92.8 (**C**–NO_2), 76.3 (**C**–NOAr), 37.3 (**C**H), 27.1 (**C**H_2), 24.3 (**C**H_2), 17.3 (**C**H_2), -0.2 (TMS–**C**H_3).

<u>**IR** ν_{max} (cm^{-1})</u>: 3032, 2953, 2870, 1595, 1551 (asym. NO_2), 1485, 1452, 1377, 1250 (sym. NO_2), 1202, 1072, 1026.

HRMS (ES+): $[M+1]^+$ calculated for $[C_{21}H_{29}N_2O_3Si]^+$: 385.1947, found 385.1949.

Synthesis of (±)-N-((1S,2R)-2-cyclobutyl-2-nitro-1-phenylethyl)-N-phenyl-O-(trimethylsilyl)hydroxyl amine **3z'**.

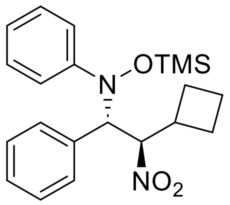

Synthesised according to **GP4** using nitrone **1aa** and nitronate **2k** in dichloromethane for 24 hours. Purification of the crude reaction mixture through flash column chromatography with a 8:2 mixture of cyclohexane:ethyl acetate afforded **3z'** as a white solid (3 mg, 8%). R_f 0.89 (Cy:AcOEt 9:1).

^{1}H-NMR (400 MHz, $CDCl_3$, 298 K) δ: 7.22–7.13 (m, 5H, Ar–C**H**), 7.03–6.96 (m, 3H, Ar–C**H**), 6.87–6.85 (m, 2H, Ar–C**H**), 5.29 (dd, *J*=10.4, 4.5, 1H, C**H**–NO_2), 4.43 (d, *J*=10.4, 1H, C**H**–NOAr), 3.57–3.47 (m, 1H, C**H**), 2.21–2.09 (m, 3H, C**H**$_2$), 1.89–1.86 (m, 2H, C**H**$_2$), 1.78–1.72 (m, 1H, C**H**$_2$), 0.05 (s, 9H, TMS–C**H**$_3$).

^{13}C-NMR (101 MHz, $CDCl_3$, 298 K) δ: 152.3 (Ar–**C**), 132.6 (Ar–**C**), 130.0 (Ar–**C**), 128.4 (Ar–**C**), 128.4 (Ar–**C**), 127.6(Ar–**C**), 124.3 (Ar–**C**), 120.3 (Ar–**C**), 90.5 (**C**–NO_2), 73.4 (**C**–NOAr), 36.3 (**C**H), 25.2 (**C**H_2), 23.5 (**C**H_2), 16.9(**C**H_2), 0.0 (TMS–**C**H_3).

IR ν_{max} (cm^{-1}): 2955, 1595, 1549 (asym. NO_2), 1485, 1454, 1375, 1252 (sym. NO_2), 1204, 1024.

HRMS (CI): $[M]^+$ calculated for $[C_{21}H_{28}N_2O_3Si]^+$: 385.18637, found 385.1862.

6. Further reactivity:

1g scale reaction for the synthesis of (±)-N-2-nitro-1-phenylbutyl)-N-phenyl-O-(trimethylsilyl)hydroxylamine **3a** and **3a'**.

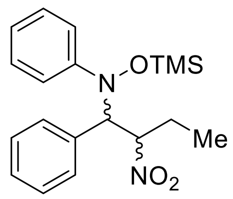

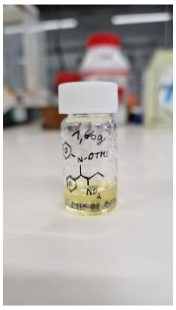

Synthesised according to **GP4** using nitrone **1aa** (1.00 g, 5.07mmol) and nitronate **2a** (1.64 g, 10.14 mmol) in dichloromethane for 24 hours. Purification of the crude reaction mixture with column chromatography with a 9:1 mixture of cyclohexane:ethyl acetate afforded a mixture of **3a** and **3a'** as a yellow oil (1.66 g, 91%).

Synthesis of (±)-N-(2-nitro-1-phenylbutyl)-N-phenylhydroxylamine **4**.

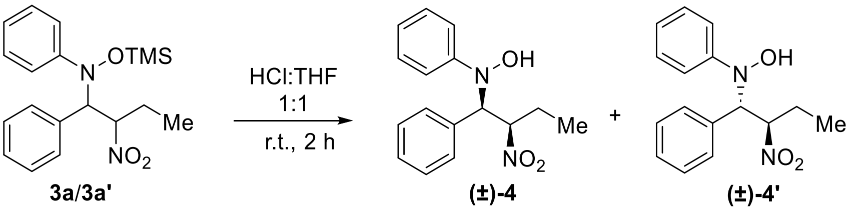

3a/3a' **(±)-4** **(±)-4'**

Following a reported method,[14] *N*-(2-nitro-1-phenylbutyl)-*N*-phenyl-*O*-(trimethylsilyl) hydroxylamine **3a/3a'** (0.2 mmol) was dissolved in 2 mL of a 1:1 mixture of HCl (1 M) and THF. The resulting mixture was left to stir at room temperature for 2 hours. Then, the reaction was

quenched with a saturated solution of $NaHCO_3$ until a pH of ~8 was reached. The aqueous layer was extracted 3 times with EtOAc, the organic layers where then collected, washed with brine, and dried over $MgSO_4$. The solvent was subsequently removed affording 55 mg of yellow oil which contained a mixture of the major and minor hydroxylamine derivatives in a diasteromeric ratio of 92:8. Only for the purpose of this work, the diasteroisomers where separated through a flash column chromatography using a 9:1 mixture of cyclohexane/EtOAc. During the purification, we observed decomposition of the minor diasteroisomer (**4'**). After column chromatography, 43 mg of a colourless oil of compound **4** was obtained (75%). R*f*: 0.46 (Cy:AcOEt 9:1).

^{1}H-NMR (400 MHz, $CDCl_3$, 298 K) δ: 7.29–7.22 (m, 3H, Ar–C**H**), 7.18–7.12 (m, 4H, Ar–C**H**), 6.93–6.91 (m, 3H, Ar–C**H**), 5.46 (td, *J*=11.1, 2.9, 1H, C**H**–NO_2), 5.19 (br s, 1H, O**H**), 4.75 (d, *J*=10.9, 1H, C**H**–NOAr), 1.90 (m, 1H, C**H**$_2$), 1.52 (m, 1H, C**H**$_2$), 0.96 (t, *J*=7.3, 3H, C**H**$_3$).

^{13}C-NMR (101 MHz, $CDCl_3$, 298 K) δ: 150.4 (Ar–**C**), 131.9 (Ar–**C**), 129.8 (Ar–**C**), 128.8 (Ar–**C**), 128.5 (Ar–**C**), 128.3 (Ar–**C**), 123.1 (Ar–**C**), 118.0 (Ar–**C**), 91.5 (**C**–NO_2), 74.5 (**C**–NOAr), 24.7 (**C**H$_2$), 10.5 (**C**H$_3$).

IR ν_{max} (cm^{-1}): 3499 (OH), 3063, 3030, 2972, 2938, 1597, 1549 (NO), 1489, 1452, 1373, 1344, 1314, 1262, 1217, 1194, 1129, 1086, 1030.

HRMS (CI): $[M]^+$ calculated for $[C_{16}H_{18}N_2O_3]^+$: 286.13119, found 286.1311.

Synthesis of (±)-N1,1-diphenylbutane-1,2-diamine **5**.

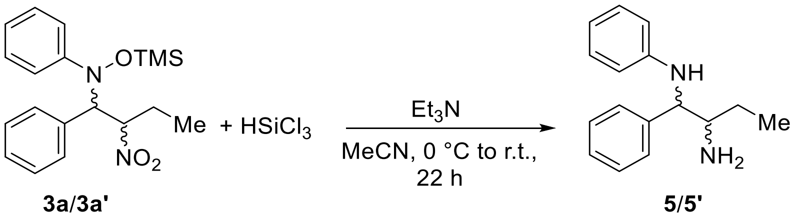

3a/3a' **5/5'**

Adapting a reported method,[15] *N*-(2-nitro-1-phenylbutyl)-*N*-phenyl-*O*-(trimethylsilyl) hydroxylamine **3a/3a'** (0.2 mmol, 1 equiv.) was dissolved in 2 mL of anhydrous and degassed MeCN. The solution was cooled to 0 °C and $HSiCl_3$ (0.07 mL, 3.5 equiv.) was added. Subsequently, Et_3N (0.14 mL, 5 equiv.) was added dropwise under vigorous stirring. The resulting mixture was left to stir for further 10 minutes at 0 °C and then at room temperature overnight. The reaction was then quenched with a saturated solution of $NaHCO_3$ until a pH of ~8 was reached. . The aqueous layer was extracted 3 times with EtOAc, the organic layers where then collected, washed with brine, and dried over $MgSO_4$. The solvent was subsequently removed affording 43 mg of yellow oil which contained a mixture of the major and minor diamine derivatives in a diasteromeric ratio of 80:20. The crude reaction mixture could be further purified by an H_3O^+/OH^- aqueous workup affording 33 mg of pale yellow oil. For the purpose of this study, no column chromatography was undertaken as the diamine product could be obtained in relatively high purity. R*f*: 0.29 (DCM + 5% MeOH).

^{1}H-NMR (400 MHz, $CDCl_3$, 298 K) δ: 7.32–7.10 (m, 7H, Ar–C**H**), 7.01–6.91 (m, 2H, Ar–C**H**), 6.53 (tt, *J*=7.3, 1.1, 1H, Ar–C**H**), 6.48–6.41 (m, 2H, Ar–C**H**$_{major}$), 5.05 (br s, 1H, N**H**) 4.28 (d, *J*=4.4, 1H, C**H**–NH$_{minor}$), 4.22 (d, *J*=3.9, 1H, C**H**–NH$_{major}$), 2.98 (m, 1H, C**H**–NH$_{2major}$), 2.94–2.88 (m, 1H, C**H**–NH$_{2minor}$), 2.41–2.15 (br s, 2H, N**H**$_2$) 1.63–1.51 (m, 1H, C**H**$_{2major+minor}$), 1.44–1.35 (m, 1H, C**H**$_{2major+minor}$), 0.91 (t, *J*=7.5, 3H, C**H**$_{3major+minor}$).

^{13}C-NMR (101 MHz, $CDCl_3$, 298 K) δ: 147.8 (Ar–**C**), 142.5 (Ar–**C**), 129.2 (Ar–**C**), 129.1 (Ar–**C**), 129.0 (Ar–**C**), 128.9 (Ar–**C**), 128.8 (Ar–**C**), 128.7 (Ar–**C**), 128.4 (Ar–**C**), 127.9 (Ar–**C**), 127.3 (Ar–

C), 127.1 (Ar–**C**), 126.93 (Ar–**C**), 126.87 (Ar–**C**), 116.8 (Ar–**C**), 114.3 (Ar–**C**), 113.5 (Ar–**C**), 113.2 (Ar–**C**), 61.4 (**C**H–NH*minor*), 60.5 (**C**H–NH*major*), 58.6 (**C**H–NH_{2major}), 57.6 (**C**H–NH_{2minor}), 27.6 (**C**$H_{2major+minor}$), 11.1 (**C**$H_{3major+minor}$).

<u>IR ν_{max}</u> <u>(cm^{-1})</u>: 3358 (NH_2), 3051, 3024, 2961, 2930, 2874, 1599 (NH), 1501, 1451, 1427, 1379, 1317, 1260, 1179, 1153, 1076, 1028.

HRMS (ES+): $[M+1]^+$ calculated for $[C_{16}H_{21}N_2]^+$: 241.1705, found 241.1709.

.

7. NMR Spectra

Figure S1. ^{1}H-NMR (400 MHz, $CDCl_3$, 298 K) spectrum of *(nitromethyl)cyclobutane***.**

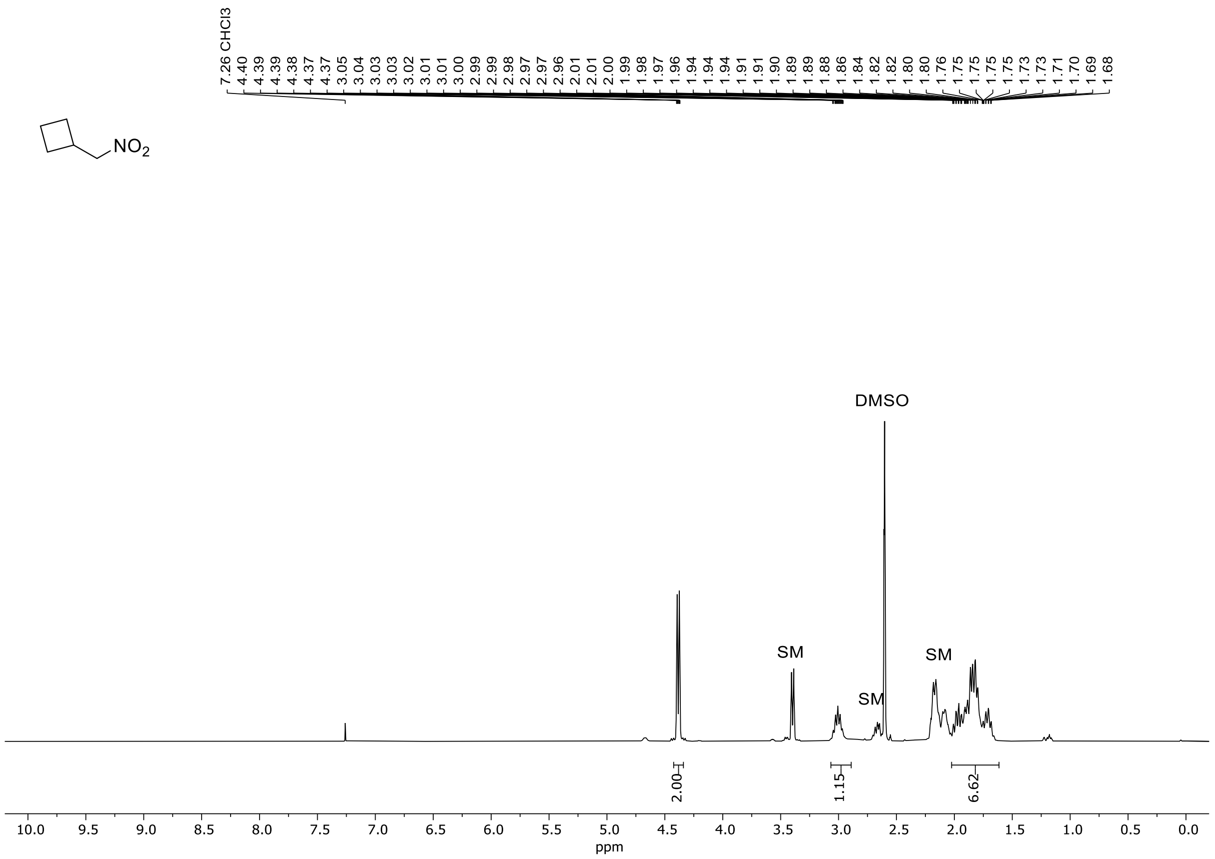

Figure S2. ^{13}C-NMR (101 MHz, $CDCl_3$, 298 K) spectrum of *(nitromethyl)cyclobutane***.**

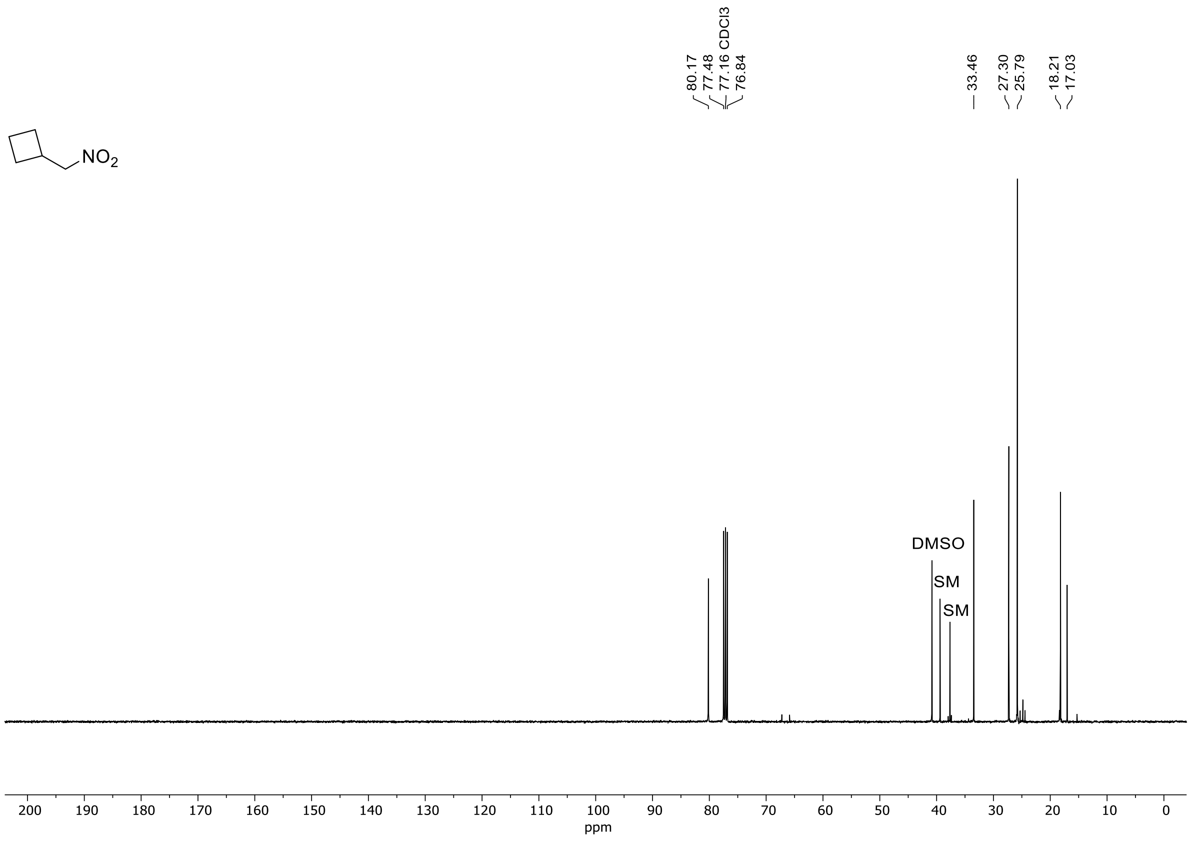

Figure S3. ^{1}H-NMR (400 MHz, $CDCl_3$, 298 K) spectrum of **1aj**.

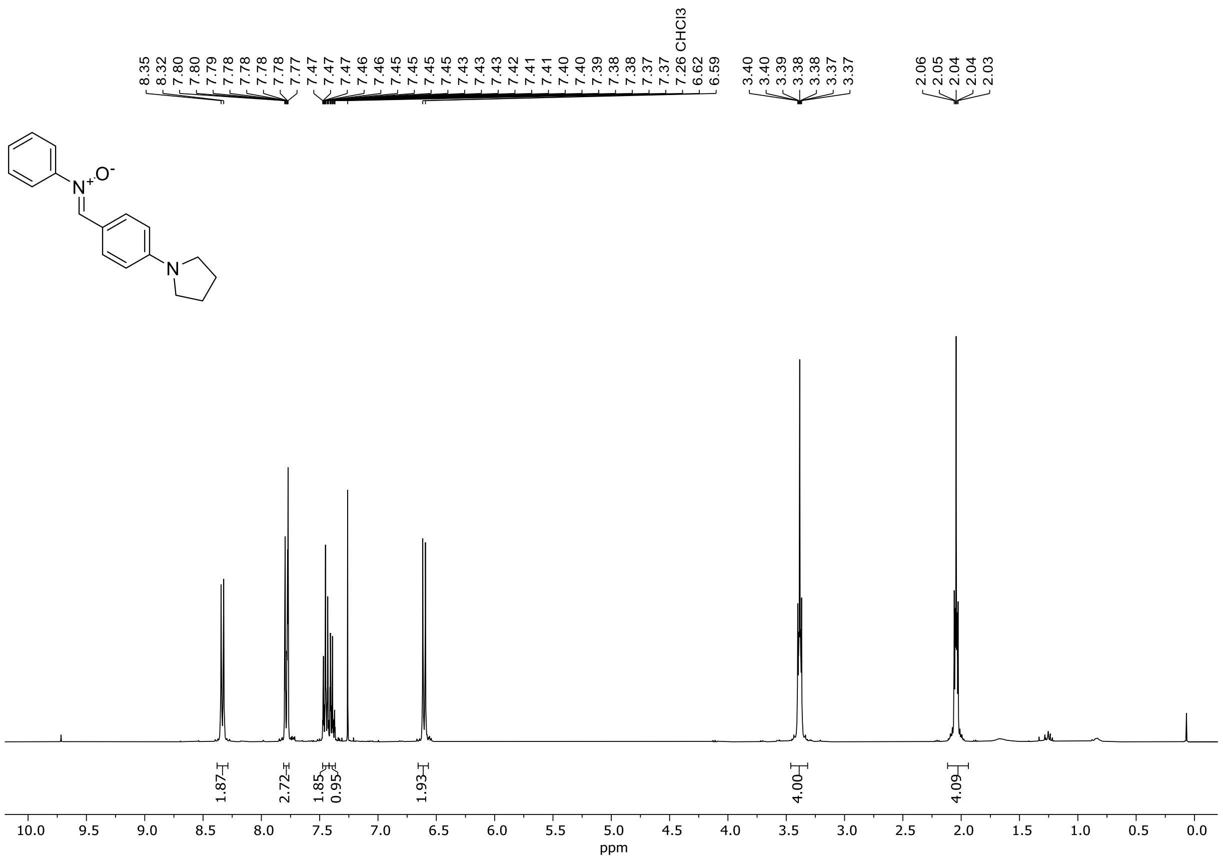

Figure S4. ^{13}C-NMR (101 MHz, $CDCl_3$, 298 K) spectrum of **1aj**.

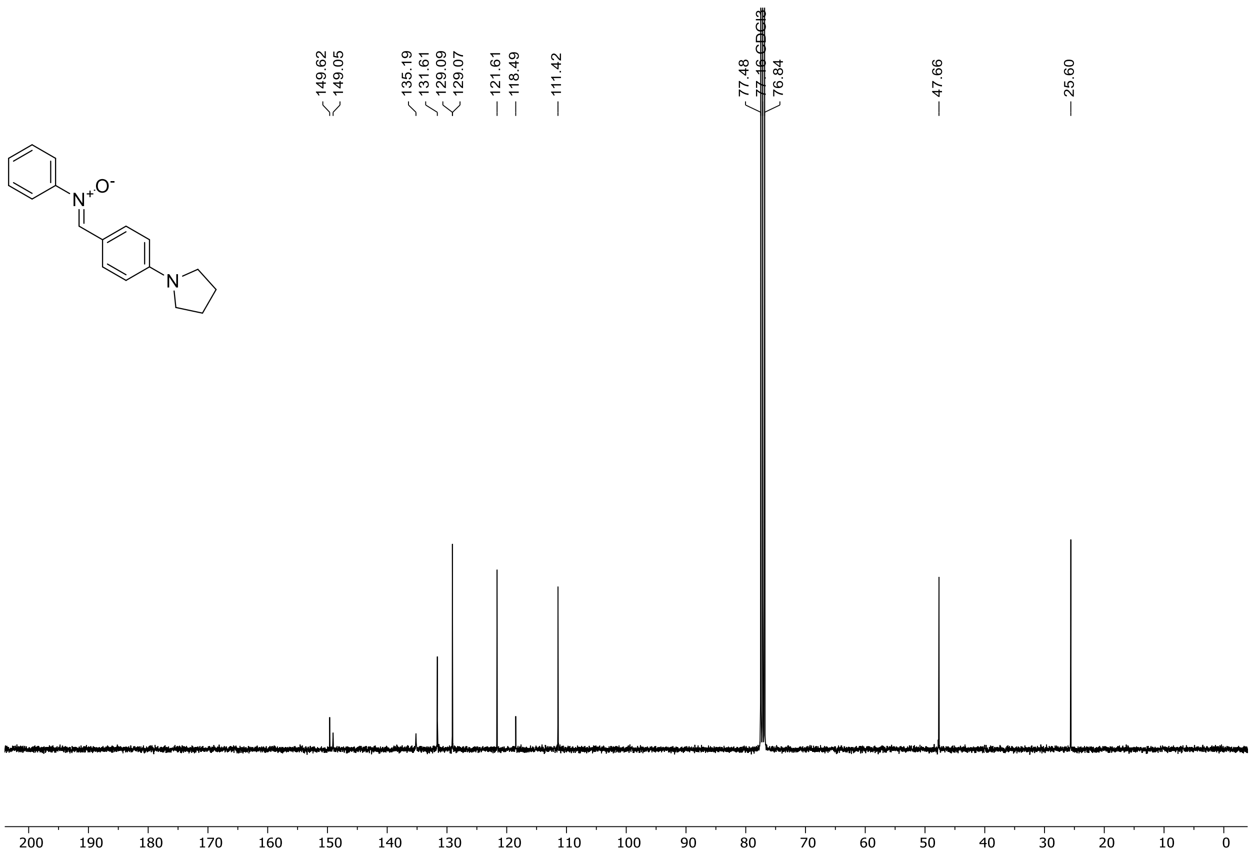

Figure S5. ^{1}H-NMR (400 MHz, $CDCl_3$, 298 K) spectrum of **1ak**.

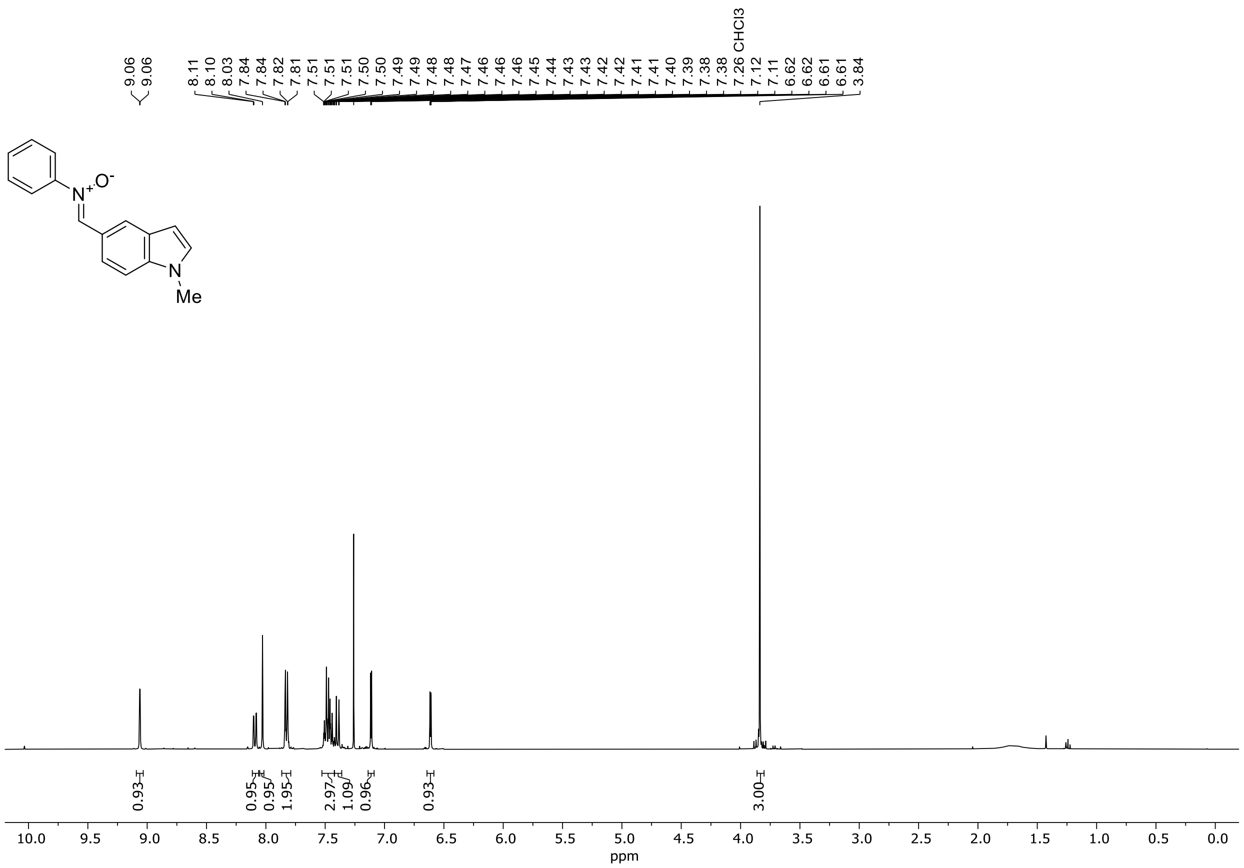

Figure S6. ^{13}C-NMR (101 MHz, $CDCl_3$, 298 K) spectrum of **1ak**.

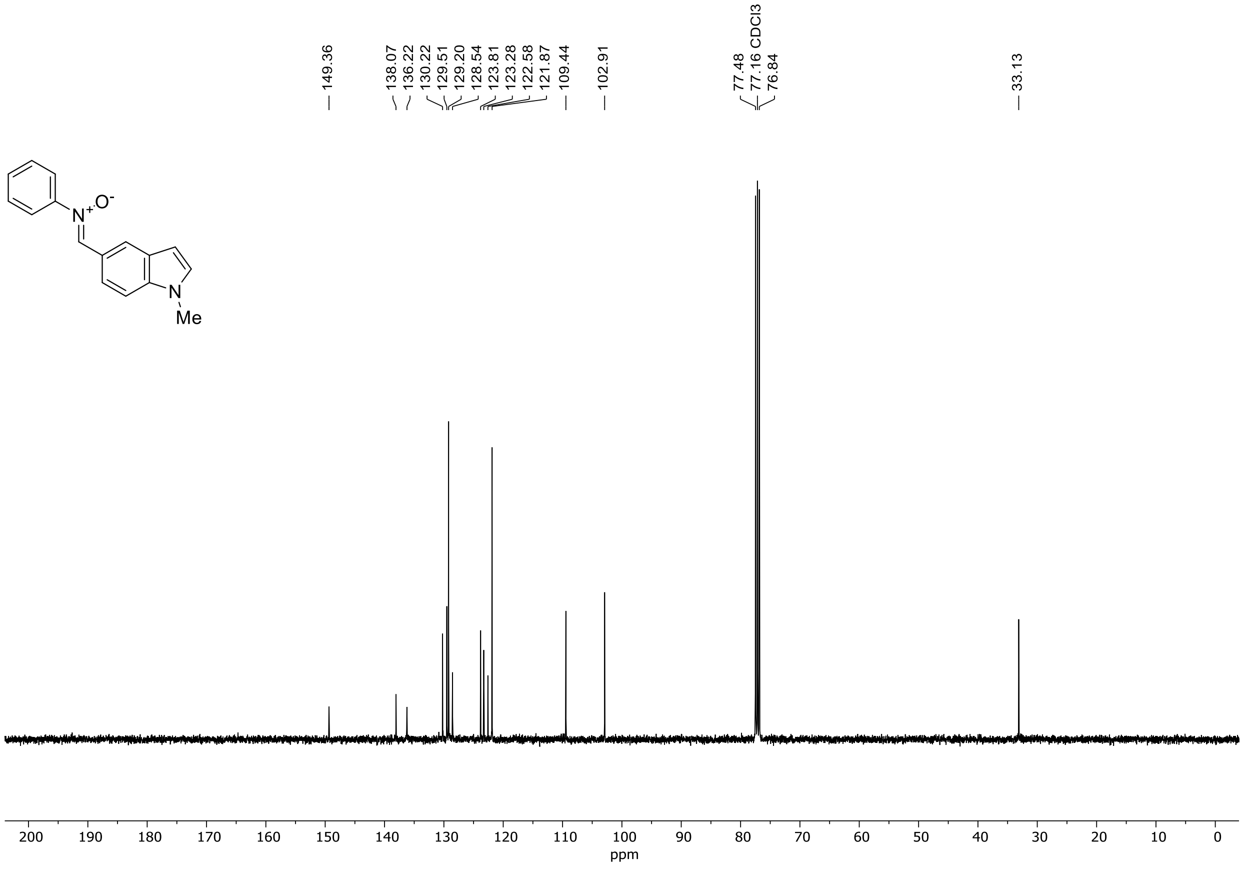

Figure S7. ^{1}H-NMR (400 MHz, $CDCl_3$, 298 K) spectrum of **1al**.

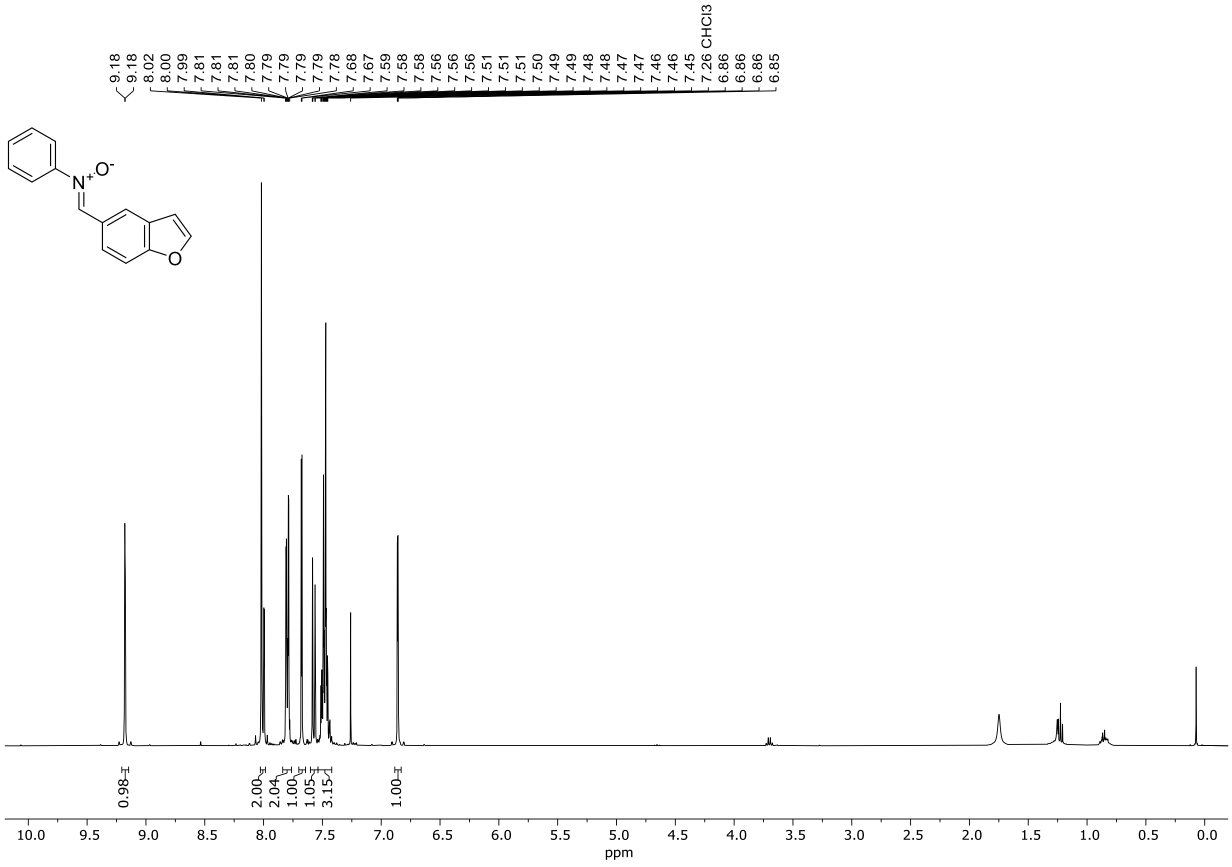

Figure S8. ^{13}C-NMR (101 MHz, $CDCl_3$, 298 K) spectrum of **1al**.

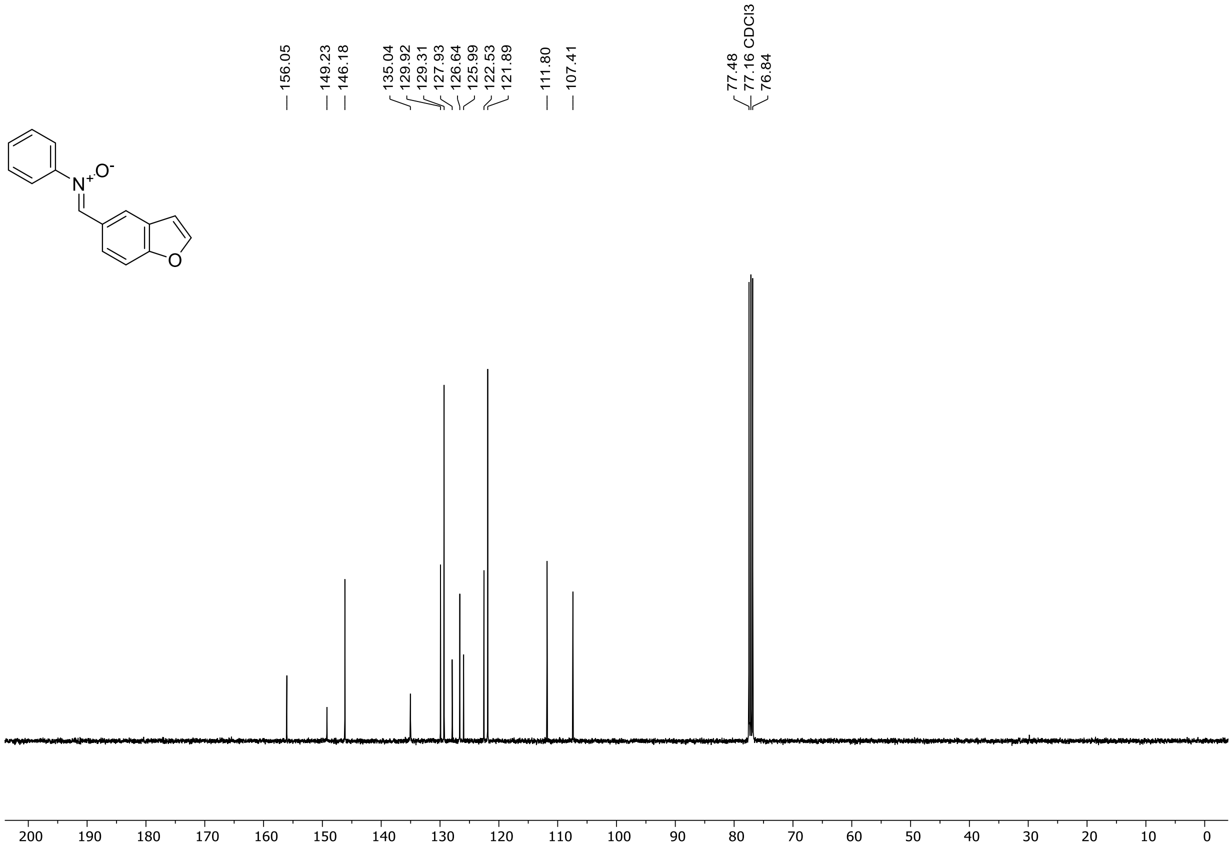

Figure S9. ^{1}H-NMR (400 MHz, $CDCl_3$, 298 K) spectrum of **1aq**.

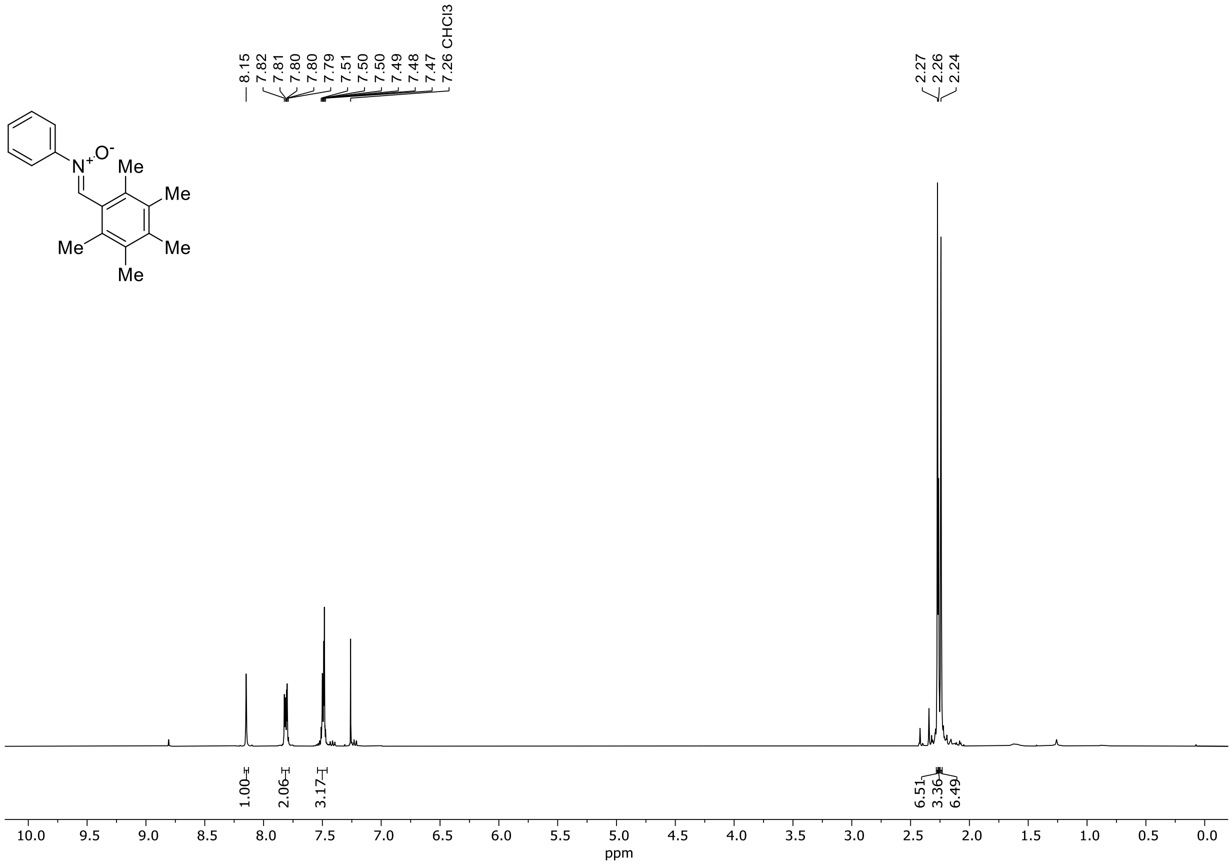

Figure S10. ^{13}C-NMR (101 MHz, $CDCl_3$, 298 K) spectrum of **1aq**.

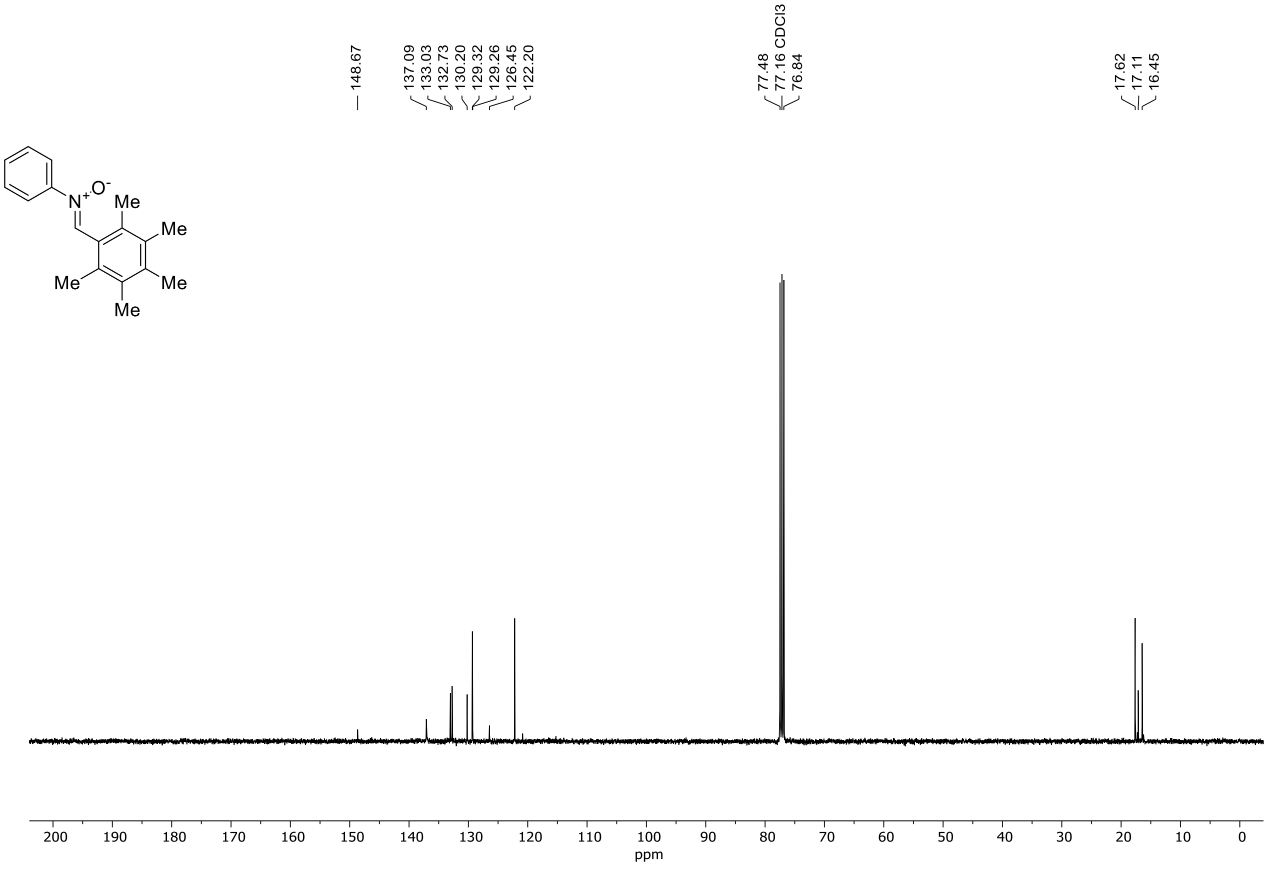

Figure S11. ^{1}H-NMR (400 MHz, $CDCl_3$, 298 K) spectrum of **1ax**.

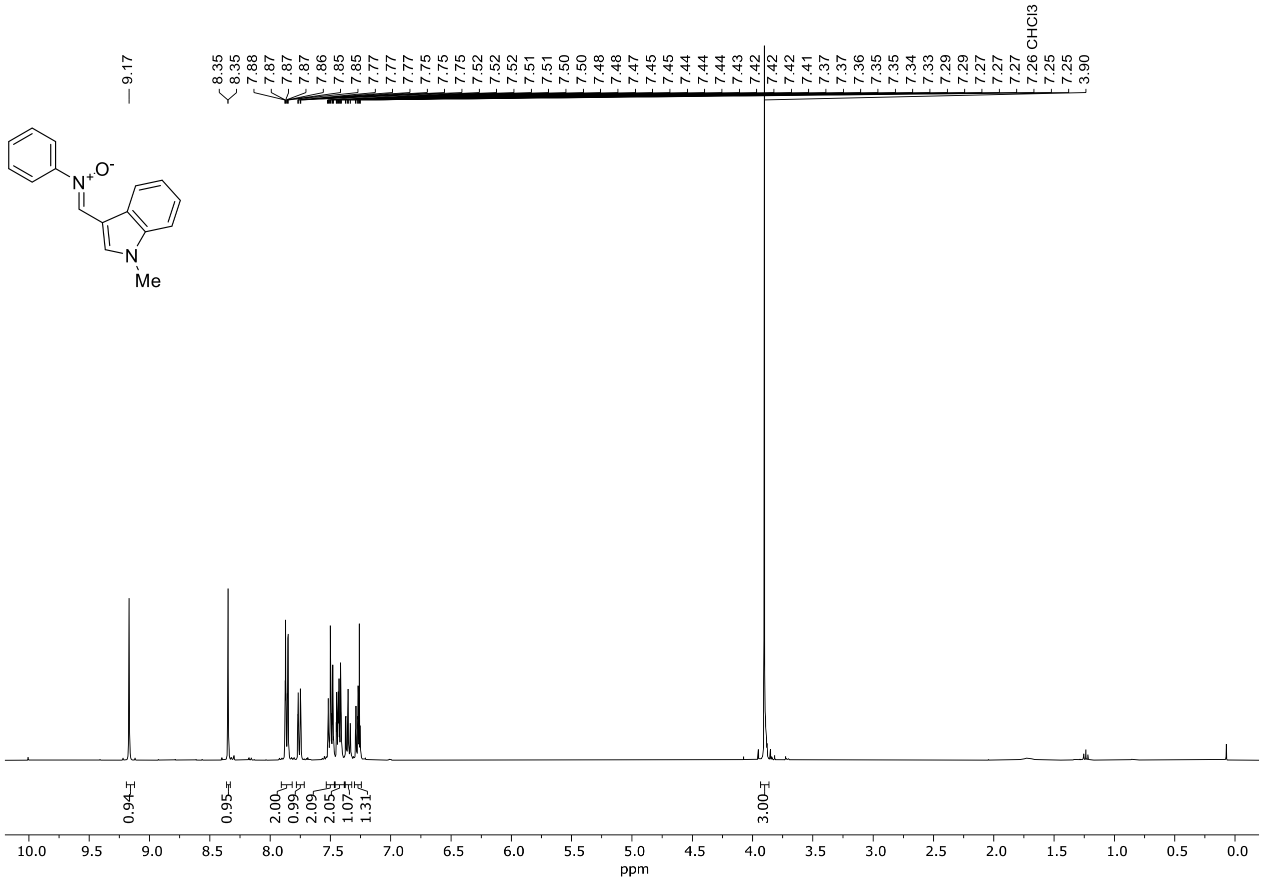

Figure S12. ^{13}C-NMR (101 MHz, $CDCl_3$, 298 K) spectrum of **1ax**.

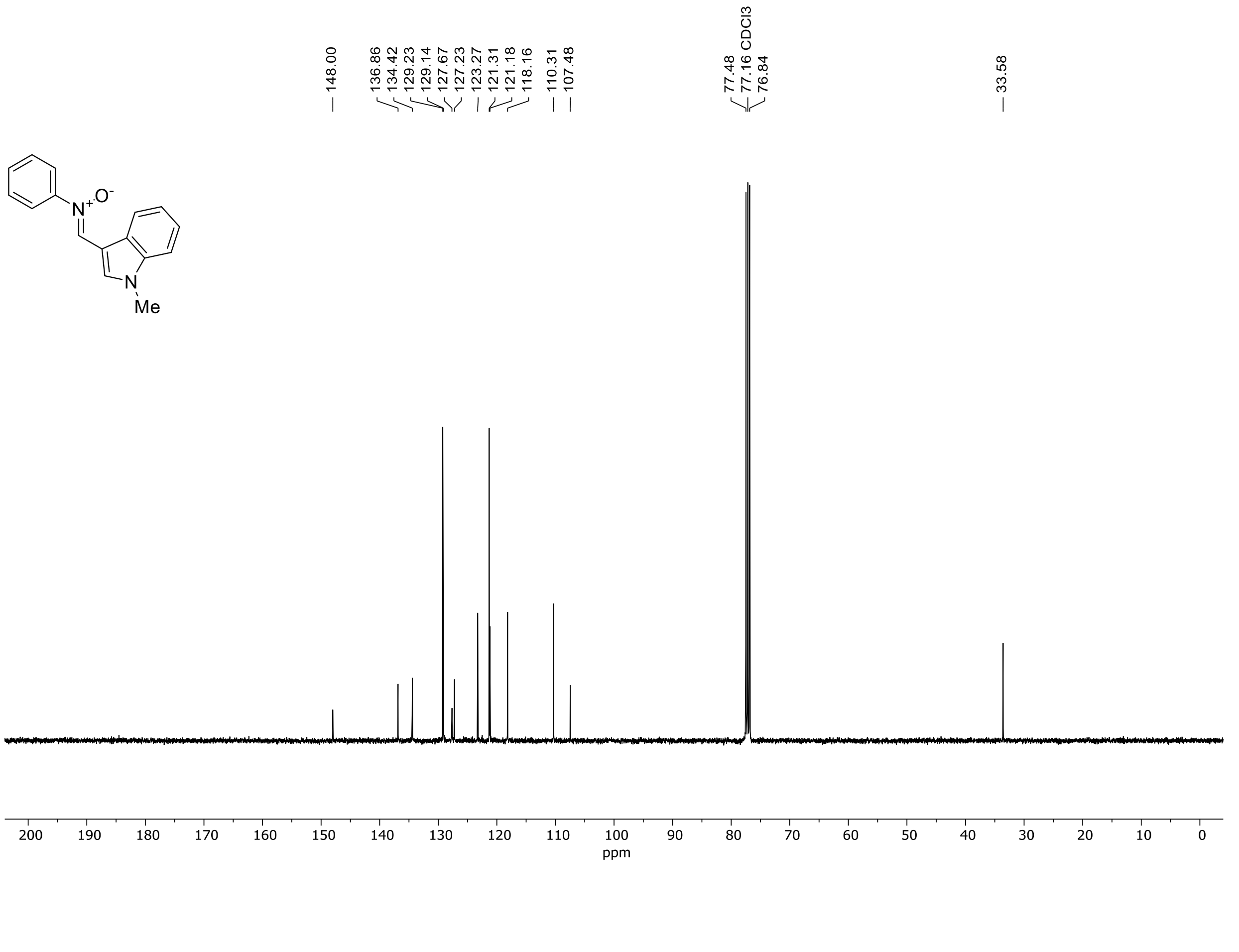

Figure S13. ^{1}H-NMR (400 MHz, $CDCl_3$, 298 K) spectrum of **2a**.

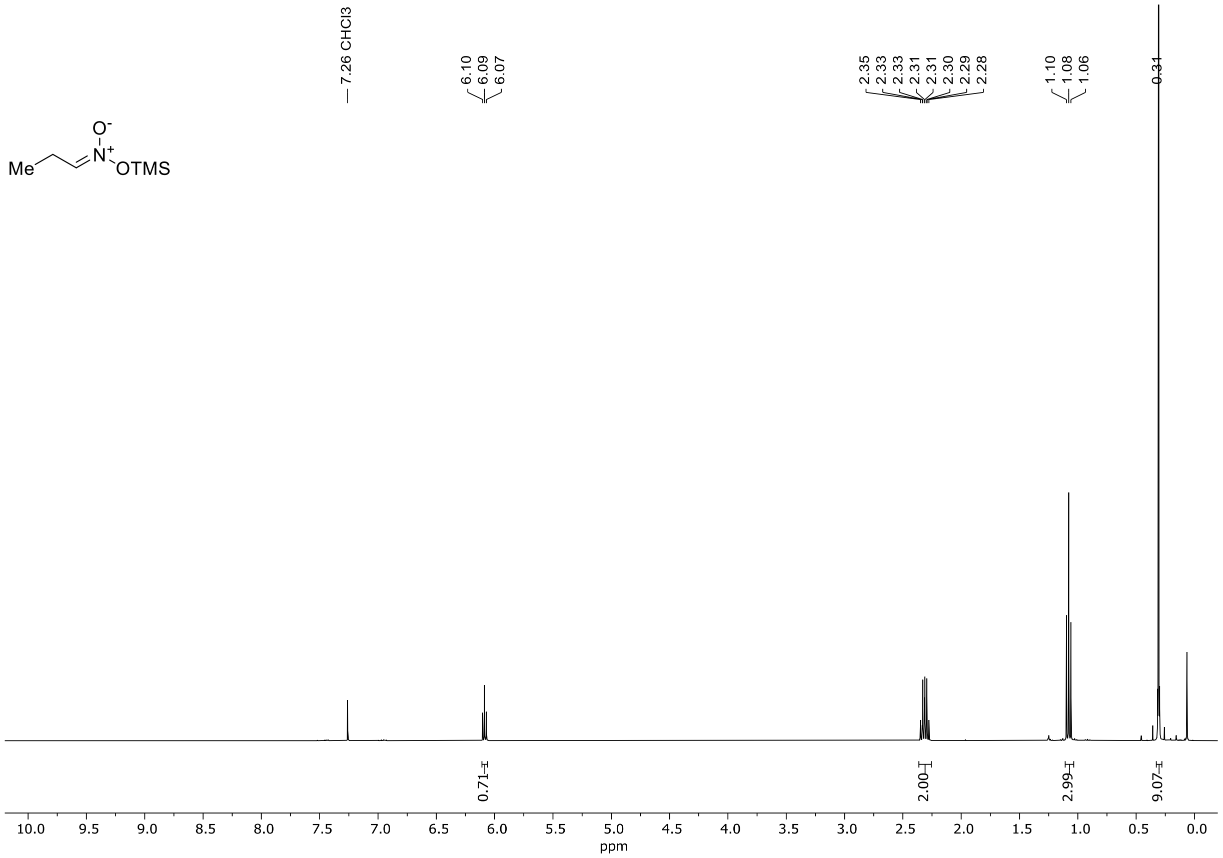

Figure S14. ^{13}C-NMR (101 MHz, $CDCl_3$, 298 K) spectrum of **2a**.

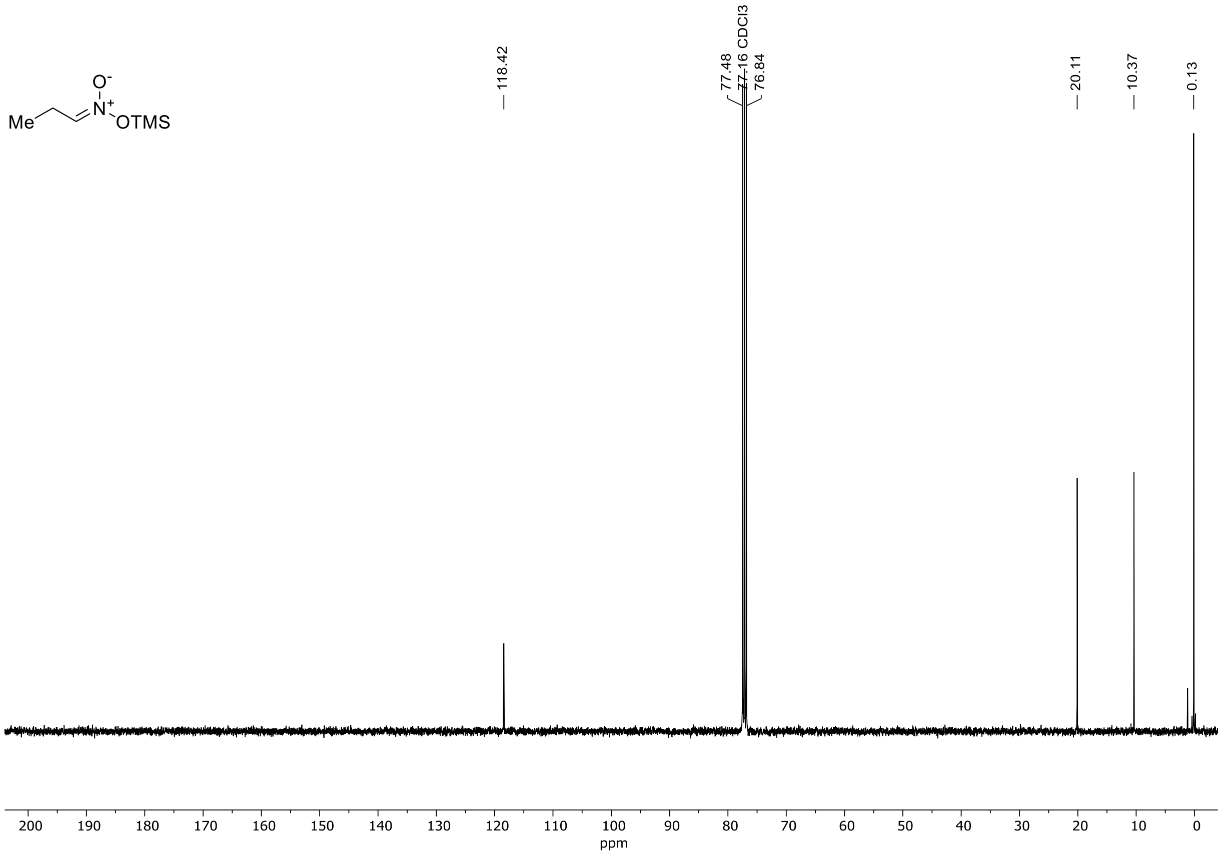

Figure S15. ^{1}H-NMR (400 MHz, $CDCl_3$, 298 K) spectrum of **2b**.

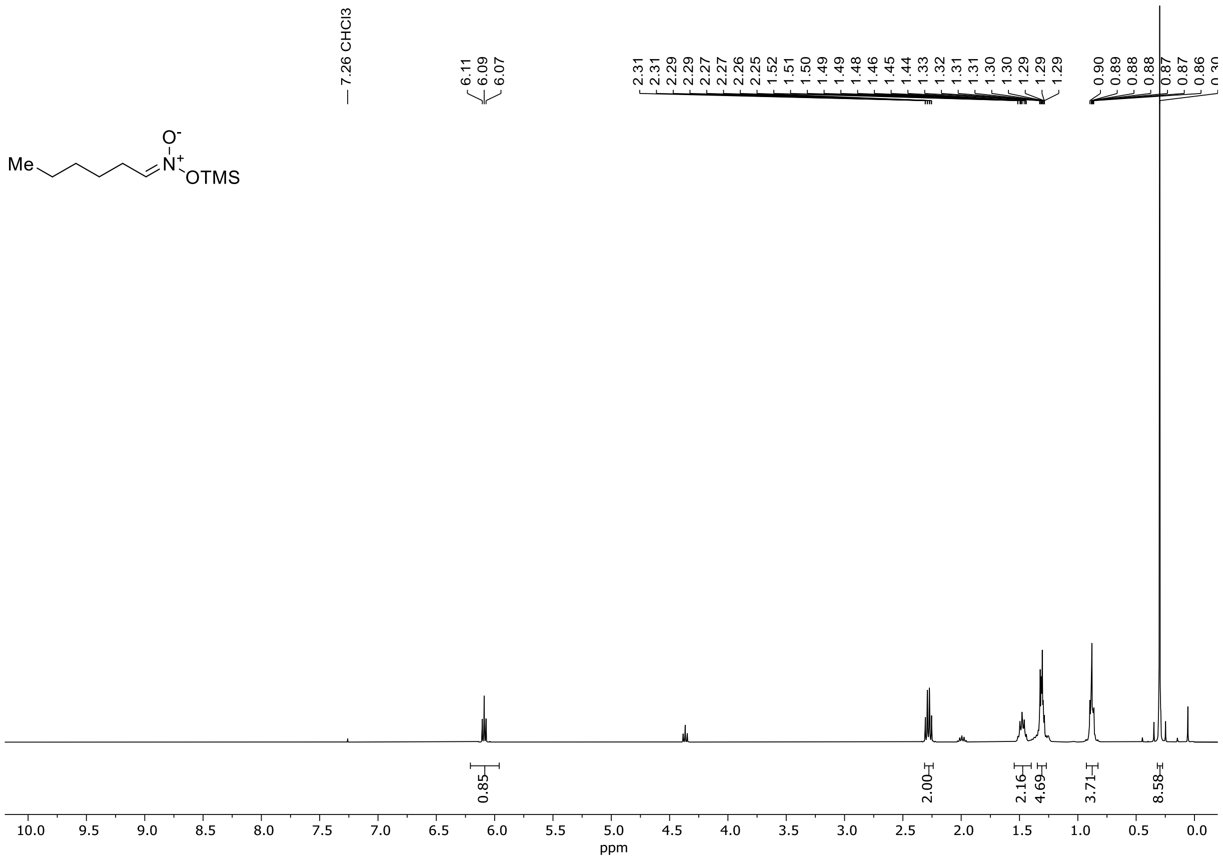

Figure S16. ^{13}C-NMR (101 MHz, $CDCl_3$, 298 K) spectrum of **2b**.

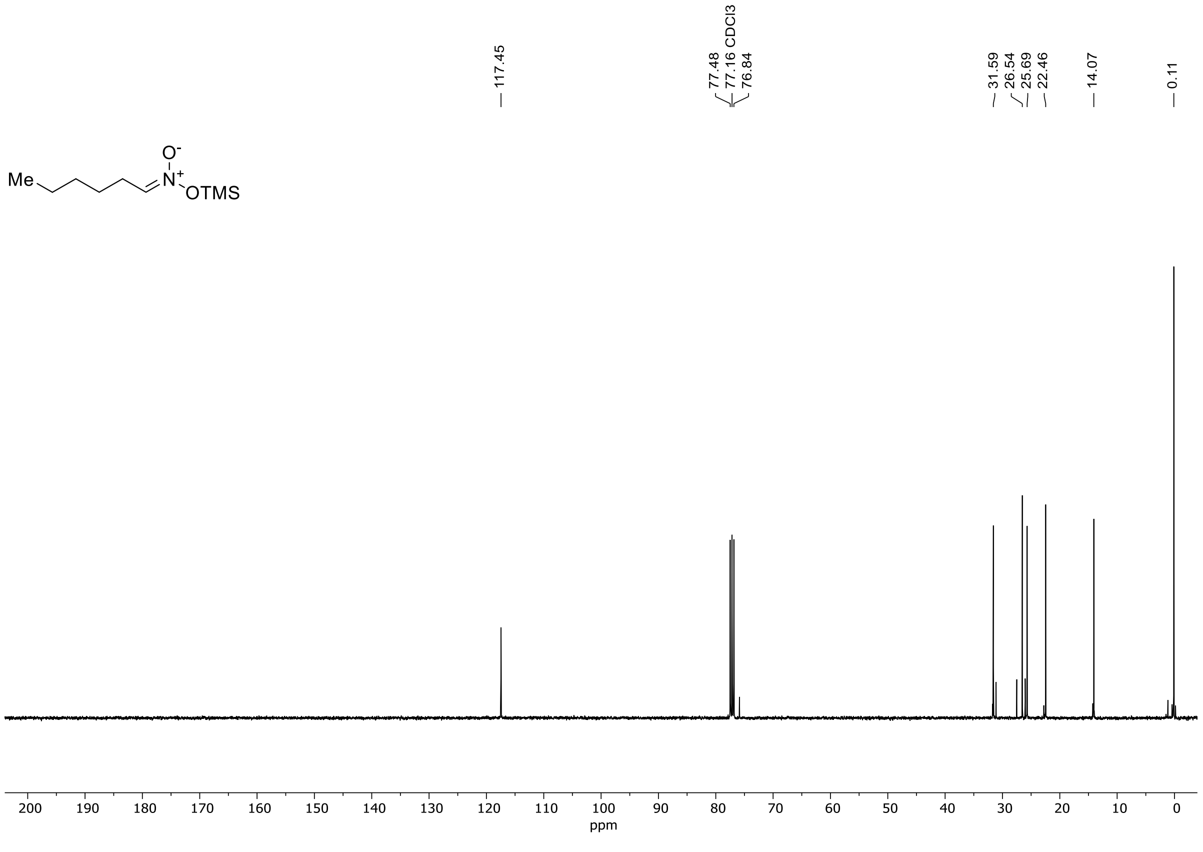

Figure S17. ^{1}H-NMR (400 MHz, $CDCl_3$, 298 K) spectrum of **2c**.

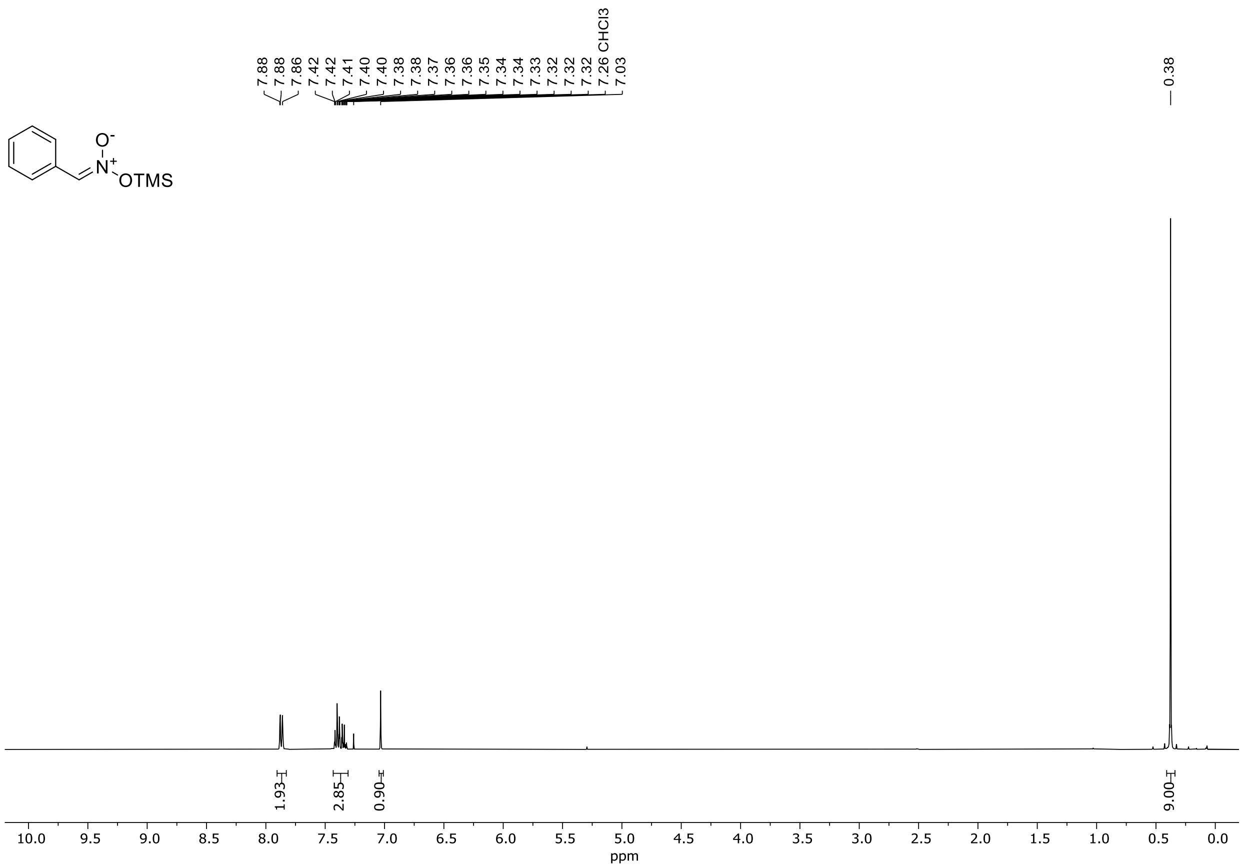

Figure S18. ^{13}C-NMR (101 MHz, $CDCl_3$, 298 K) spectrum of **2c**.

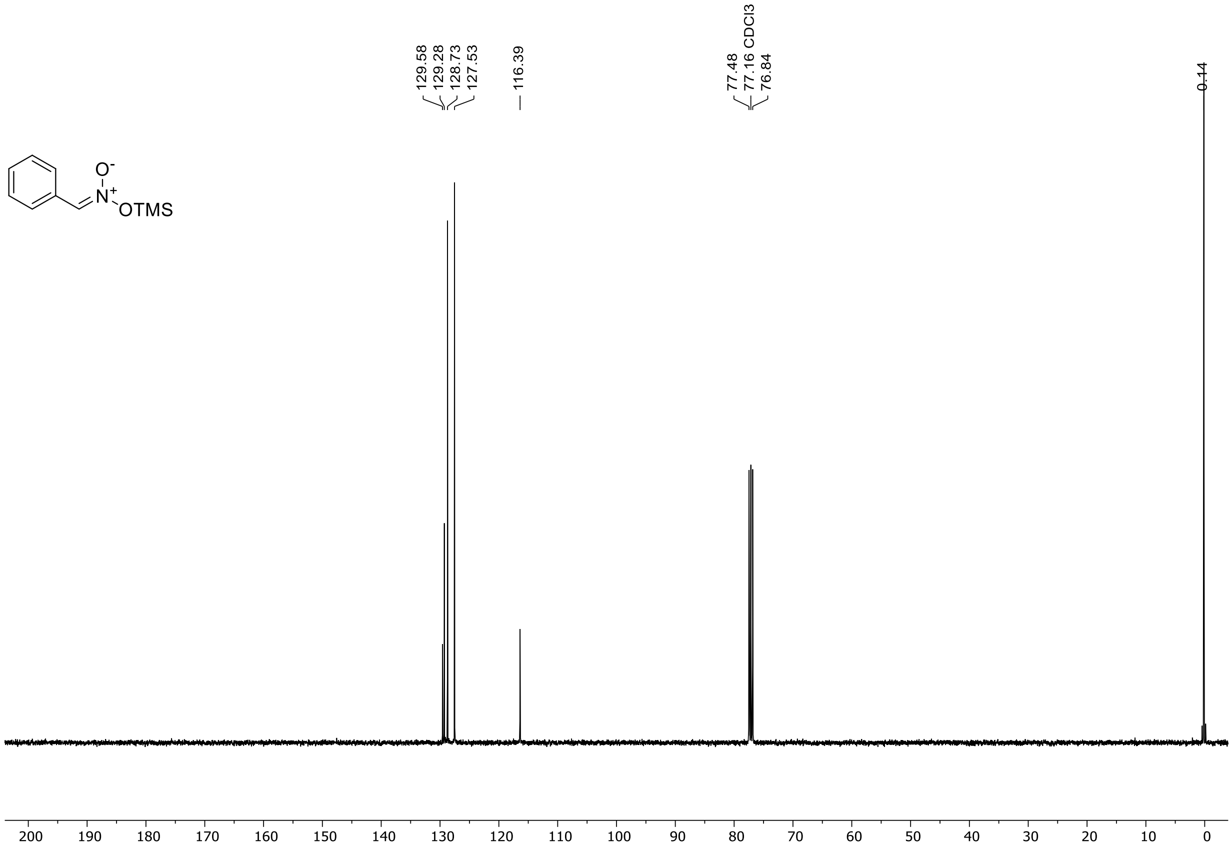

Figure S19. ^{1}H-NMR (400 MHz, $CDCl_3$, 298 K) spectrum of **2d**.

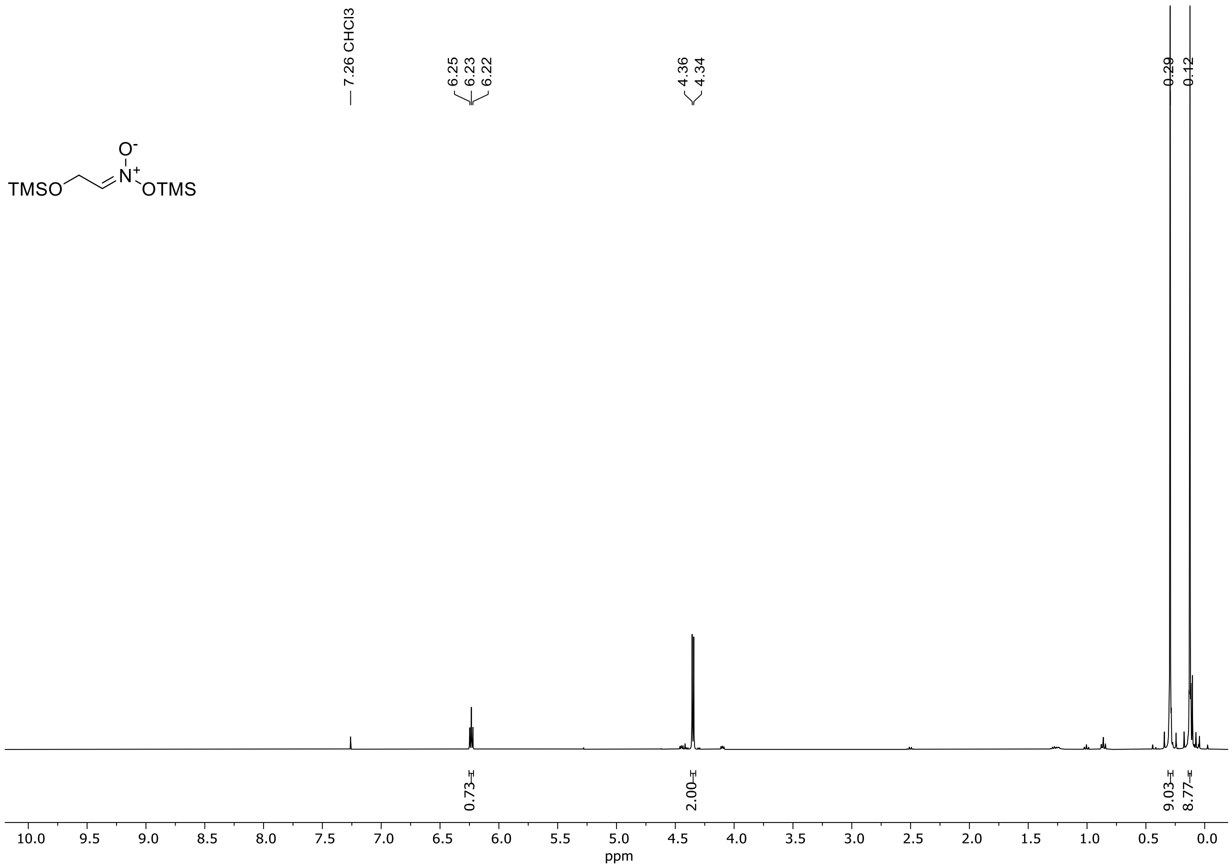

Figure S20. ^{13}C-NMR (101 MHz, $CDCl_3$, 298 K) spectrum of **2d**.

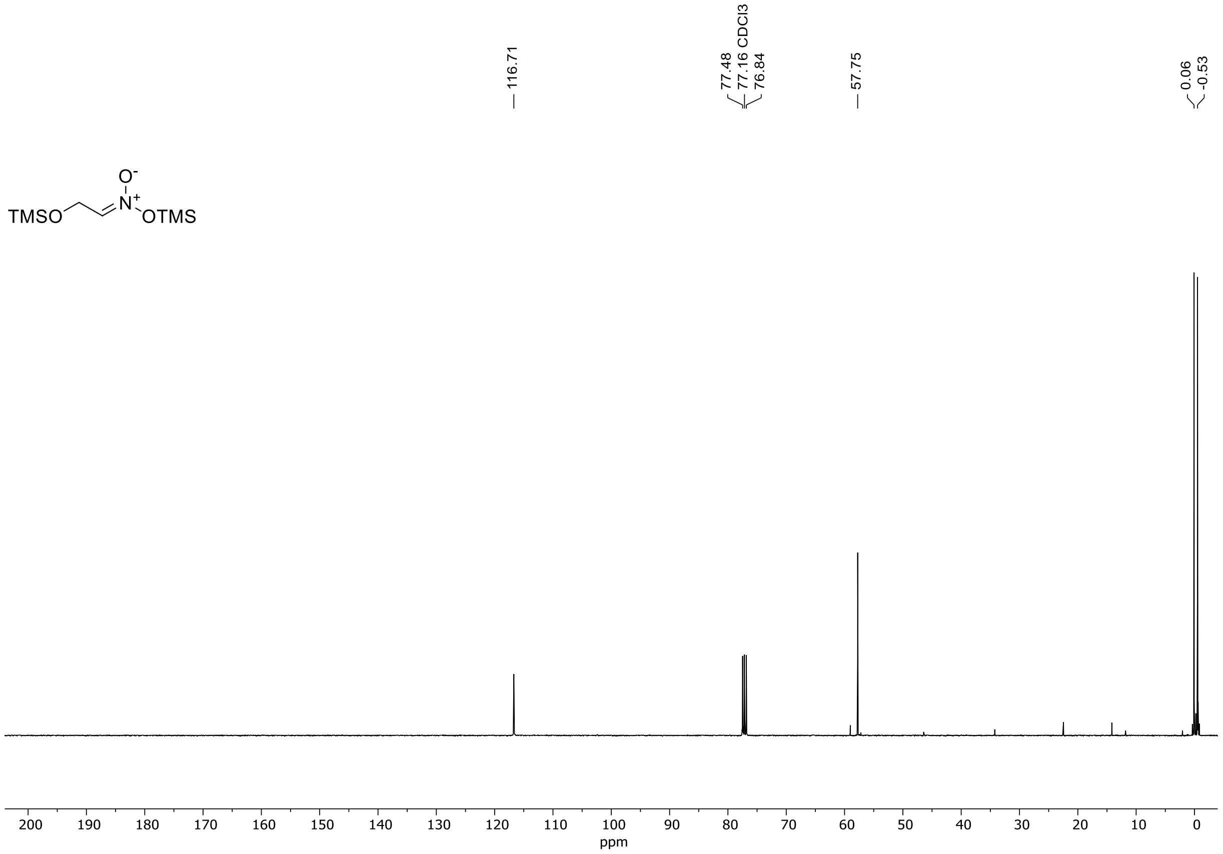

Figure S21. ^{1}H-NMR (400 MHz, $CDCl_3$, 298 K) spectrum of **2e**.

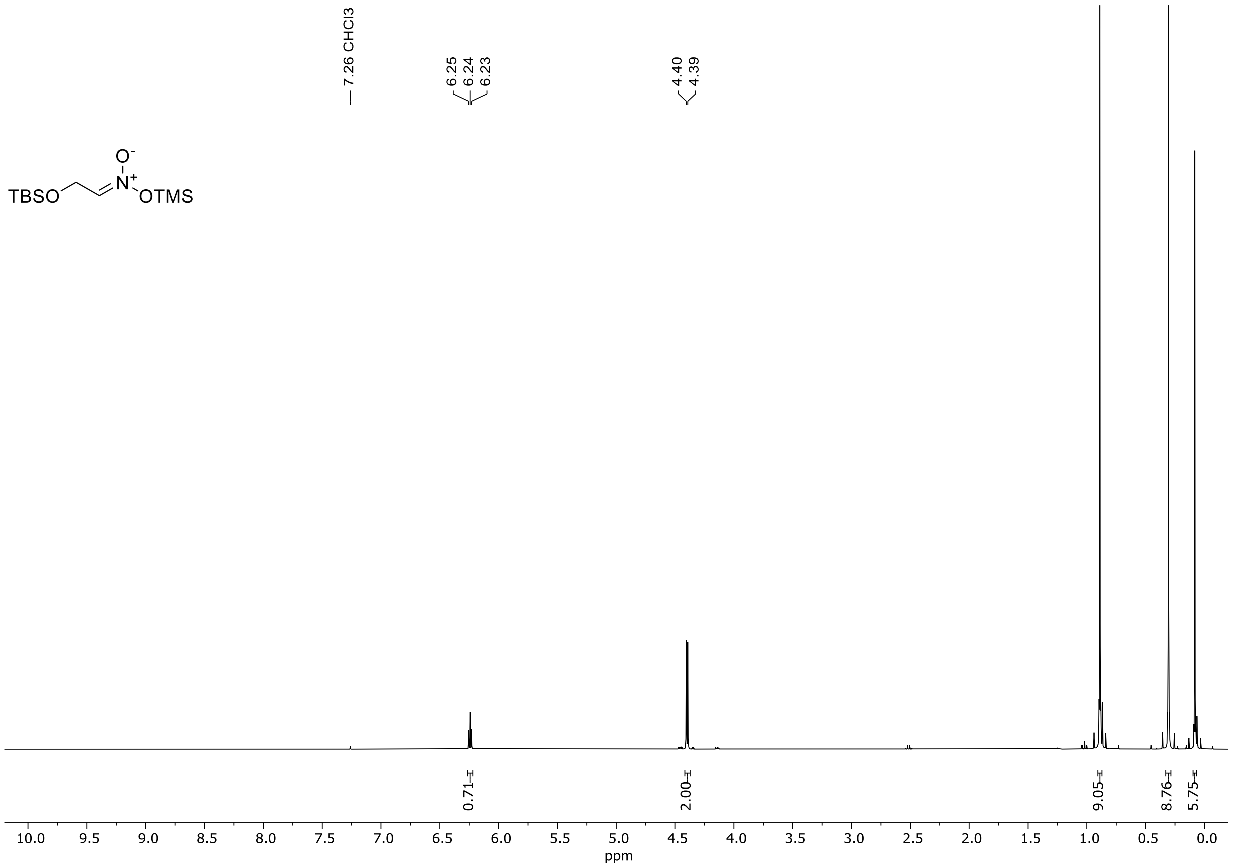

Figure S22. ^{13}C-NMR (101 MHz, $CDCl_3$, 298 K) spectrum of **2e**.

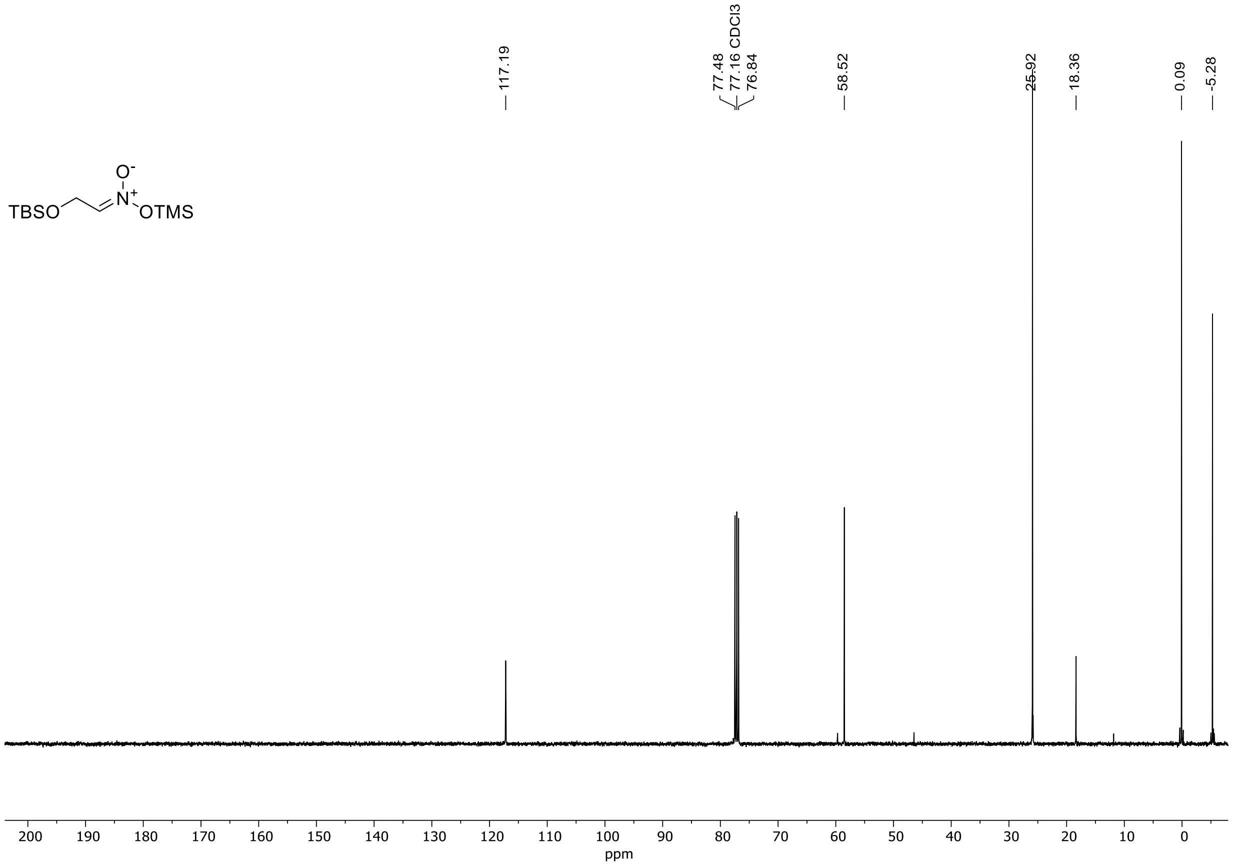

Figure S23. ^{1}H-NMR (400 MHz, $CDCl_3$, 298 K) spectrum of **2f**.

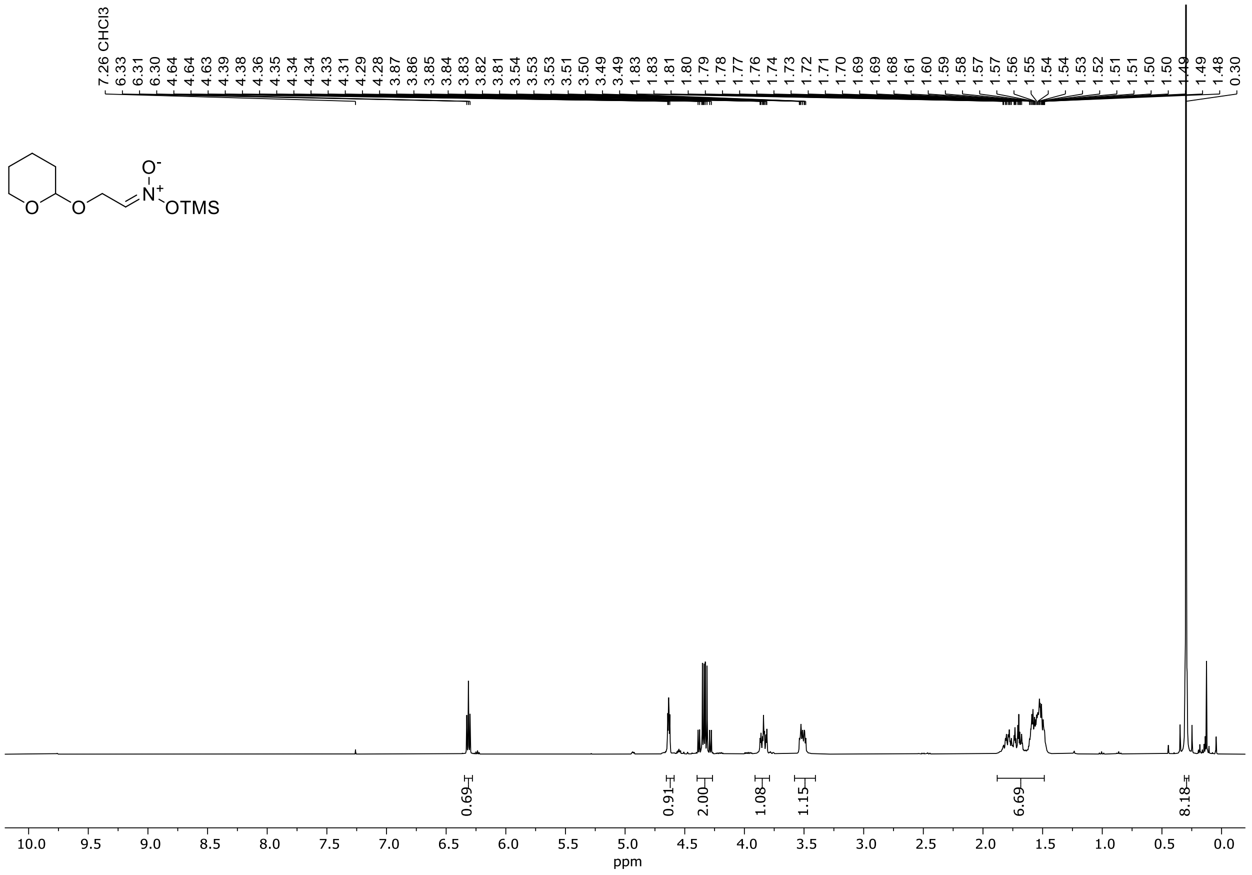

Figure S24. ^{13}C-NMR (101 MHz, $CDCl_3$, 298 K) spectrum of **2f**.

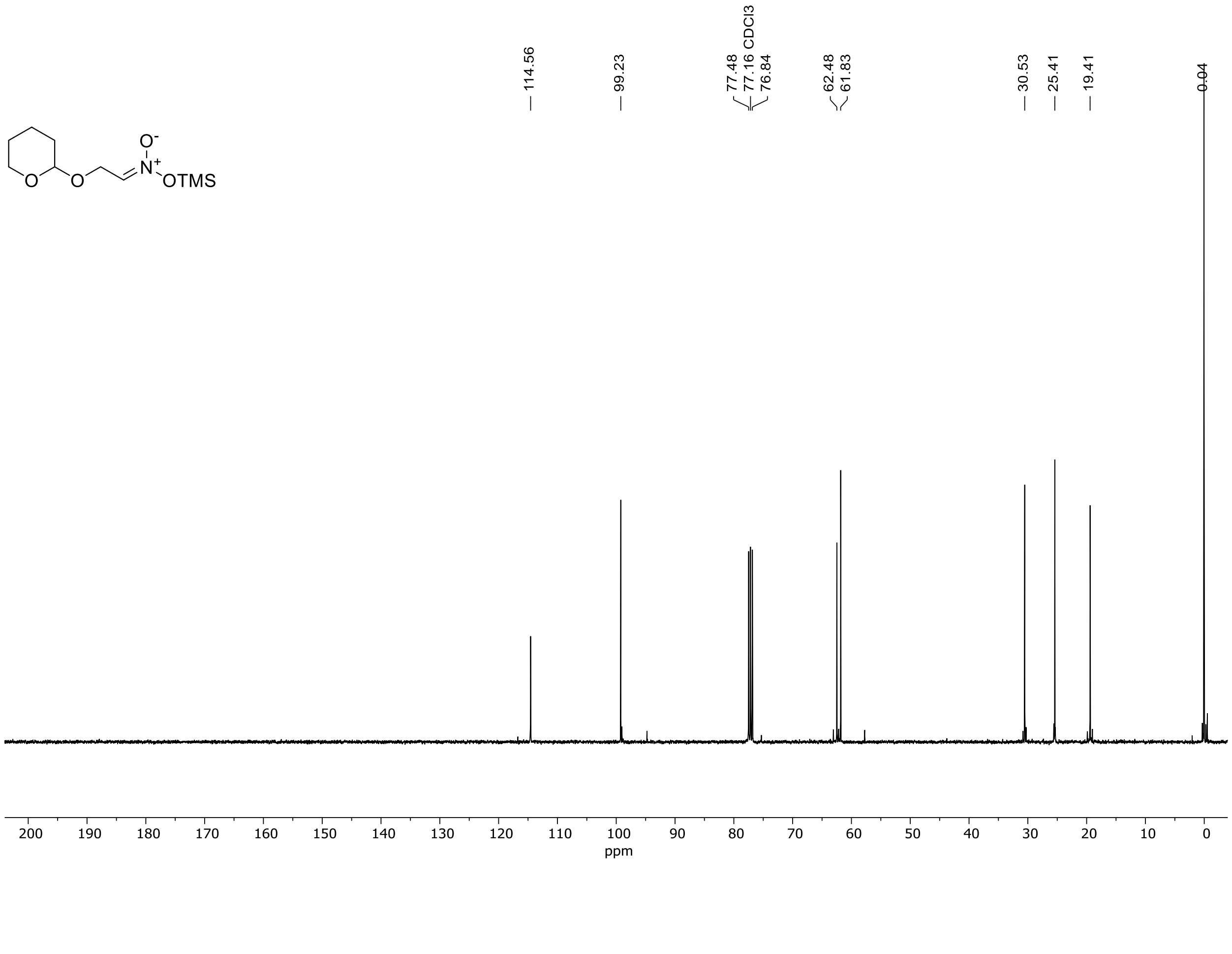

Figure S25. ^{1}H-NMR (400 MHz, $CDCl_3$, 298 K) spectrum of **2g**.

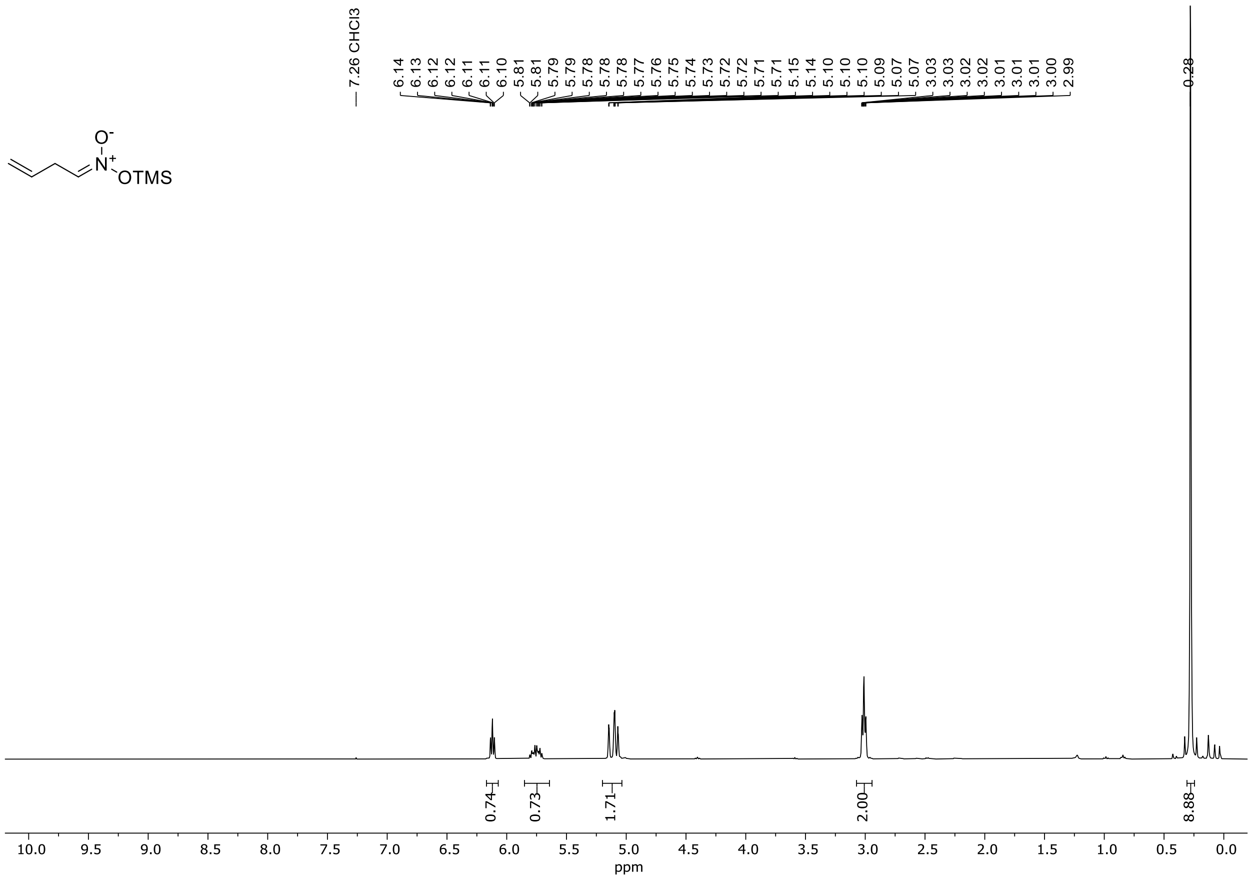

Figure S26. ^{13}C-NMR (101 MHz, $CDCl_3$, 298 K) spectrum of **2g**.

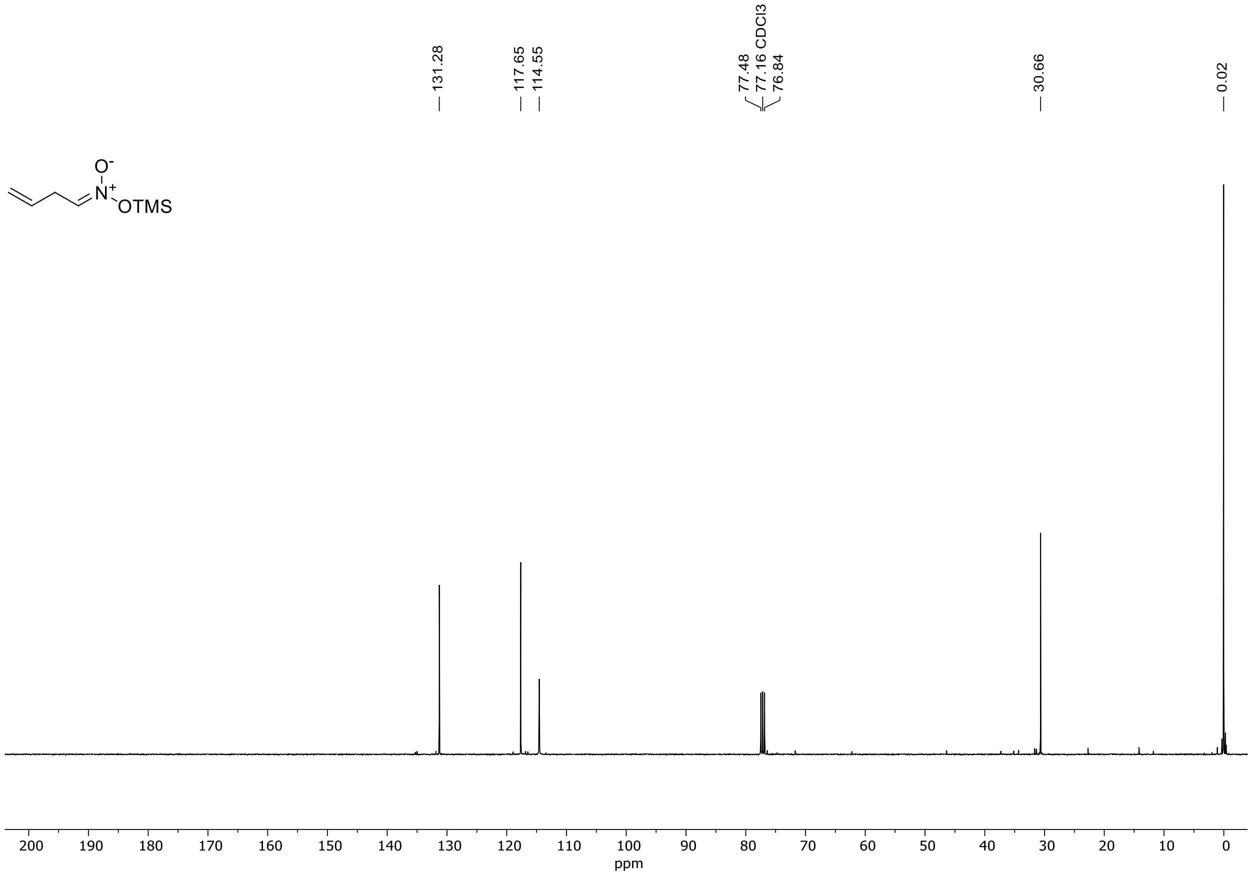

Figure S27. ^{1}H-NMR (400 MHz, $CDCl_3$, 298 K) spectrum of **2h**.

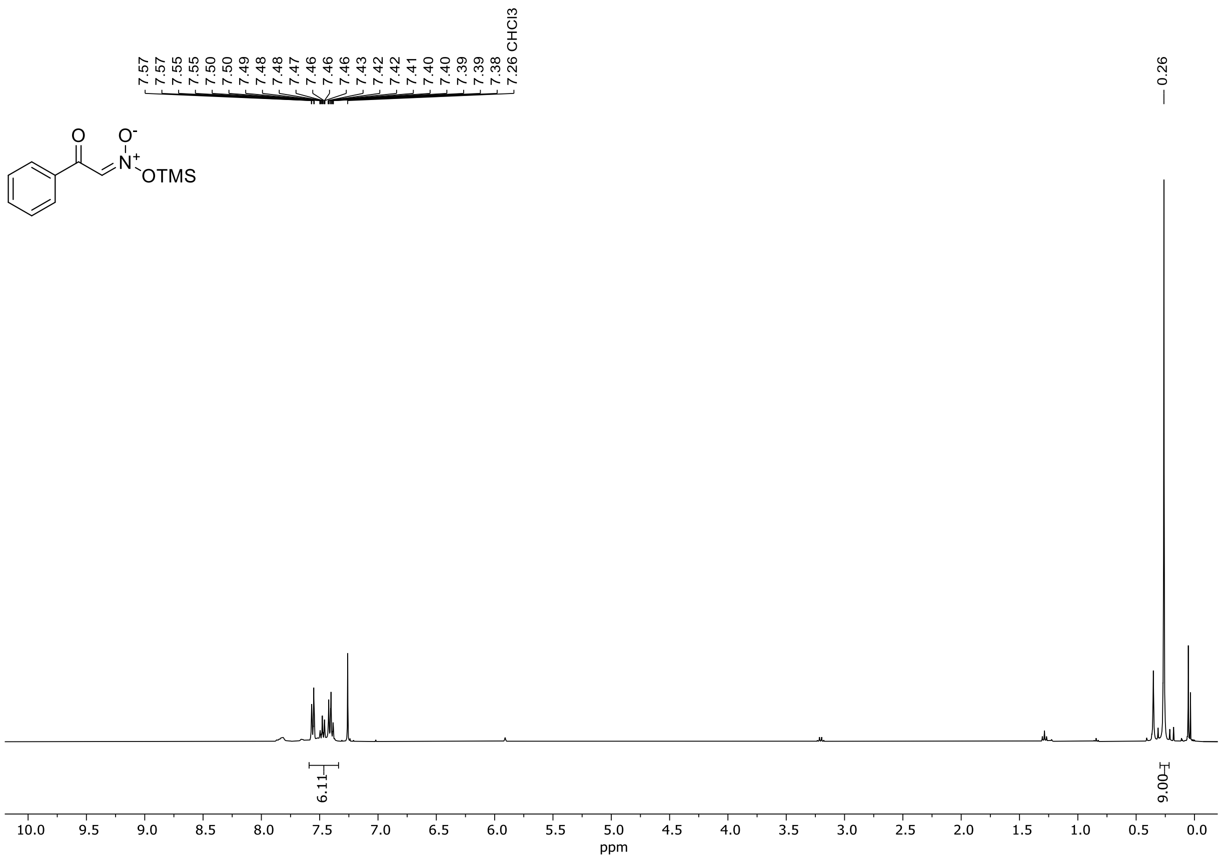

Figure S28. ^{13}C-NMR (101 MHz, $CDCl_3$, 298 K) spectrum of **2h**.

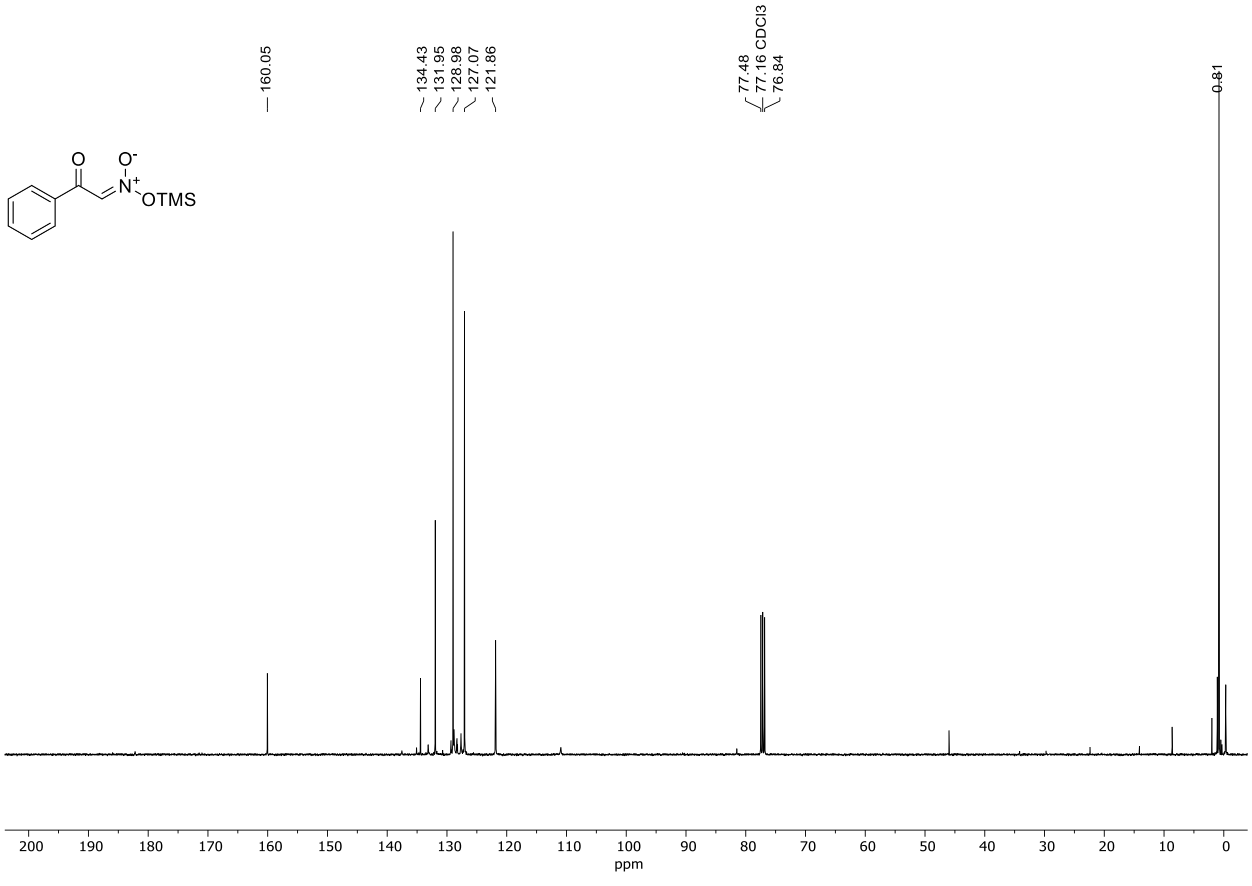

Figure S29. ^{1}H-NMR (400 MHz, $CDCl_3$, 298 K) spectrum of **2i**.

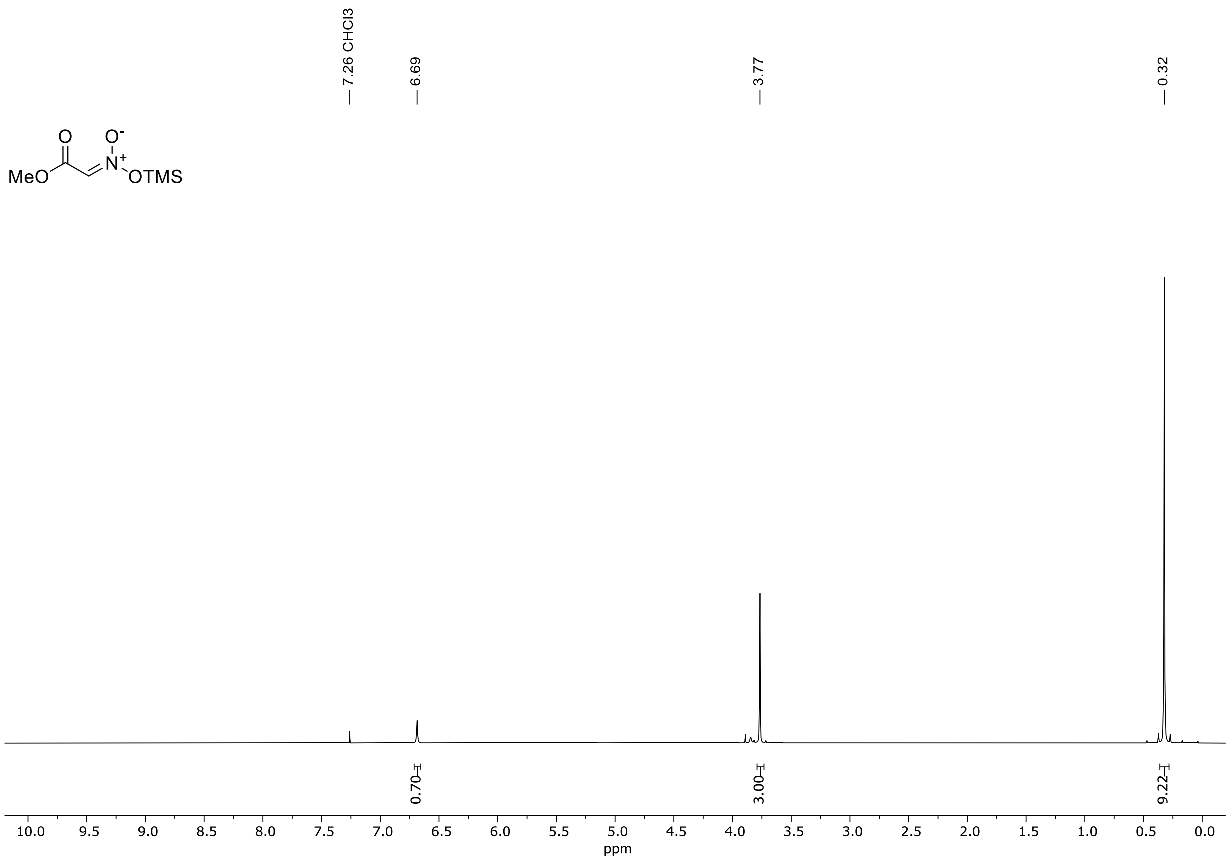

Figure S30. ^{13}C-NMR (101 MHz, $CDCl_3$, 298 K) spectrum of **2i**.

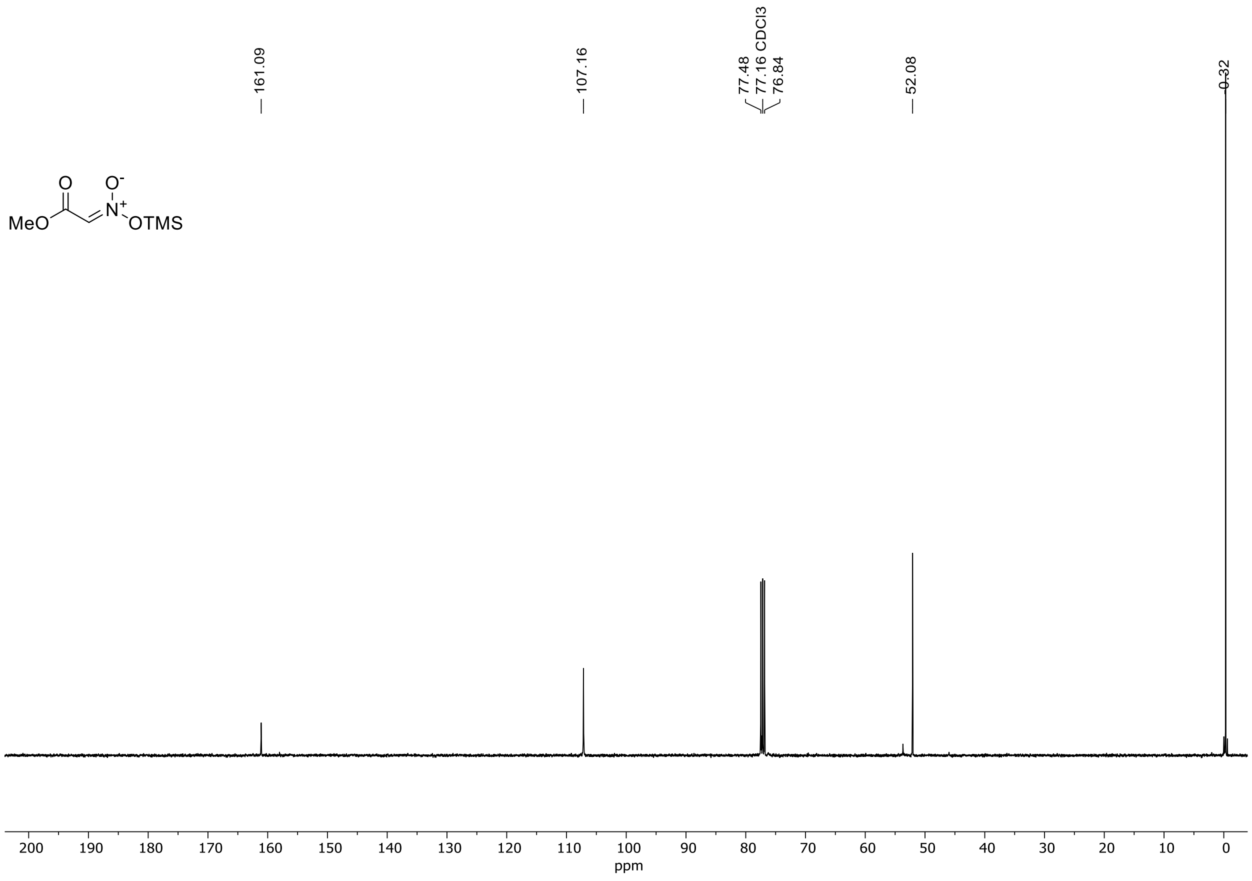

Figure S31. ^{1}H-NMR (400 MHz, $CDCl_3$, 298 K) spectrum of **2j**.

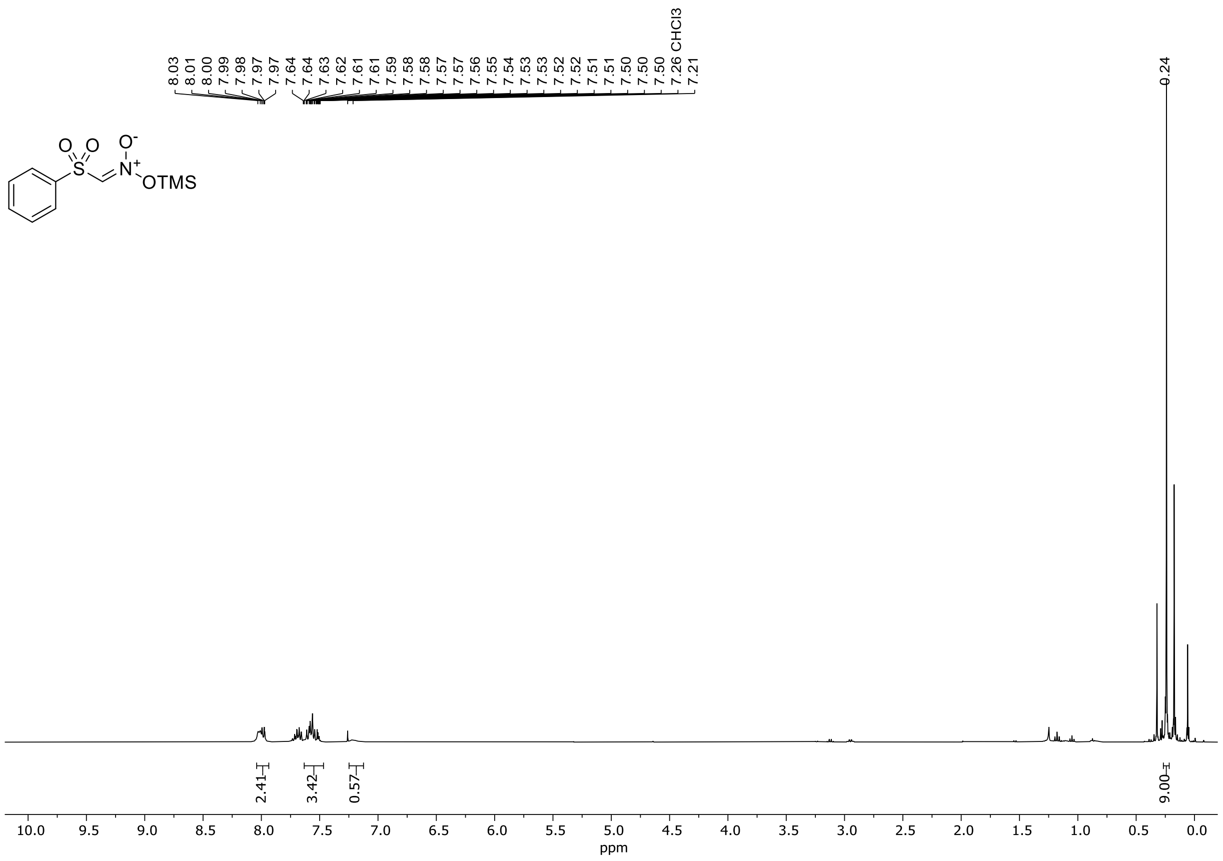

Figure S32. ^{13}C-NMR (101 MHz, $CDCl_3$, 298 K) spectrum of **2j**.

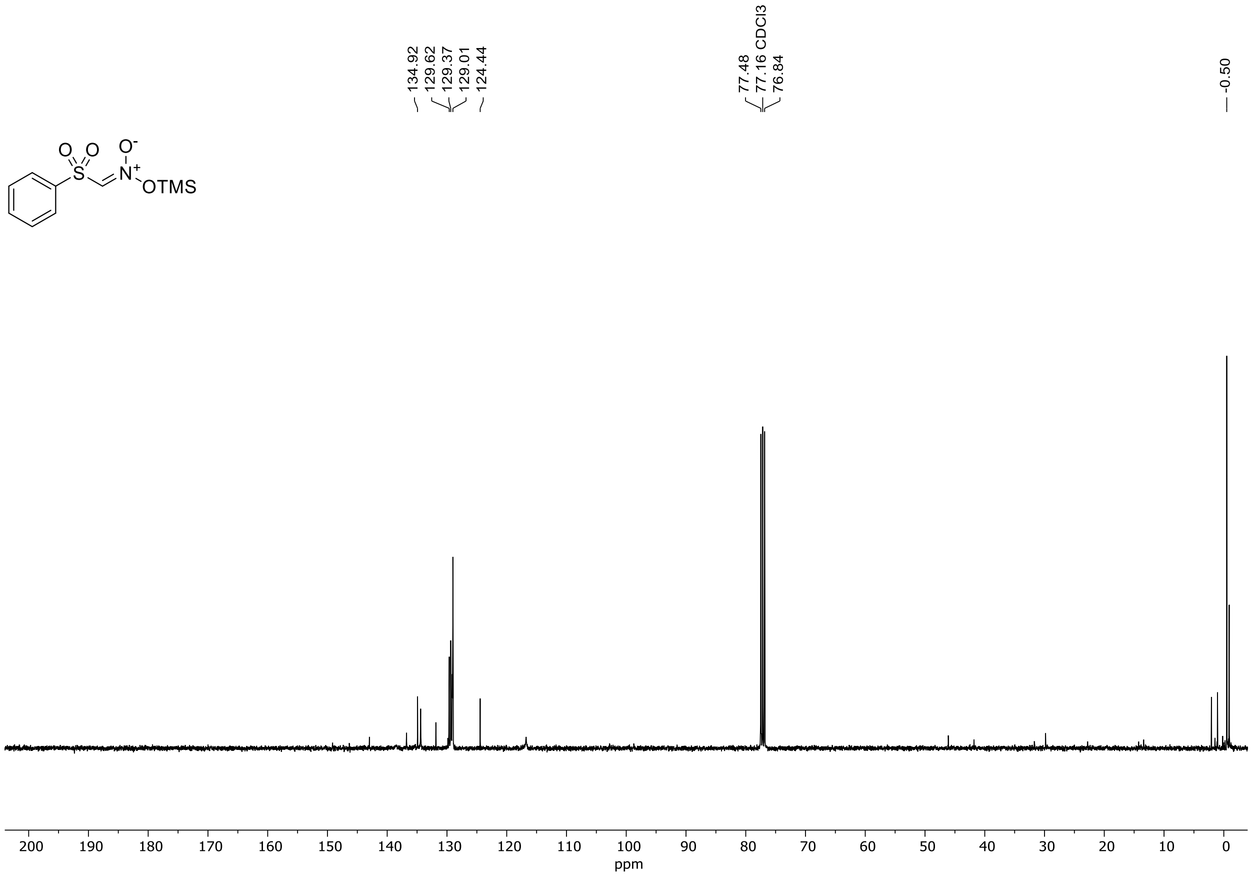

Figure S33. ^{1}H-NMR (400 MHz, $CDCl_3$, 298 K) spectrum of **2k**.

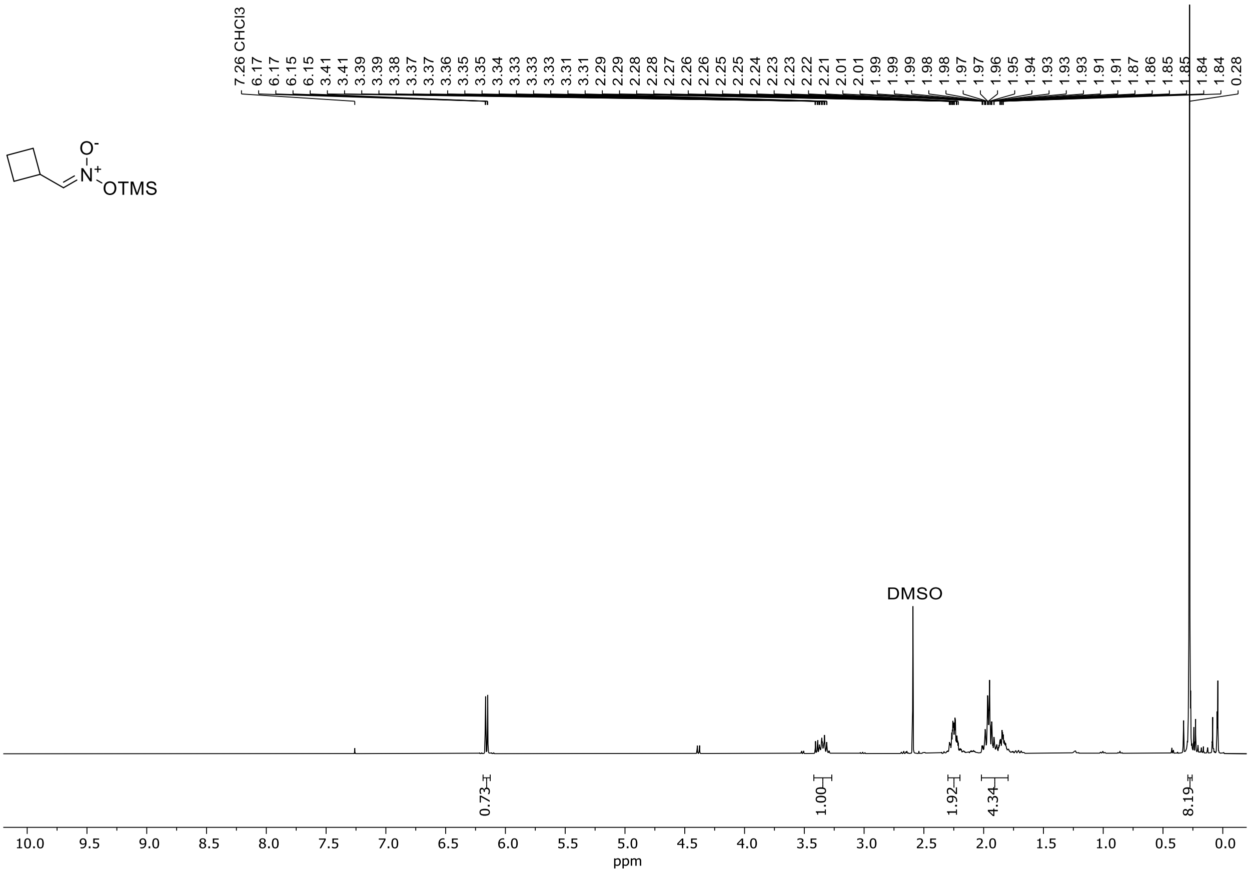

Figure S34. ^{13}C-NMR (101 MHz, $CDCl_3$, 298 K) spectrum of **2k**.

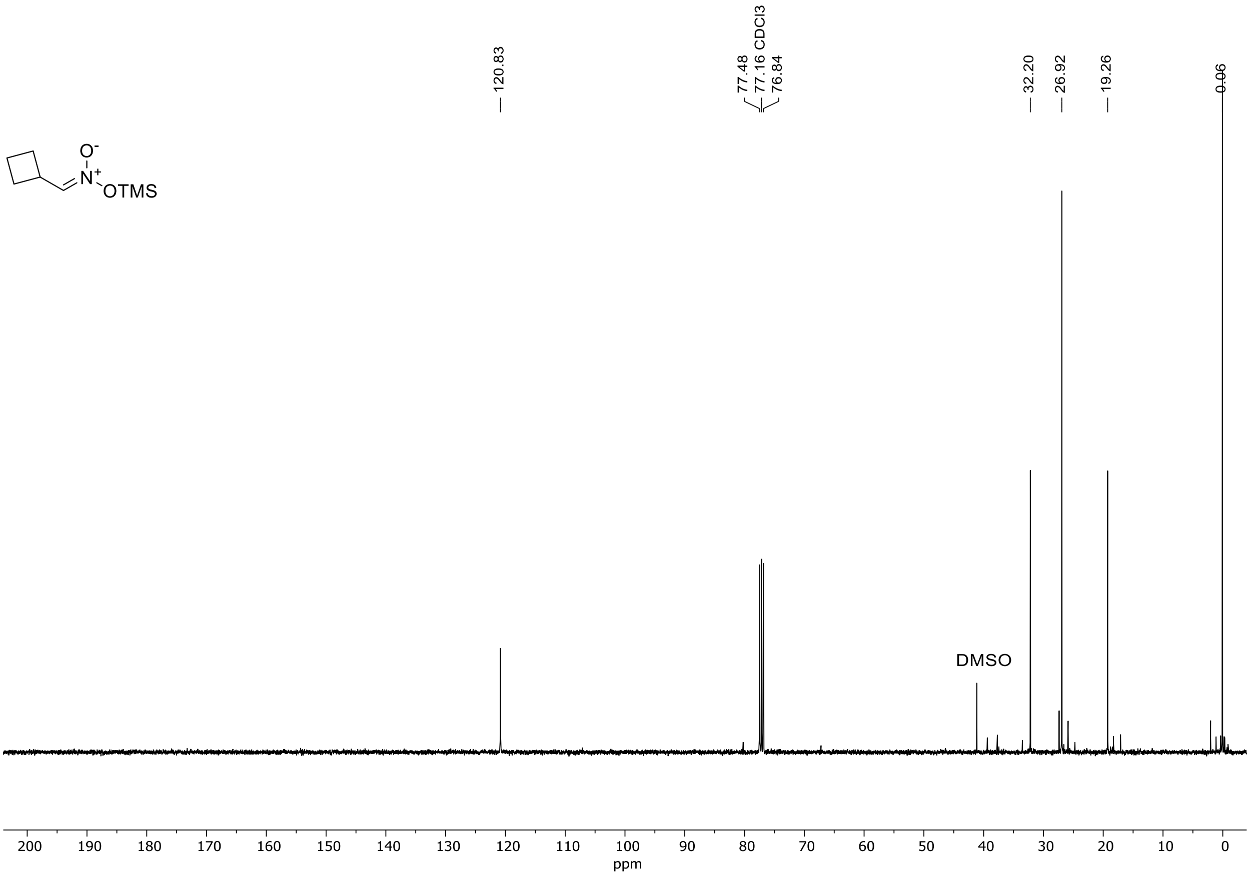

Figure S35. ^{1}H-NMR (400 MHz, $CDCl_3$, 298 K) spectrum of **2l**.

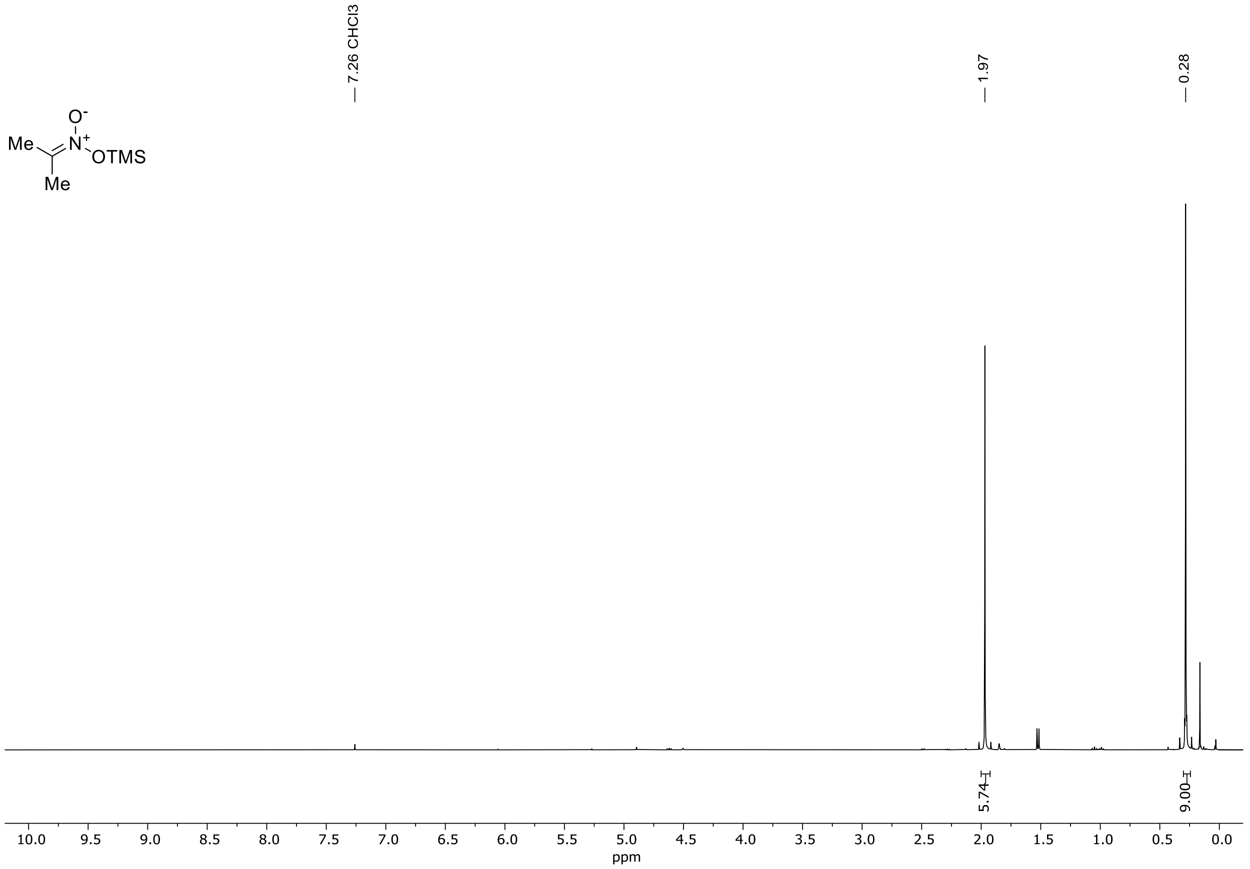

Figure S36. ^{13}C-NMR (101 MHz, $CDCl_3$, 298 K) spectrum of **2l**.

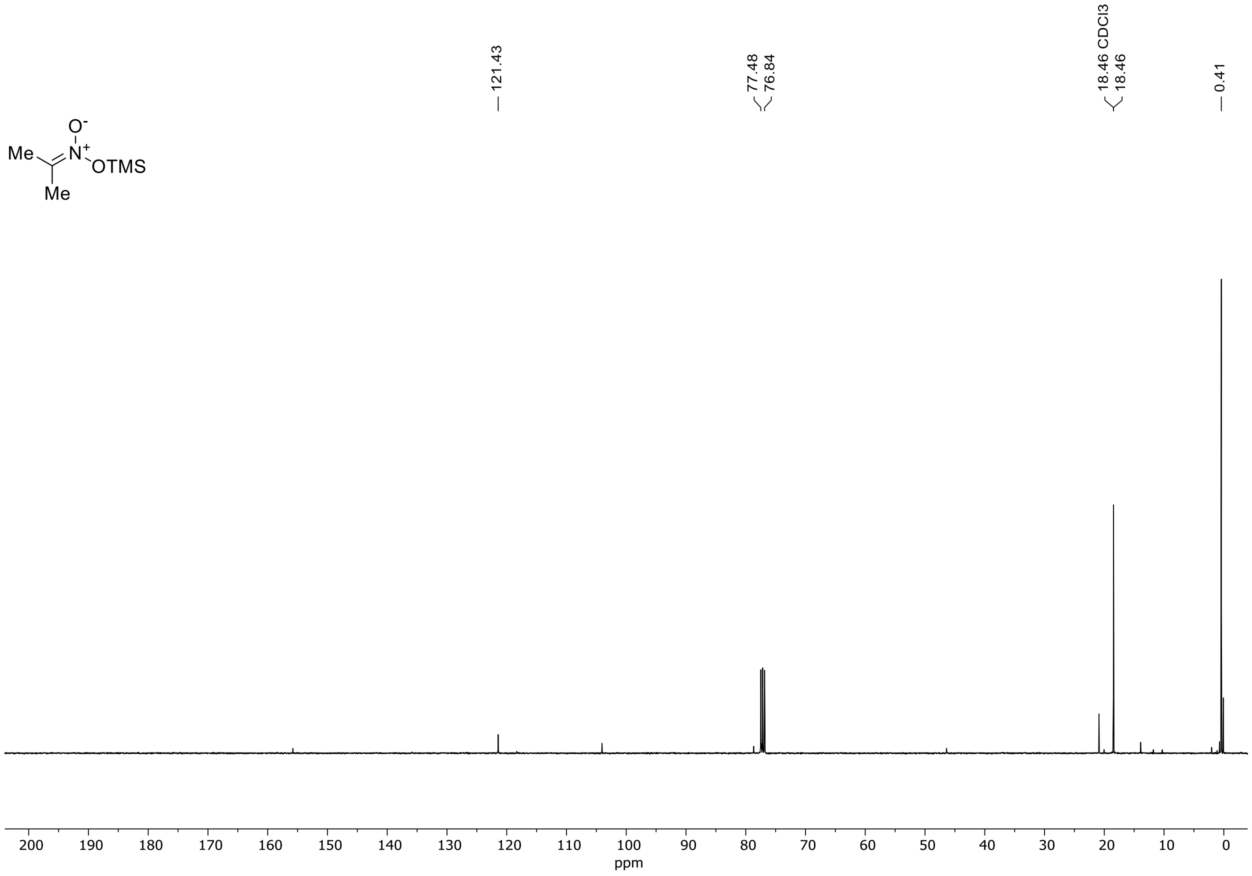

Figure S37. ^{1}H-NMR (400 MHz, $CDCl_3$, 298 K) spectrum of **2m**.

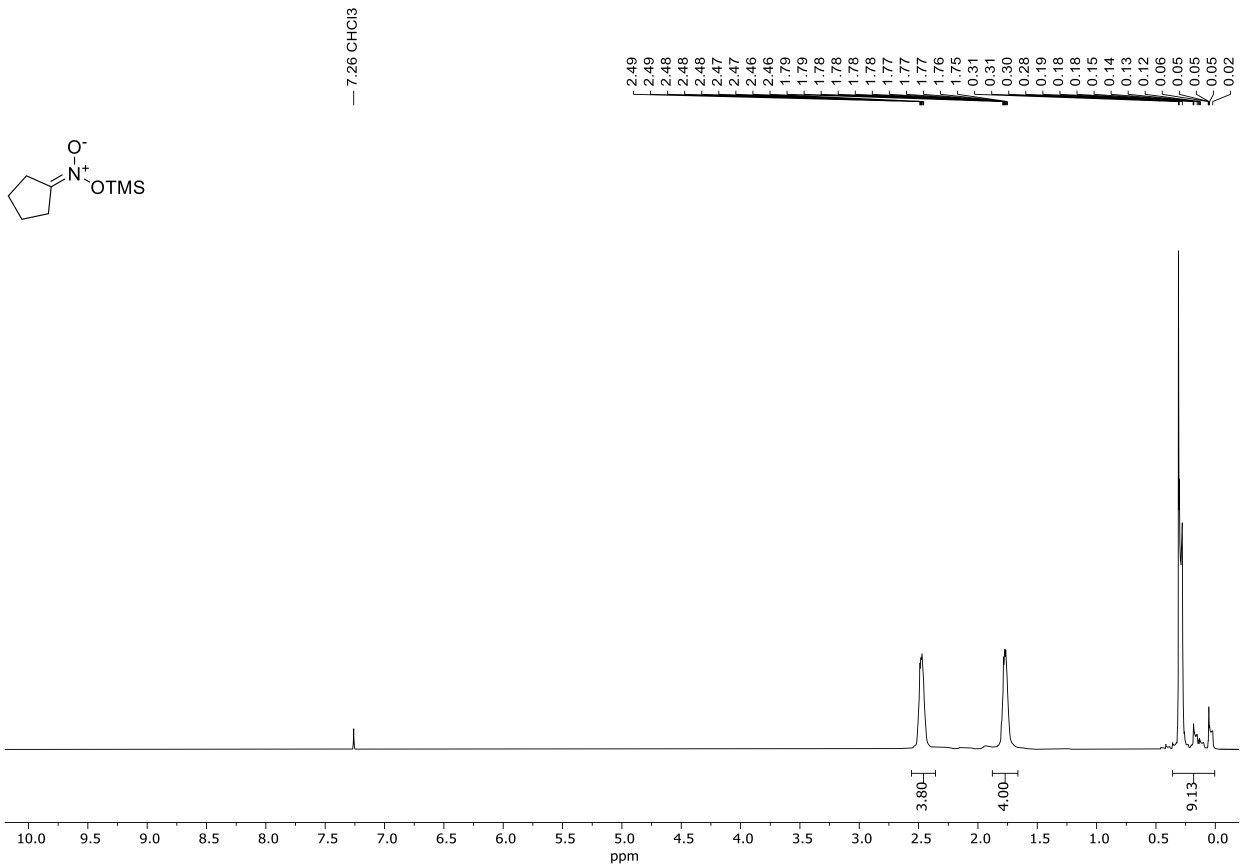

Figure S38. ^{13}C-NMR (101 MHz, $CDCl_3$, 298 K) spectrum of **2m**.

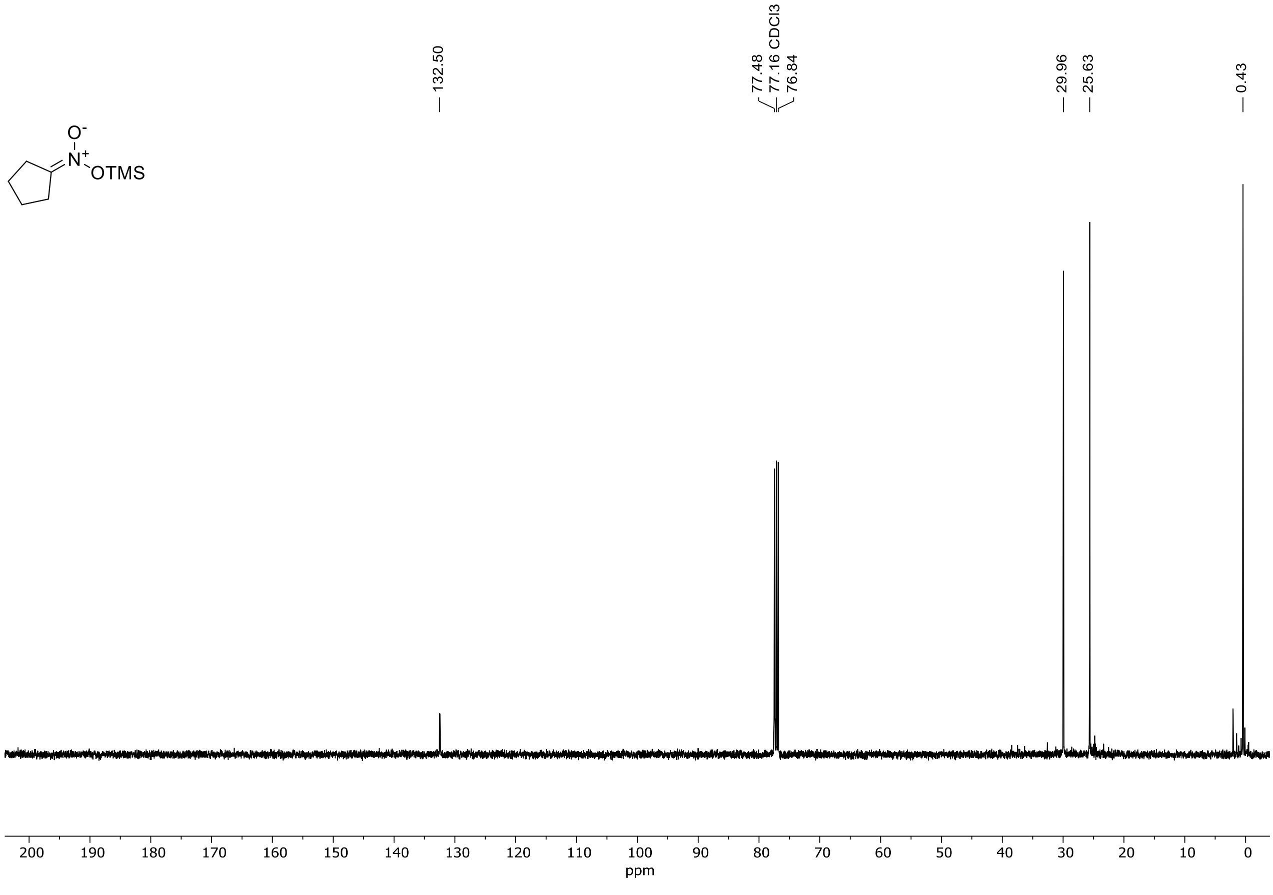

Figure S39. ^{1}H-NMR (400 MHz, $CDCl_3$, 298 K) spectrum of **3a**.

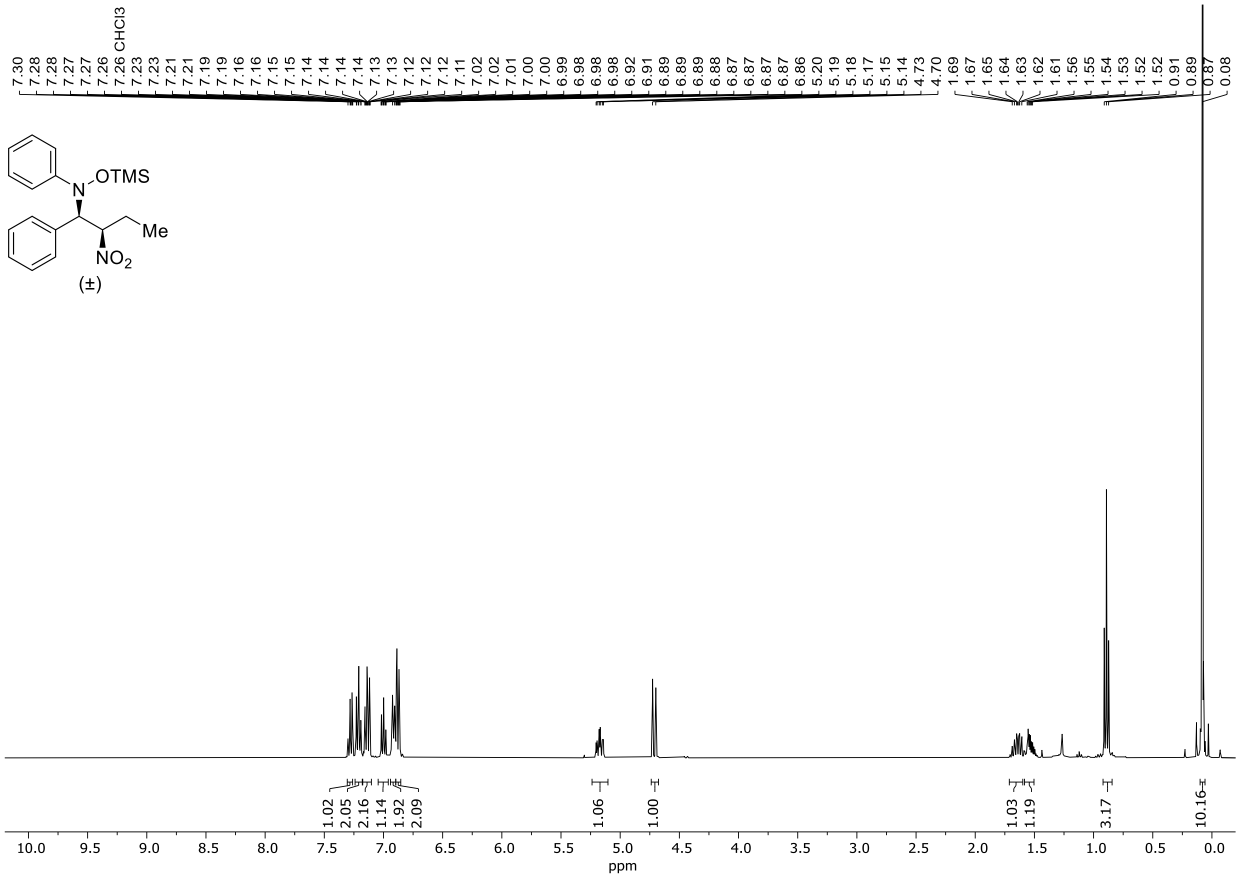

Figure S40. ^{13}C-NMR (101 MHz, $CDCl_3$, 298 K) spectrum of **3a**.

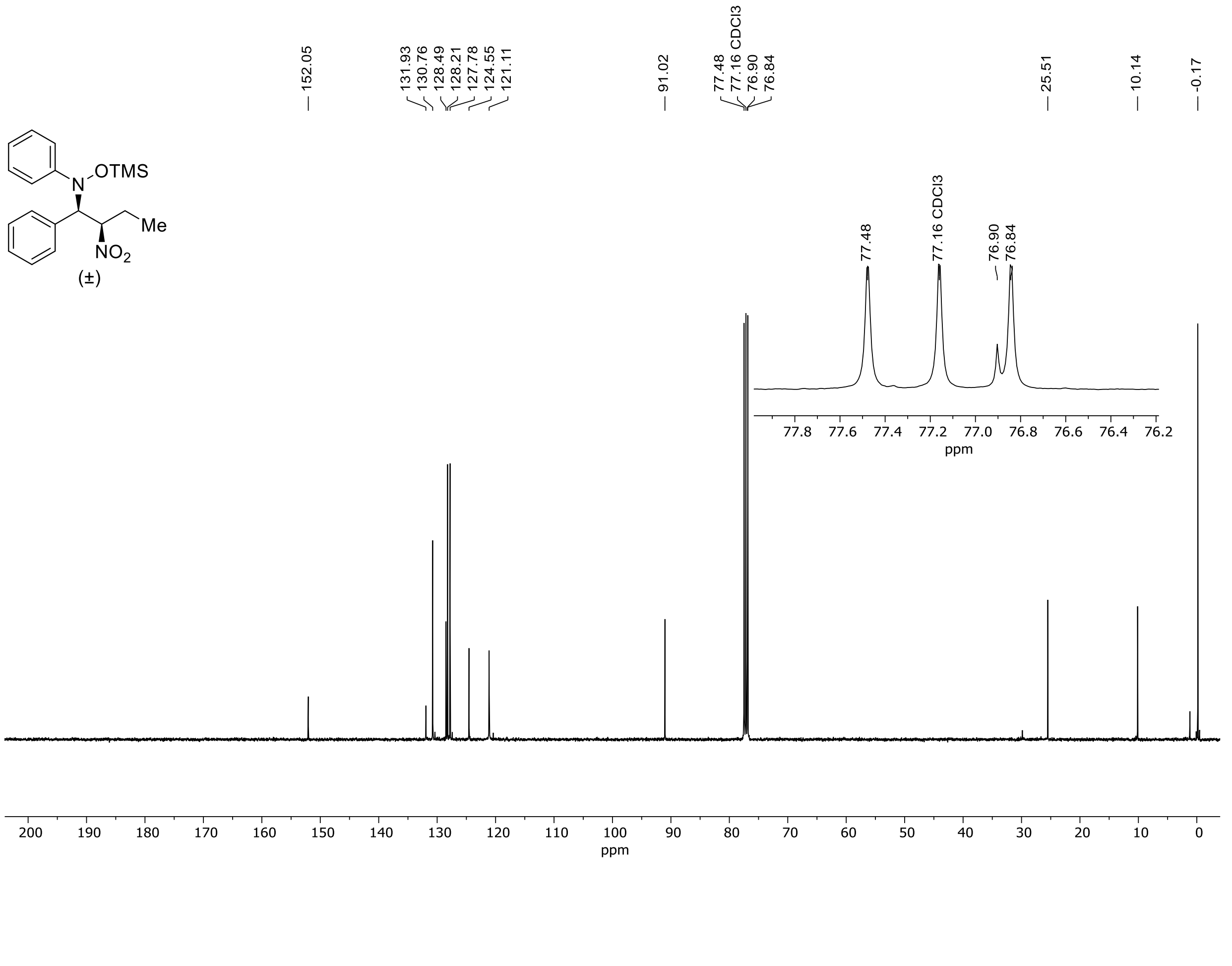

Figure S41. ^{1}H-NMR (400 MHz, CDCl$_3$, 298 K) spectrum of **3a'**.

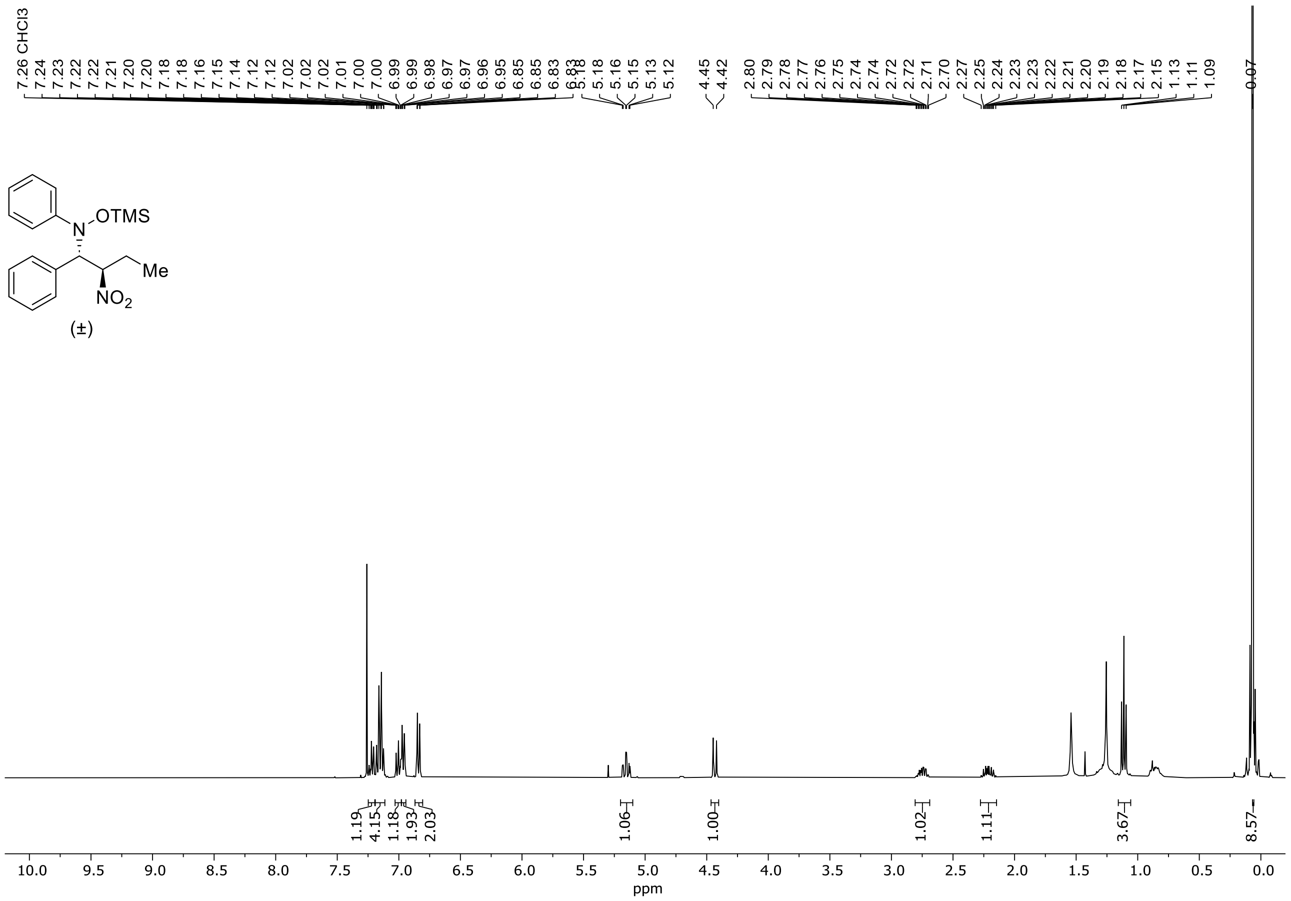

Figure S42. ^{13}C-NMR (101 MHz, CDCl$_3$, 298 K) spectrum of **3a'**.

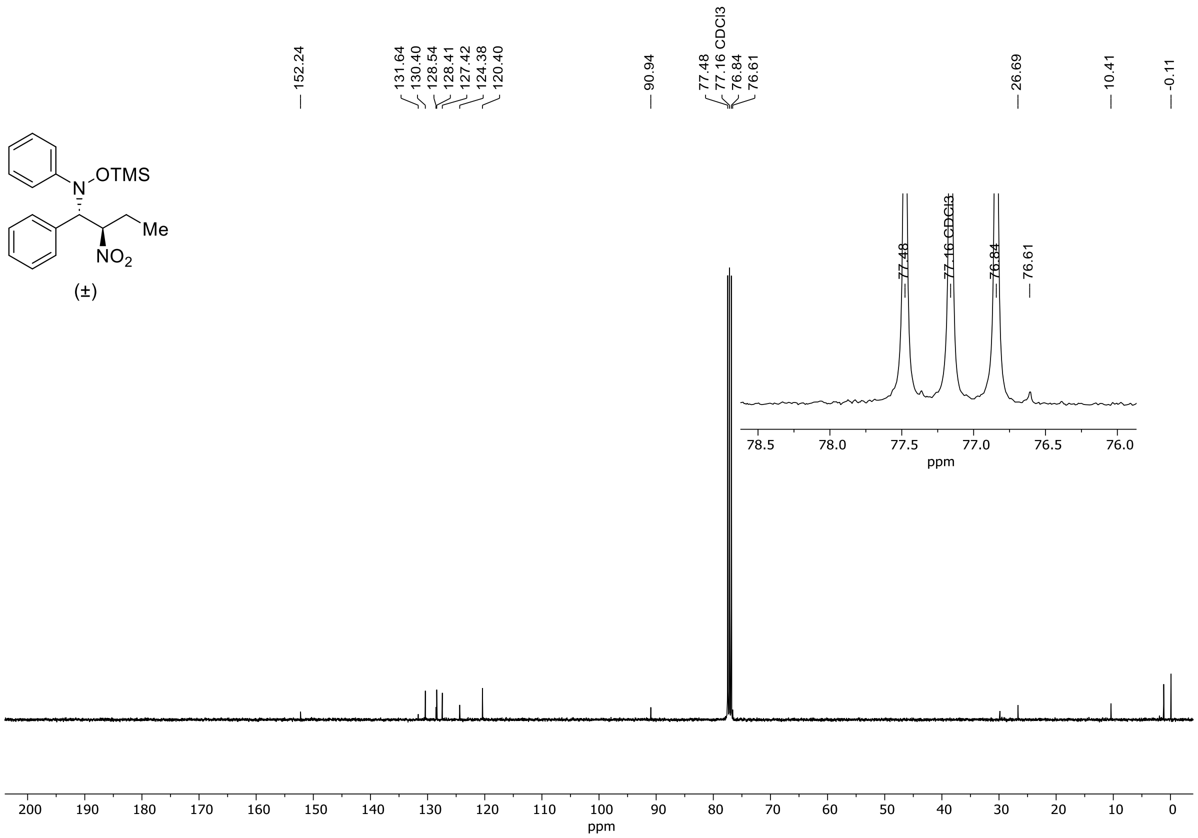

Figure S43. ^{1}H-NMR (400 MHz, $CDCl_3$, 298 K) spectrum of **3b**.

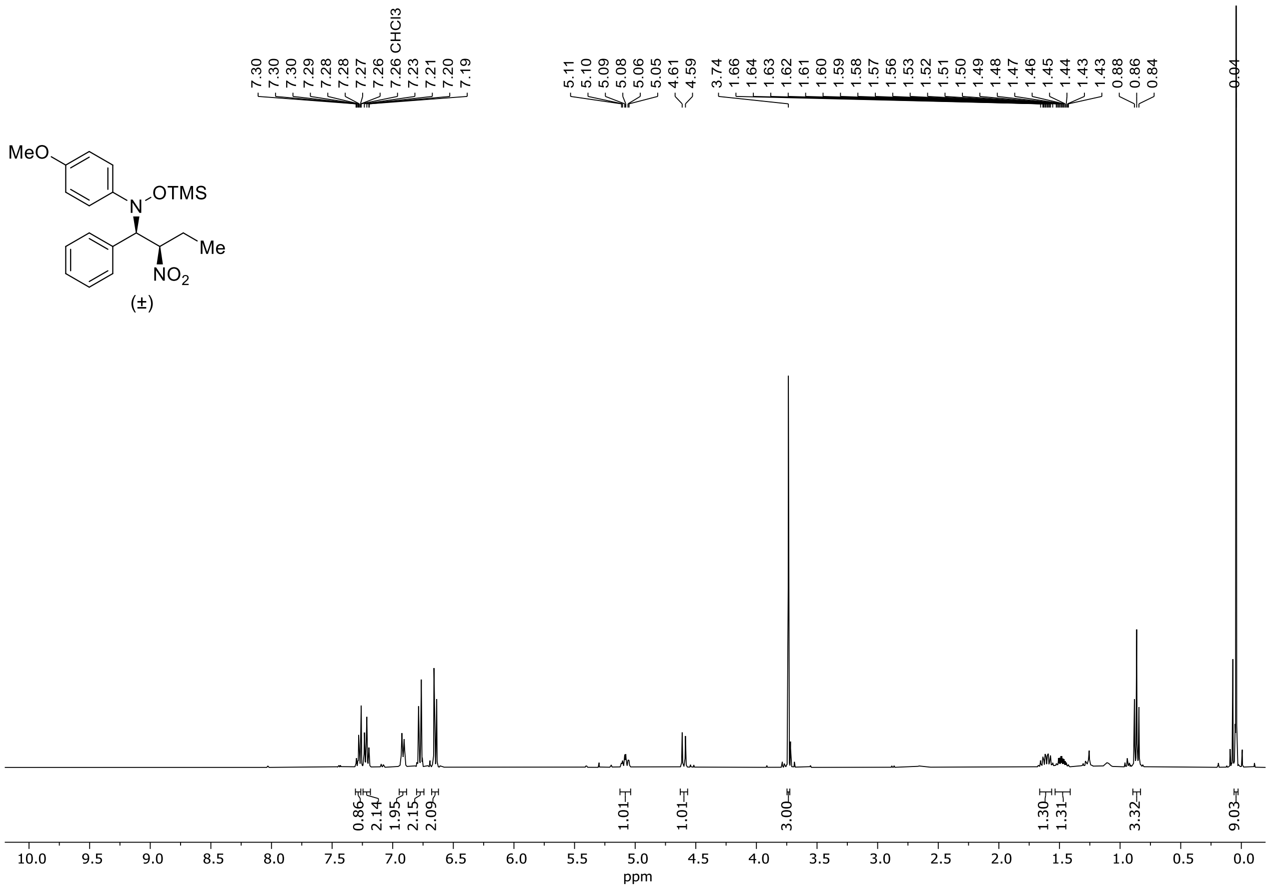

Figure S44. ^{13}C-NMR (101 MHz, $CDCl_3$, 298 K) spectrum of **3b**.

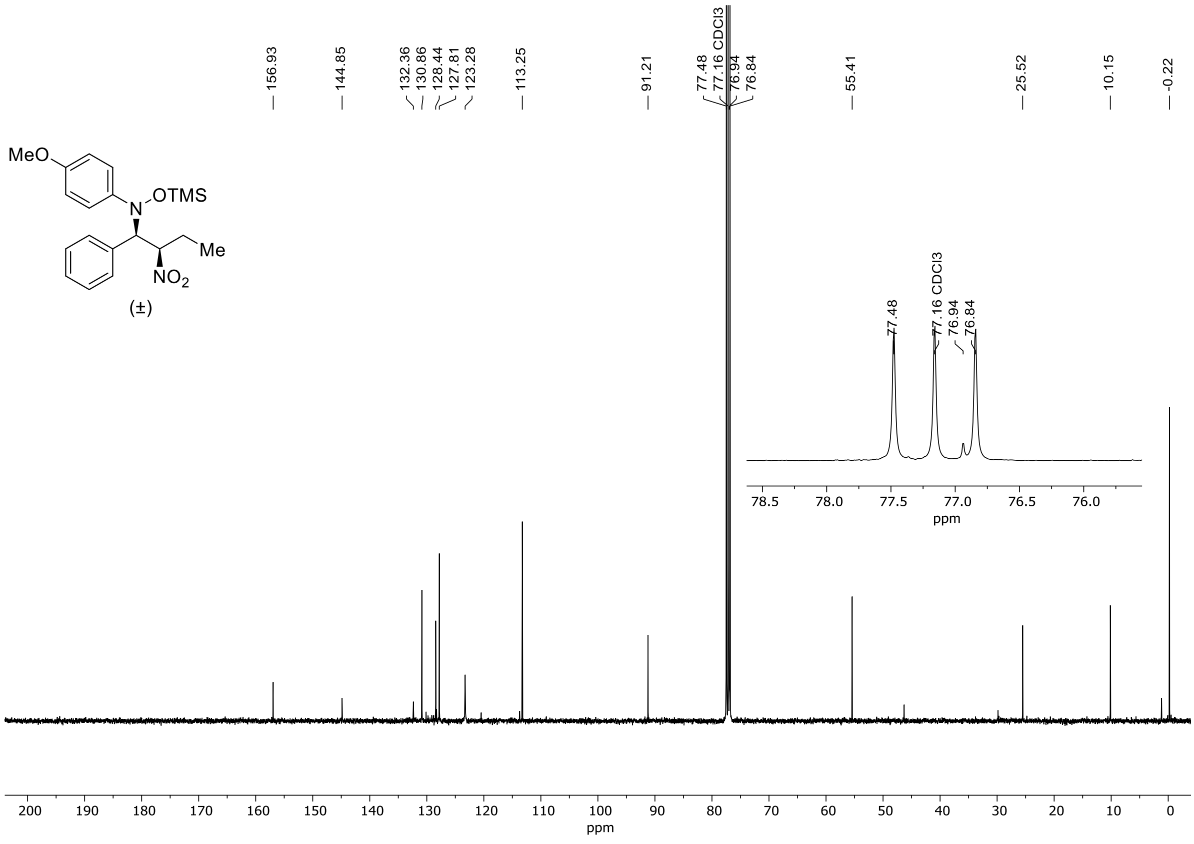

Figure S45. ^{1}H-NMR (400 MHz, $CDCl_3$, 298 K) spectrum of **3c**.

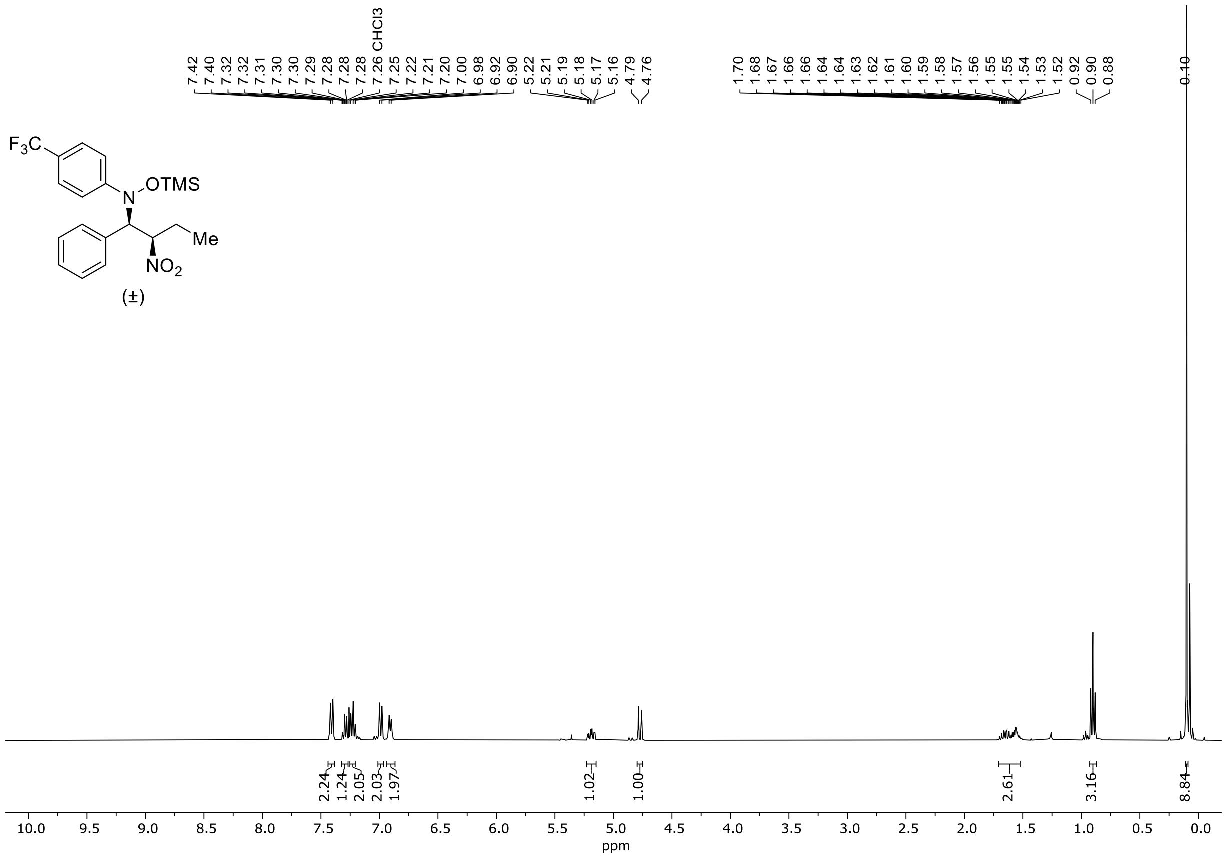

Figure S46. ^{13}C-NMR (101 MHz, $CDCl_3$, 298 K) spectrum of **3c**.

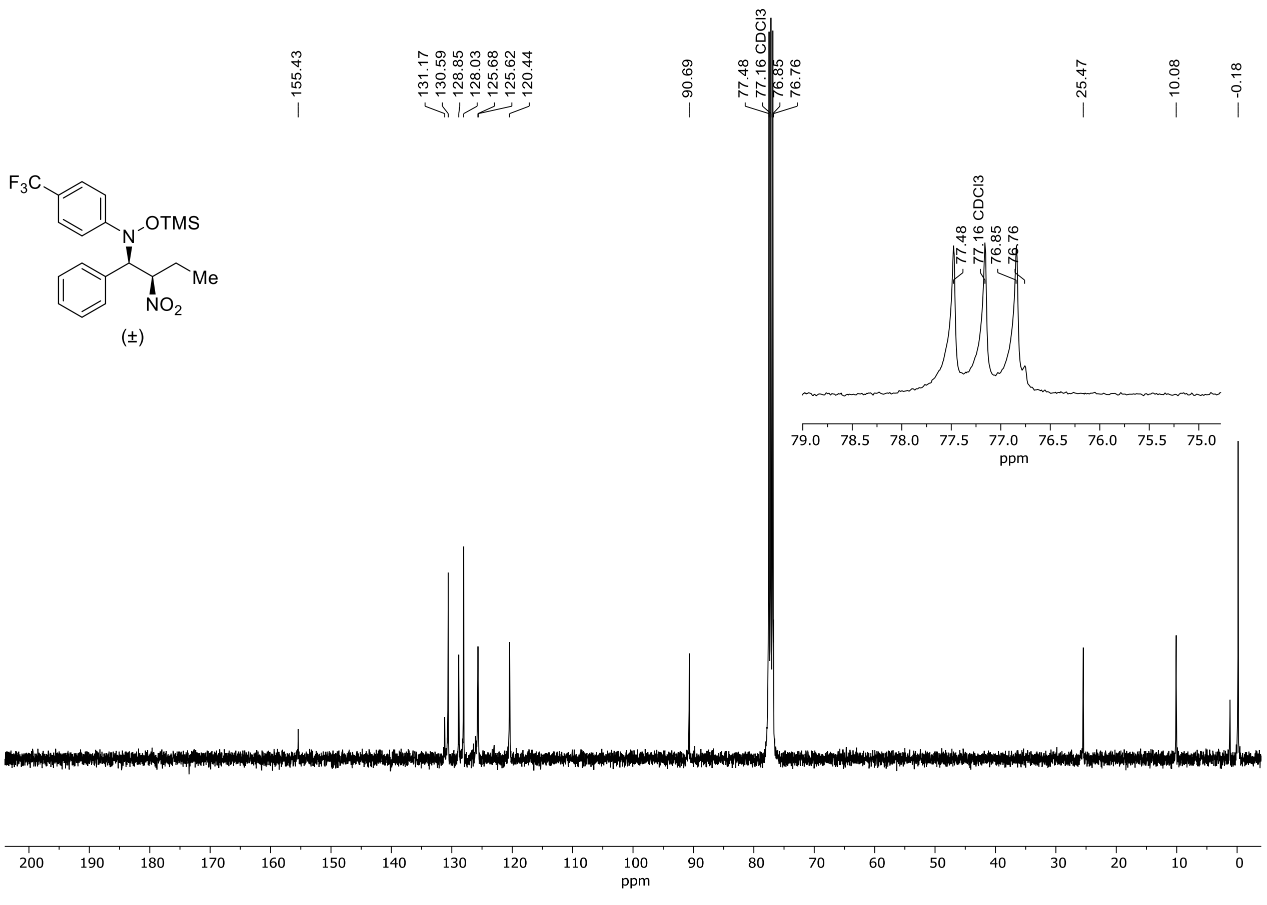

Figure S47. ^{19}F-NMR (376 MHz, $CDCl_3$, 298 K) spectrum of **3c**.

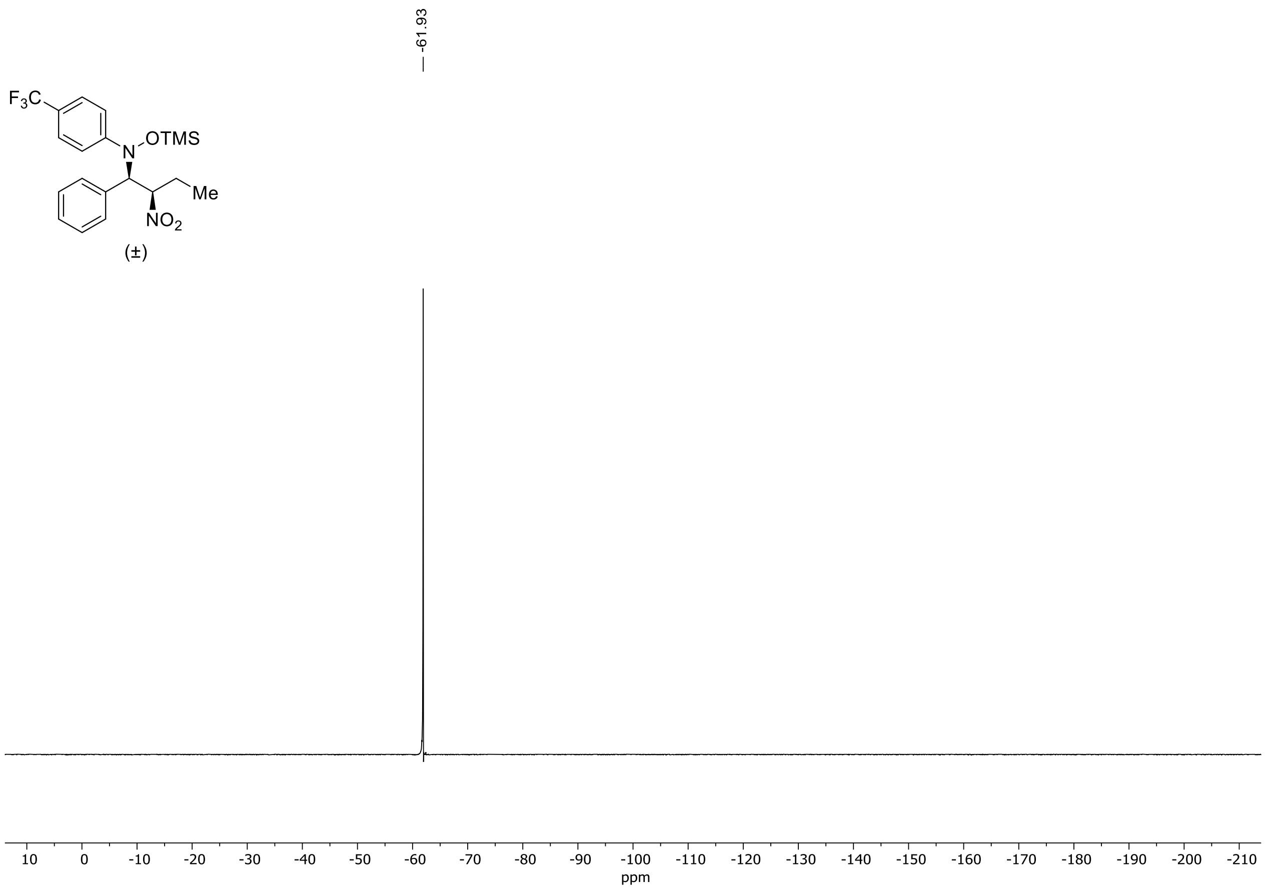

Figure S48. ^{1}H-NMR (400 MHz, $CDCl_3$, 298 K) spectrum of **3d**.

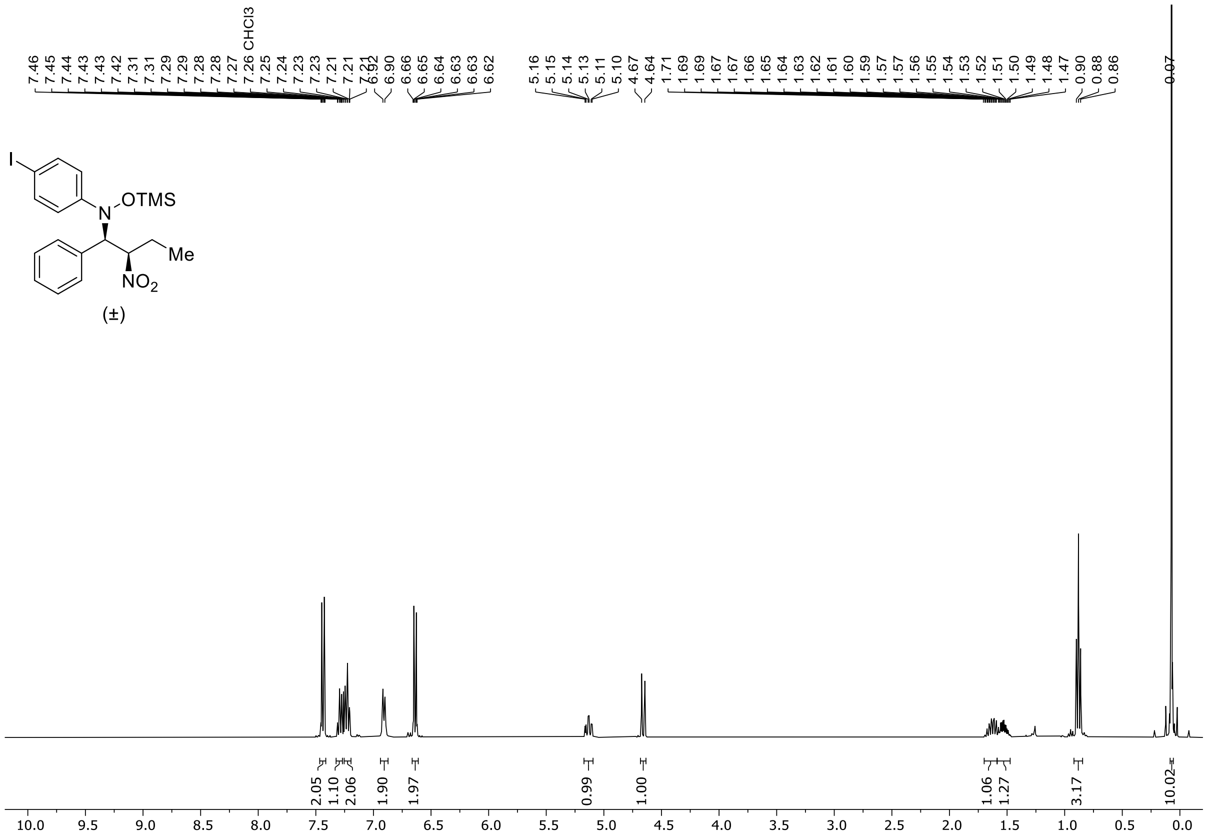

Figure S49. ^{13}C-NMR (101 MHz, $CDCl_3$, 298 K) spectrum of **3d**.

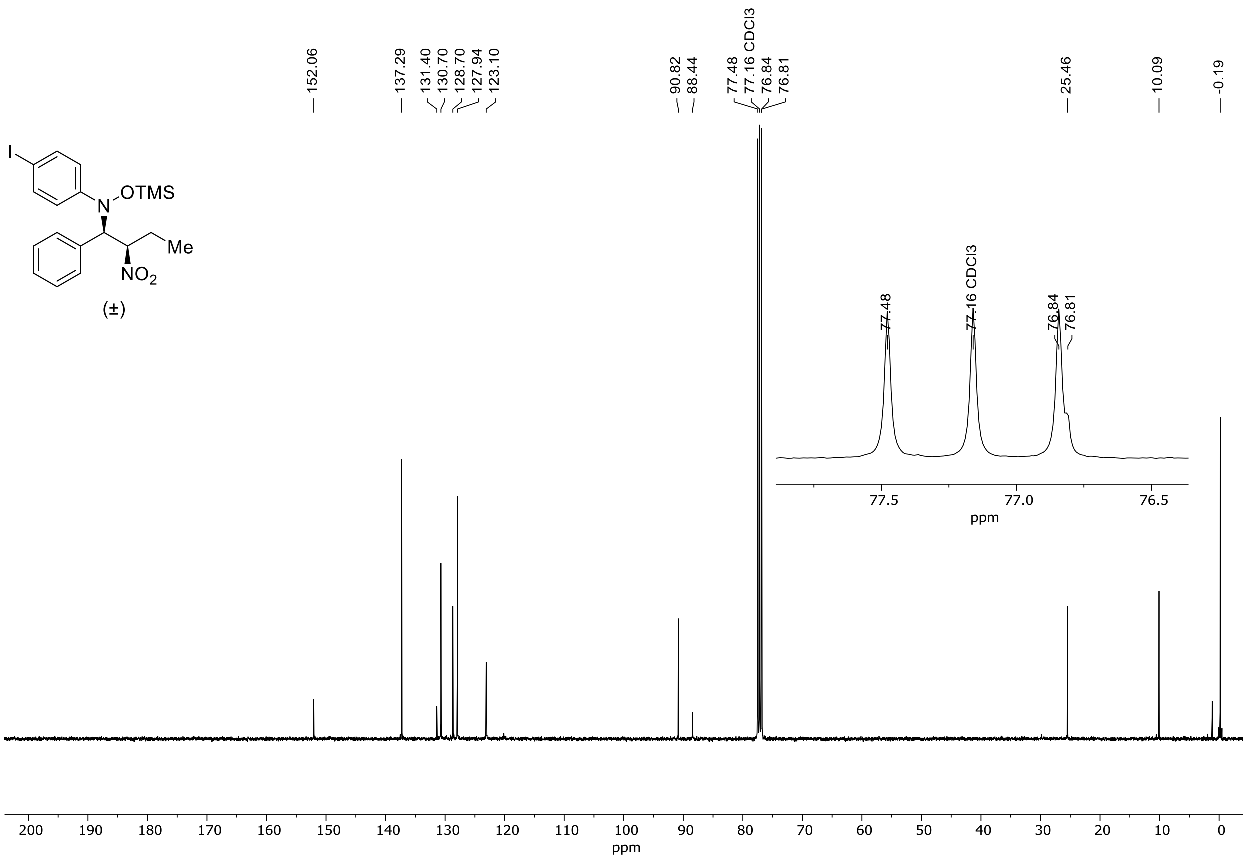

Figure S50. ^{1}H-NMR (400 MHz, $CDCl_3$, 298 K) spectrum of **3d'**.

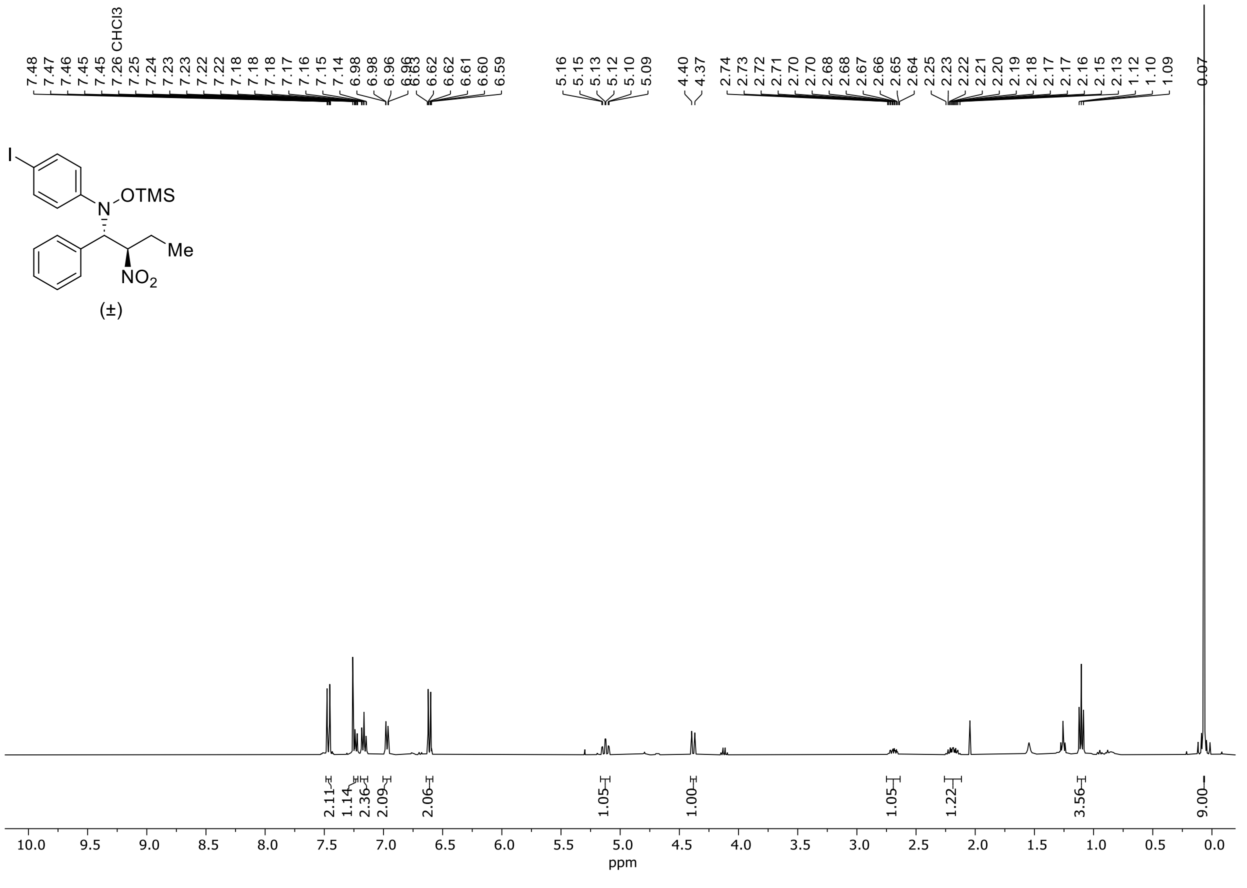

Figure S51. ^{13}C-NMR (101 MHz, $CDCl_3$, 298 K) spectrum of **3d'**.

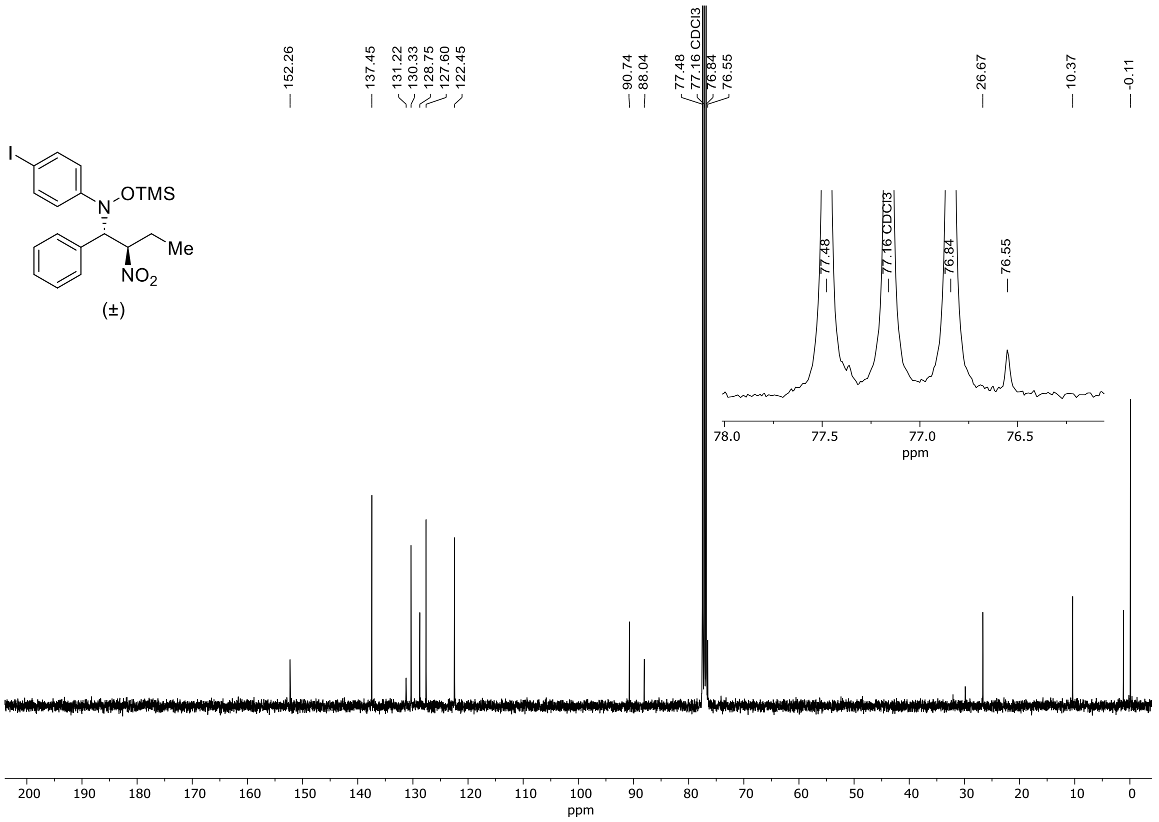

Figure S52. ^{1}H-NMR (400 MHz, $CDCl_3$, 298 K) spectrum of **3e**.

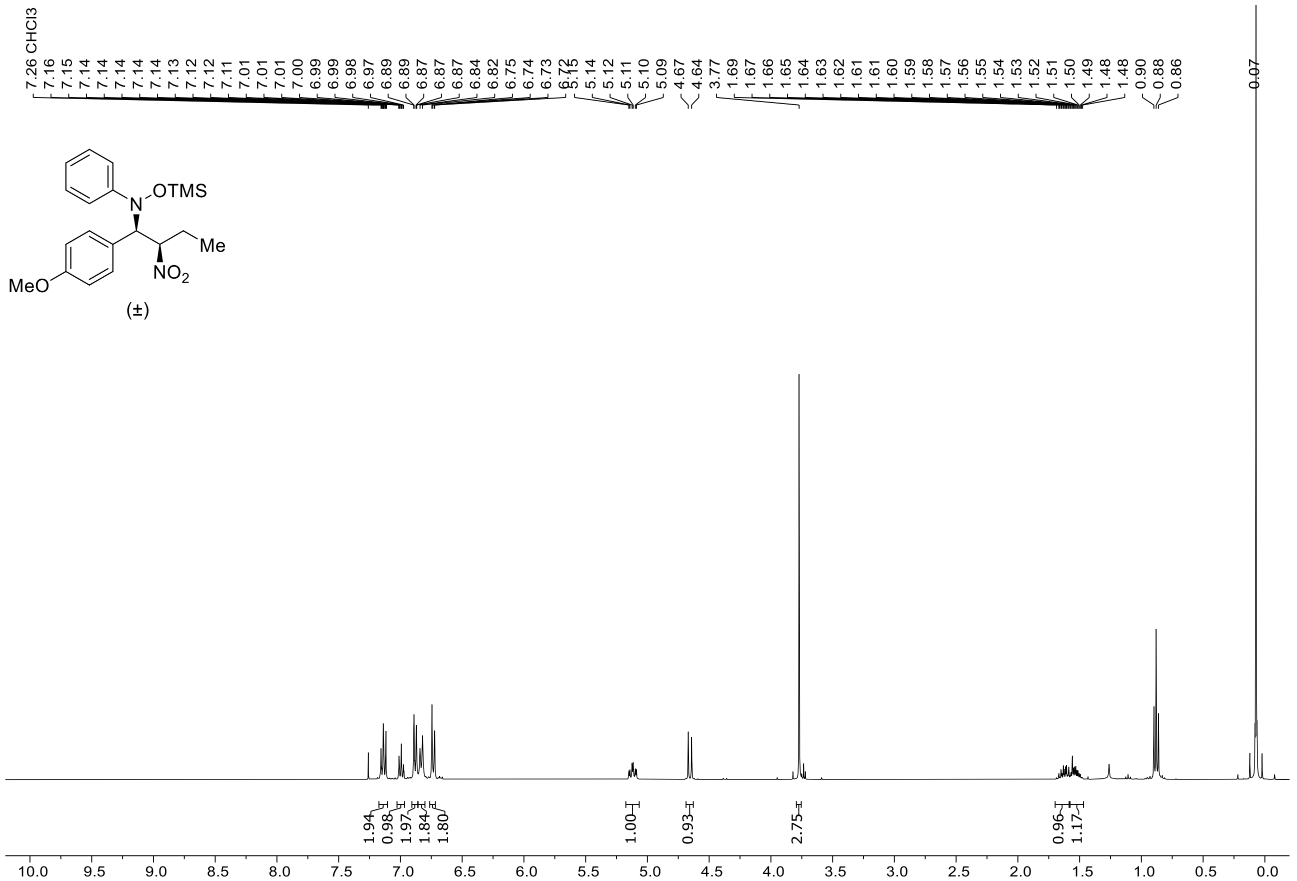

Figure S53. ^{13}C-NMR (101 MHz, $CDCl_3$, 298 K) spectrum of **3e**.

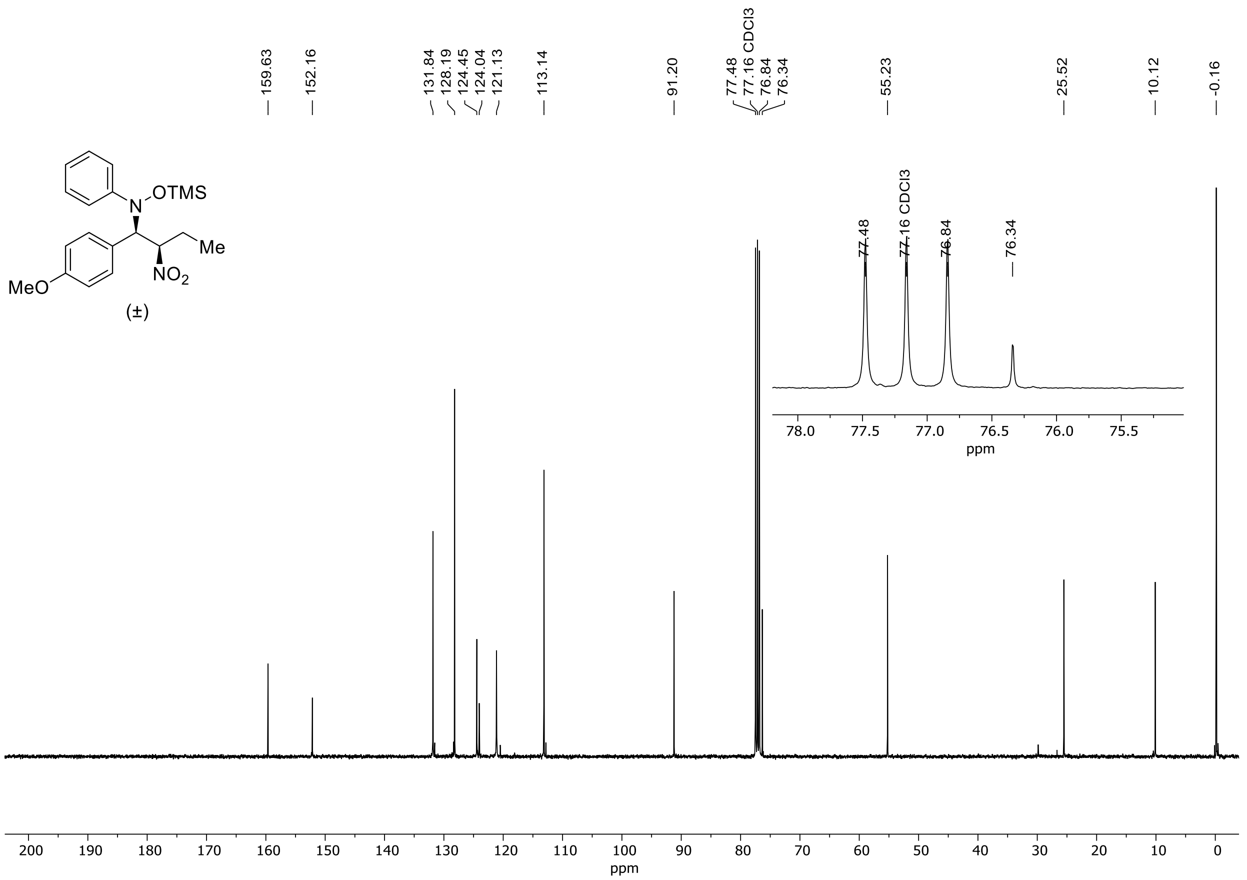

Figure S54. ^{1}H-NMR (400 MHz, $CDCl_3$, 298 K) spectrum of **3e'**.

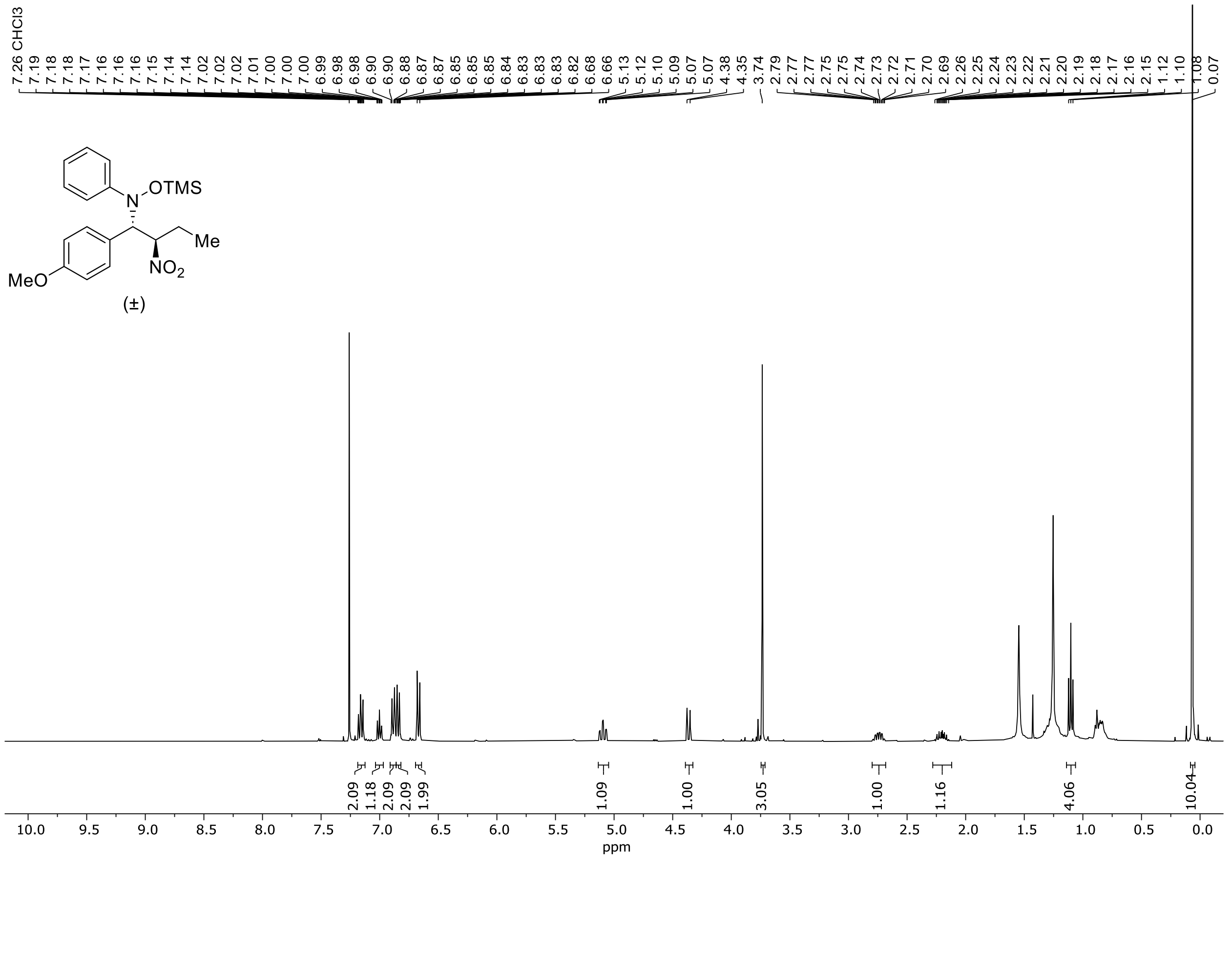

Figure S55. ^{13}C-NMR (101 MHz, $CDCl_3$, 298 K) spectrum of **3e'**.

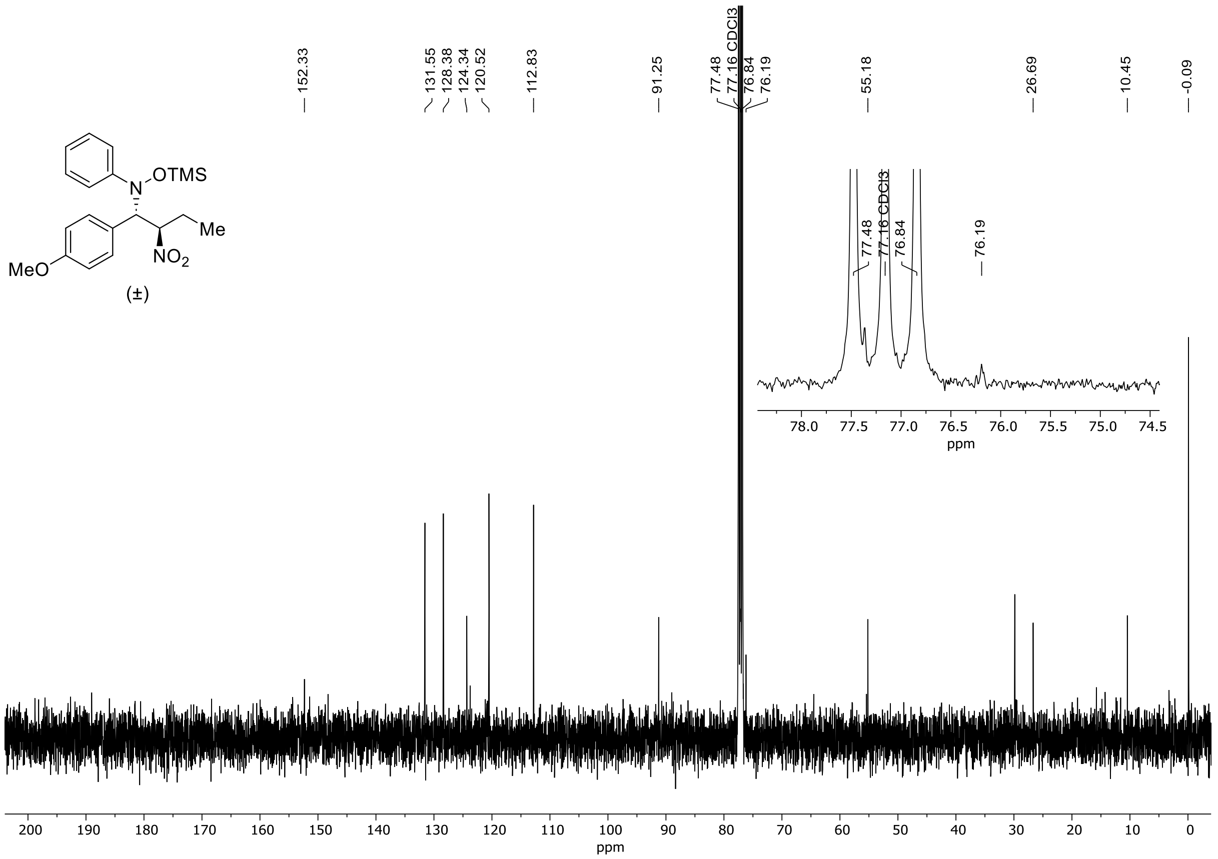

Figure S56. ^{1}H-NMR (400 MHz, $CDCl_3$, 298 K) spectrum of **3f**.

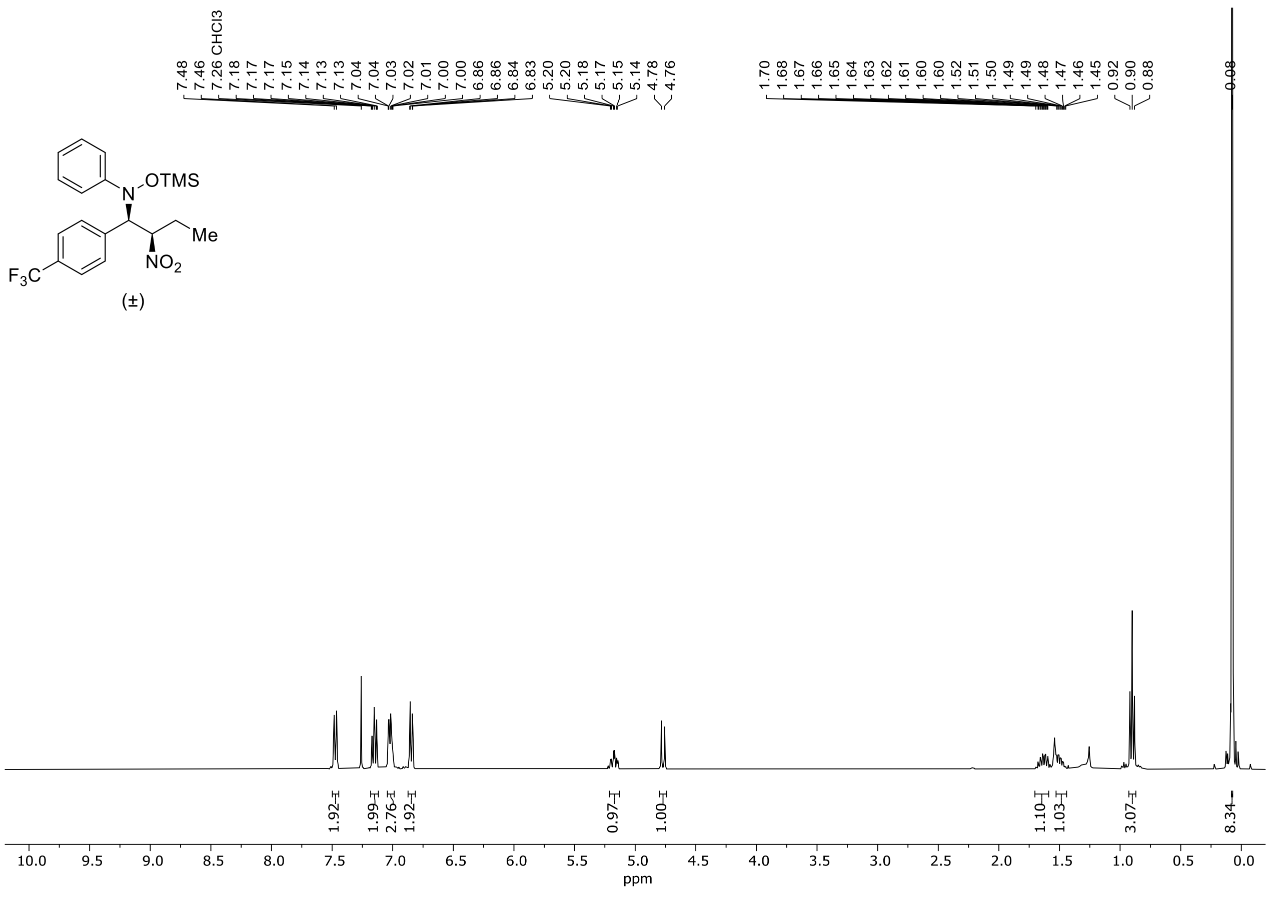

Figure S57. ^{13}C-NMR (101 MHz, $CDCl_3$, 298 K) spectrum of **3f**.

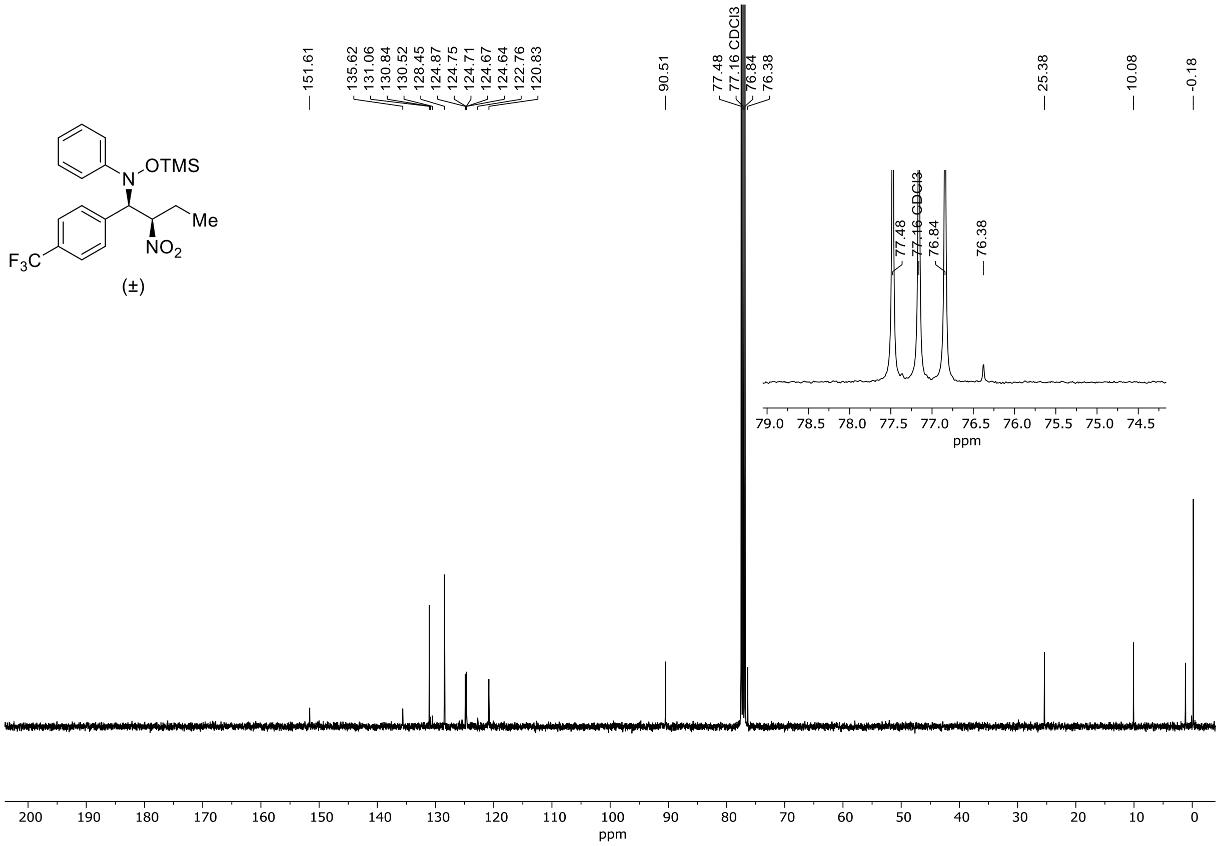

Figure S58. ^{19}F-NMR (376 MHz, $CDCl_3$, 298 K) spectrum of **3f**.

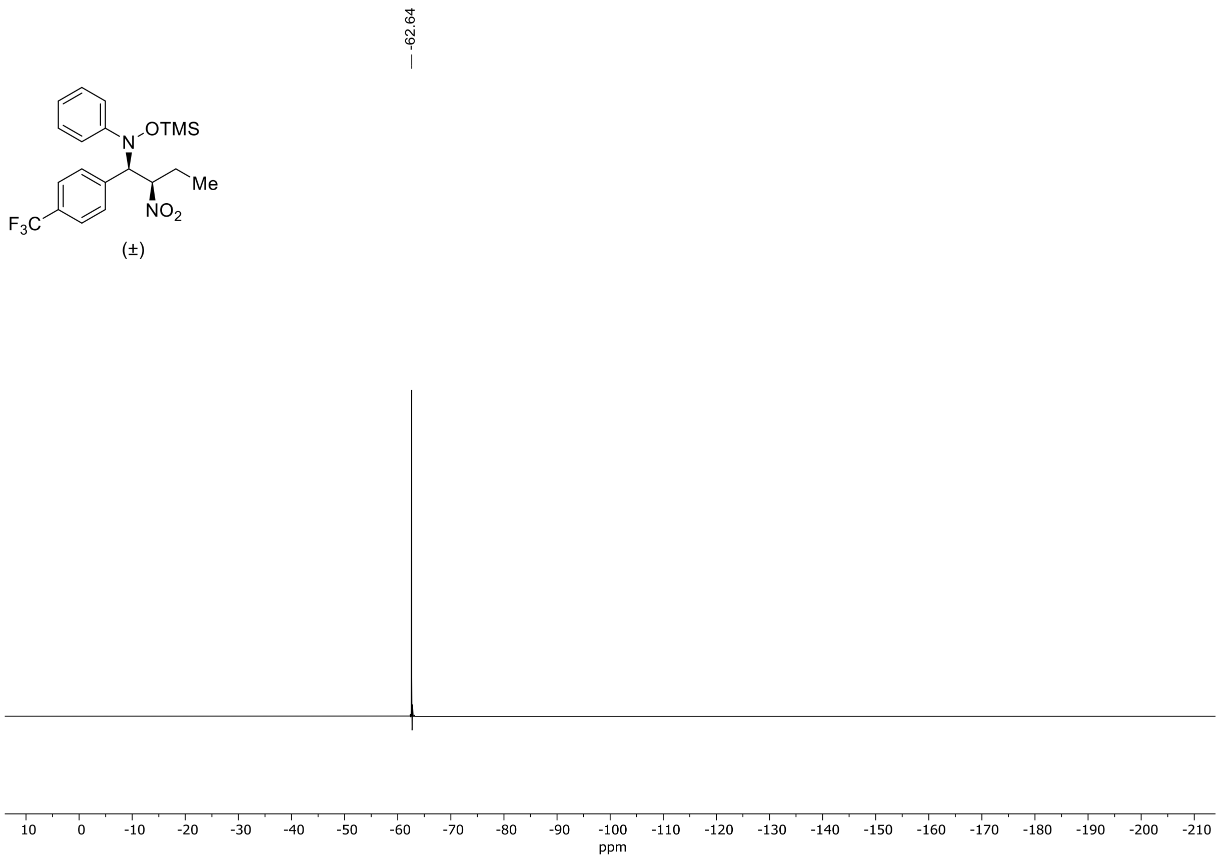

Figure S59. ^{1}H-NMR (400 MHz, $CDCl_3$, 298 K) spectrum of **3f'**.

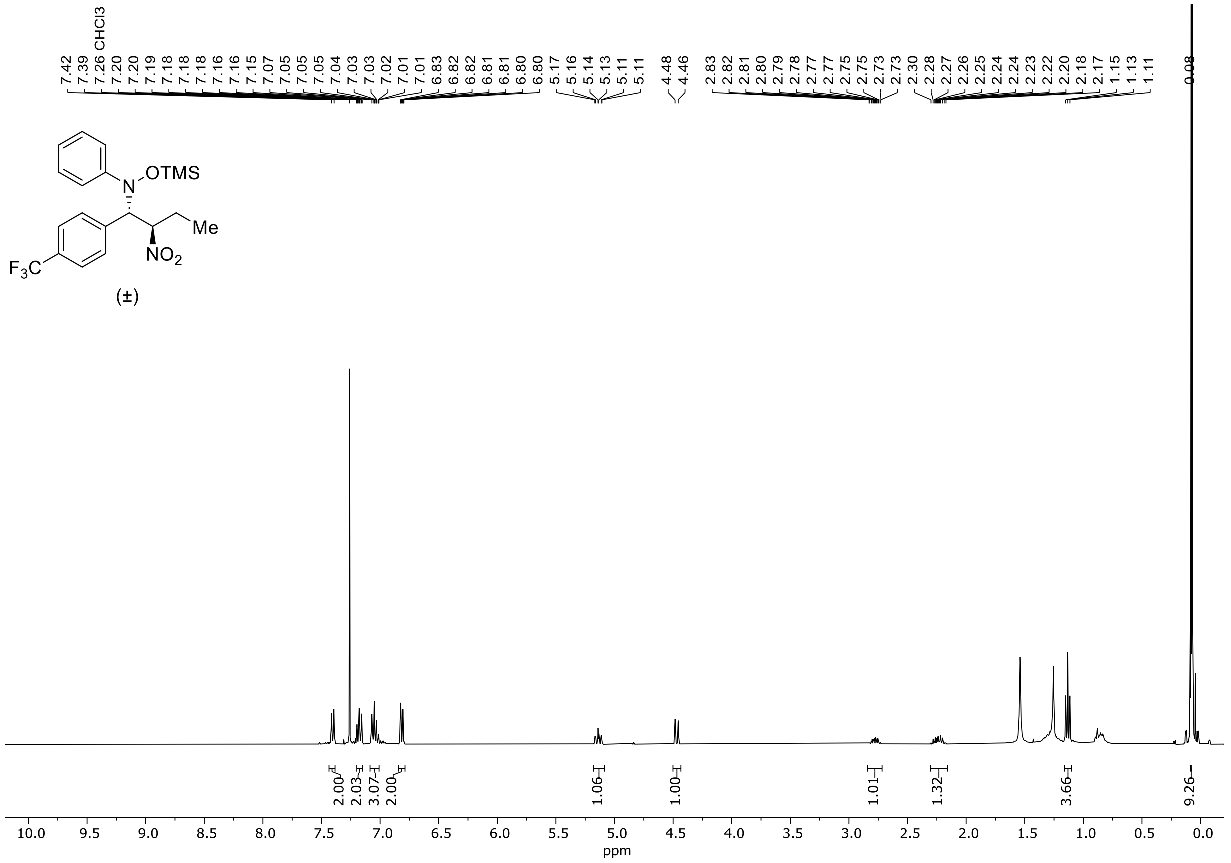

Figure S60. ^{13}C-NMR (101 MHz, $CDCl_3$, 298 K) spectrum of **3f'**.

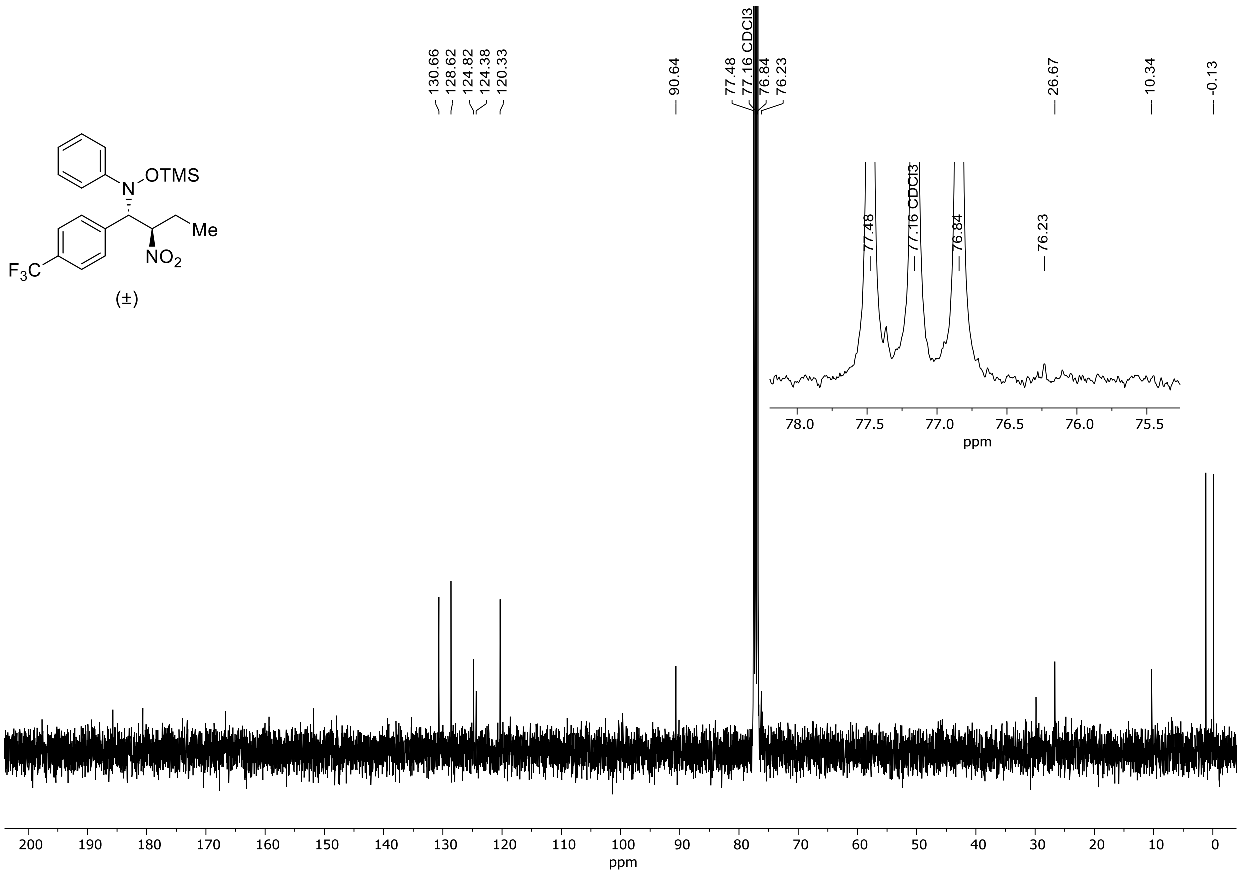

Figure S61. ^{19}F-NMR (376 MHz, $CDCl_3$, 298 K) spectrum of **3f’**.

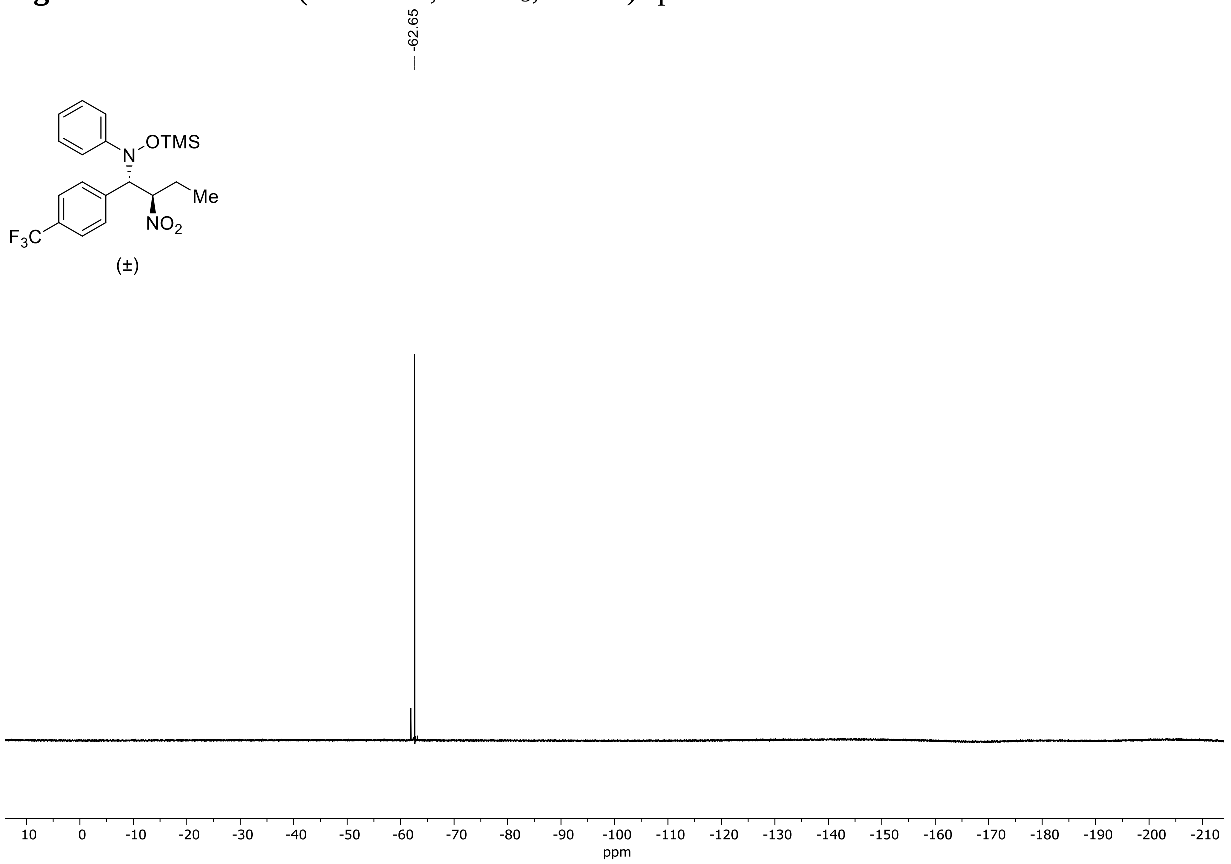

Figure S62. ^{1}H-NMR (400 MHz, $CDCl_3$, 298 K) spectrum of **3g**.

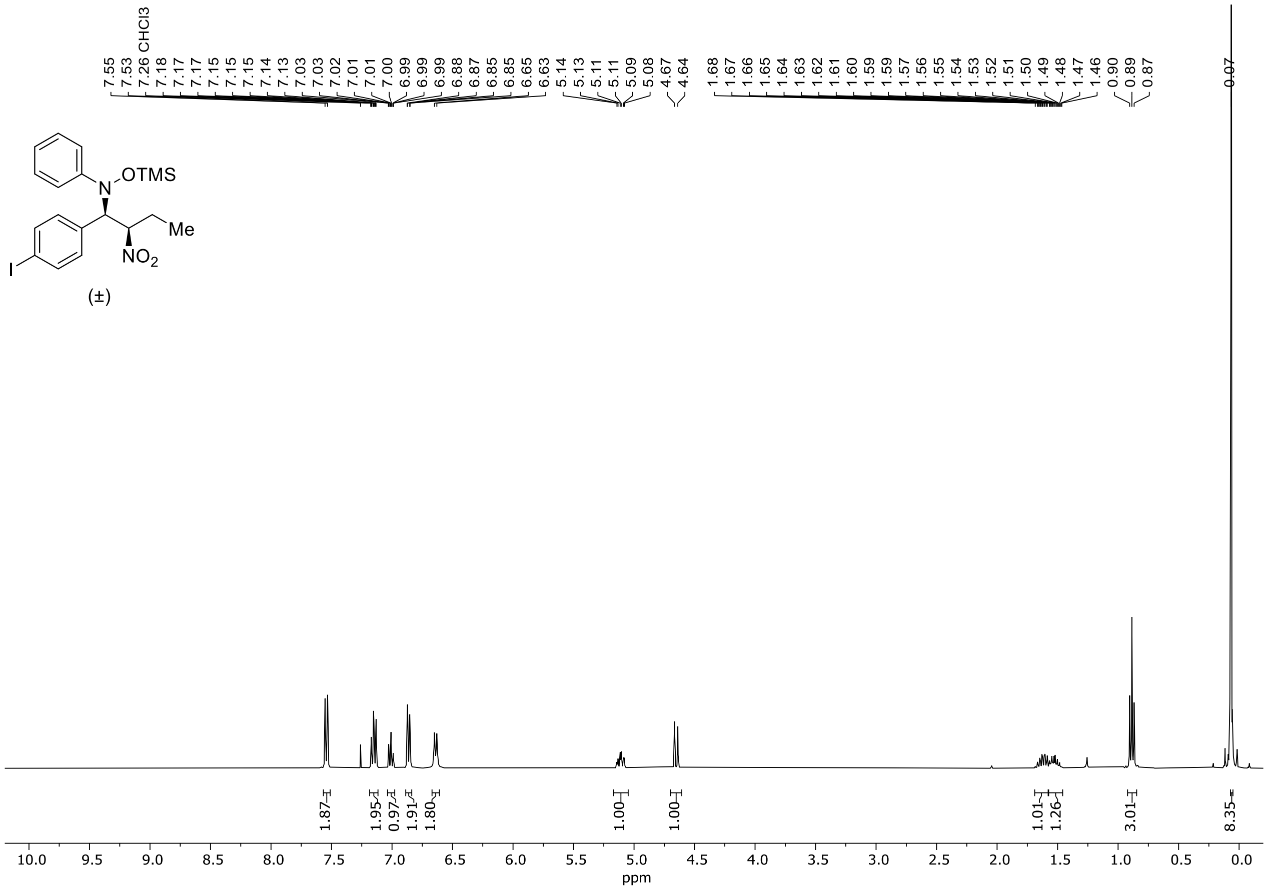

Figure S63. ^{13}C-NMR (101 MHz, $CDCl_3$, 298 K) spectrum of **3g**.

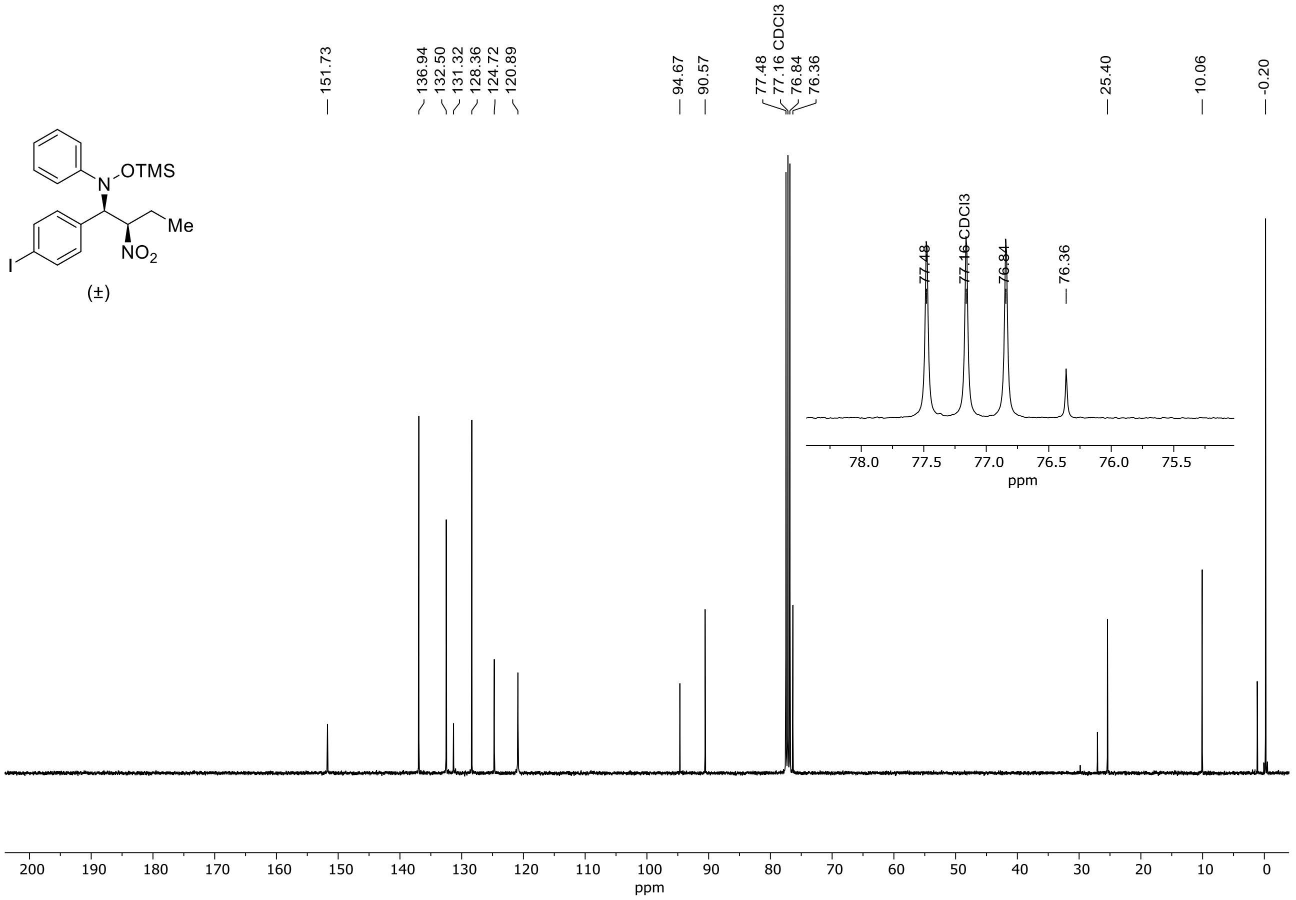

Figure S64. ^{1}H-NMR (400 MHz, $CDCl_3$, 298 K) spectrum of **3g'**.

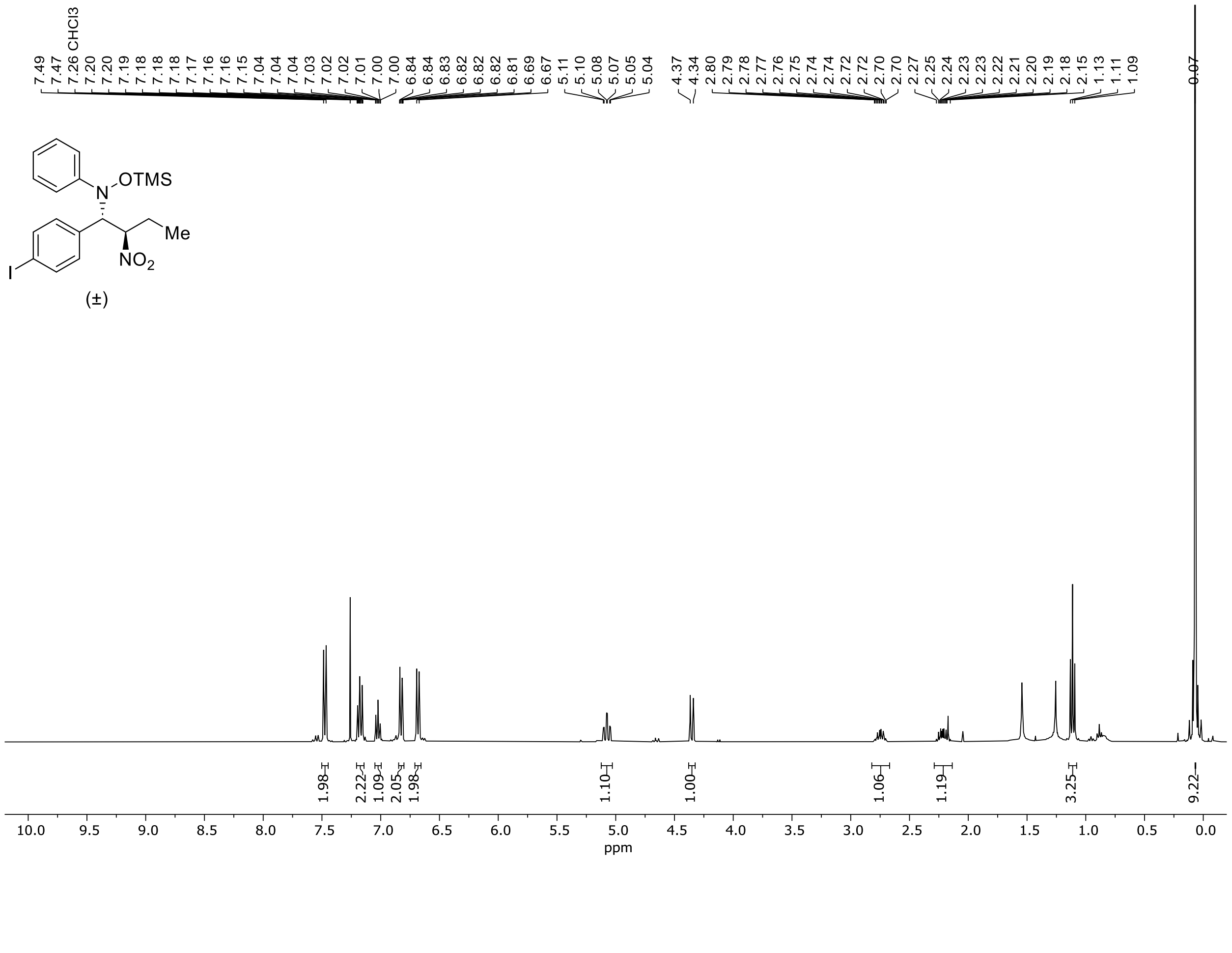

Figure S65. ^{13}C-NMR (101 MHz, $CDCl_3$, 298 K) spectrum of **3g'**.

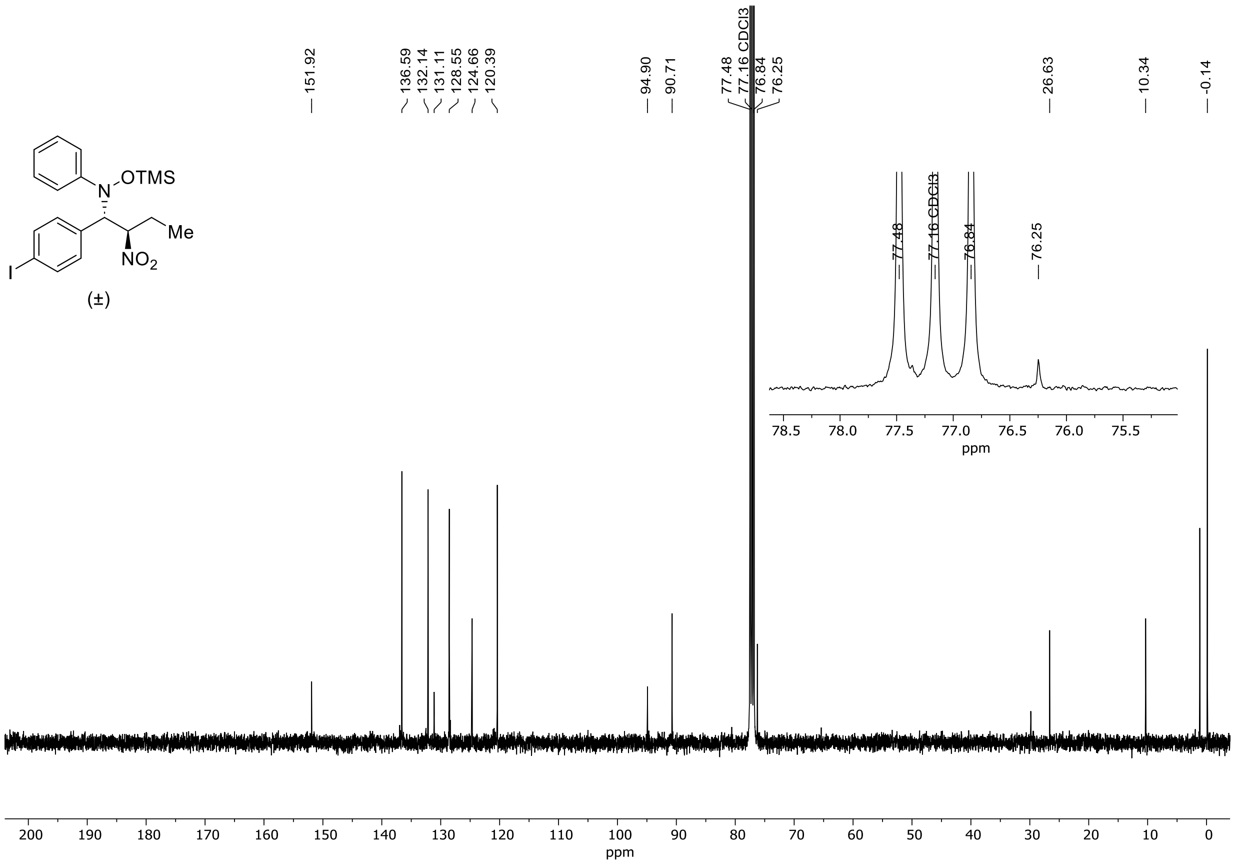

Figure S66. ^{1}H-NMR (400 MHz, $CDCl_3$, 298 K) spectrum of **3h**.

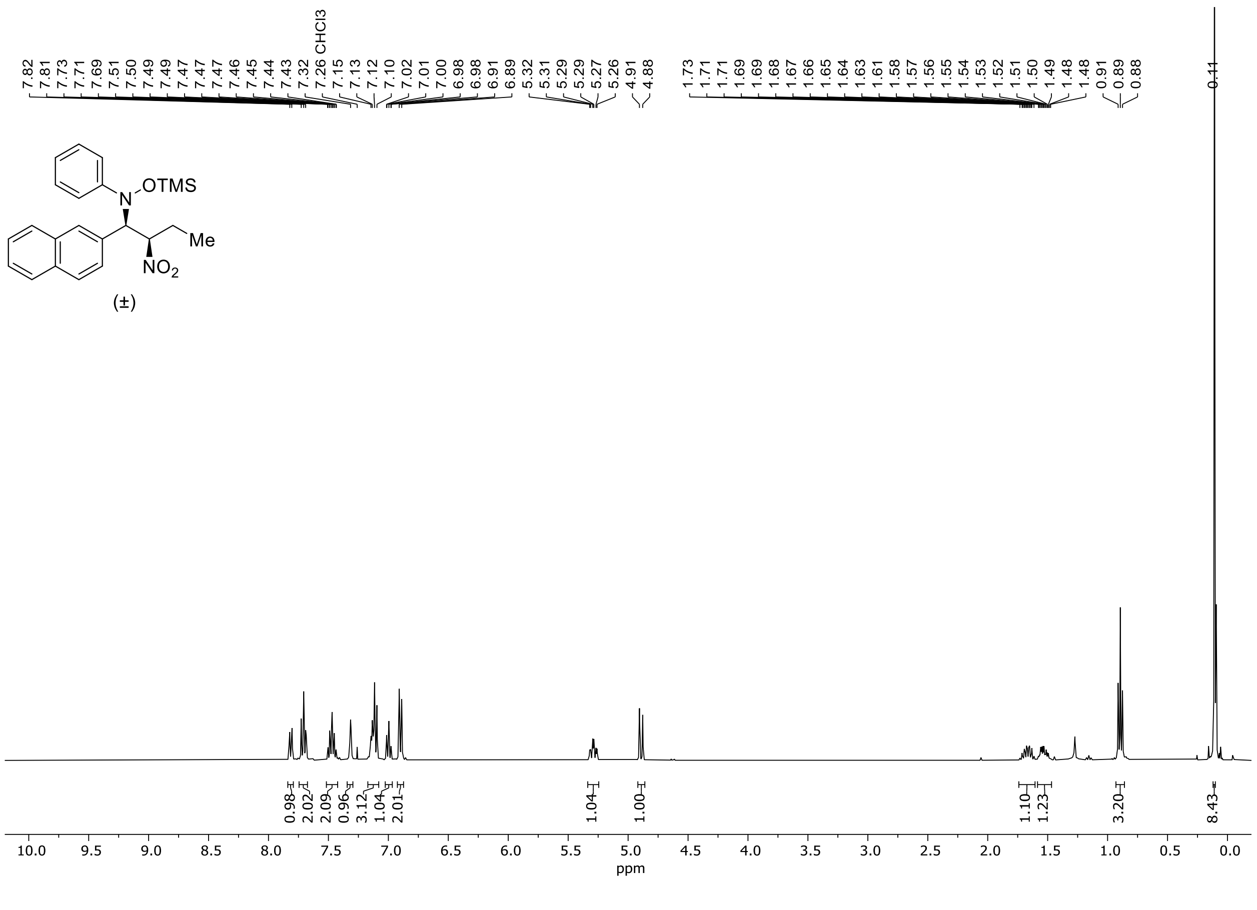

Figure S67. ^{13}C-NMR (101 MHz, $CDCl_3$, 298 K) spectrum of **3h**.

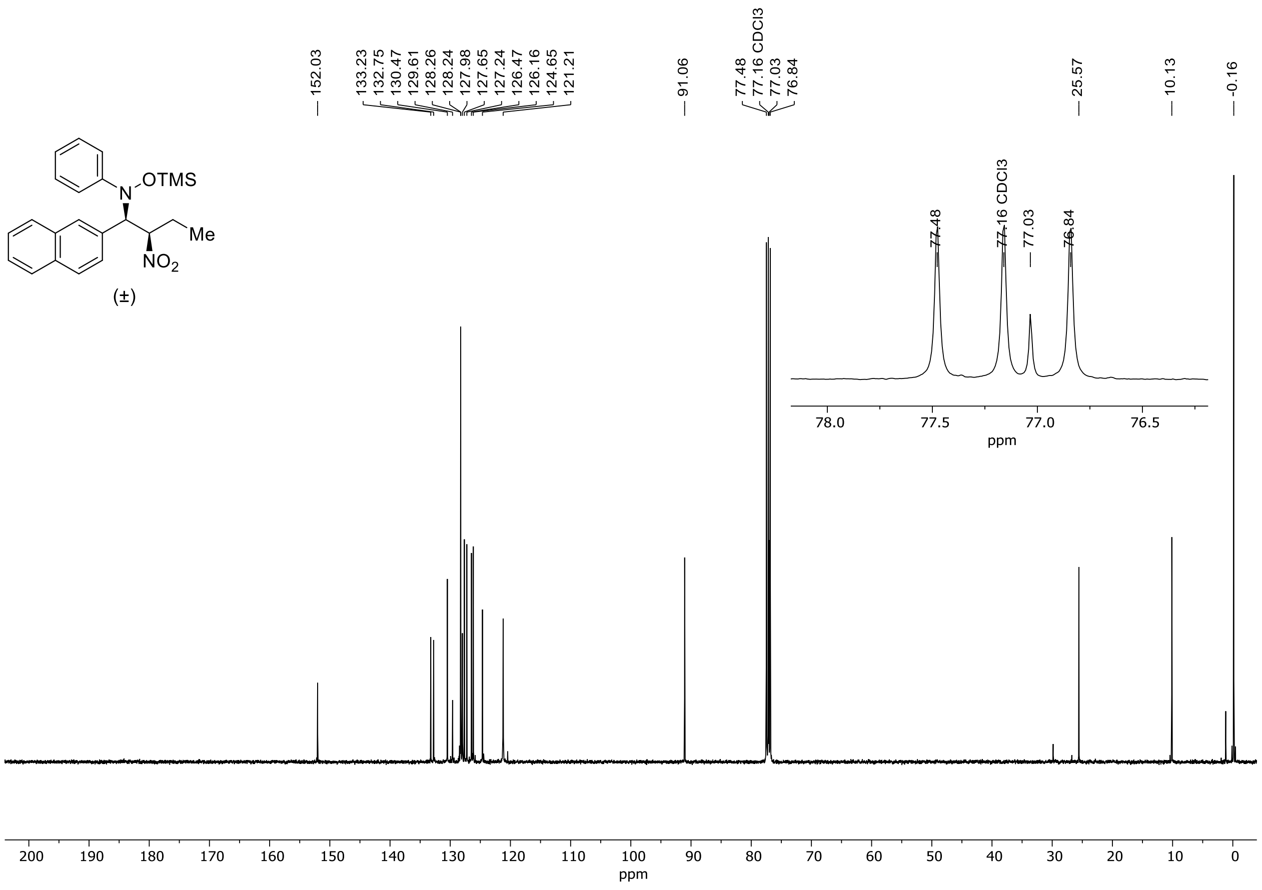

Figure S68. ^{1}H-NMR (400 MHz, $CDCl_3$, 298 K) spectrum of **3h'**.

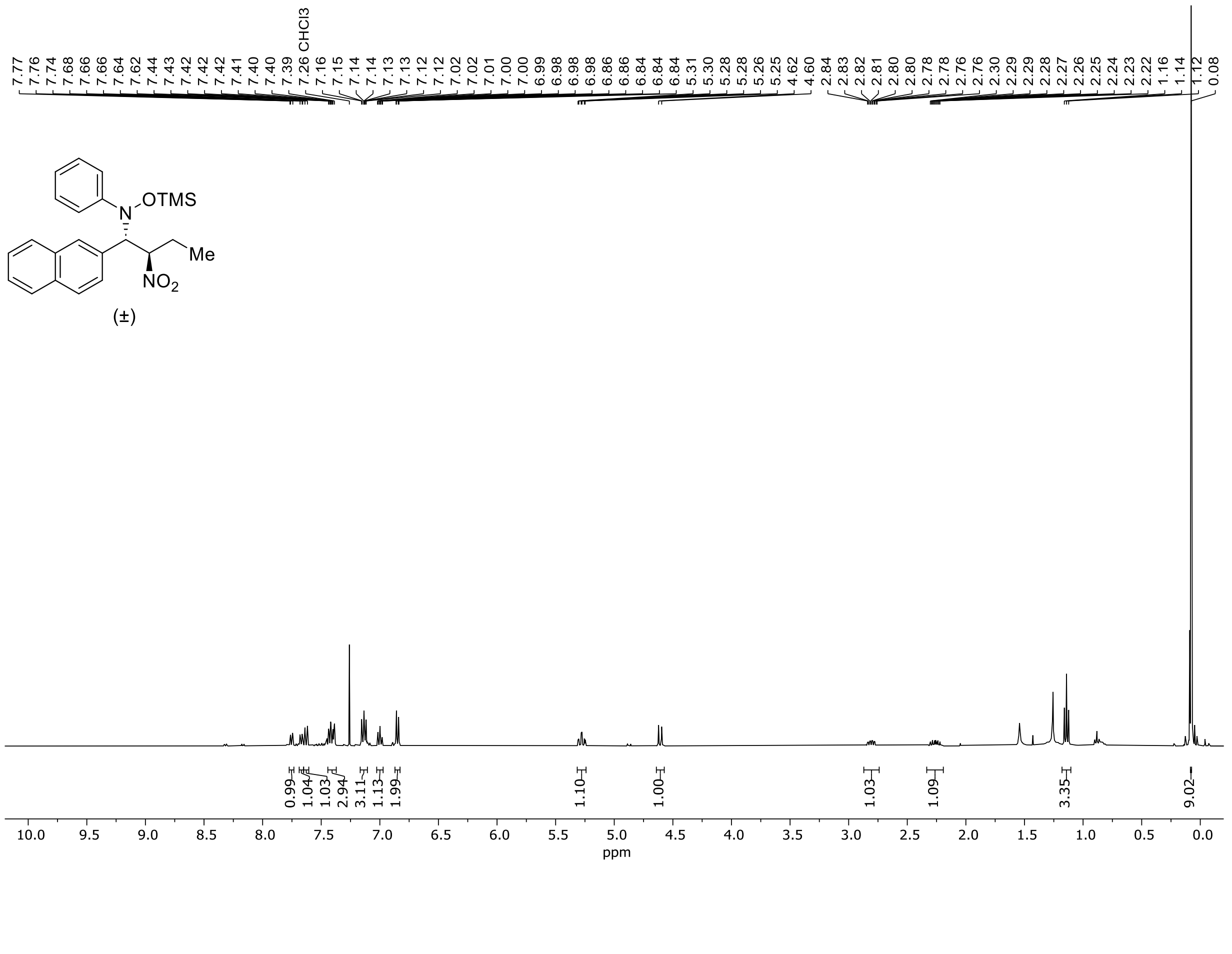

Figure S69. ^{13}C-NMR (101 MHz, $CDCl_3$, 298 K) spectrum of **3h'**.

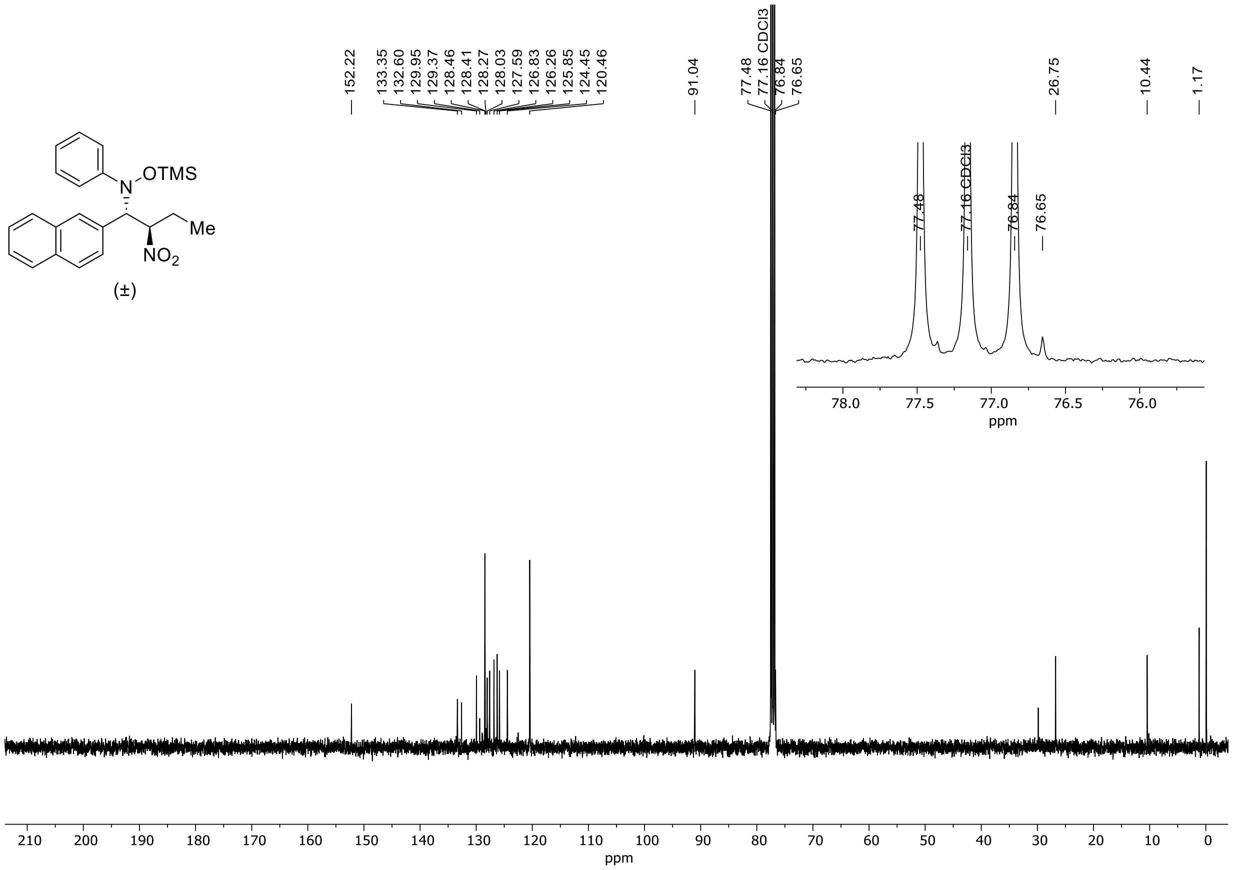

Figure S70. ^{1}H-NMR (400 MHz, $CDCl_3$, 298 K) spectrum of **3i**.

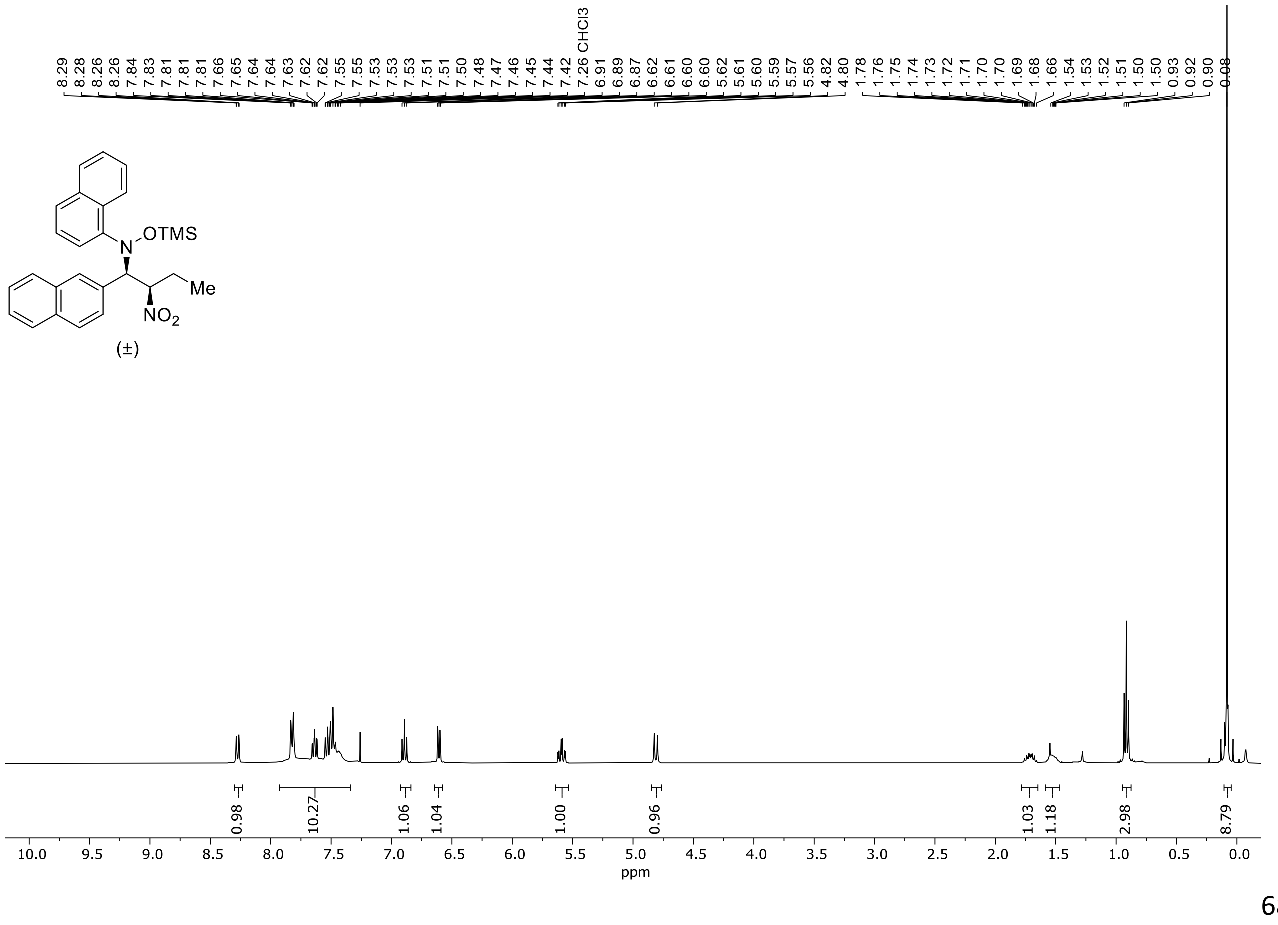

Figure S71. ^{13}C-NMR (101 MHz, $CDCl_3$, 298 K) spectrum of **3i**.

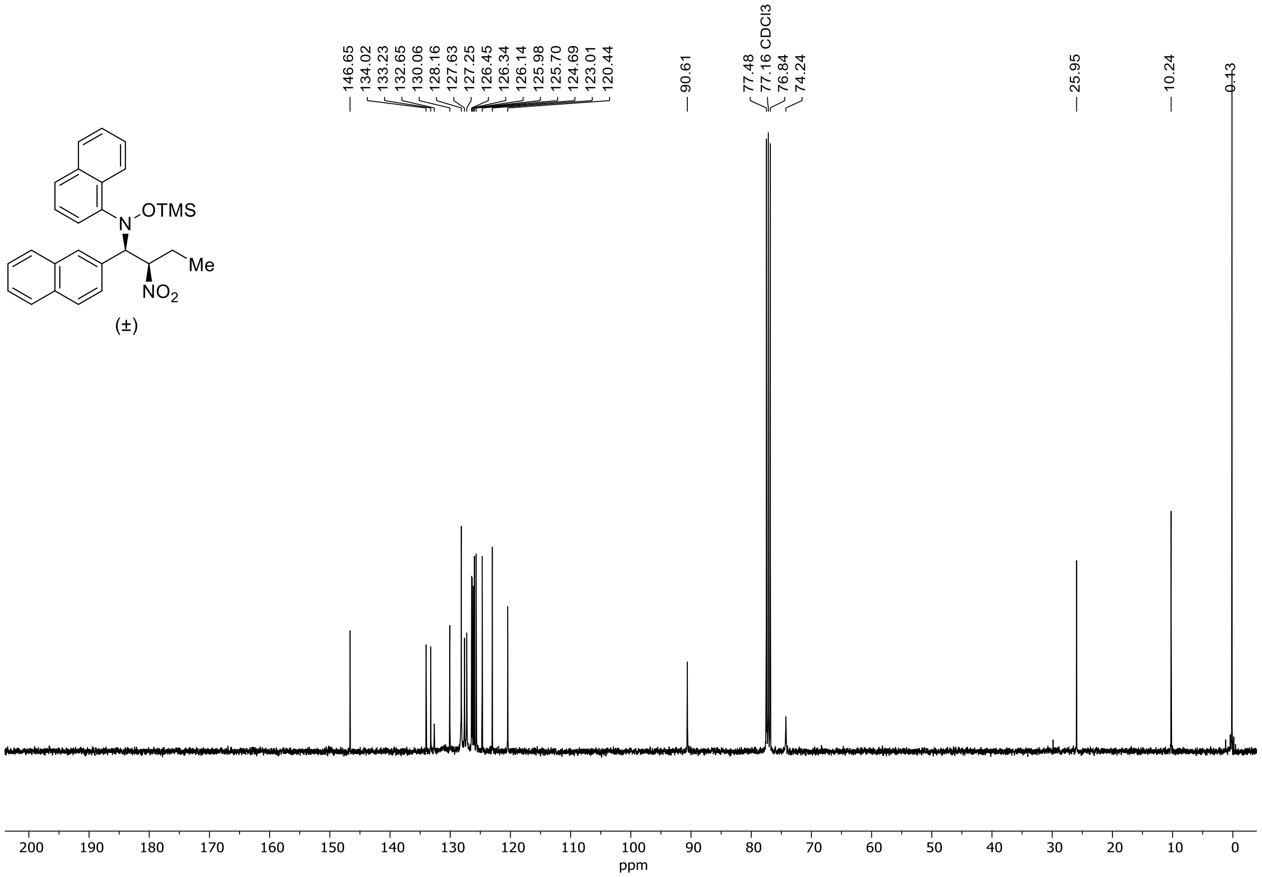

Figure S72. ^{1}H-NMR (400 MHz, $CDCl_3$, 298 K) spectrum of **3j**.

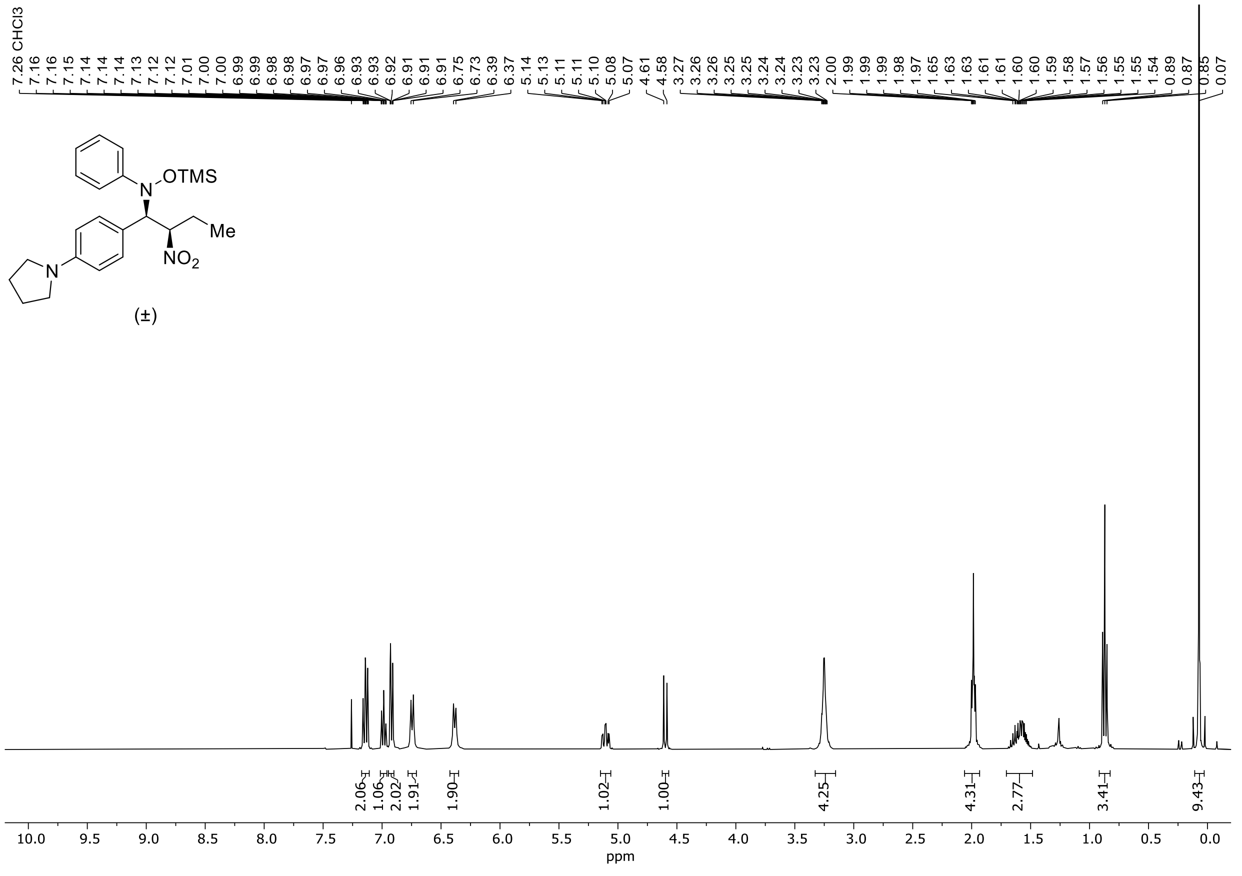

Figure S73. ^{13}C-NMR (101 MHz, $CDCl_3$, 298 K) spectrum of **3j**.

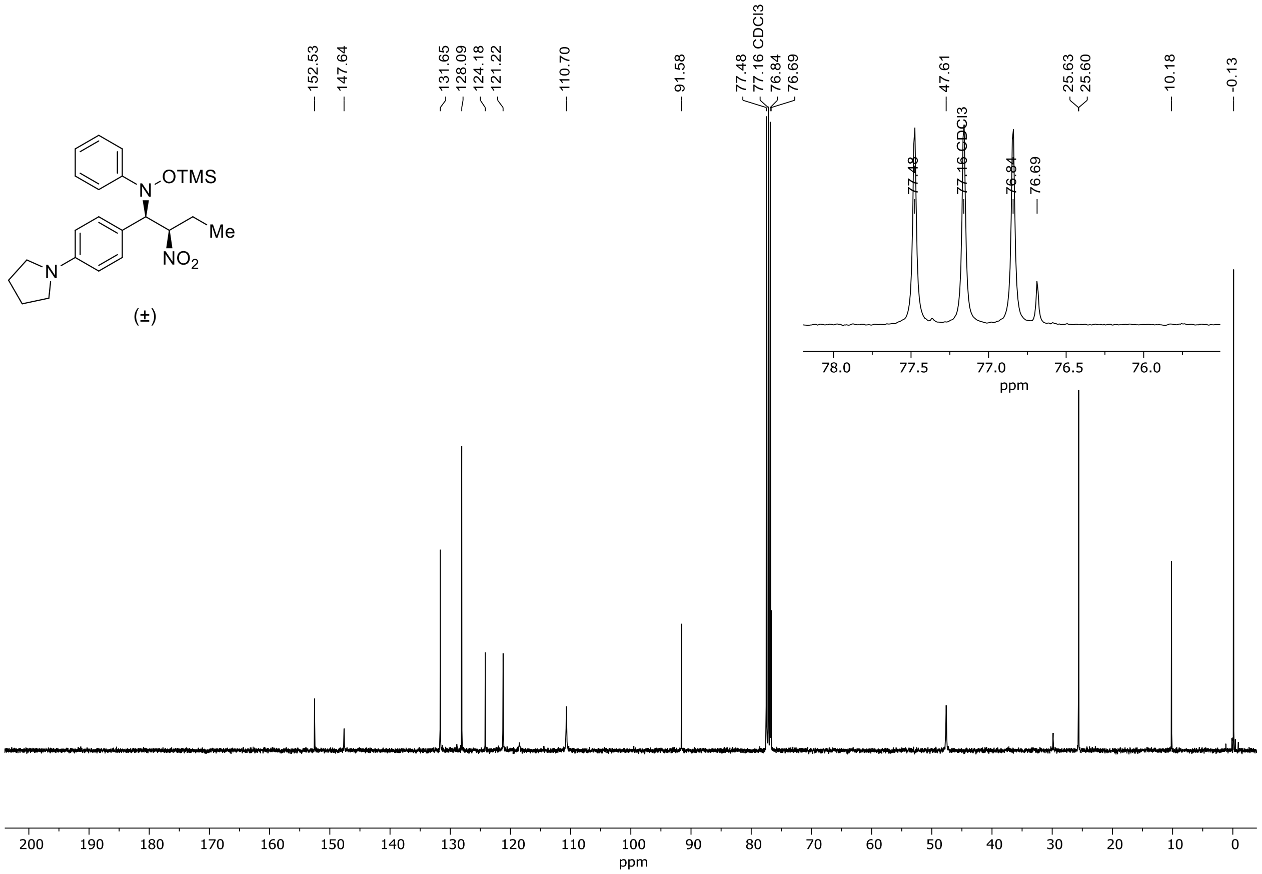

Figure S74. ^{1}H-NMR (400 MHz, $CDCl_3$, 298 K) spectrum of **3k**.

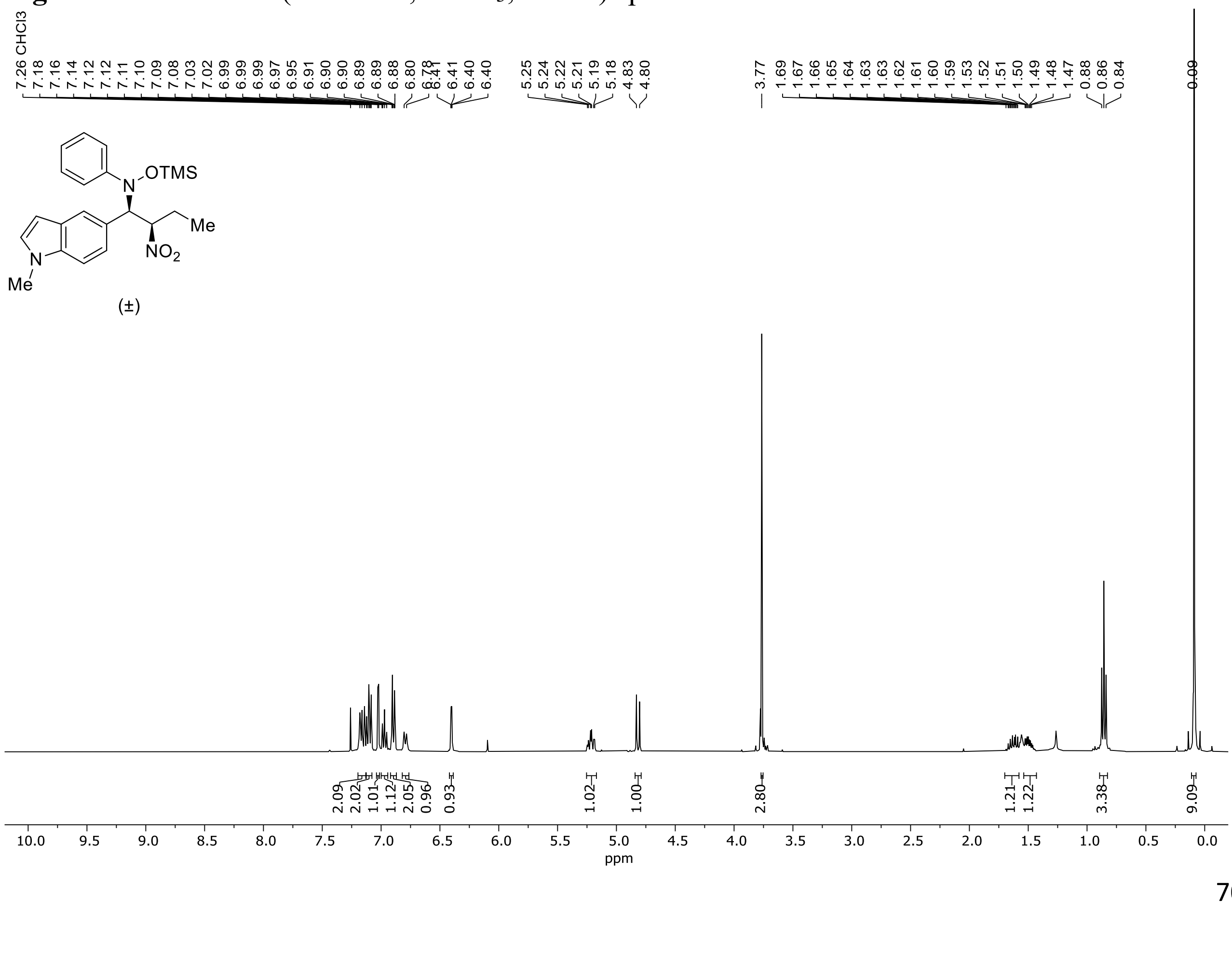

Figure S75. ^{13}C-NMR (101 MHz, $CDCl_3$, 298 K) spectrum of **3k**.

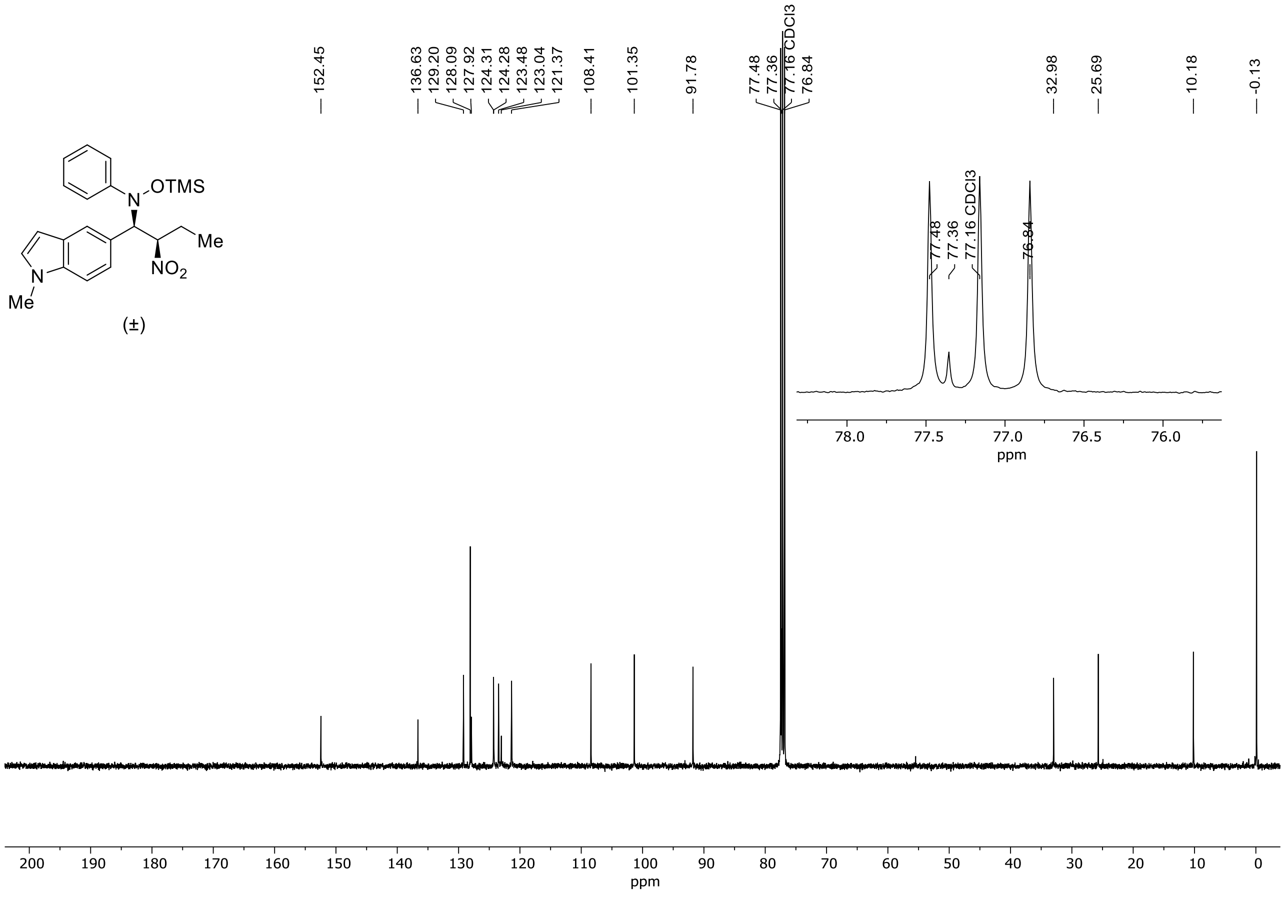

Figure S76. ^{1}H-NMR (400 MHz, $CDCl_3$, 298 K) spectrum of **3l**.

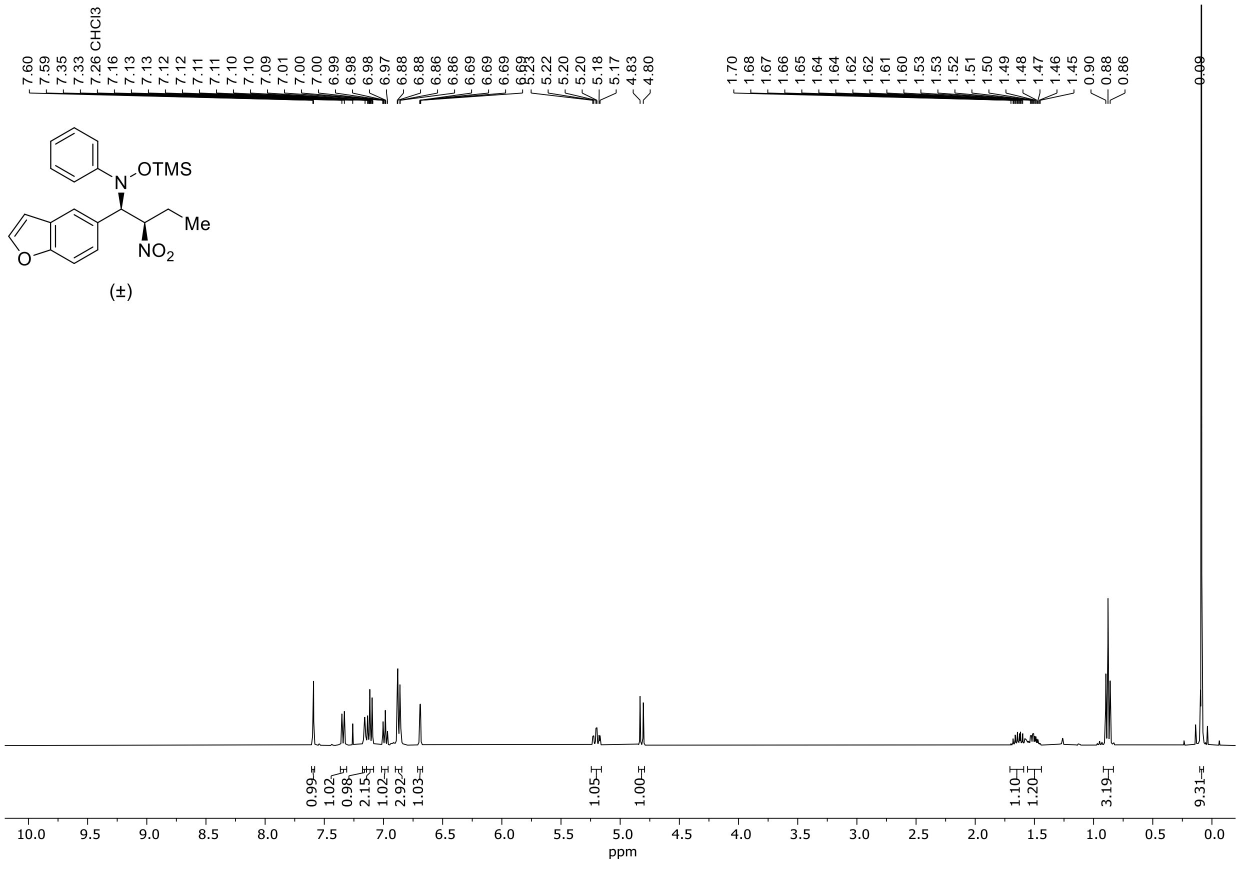

Figure S77. ^{13}C-NMR (101 MHz, $CDCl_3$, 298 K) spectrum of **3l**.

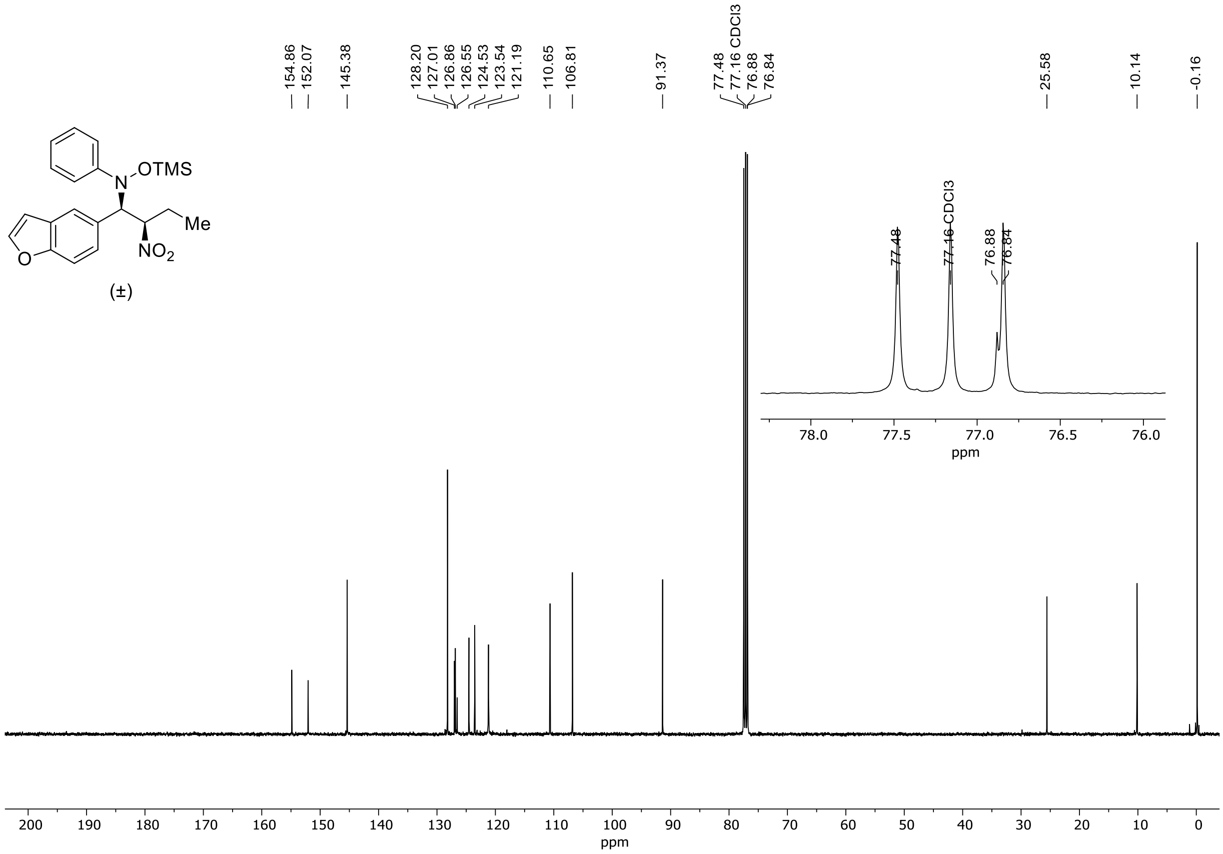

Figure S78. ^{1}H-NMR (400 MHz, $CDCl_3$, 298 K) spectrum of **3l'**.

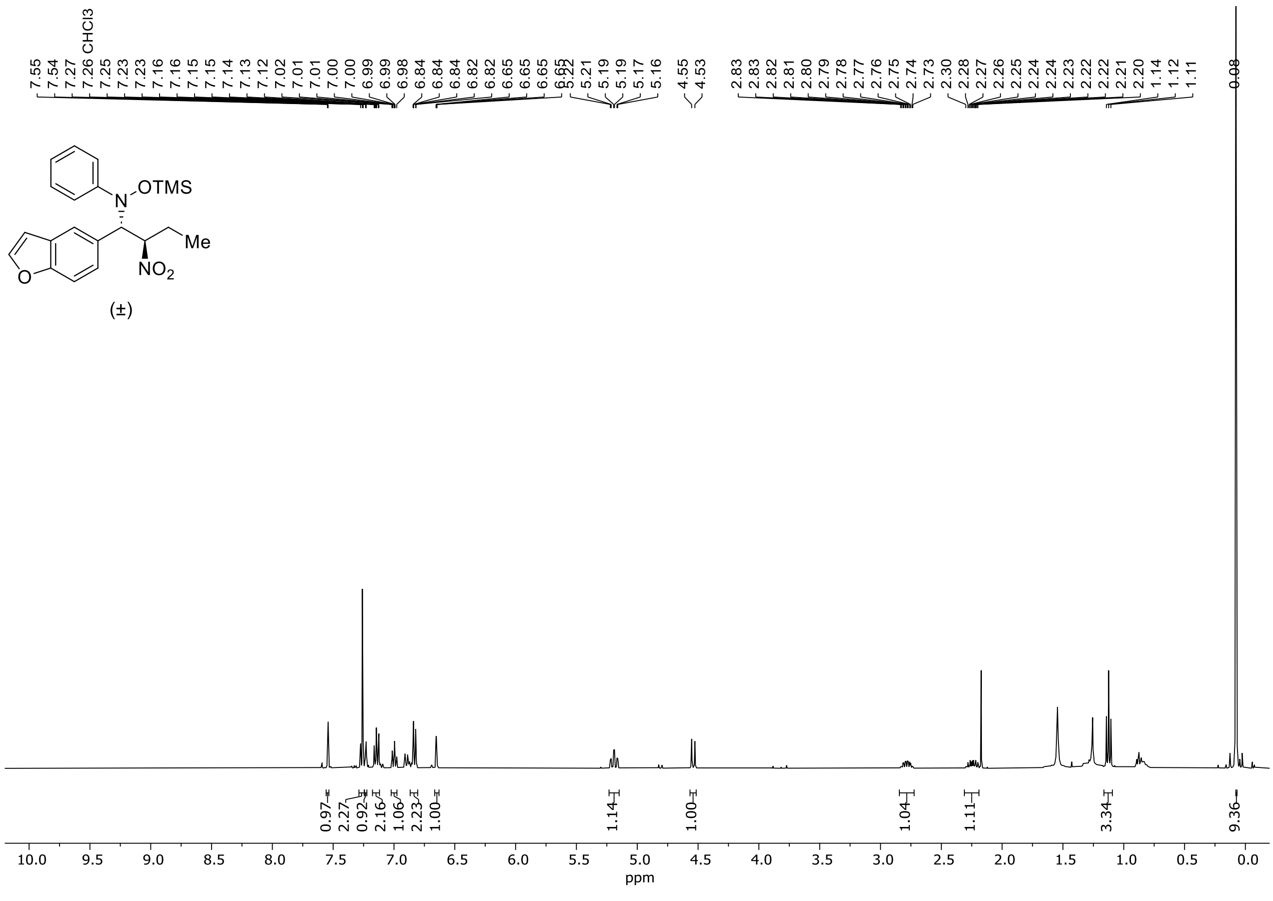

Figure S79. ^{13}C-NMR (101 MHz, $CDCl_3$, 298 K) spectrum of **3l'**.

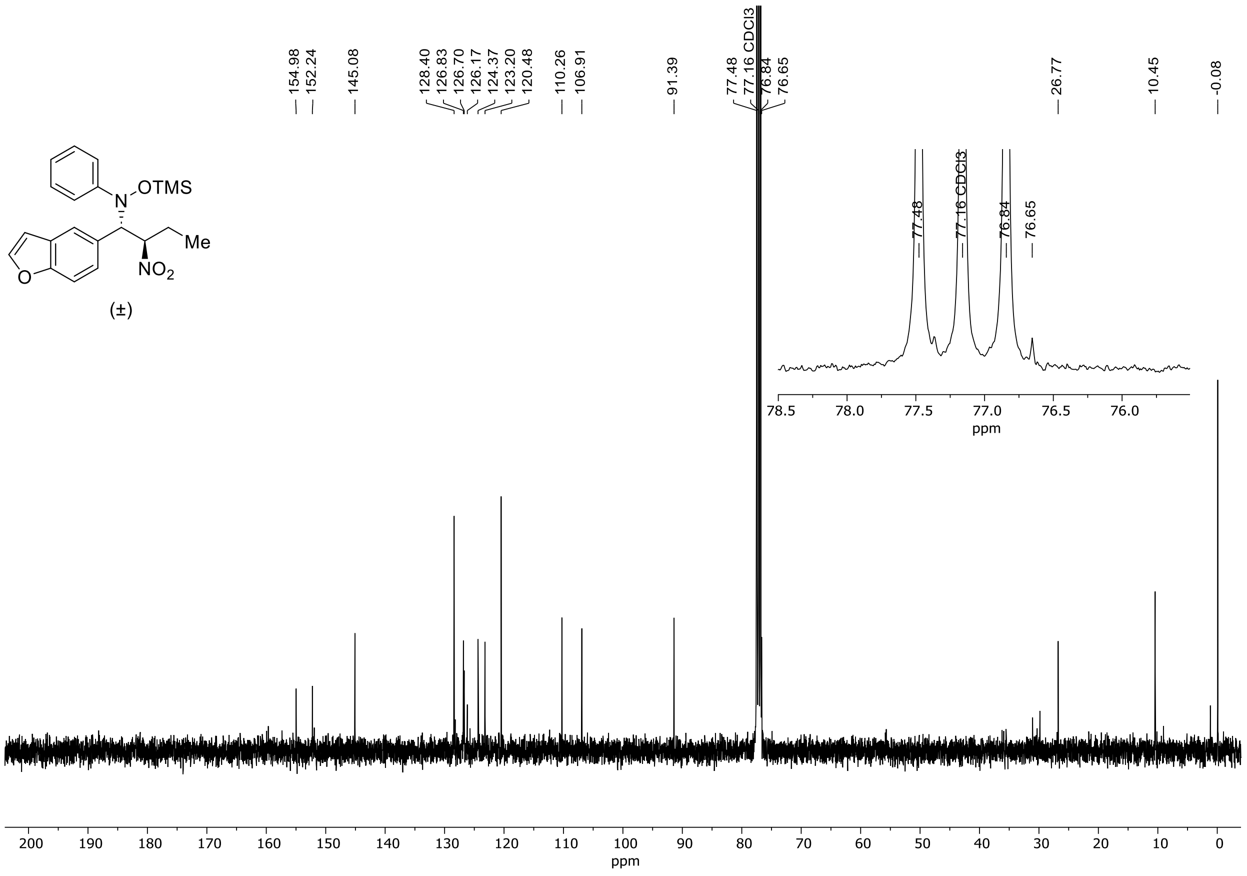

Figure S80. ^{1}H-NMR (400 MHz, $CDCl_3$, 298 K) spectrum of **3m.**

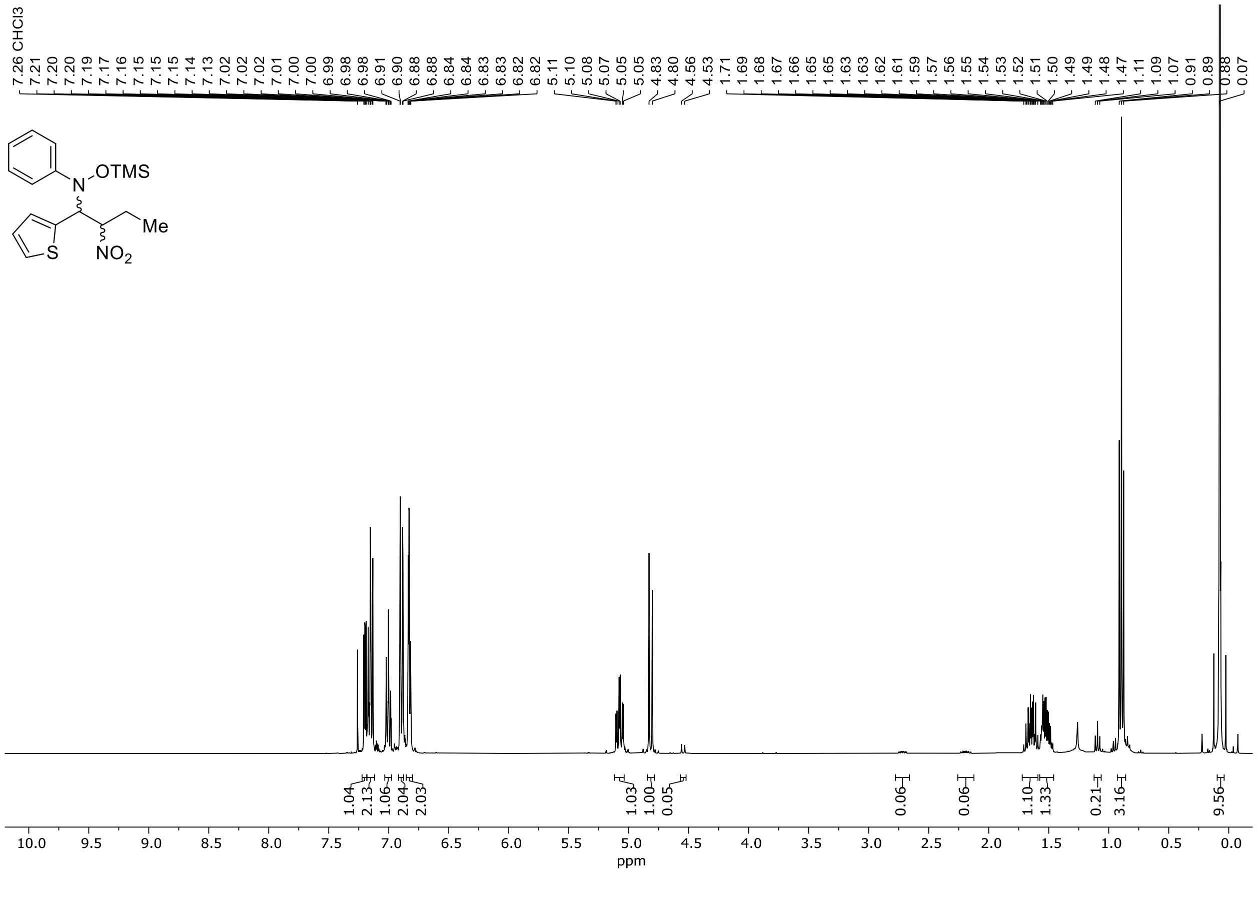

Figure S81. ^{13}C-NMR (101 MHz, $CDCl_3$, 298 K) spectrum of **3m**.

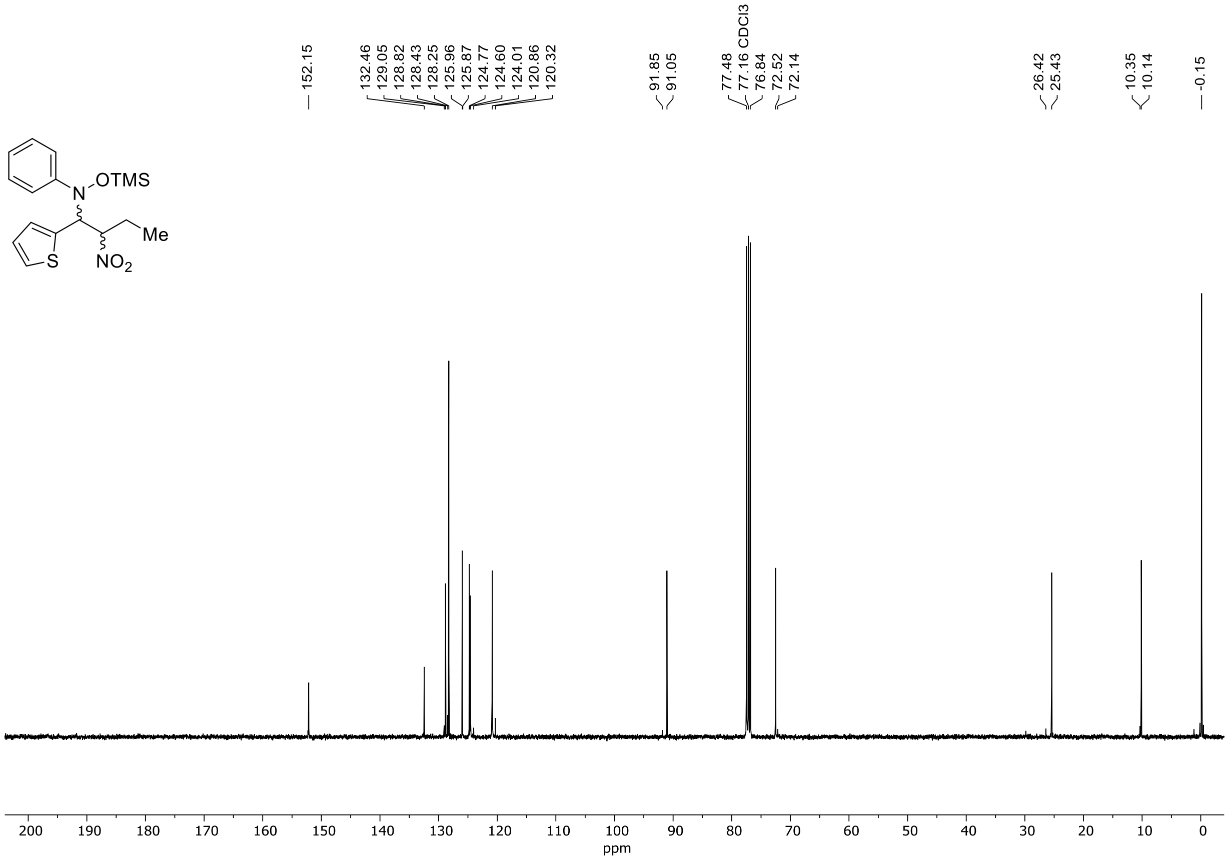

Figure S82. ^{1}H-NMR (400 MHz, $CDCl_3$, 298 K) spectrum of **3n**.

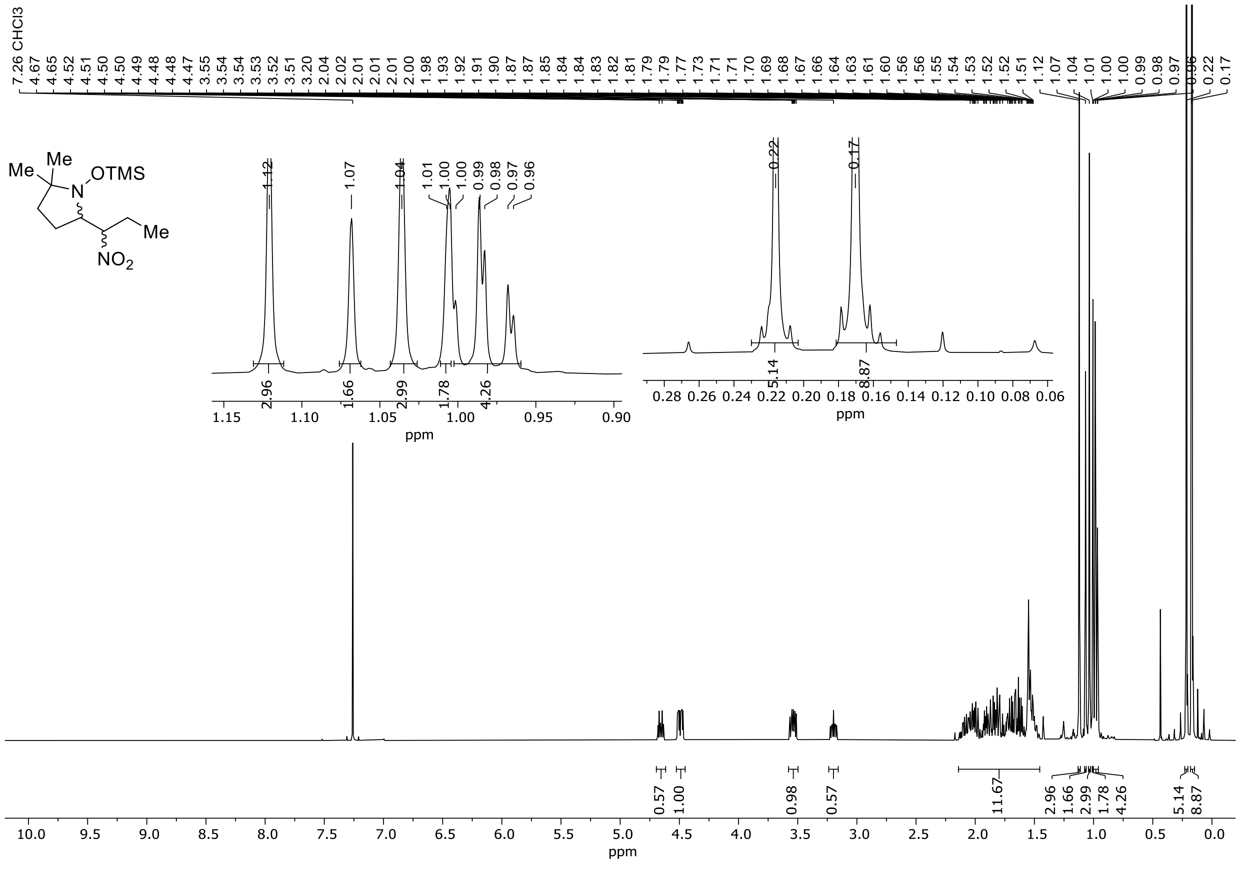

Figure S83. ^{13}C-NMR (101 MHz, $CDCl_3$, 298 K) spectrum of **3n**.

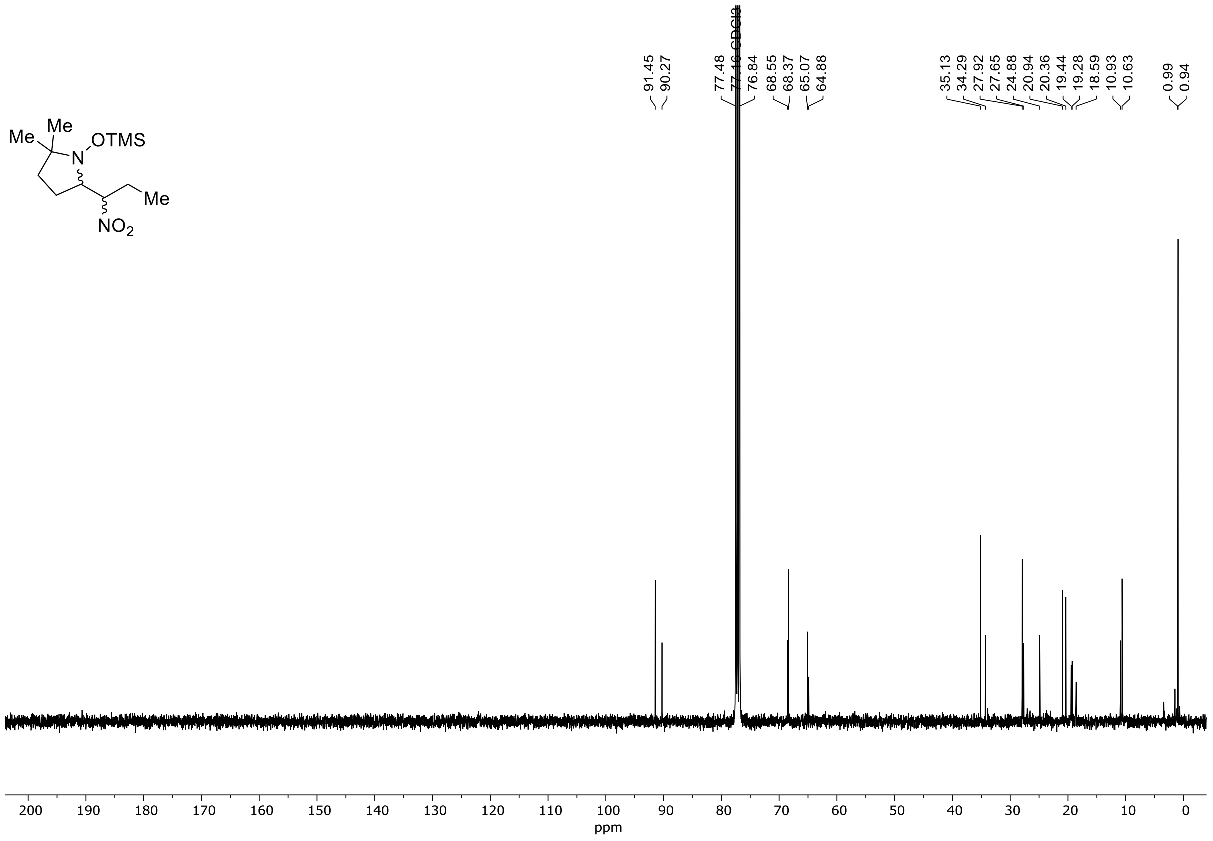

Figure S84. ^{1}H-NMR (400 MHz, $CDCl_3$, 298 K) spectrum of **3o**.

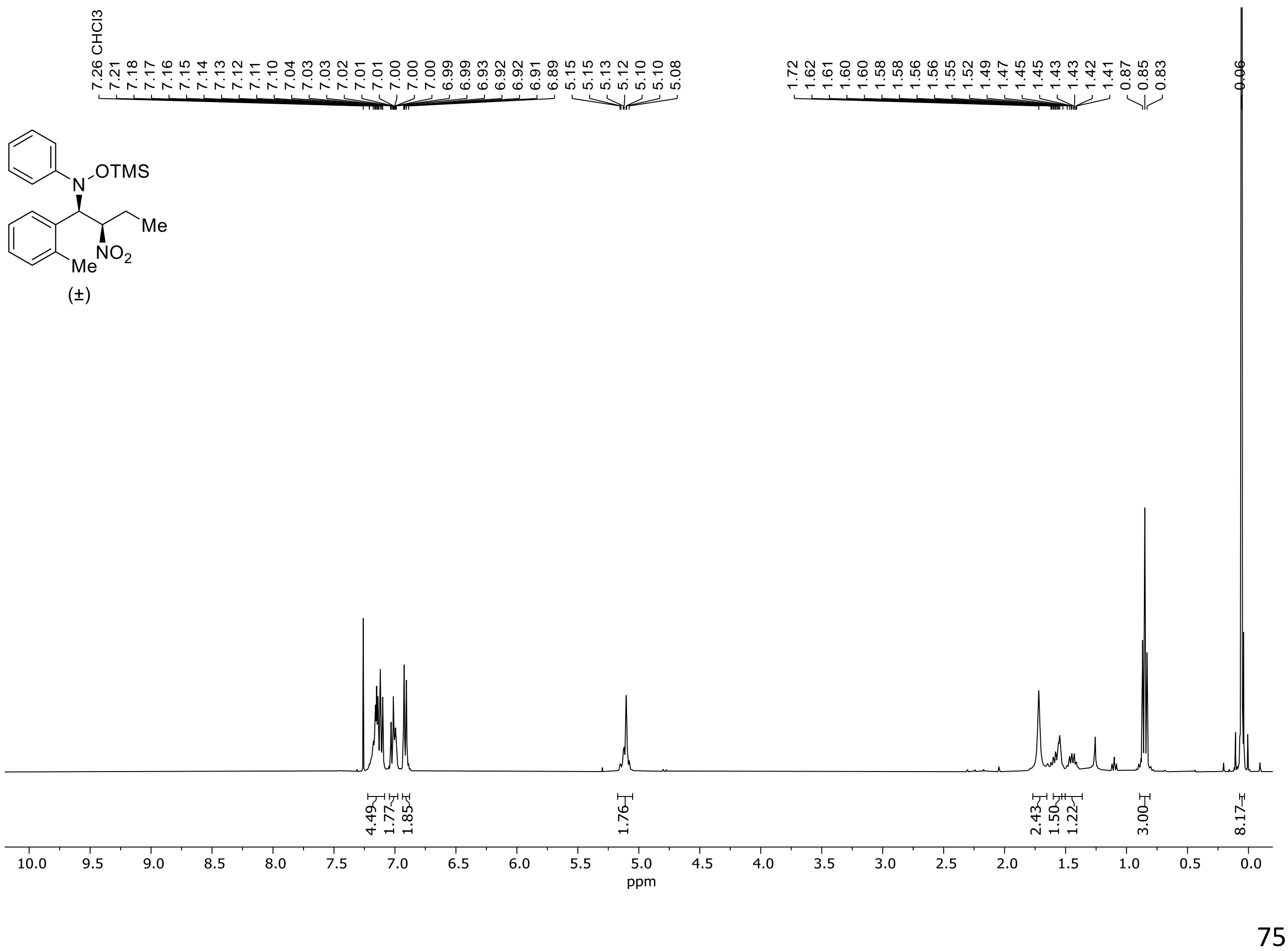

Figure S85. ^{13}C-NMR (101 MHz, $CDCl_3$, 298 K) spectrum of **3o**.

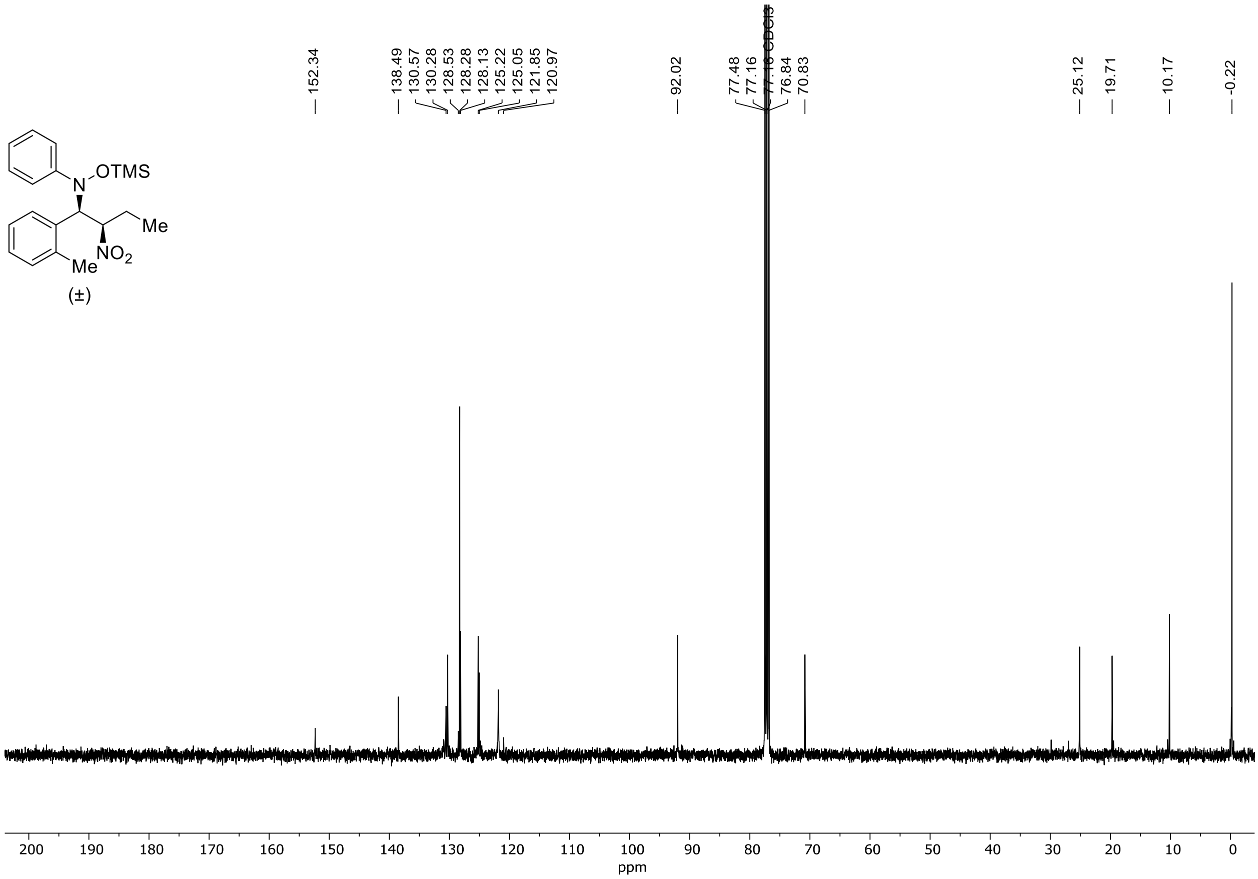

Figure S86. ^{1}H-NMR (400 MHz, $CDCl_3$, 298 K) spectrum of **3o'**.

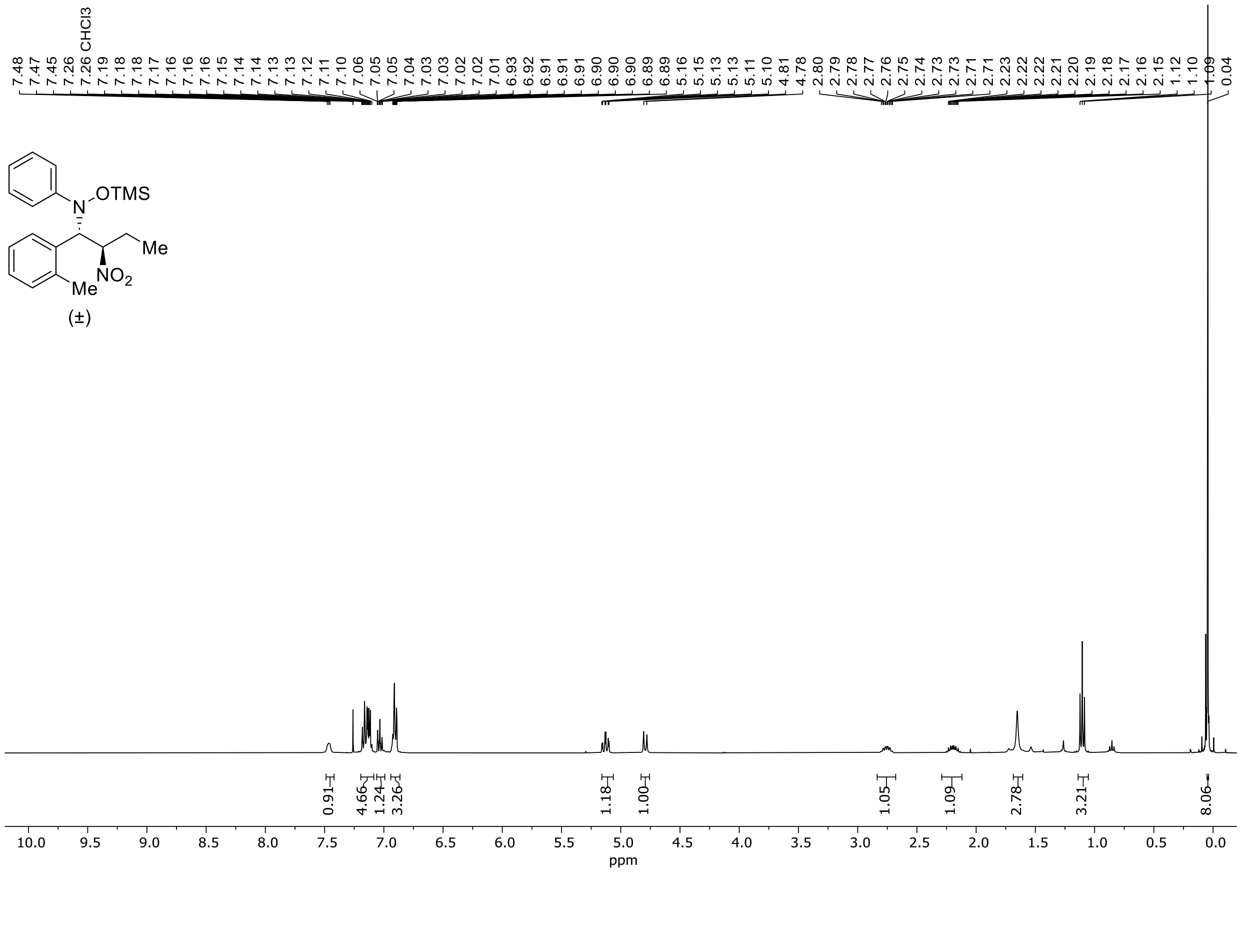

Figure S87. ^{13}C-NMR (101 MHz, $CDCl_3$, 298 K) spectrum of **3o’**.

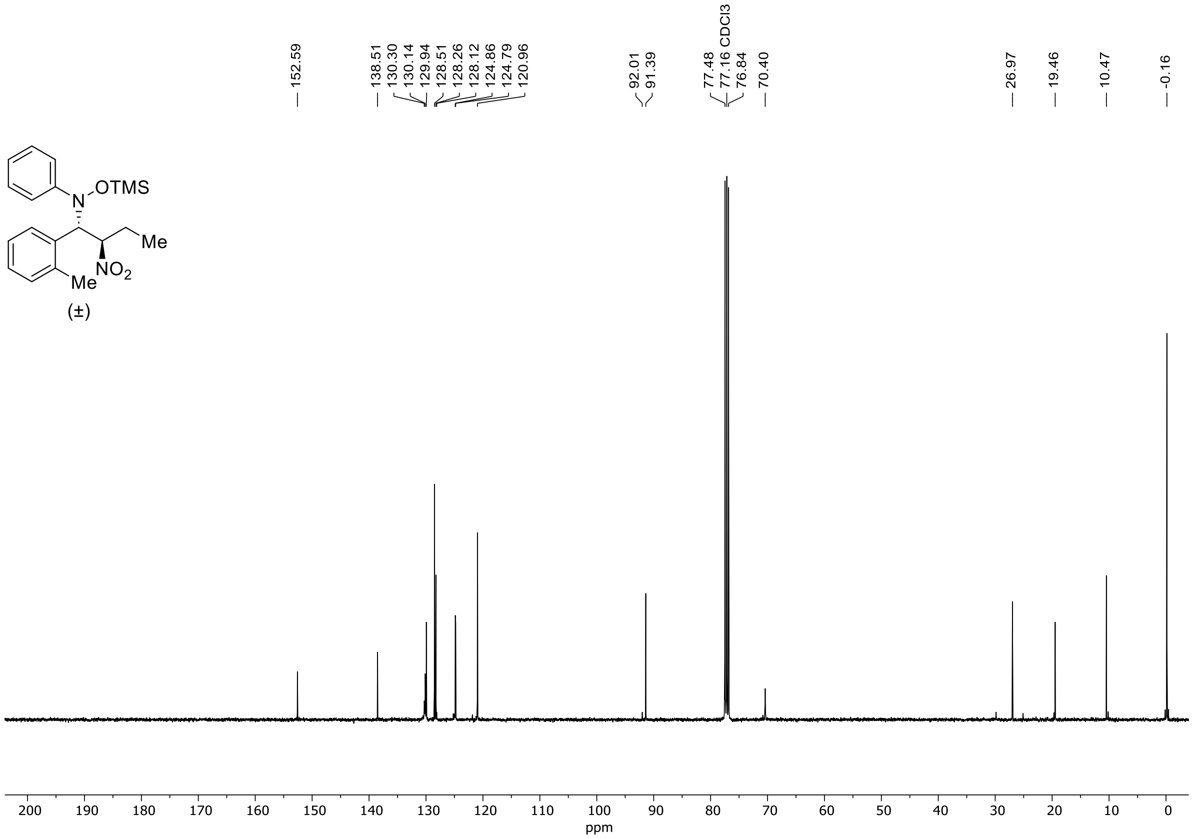

Figure S88. ^{1}H-NMR (400 MHz, $CDCl_3$, 298 K) spectrum of **3p**.

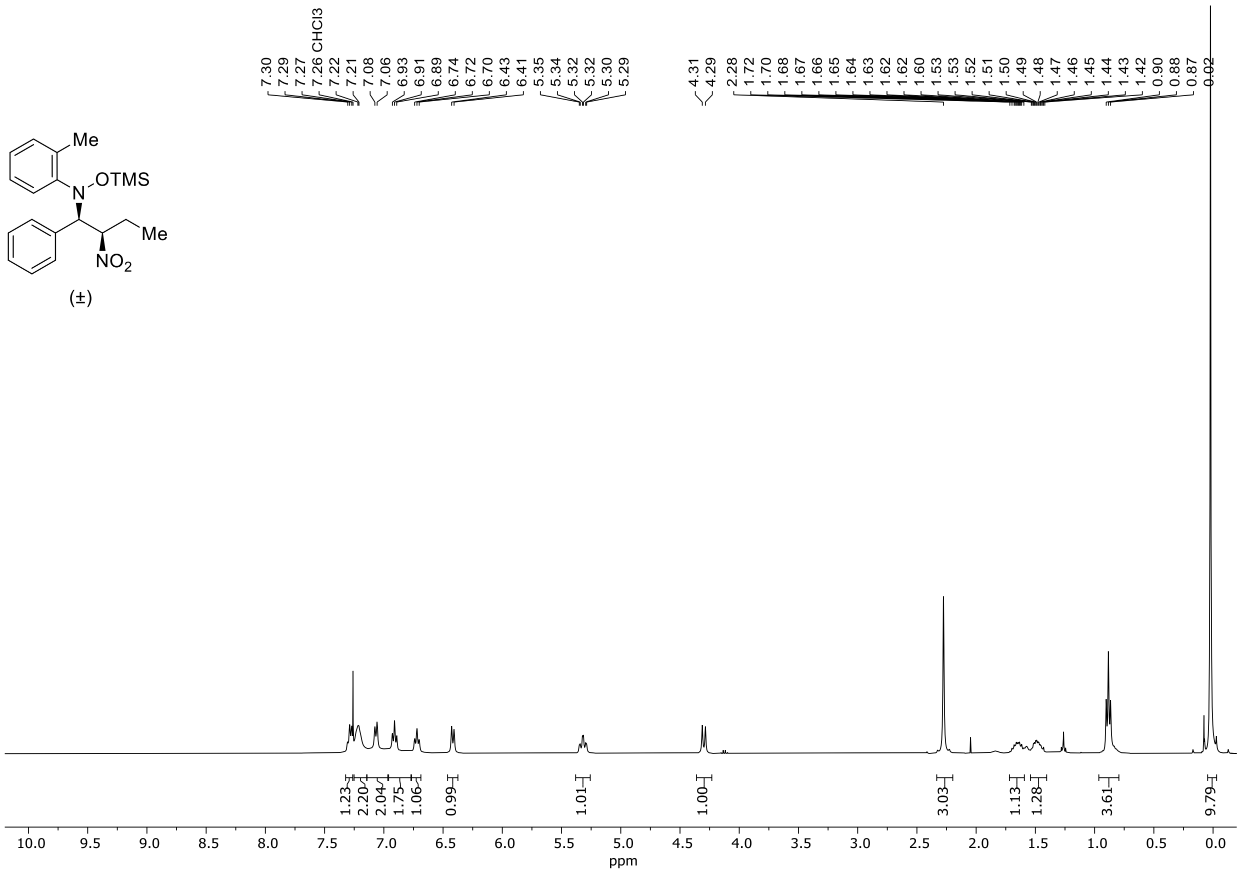

Figure S89. ^{13}C-NMR (101 MHz, $CDCl_3$, 298 K) spectrum of **3p**.

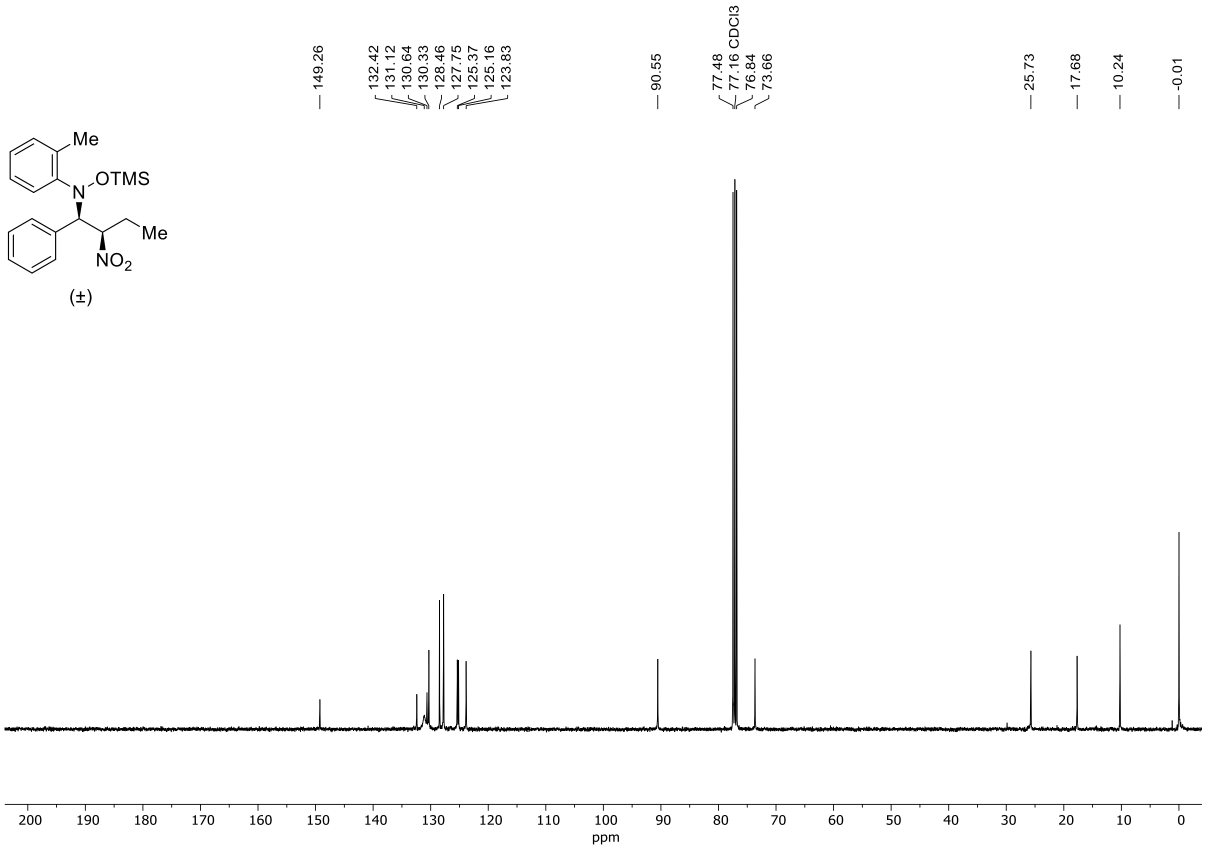

Figure S90. ^{1}H-NMR (400 MHz, $CDCl_3$, 298 K) spectrum of **3q**.

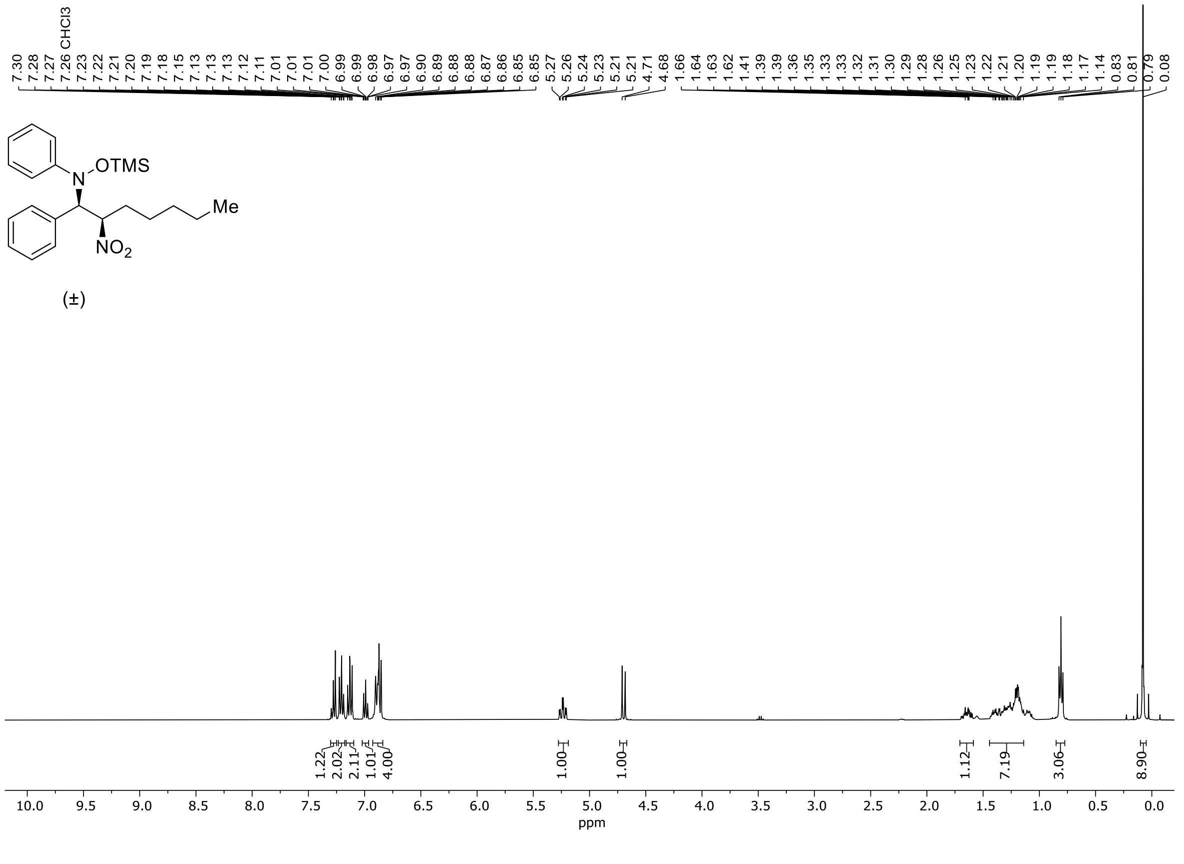

Figure S91. ^{13}C-NMR (101 MHz, $CDCl_3$, 298 K) spectrum of **3q**.

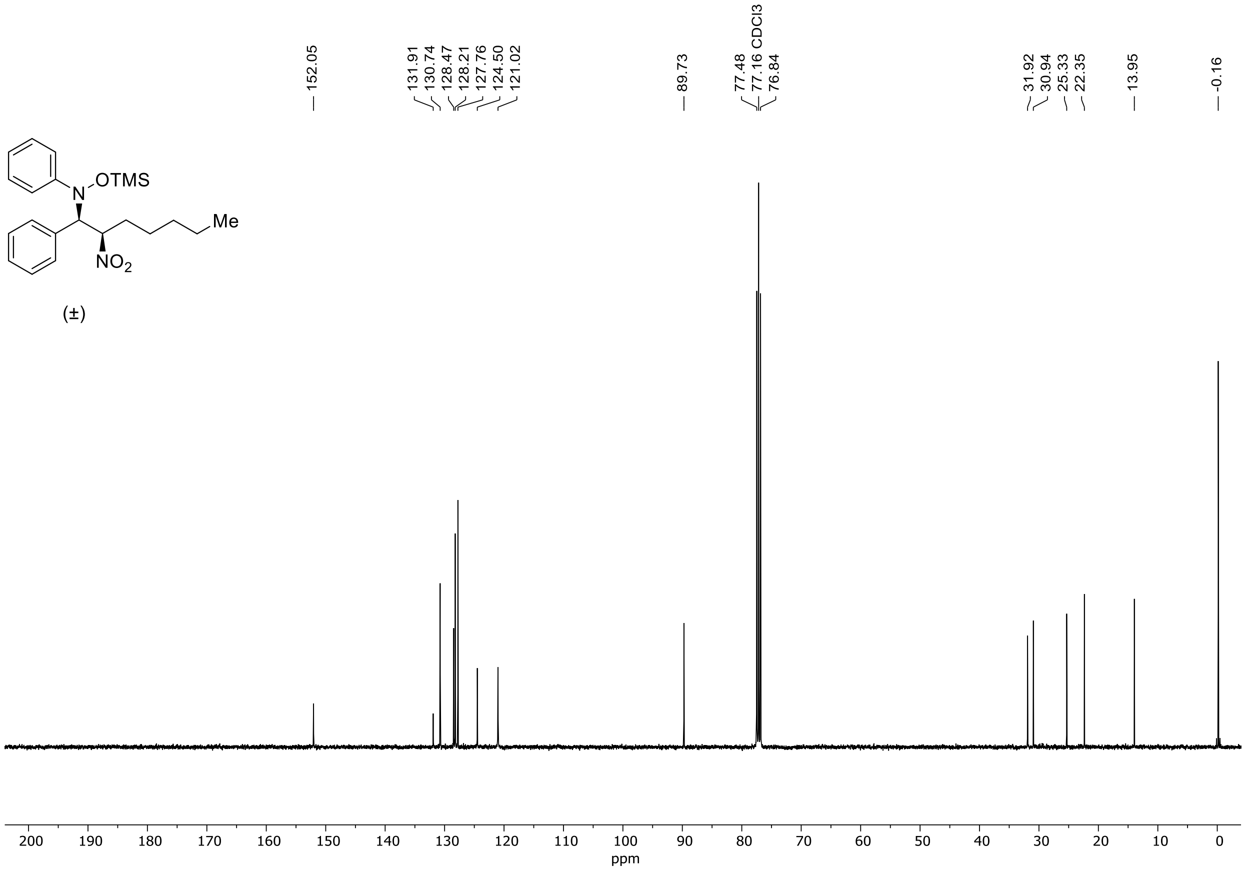

Figure S92. ^{1}H-NMR (400 MHz, $CDCl_3$, 298 K) spectrum of **3q'**.

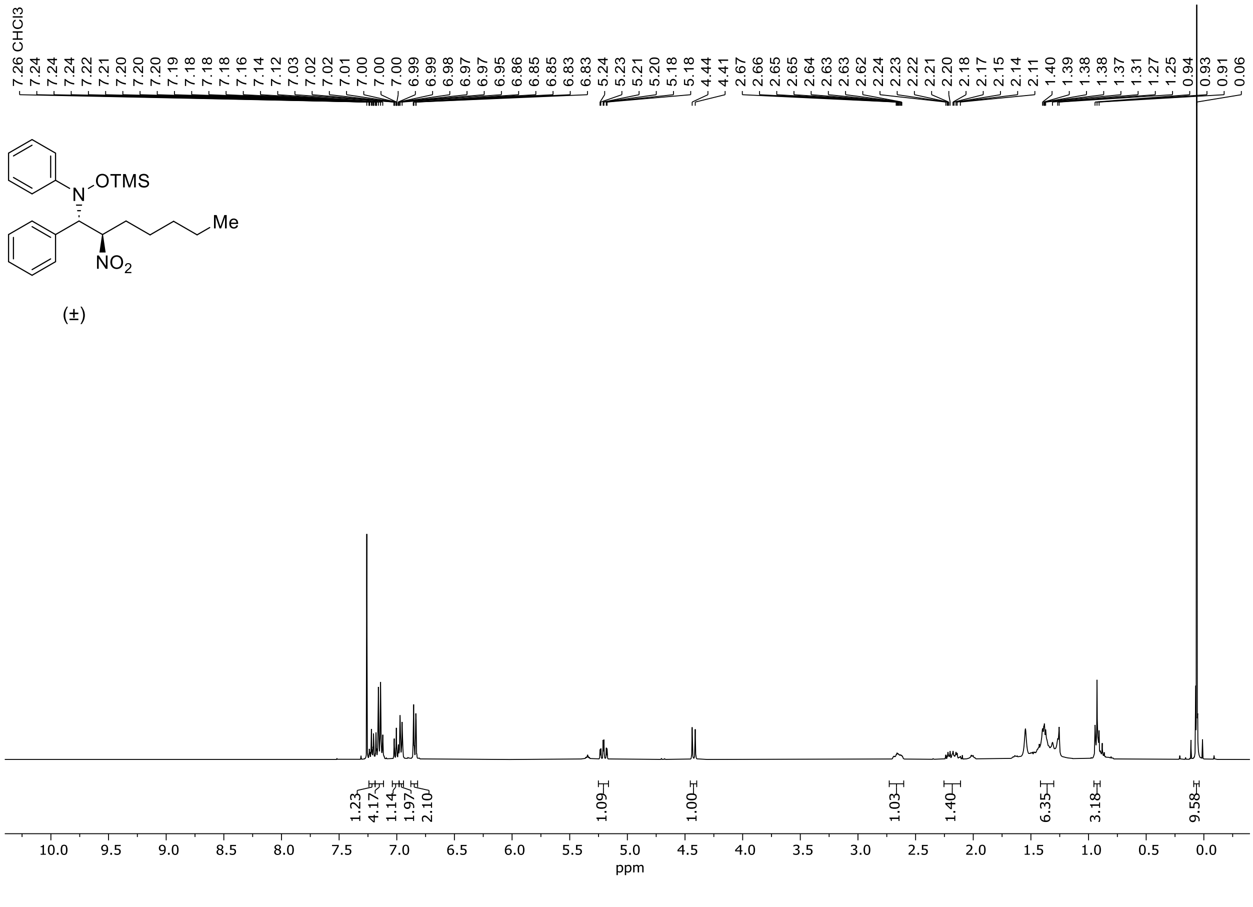

Figure S93. ^{13}C-NMR (101 MHz, $CDCl_3$, 298 K) spectrum of **3q'**.

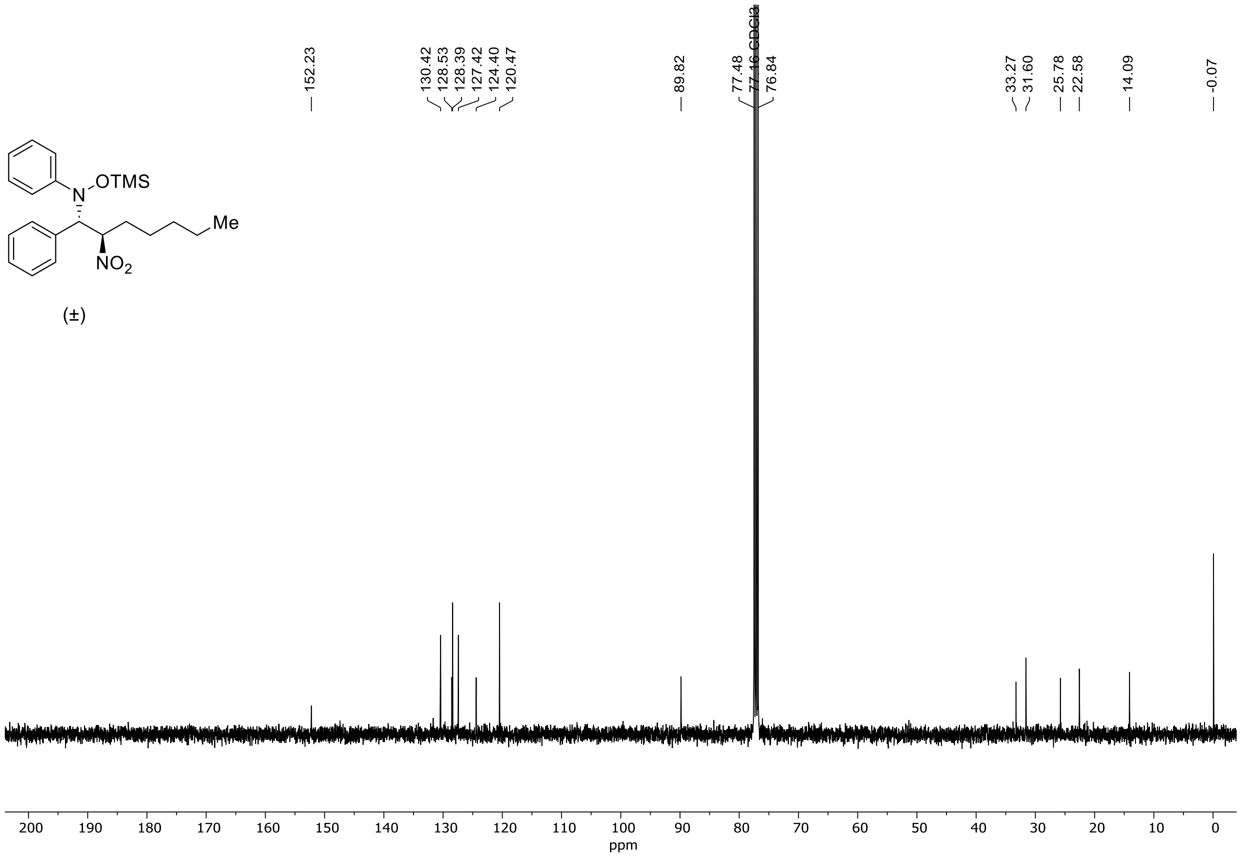

Figure S94. ^{1}H-NMR (400 MHz, $CDCl_3$, 298 K) spectrum of **3r**.

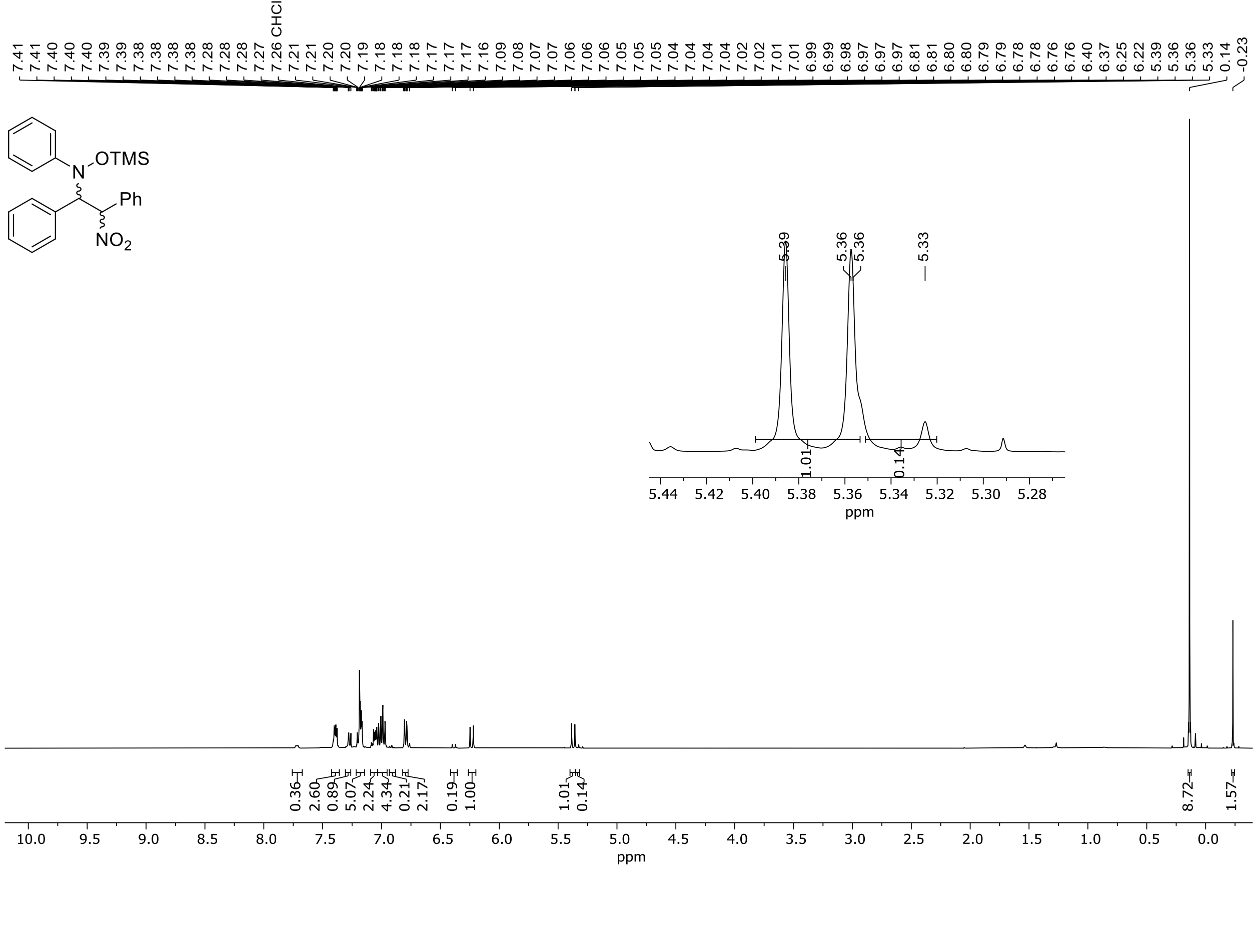

Figure S95. ^{13}C-NMR (101 MHz, $CDCl_3$, 298 K) spectrum of **3r**.

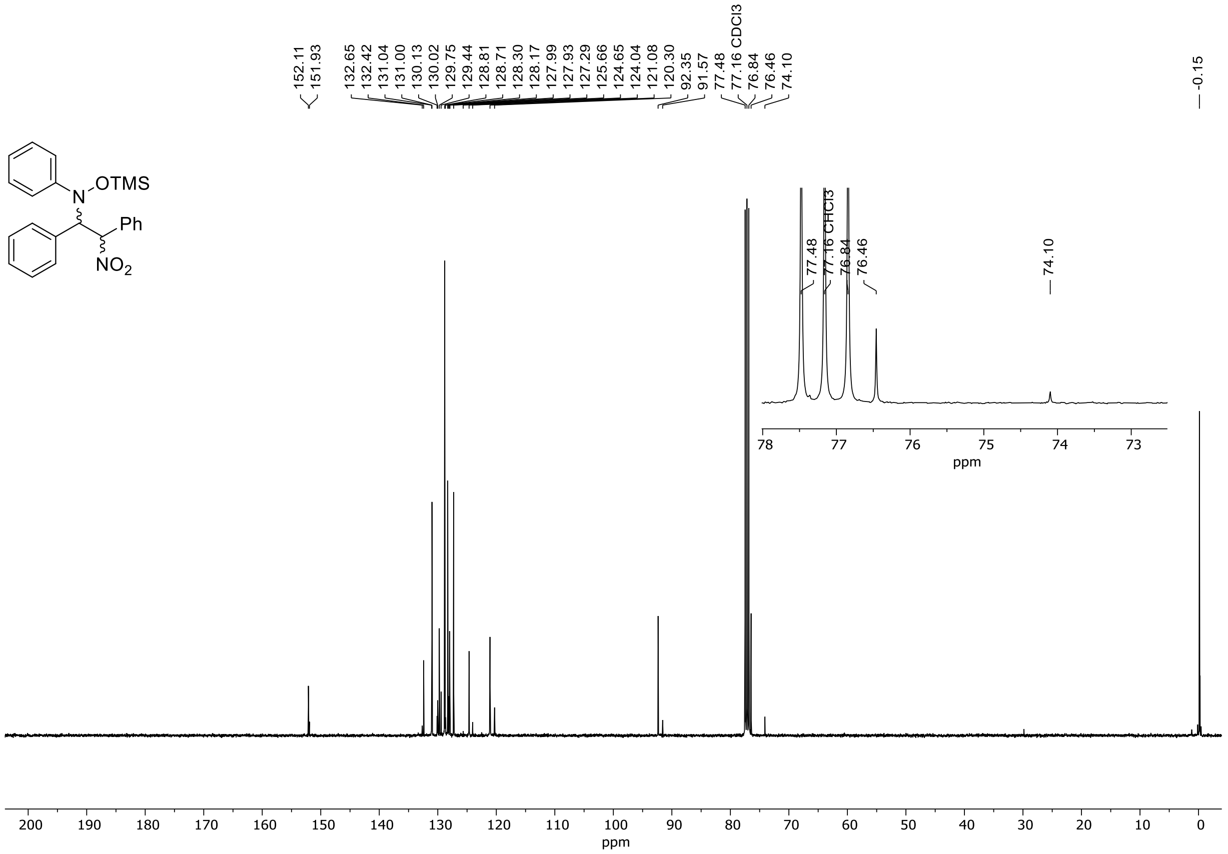

Figure S96. ^{1}H-NMR (400 MHz, $CDCl_3$, 298 K) spectrum of **3s**.

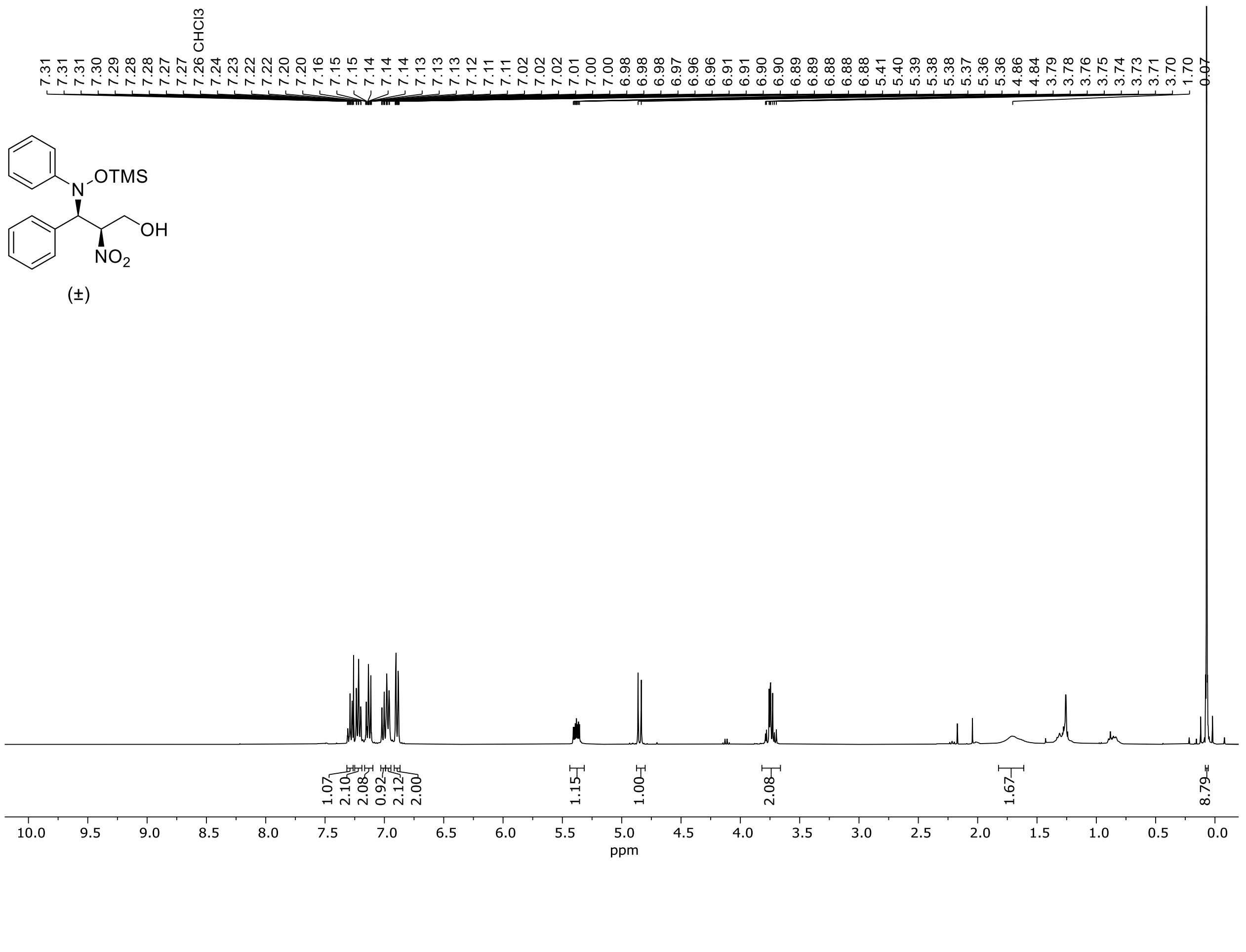

Figure S97. ^{13}C-NMR (101 MHz, $CDCl_3$, 298 K) spectrum of **3s**.

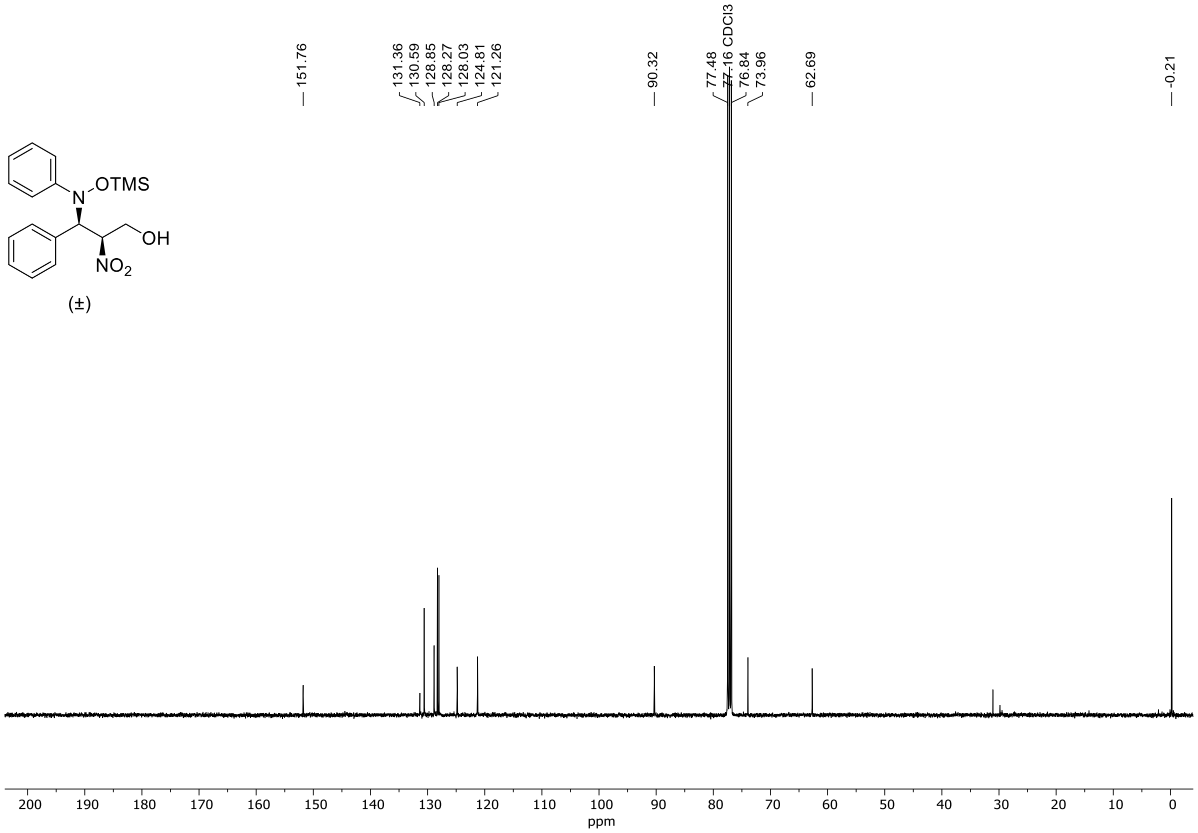

Figure S98. ^{1}H-NMR (400 MHz, $CDCl_3$, 298 K) spectrum of **3t**.

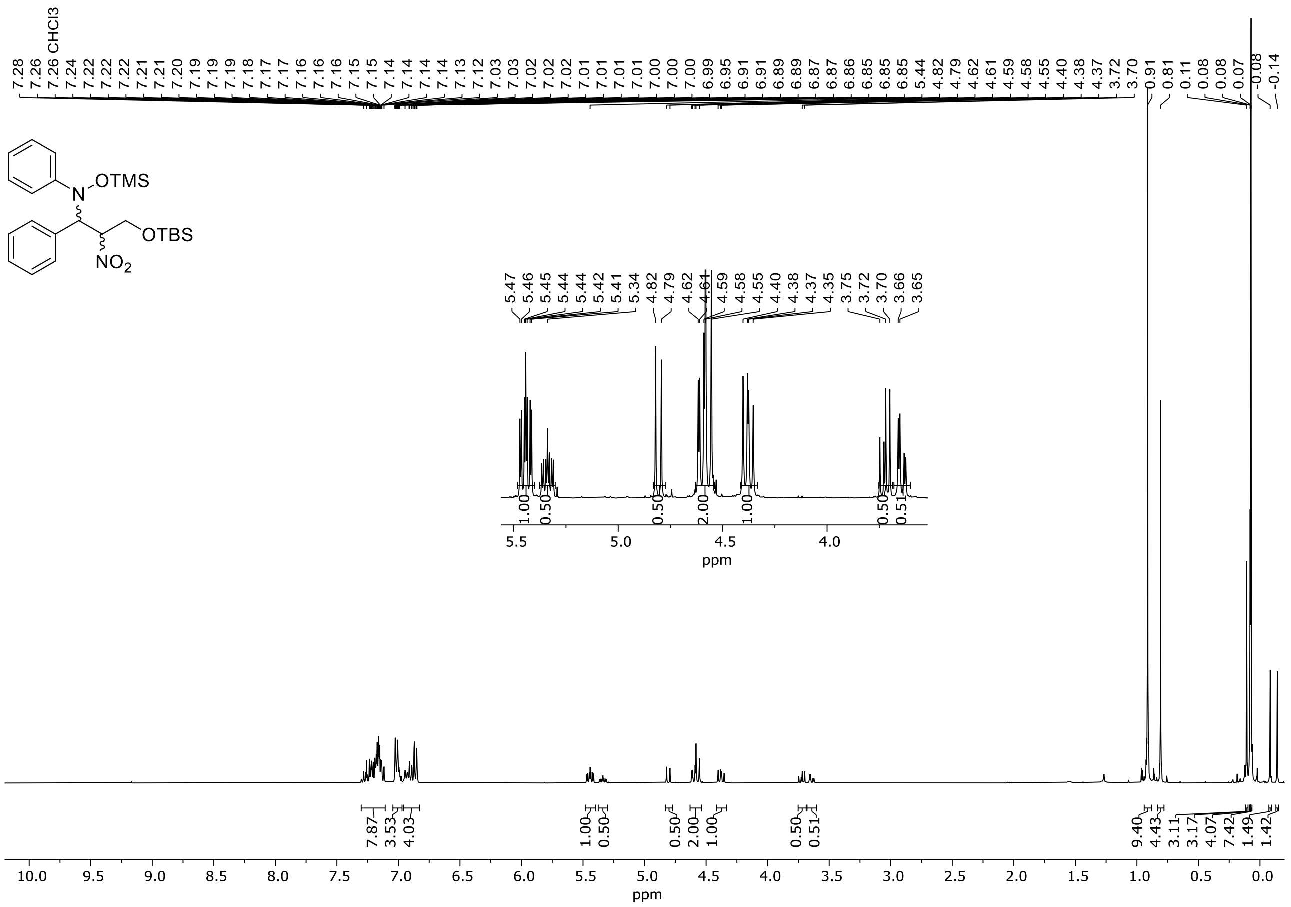

Figure S99. ^{13}C-NMR (101 MHz, $CDCl_3$, 298 K) spectrum of **3t**.

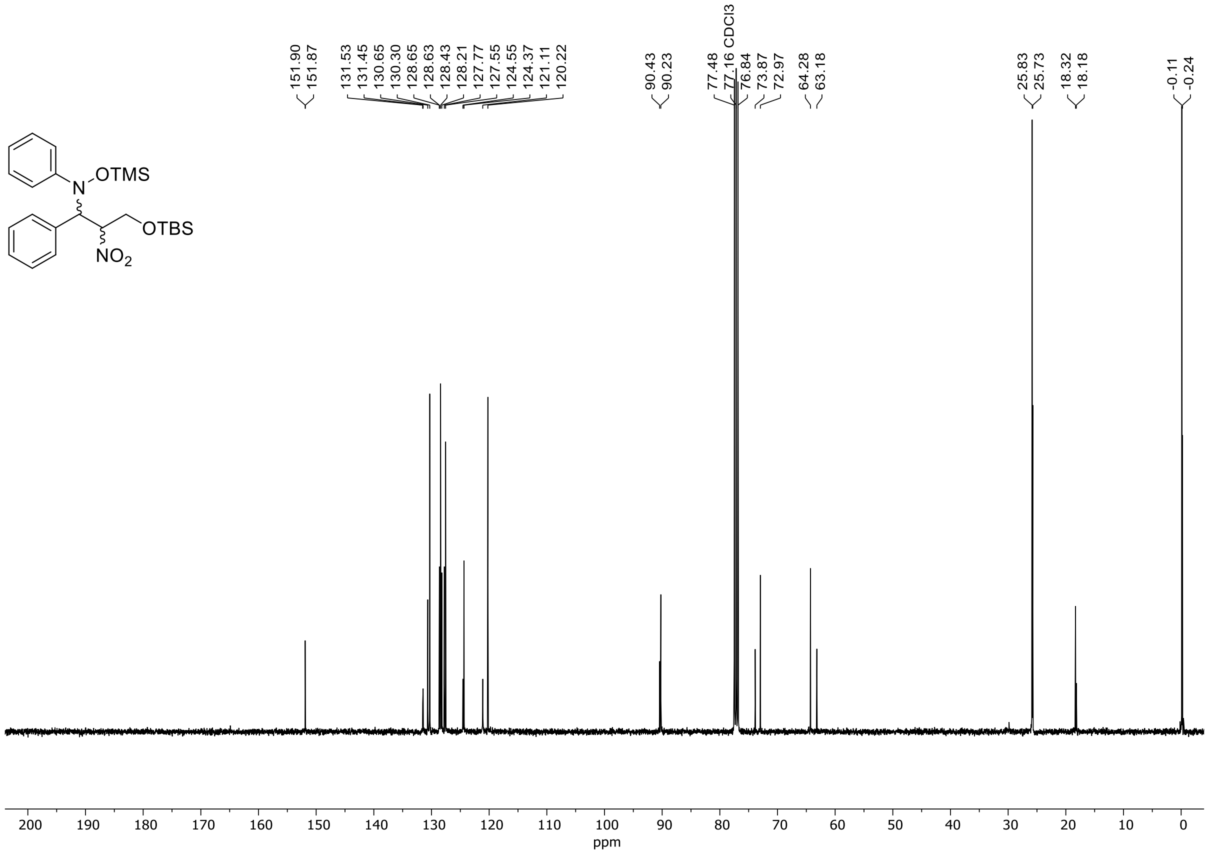

Figure S100. ^{1}H-NMR (400 MHz, $CDCl_3$, 298 K) spectrum of **3u**.

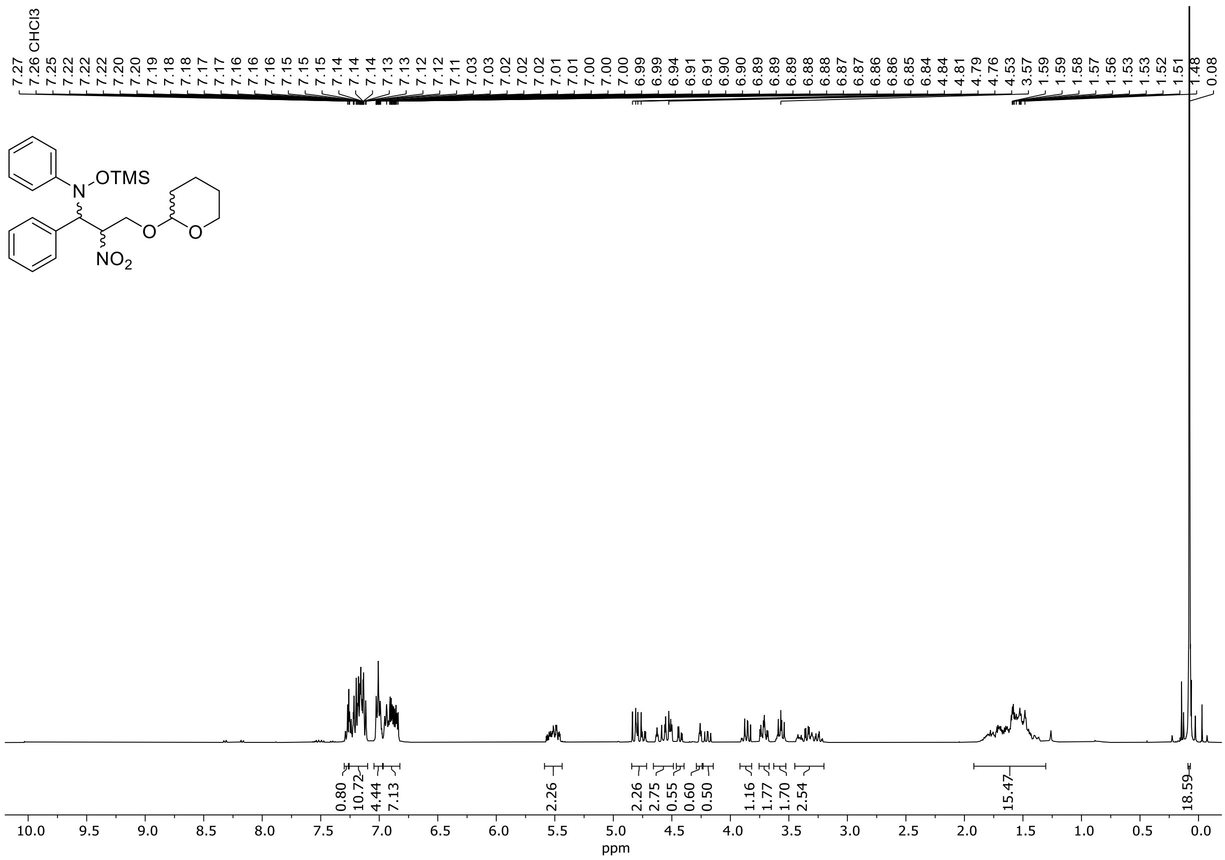

Figure S101. ^{13}C-NMR (101 MHz, $CDCl_3$, 298 K) spectrum of **3u**.

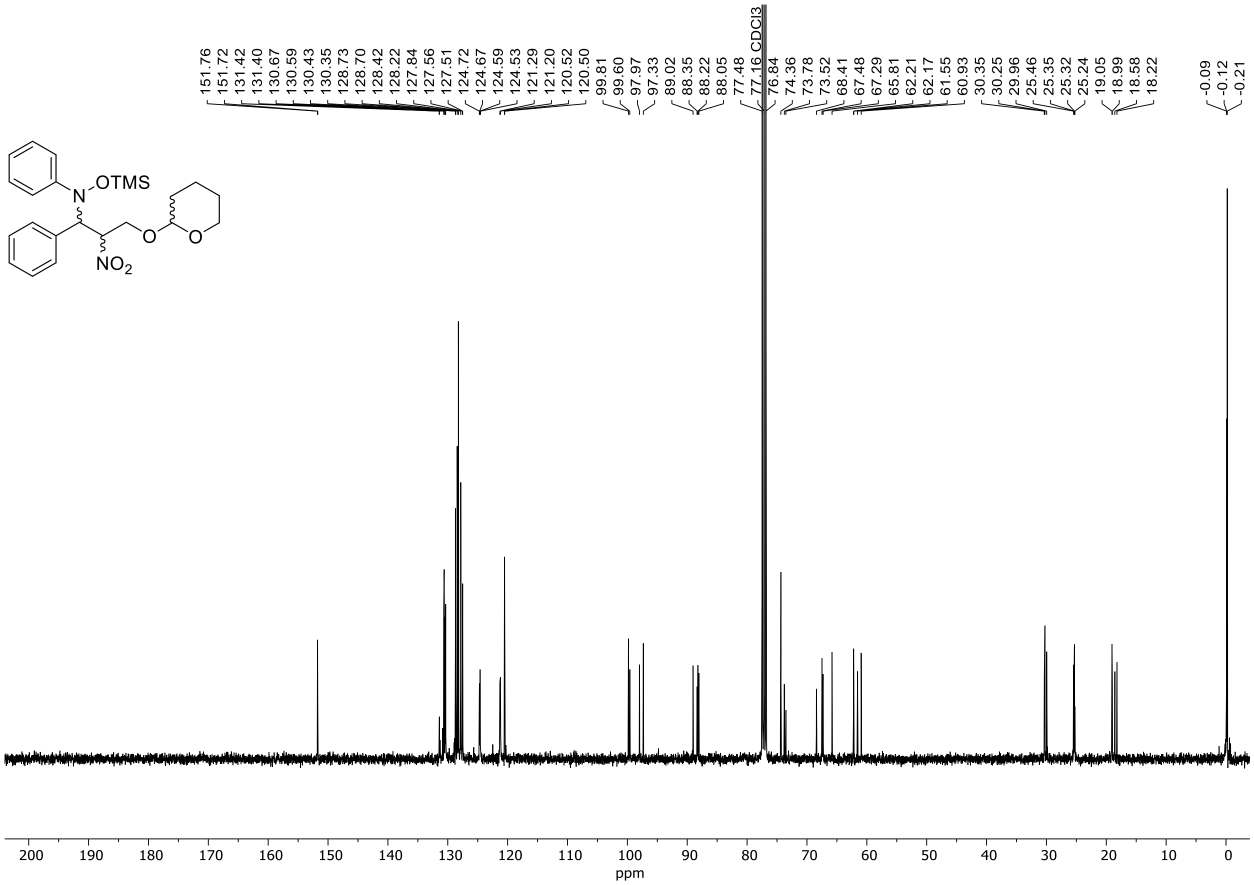

Figure S102. ^{1}H-NMR (400 MHz, $CDCl_3$, 298 K) spectrum of **3v**.

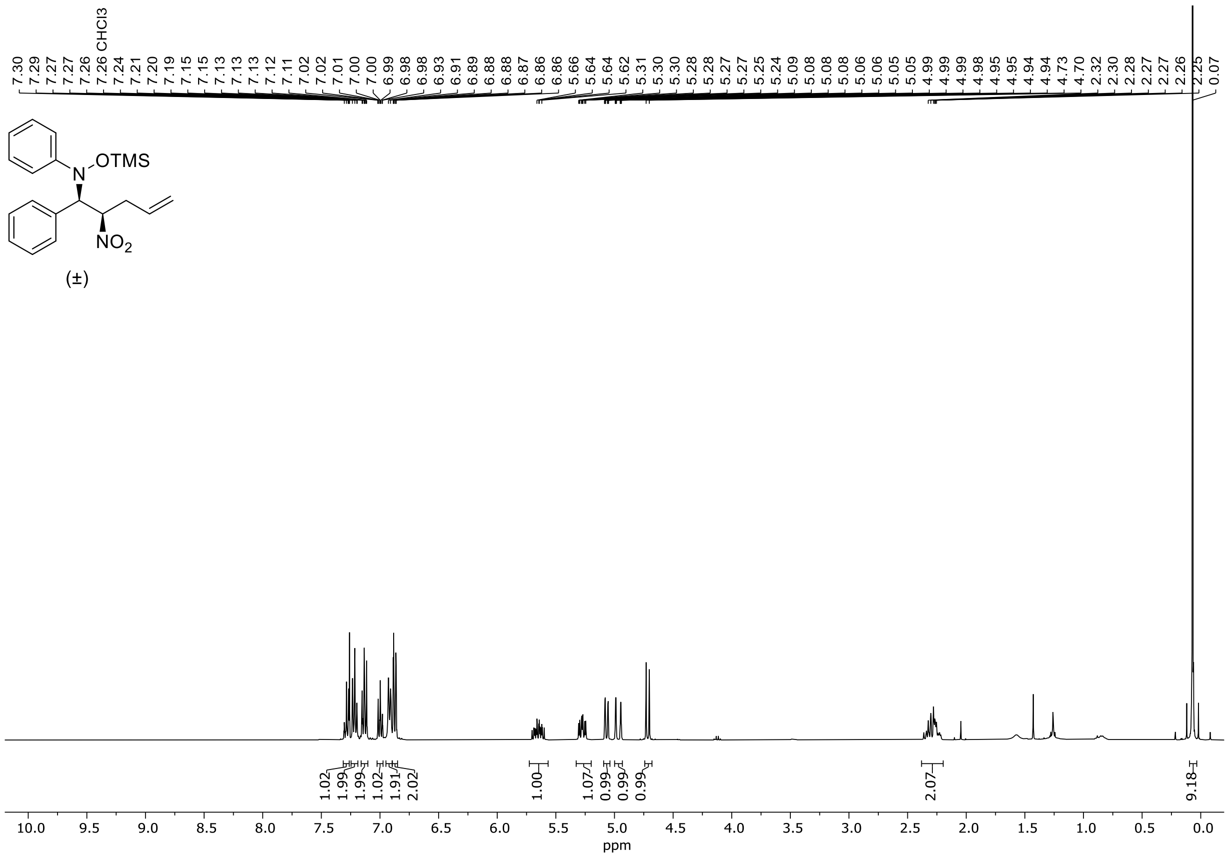

Figure S103. ^{13}C-NMR (101 MHz, $CDCl_3$, 298 K) spectrum of **3v**.

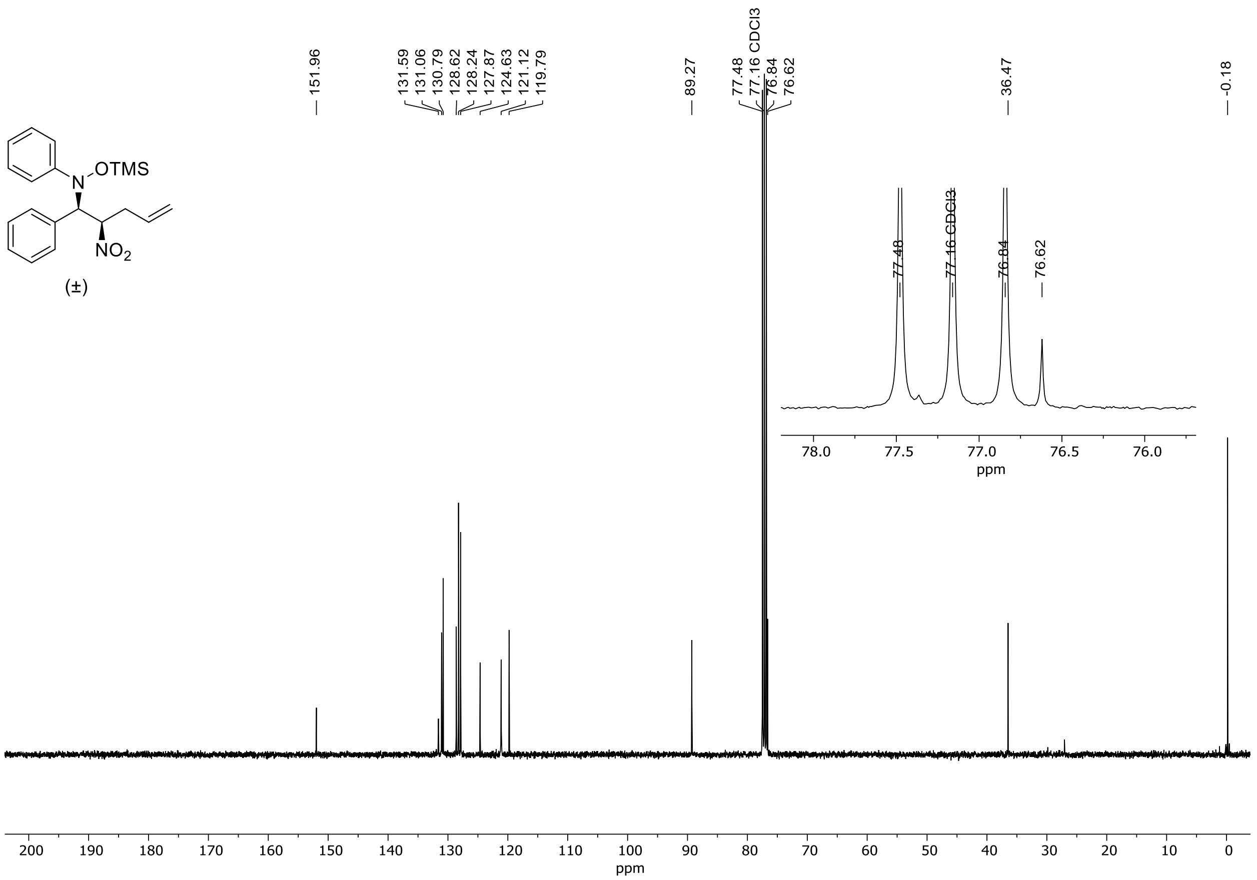

Figure S104. ^{1}H-NMR (400 MHz, $CDCl_3$, 298 K) spectrum of **3v'**.

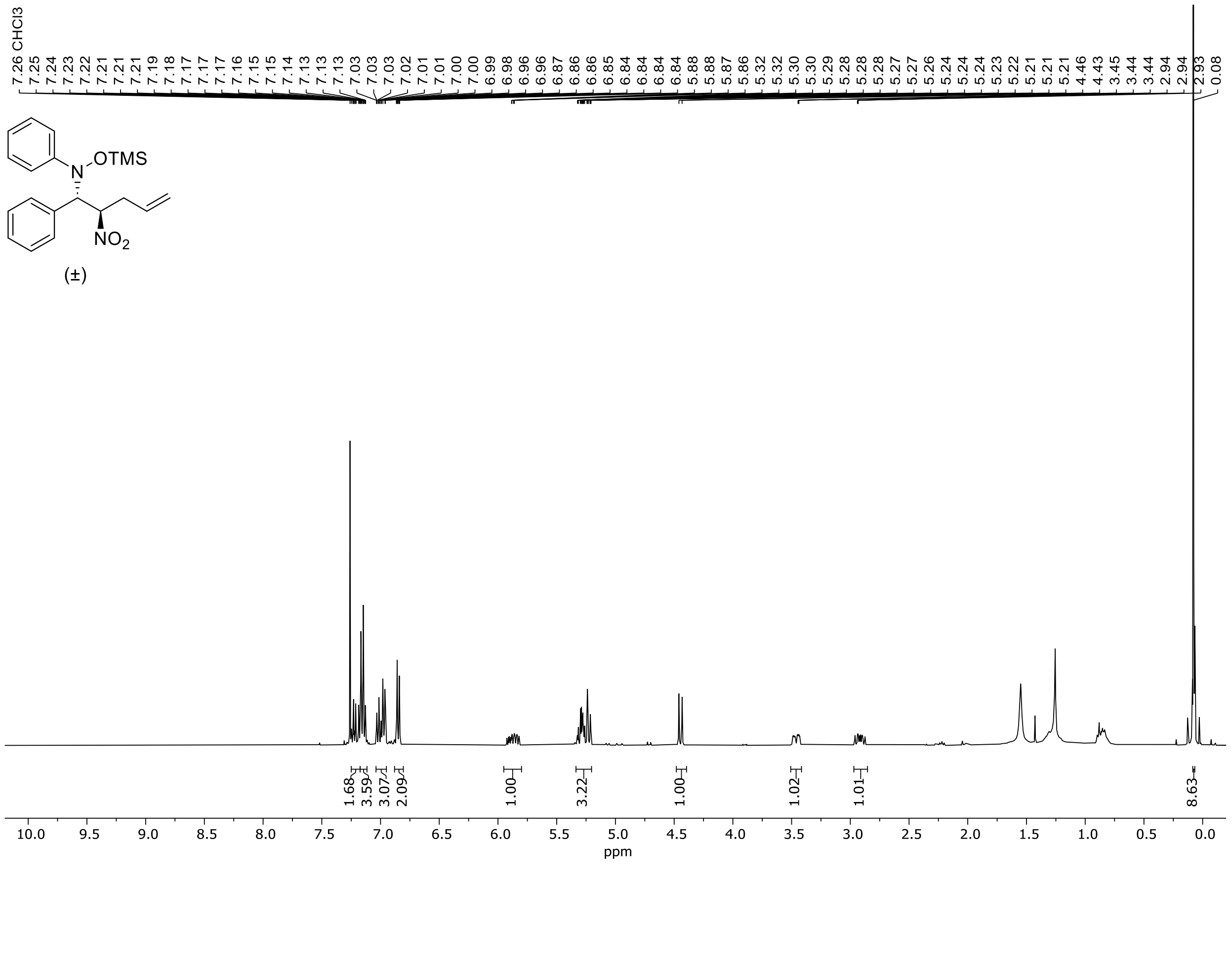

Figure S105. ^{13}C-NMR (101 MHz, $CDCl_3$, 298 K) spectrum of **3v'**.

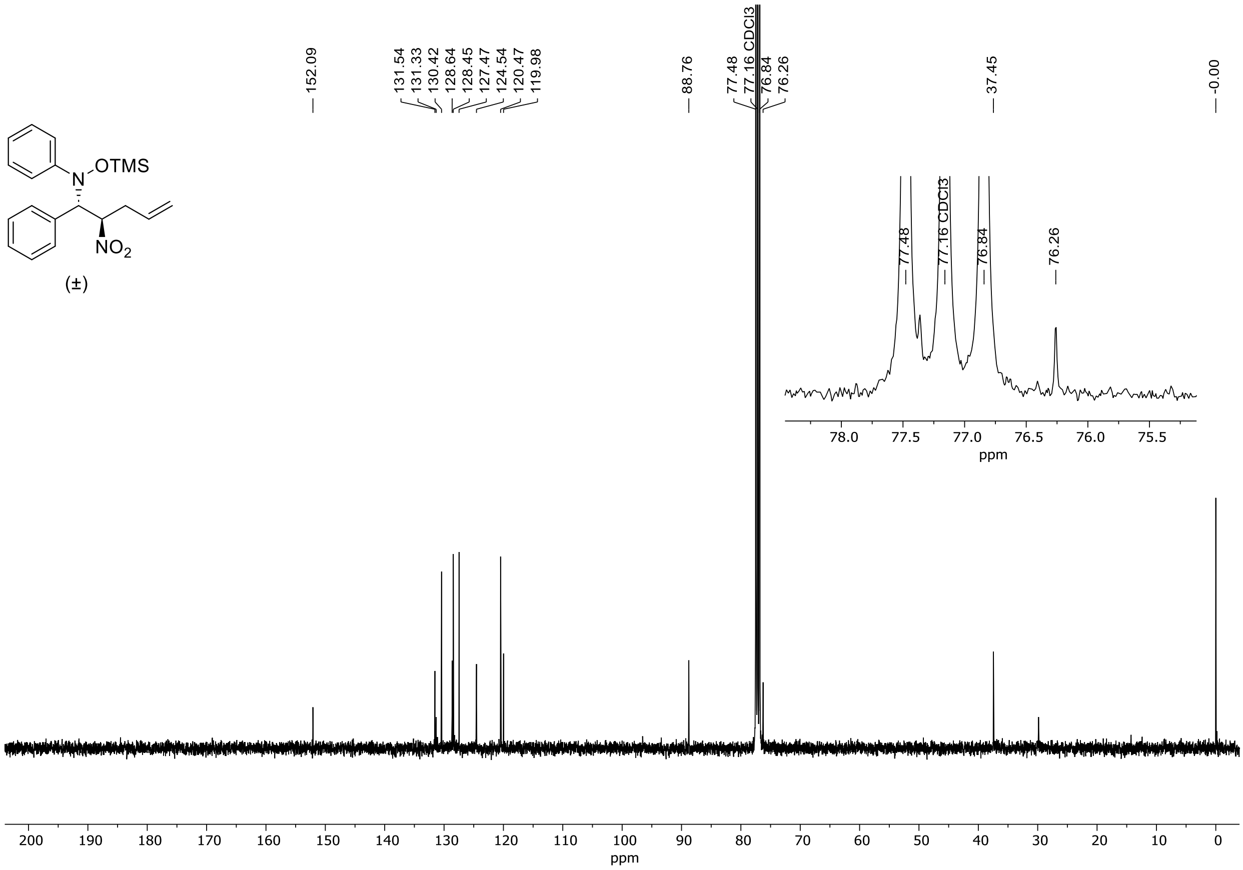

Figure S106. ^{1}H-NMR (400 MHz, $CDCl_3$, 298 K) spectrum of **3w**.

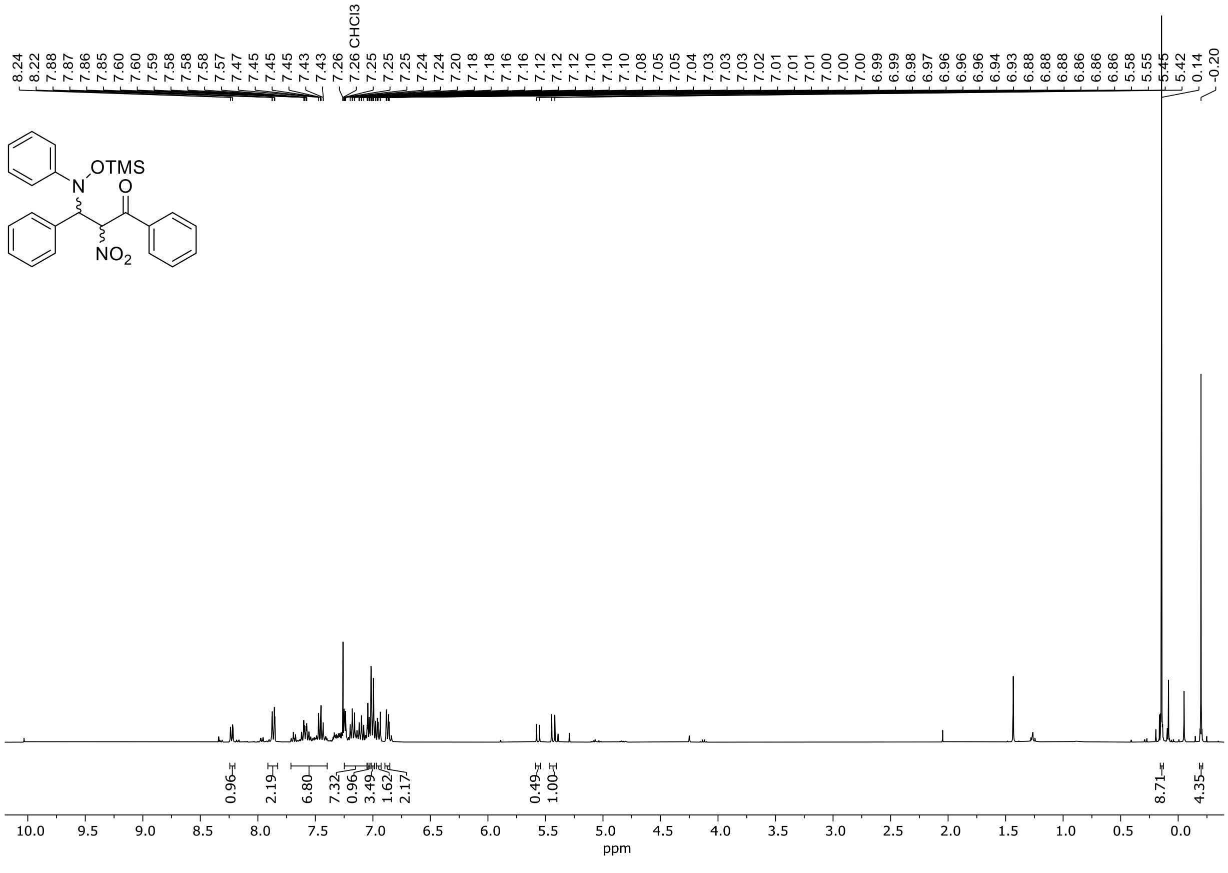

Figure S107. ^{13}C-NMR (101 MHz, $CDCl_3$, 298 K) spectrum of **3w**.

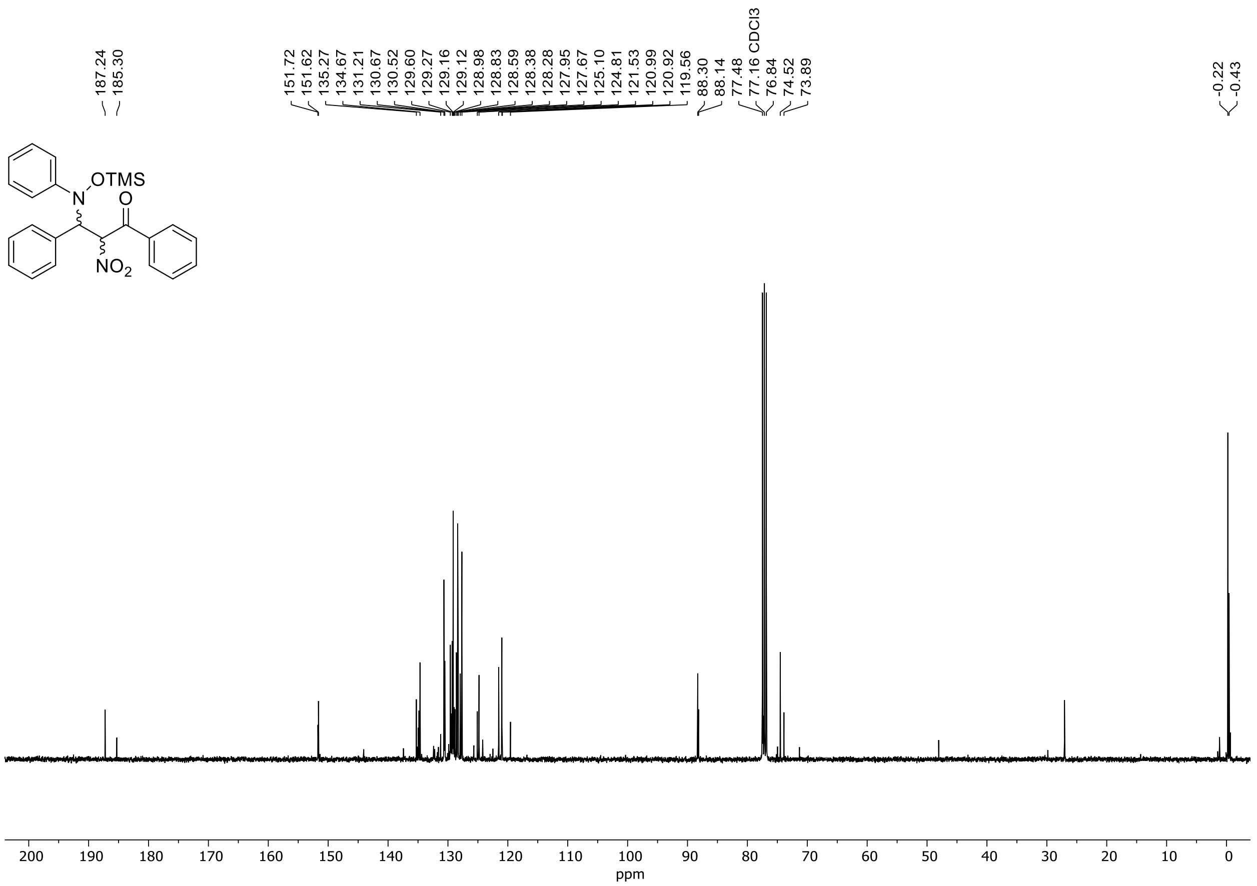

Figure S108. ^{1}H-NMR (400 MHz, $CDCl_3$, 298 K) spectrum of **3x**.

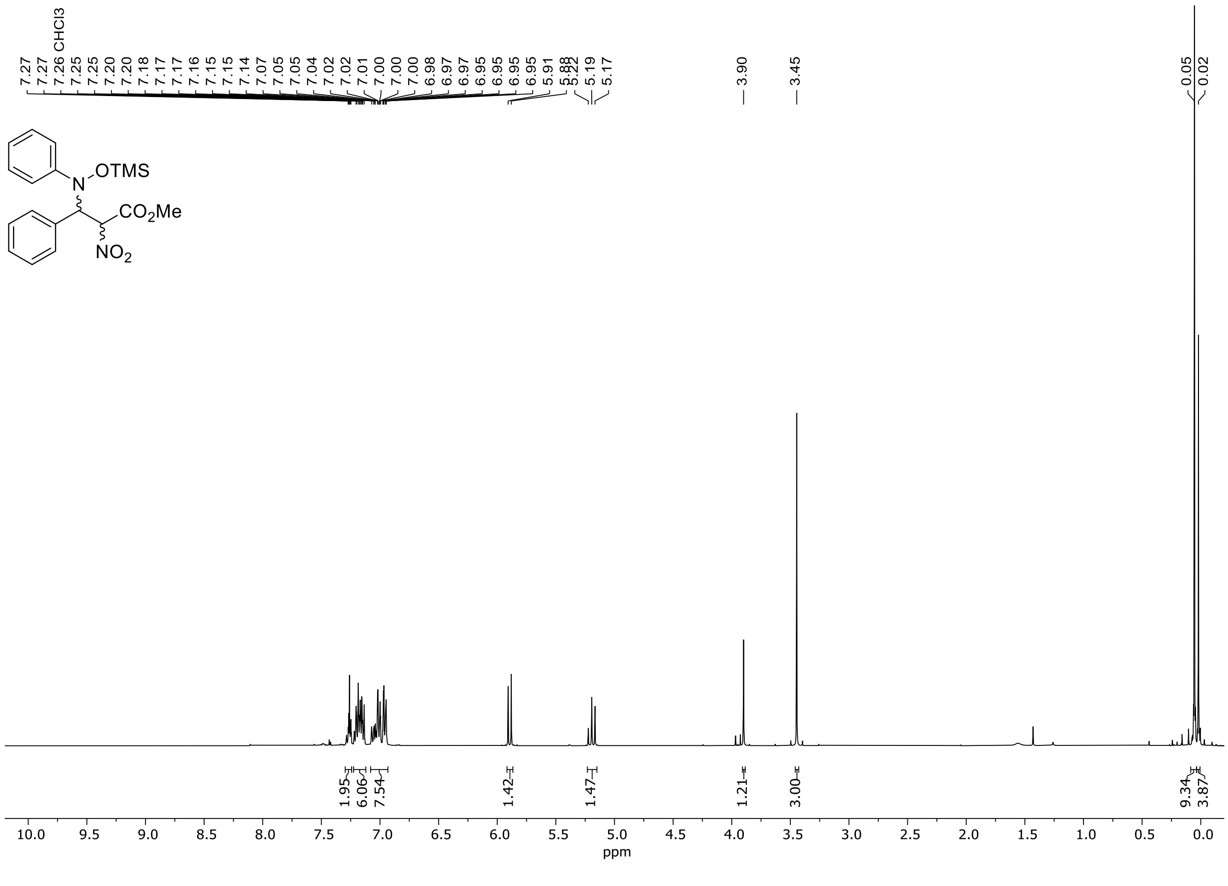

Figure S109. ^{13}C-NMR (101 MHz, $CDCl_3$, 298 K) spectrum of **3x**.

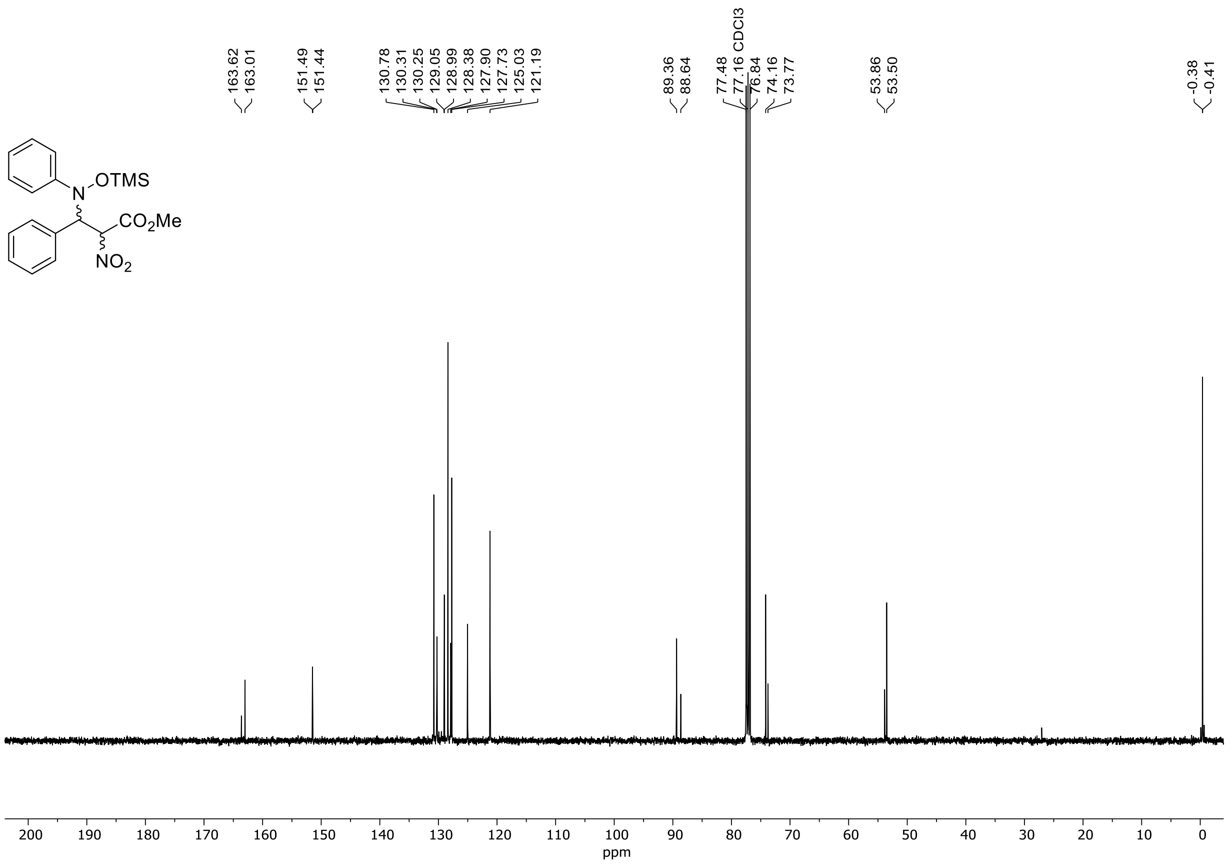

Figure S110. ^{1}H-NMR (400 MHz, $CDCl_3$, 298 K) spectrum of **3y**.

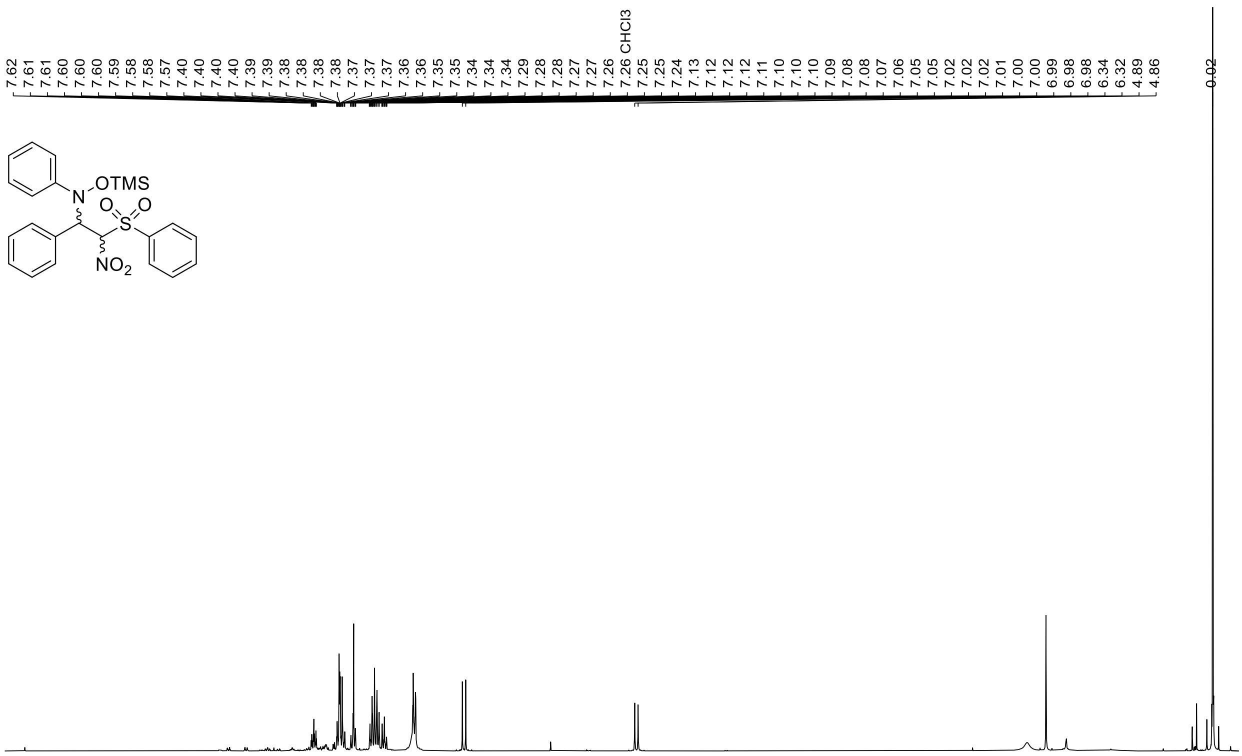

Figure S111. ^{13}C-NMR (101 MHz, $CDCl_3$, 298 K) spectrum of **3y**.

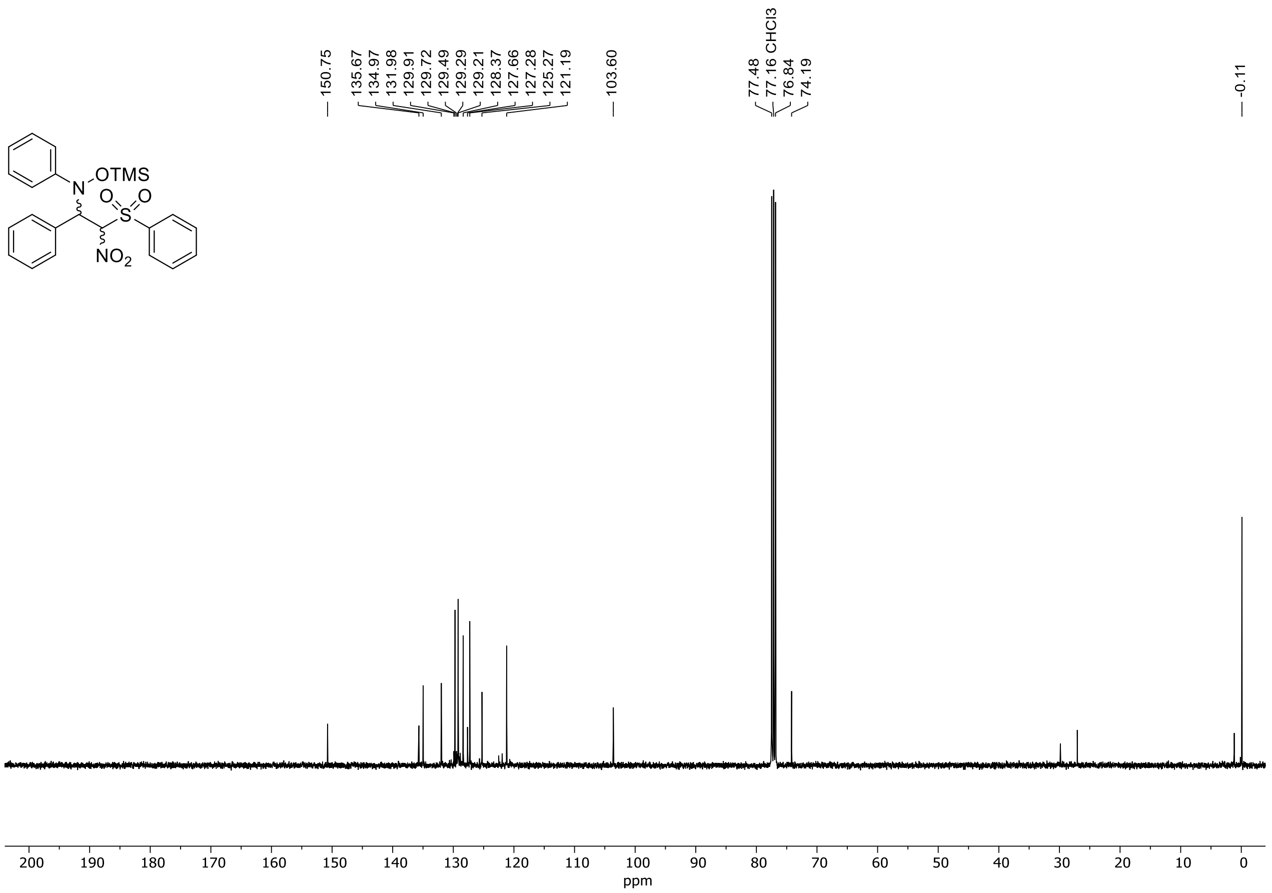

Figure S112. ^{1}H-NMR (400 MHz, $CDCl_3$, 298 K) spectrum of **3z**.

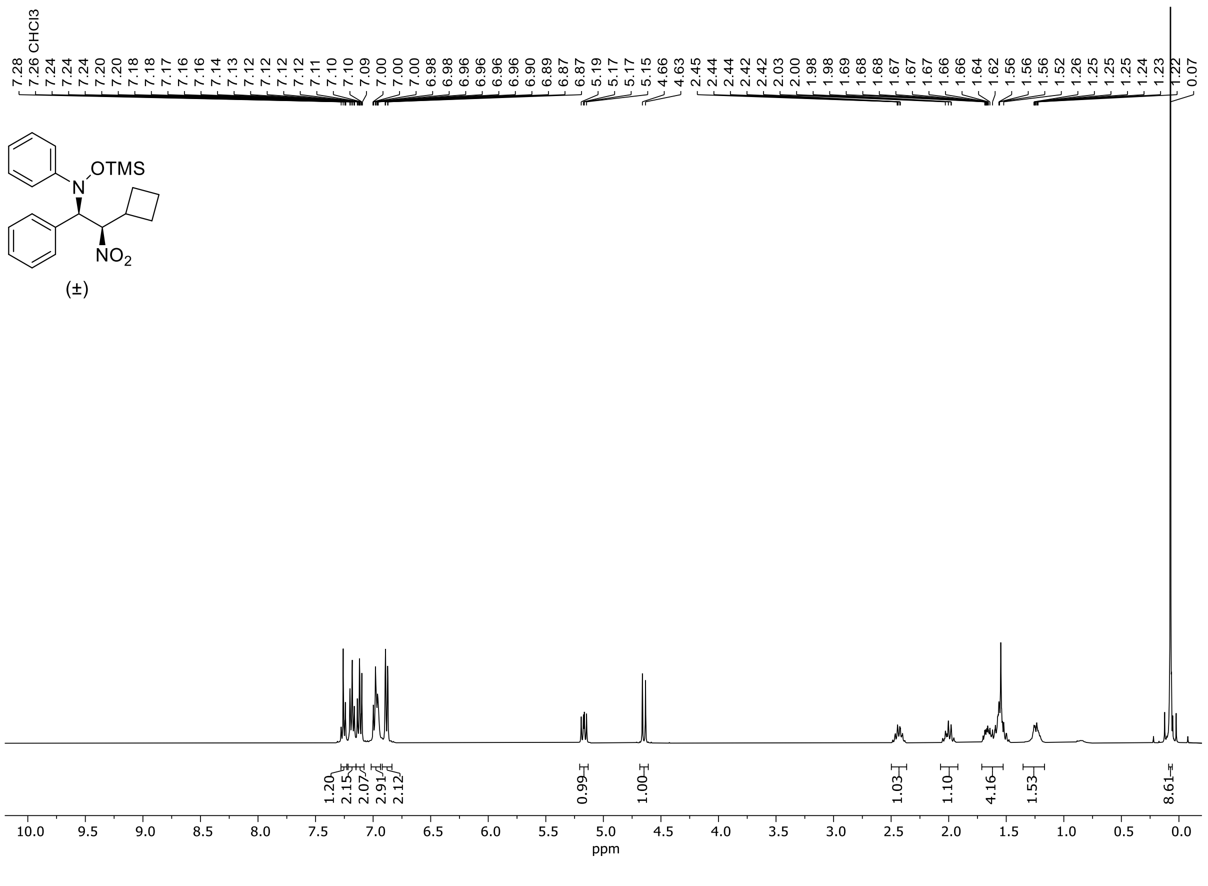

Figure S113. ^{13}C-NMR (101 MHz, $CDCl_3$, 298 K) spectrum of **3z**.

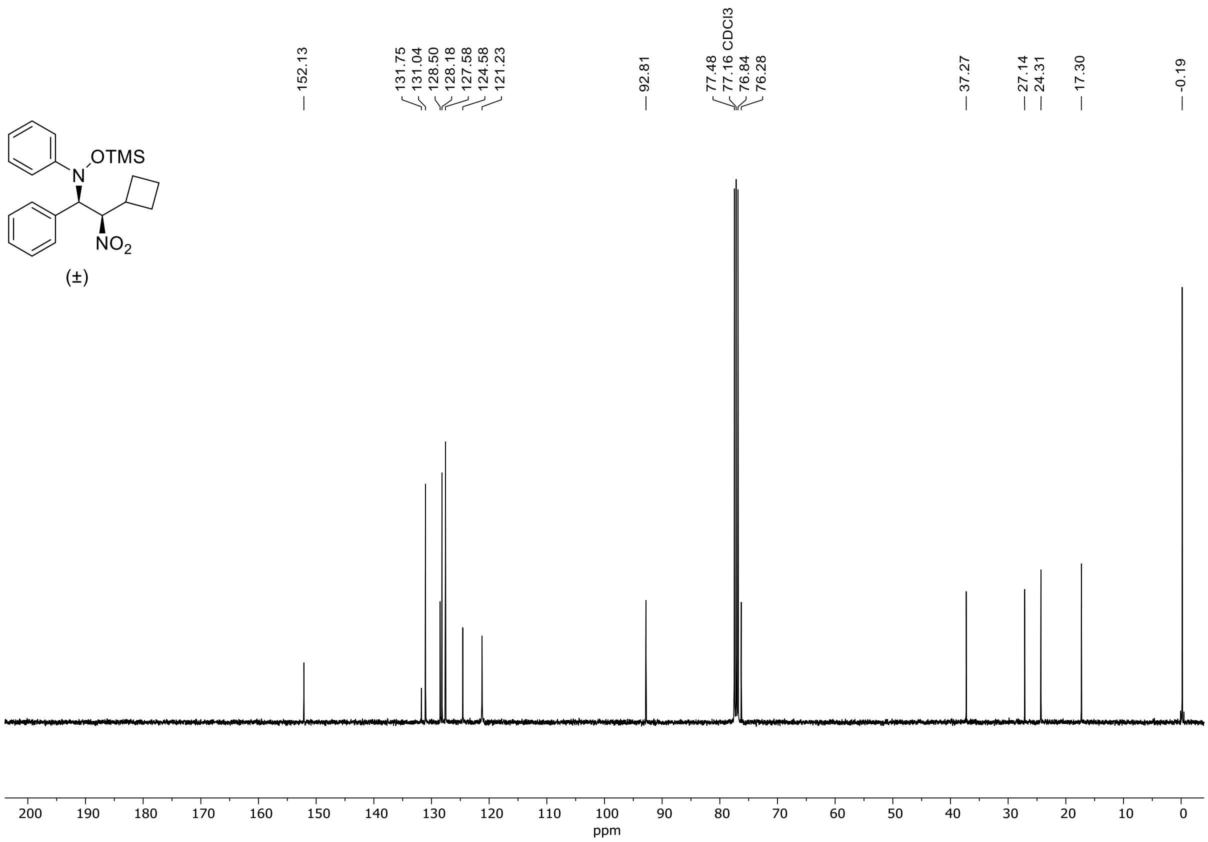

Figure S114. ^{1}H-NMR (400 MHz, $CDCl_3$, 298 K) spectrum of **3z'**.

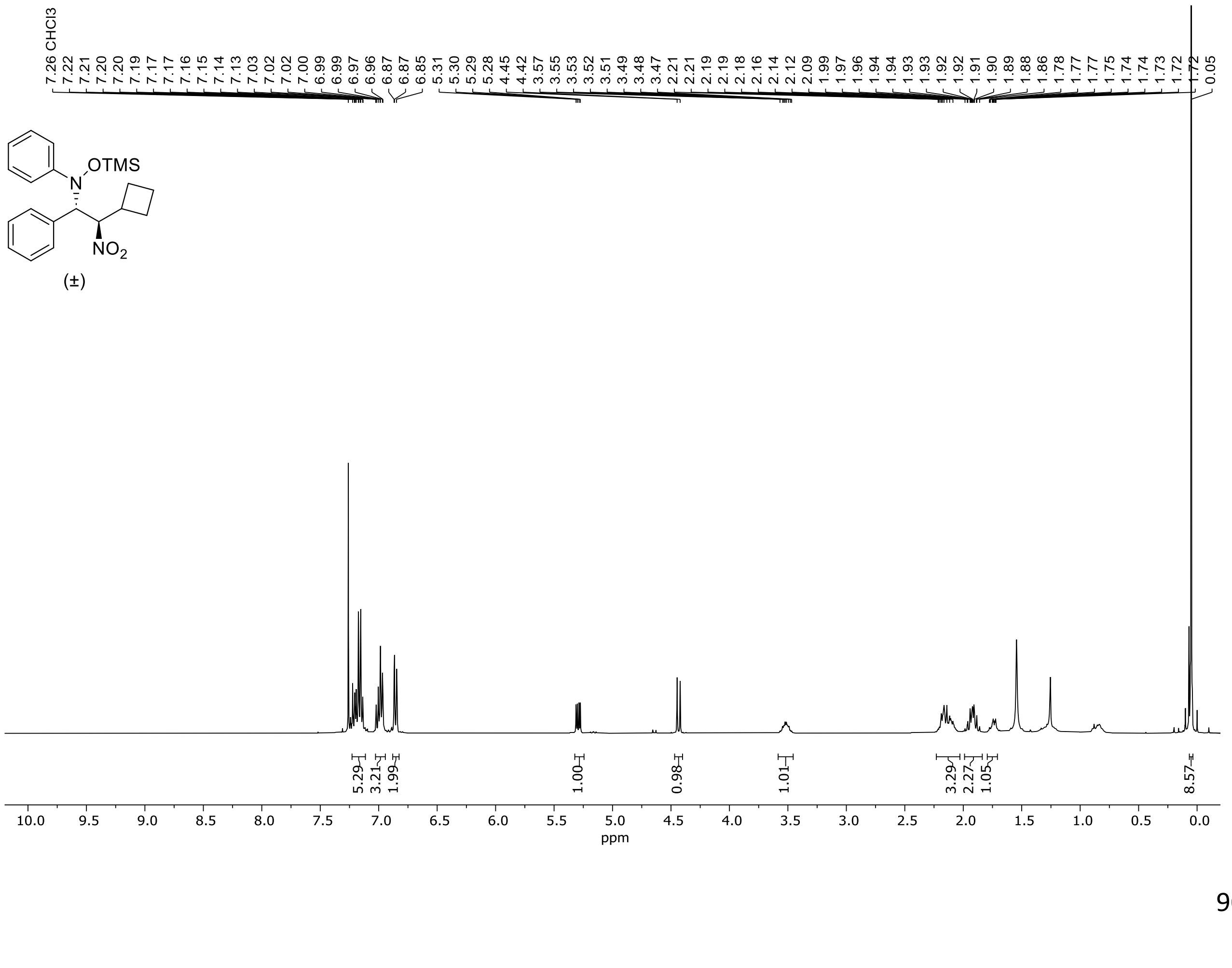

Figure S115. ^{13}C-NMR (101 MHz, $CDCl_3$, 298 K) spectrum of **3z'**.

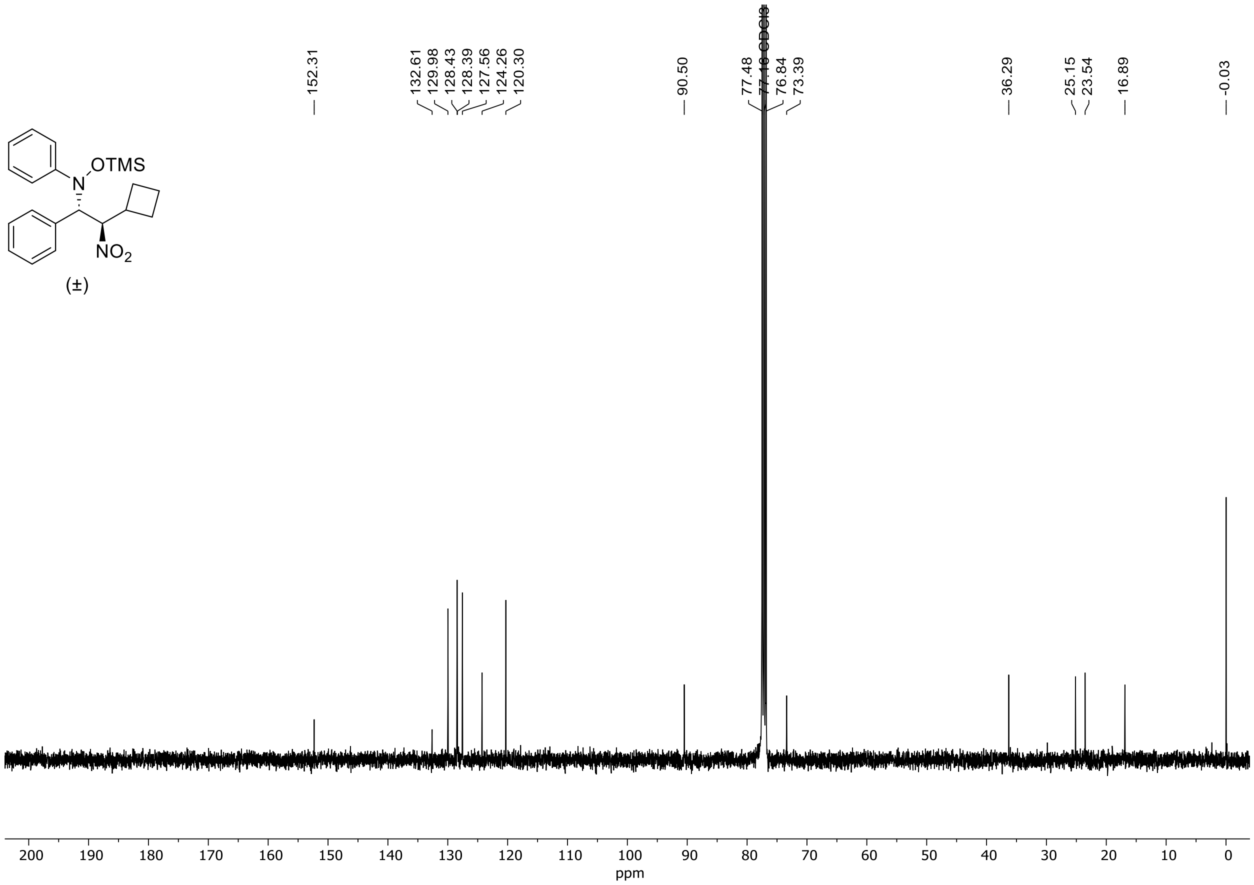

Figure S116. ^{1}H-NMR (400 MHz, $CDCl_3$, 298 K) spectrum of **4**.

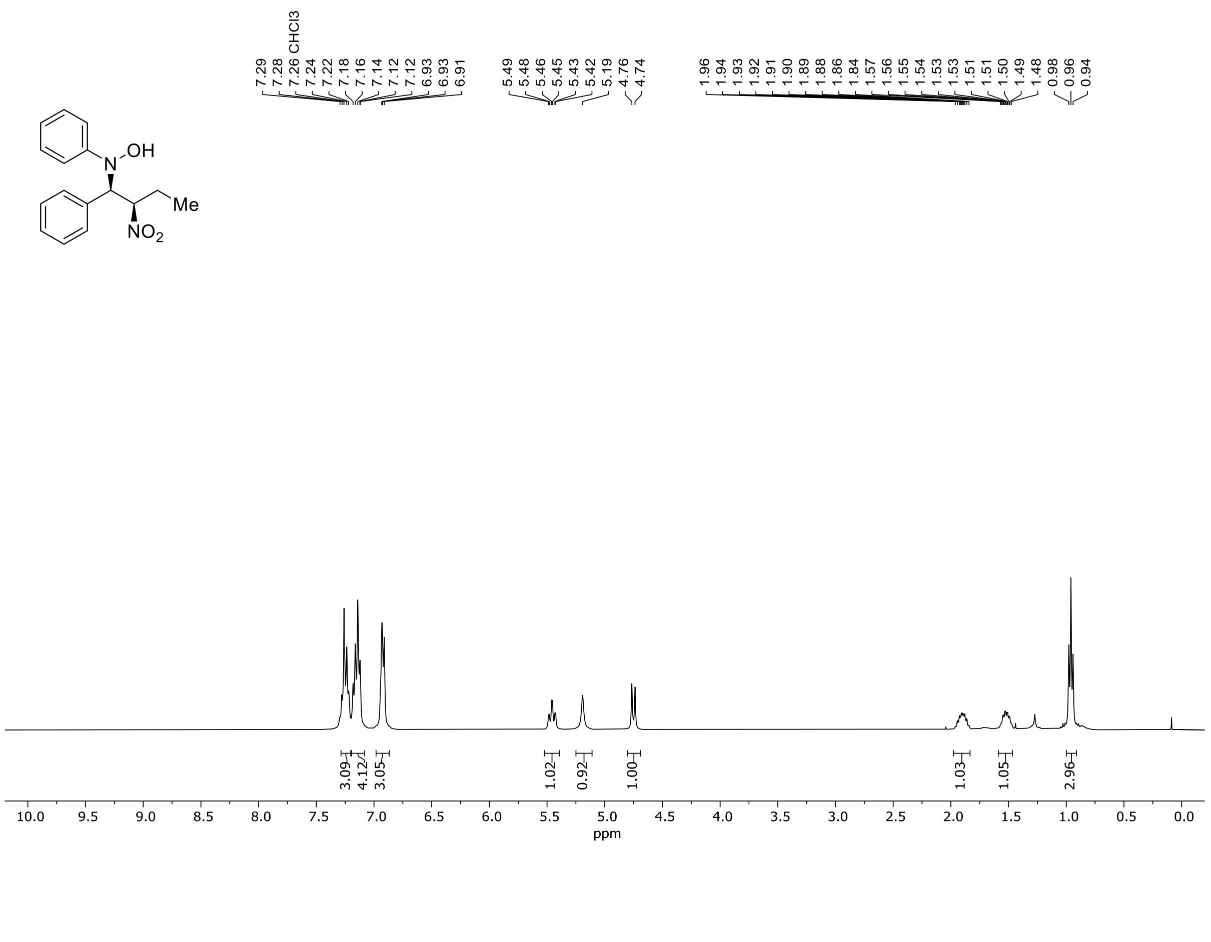

Figure S117. ^{13}C-NMR (101 MHz, $CDCl_3$, 298 K) spectrum of **4**.

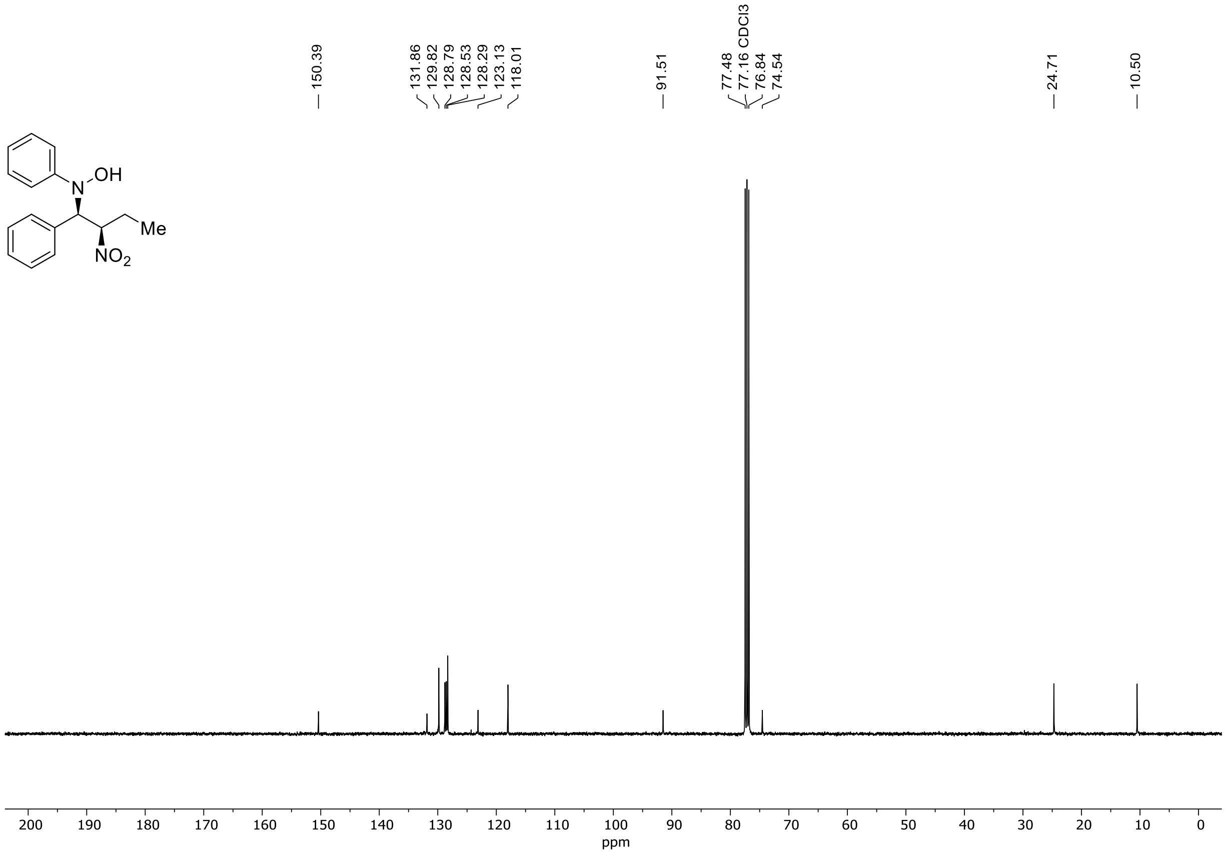

Figure S118. ^{1}H-NMR (400 MHz, $CDCl_3$, 298 K) spectrum of **5**.

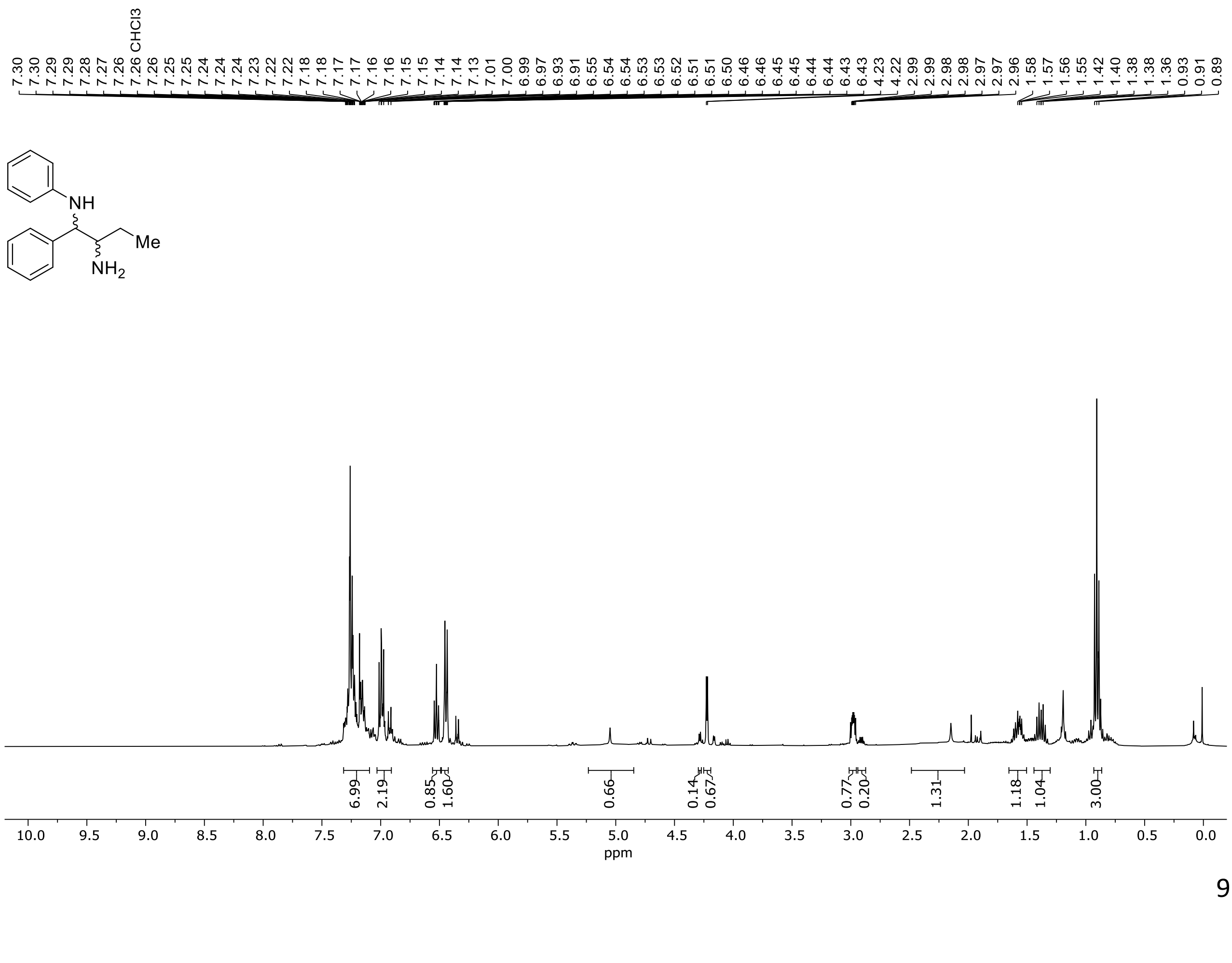

Figure S119. ^{13}C-NMR (101 MHz, $CDCl_3$, 298 K) spectrum of **5**.

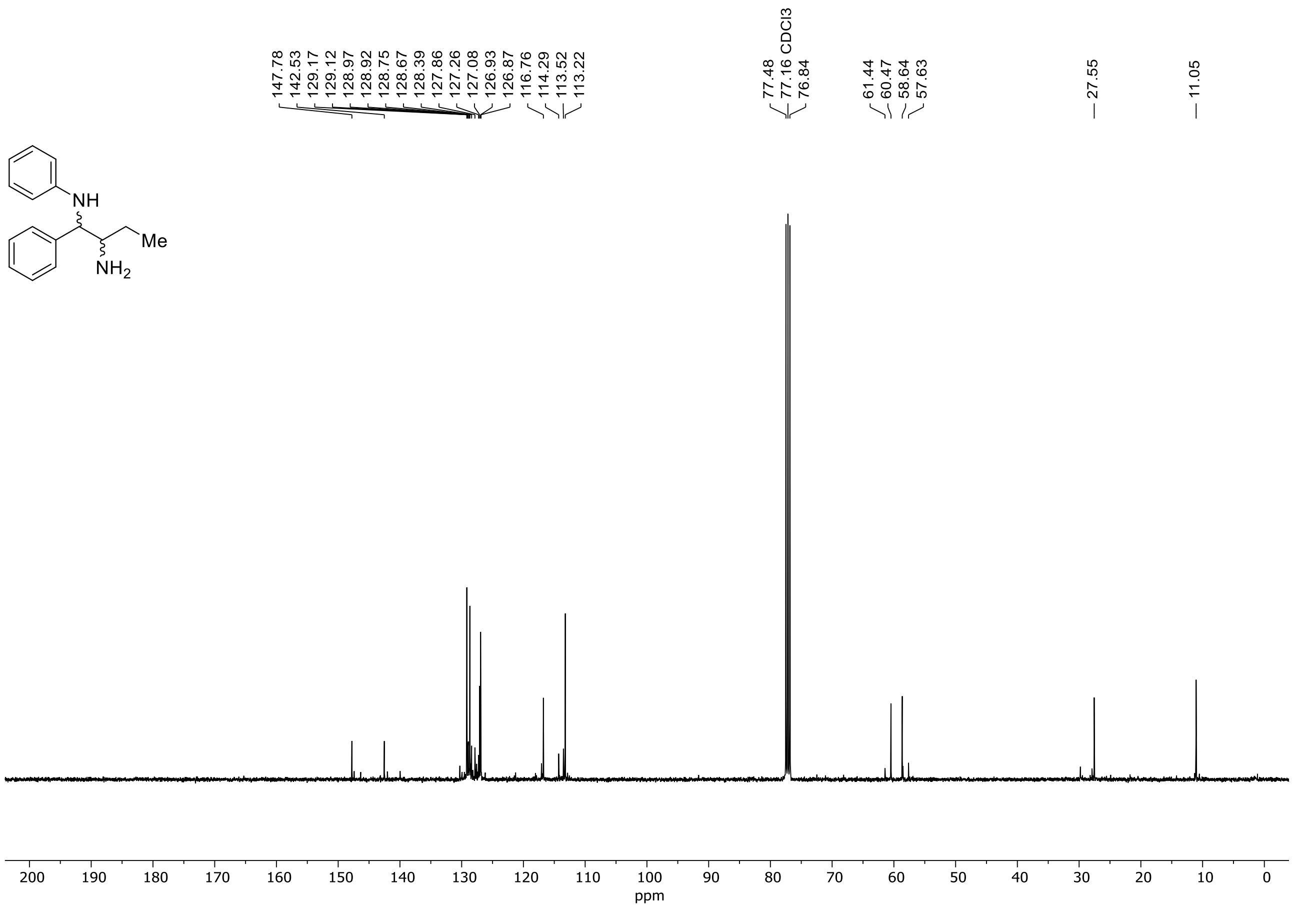

8. Crystallographic data

Single crystals of **3a'** and **3l'** were grown from dichloromethane at room temperature, crystals of **1aa**·$B(C_6F_5)_3$ and **1ao**·$B(C_6F_5)_3$ were grown in an N_2 filled glovebox by slow evaporation from dichloromethane and toluene respectively at -38 °C. Crystallographic studies were undertaken on a single crystal mounted in Fomblin®Y and studied on an Agilent SuperNova Dual Atlas three-circle diffractometer using Cu-Kα radiation and a CCD detector. Measurements were taken at 200(2) K with temperatures maintained using an Oxford cryostream. Data were collected, integrated and corrected for absorption within CrysAlisPro.[16] The absorption correction implemented a numerical absorption correction based on Gaussian integration over a multifaceted crystal model. The structure was solved by intrinsic phasing and refined against F^2 within SHELXL-2013.[17] The structures have been deposited with the Cambridge Structural Database (deposition codes: 2301585–2301589). This can be obtained free of charge from the Cambridge Crystallographic Data Centre via www.ccdc.cam.ac.uk/data_request/cif.

Figure S120. Solid-state structure of compound **3a'**, thermal ellipsoids drawn at 50% probability. H atoms (except on stereocenters) omitted for clarity. C atoms in grey, N in blue, O in red, Si in beige.

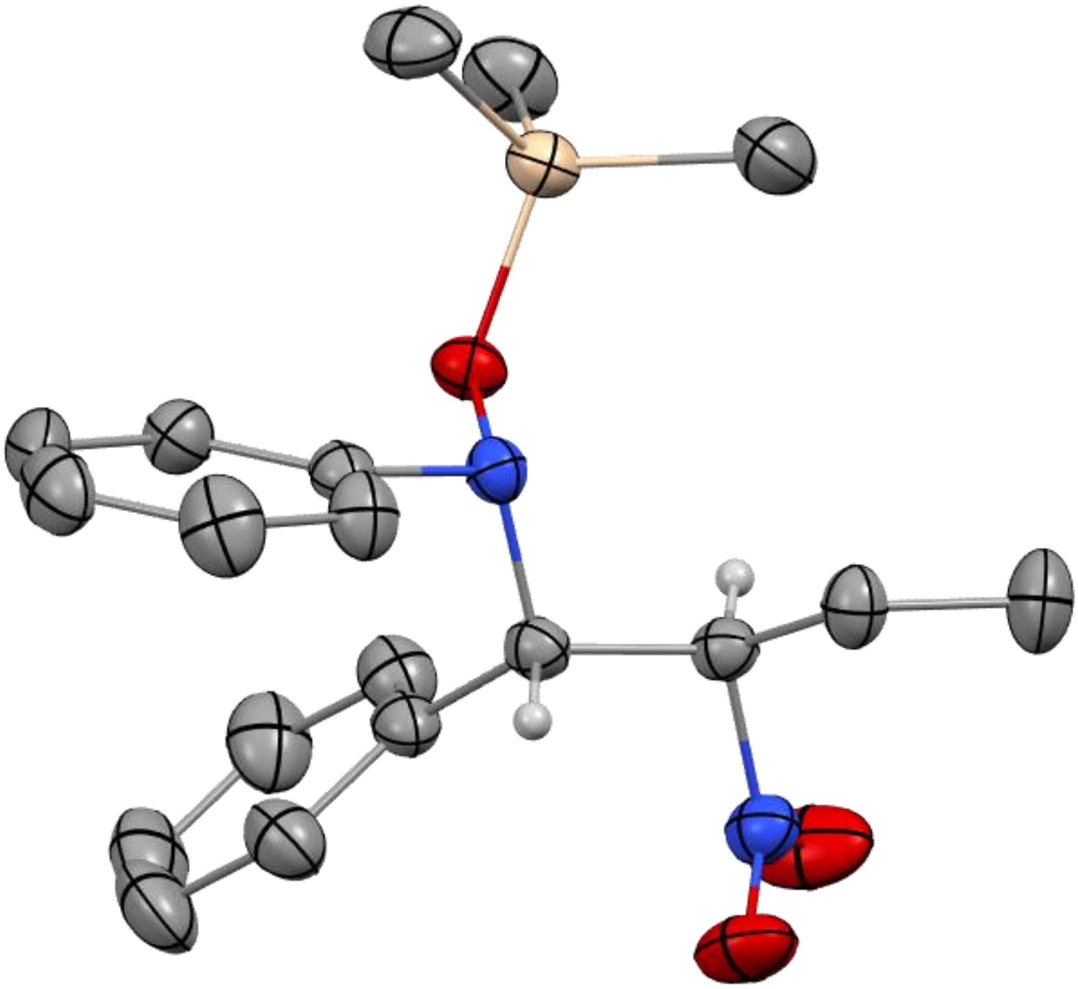

Figure S121. Solid-state structure of compound **3l'**, thermal ellipsoids drawn at 50% probability. H atoms (except on stereocenters) omitted for clarity. C atoms in grey, N in blue, O in red, Si in beige).

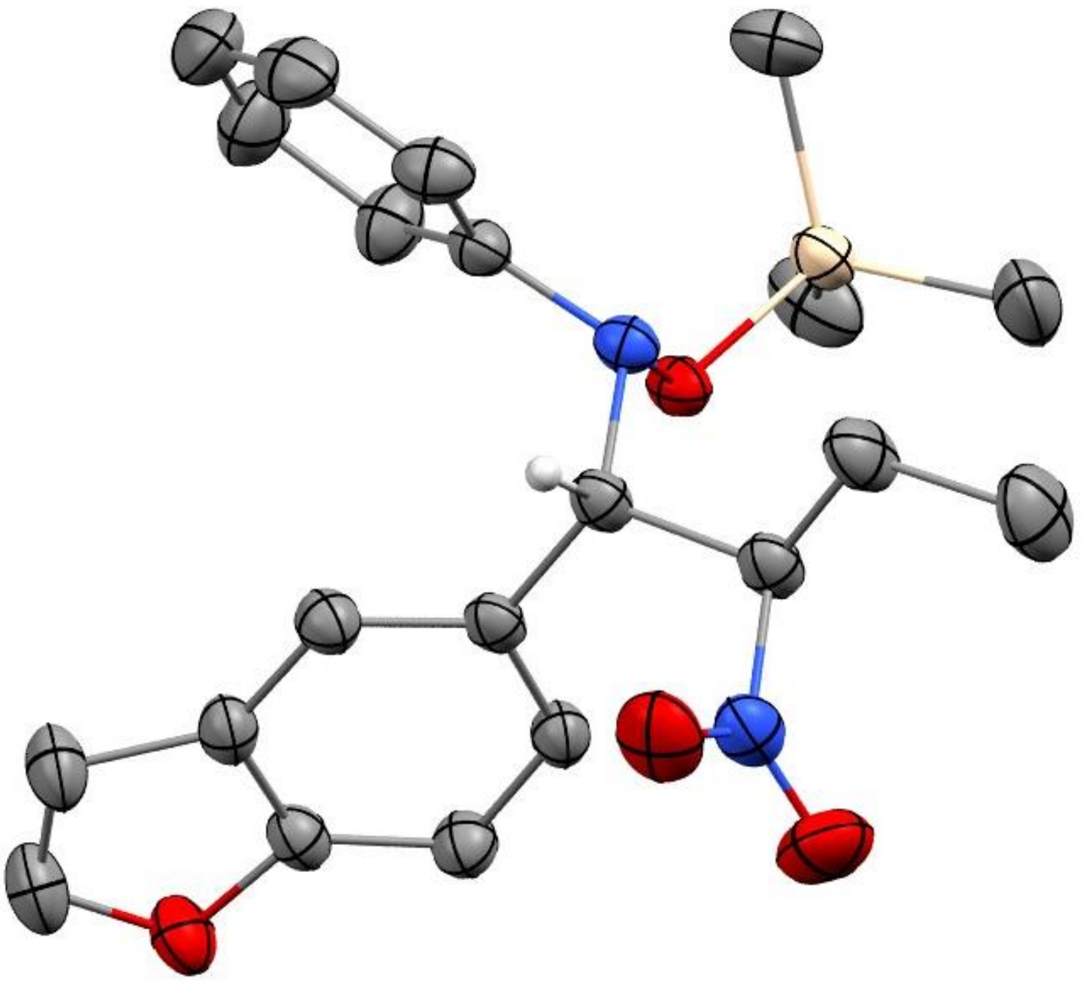

Figure S122. Solid-state structure of compound **1aa**·$B(C_6F_5)_3$, thermal ellipsoids drawn at 35% probability. H atoms omitted for clarity. C atoms in black, N in blue, O in red, B in pink, F in green.

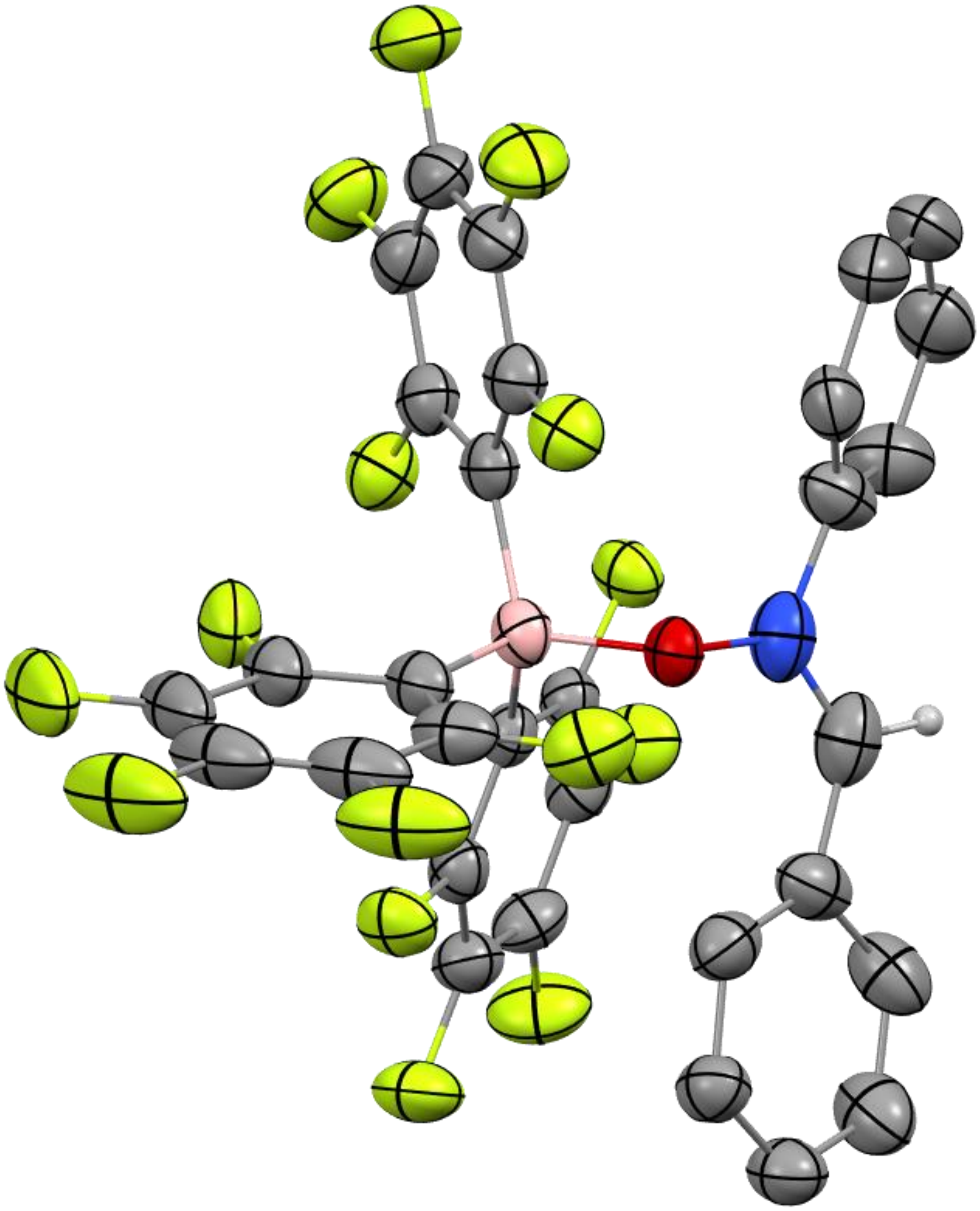

Figure S123. Solid-state structure of compound **1ao**·B(C_6F_5)$_3$, thermal ellipsoids drawn at 50% probability. H atoms (except *H*–C=N) omitted for clarity. C atoms in grey, N in blue, O in red, B in pink, F in green.

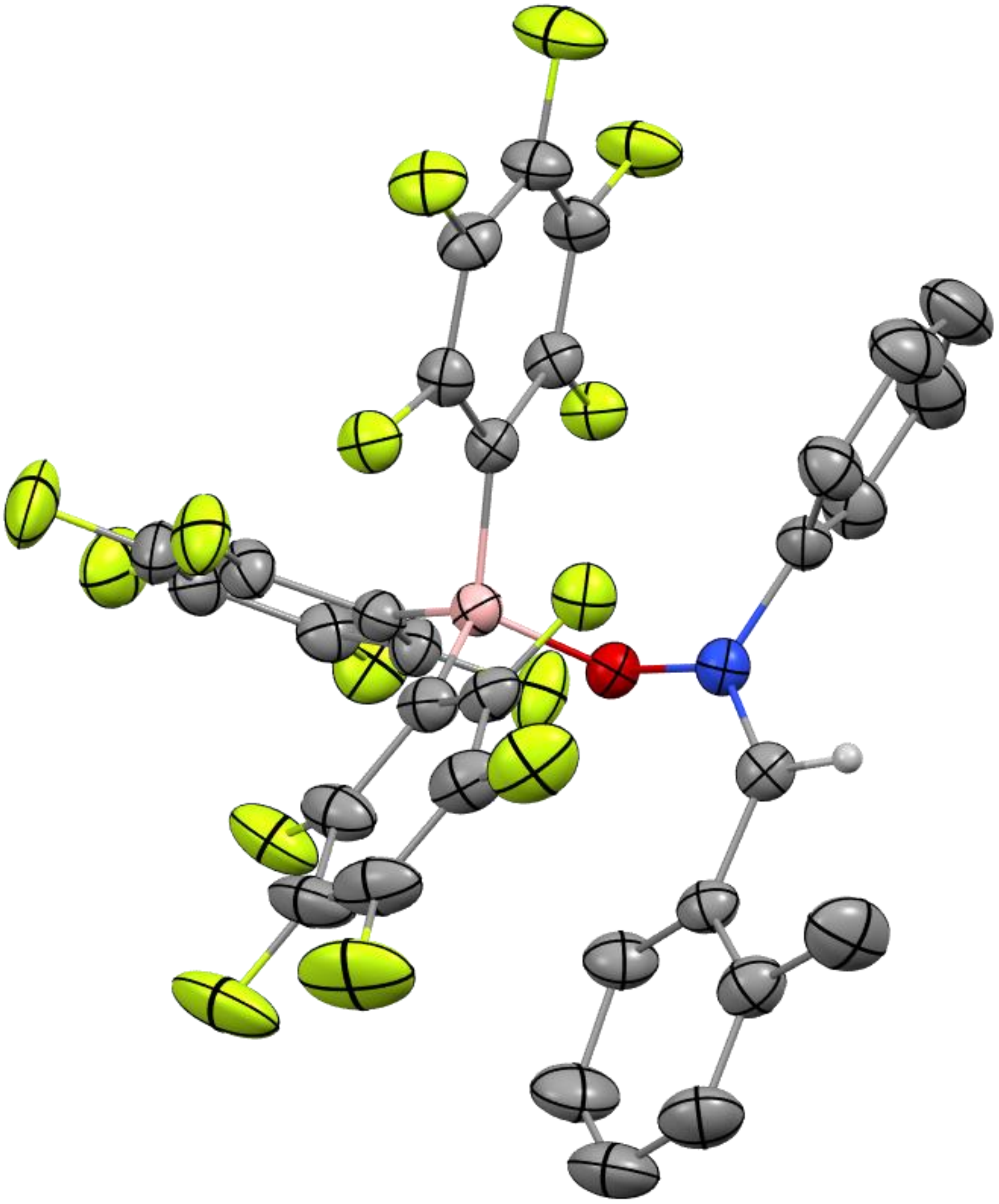

Figure S124. Solid-state structure of compound **1ap·B(C₆F₅)₃**, thermal ellipsoids drawn at 35% probability. H atoms omitted for clarity. C atoms in black, N in blue, O in red, B in pink, Cl in green.

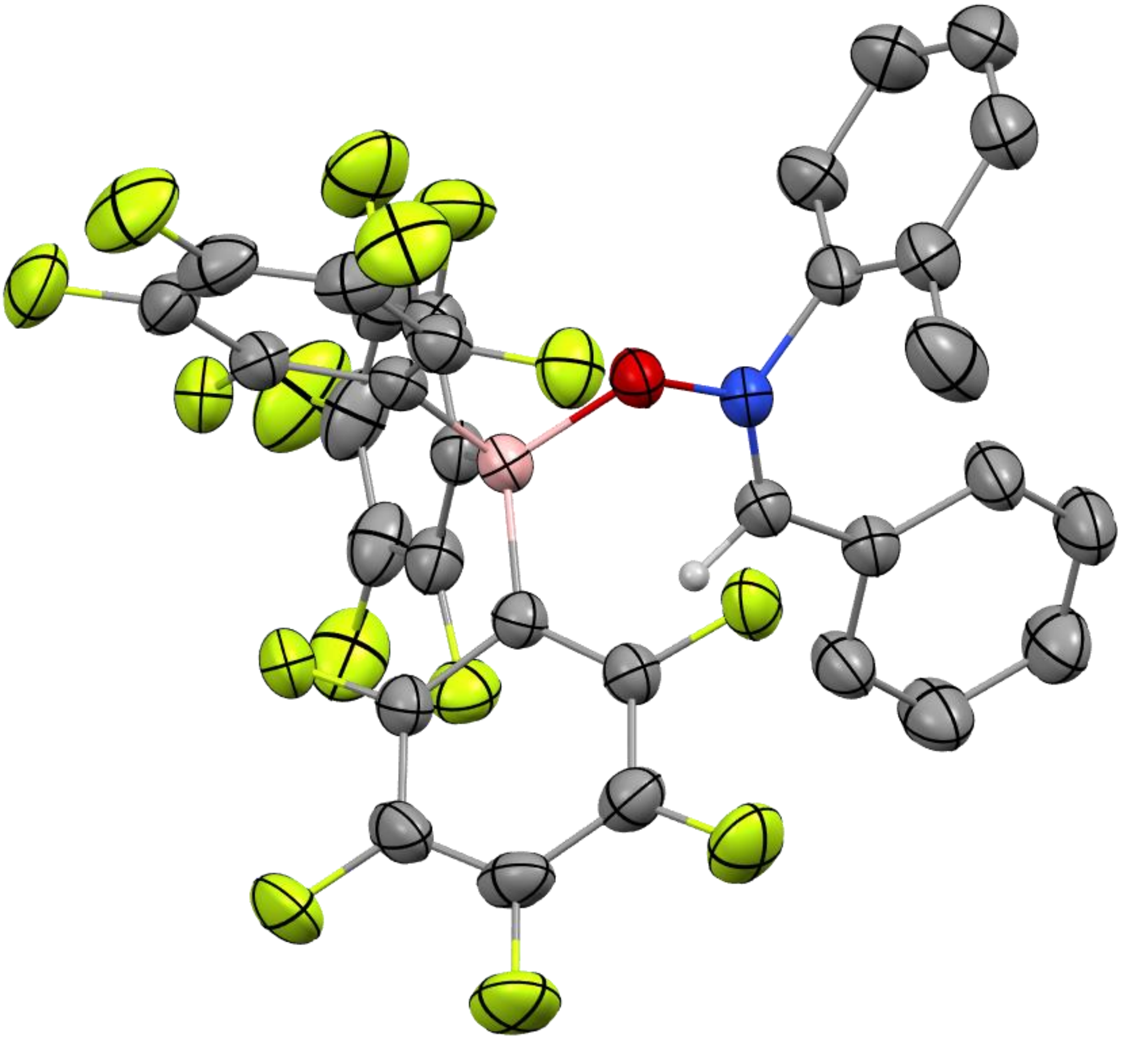

9.1 X-Ray refinement data

Table S2. Crystal data and structure refinement for compound **3a'**.

**Compound**	**3a'**
Empirical formula	$C_{19} H_{26} N_2 O_3 Si$
M_r	358.51
Crystal system	Monoclinic
Space group	Cc
Temperature (K)	200
a, *b*, *c* (Å)	15.3631(5), 11.8472(3), 22.4626(7)
α, β, γ (°)	90, 90.492(3), 90
Volume, V (Å^3)	4088.3(2)
Z	8
Density, calc (g cm^{-3})	1.165
Absorption coefficient, μ (mm^{-1})	0.133
Crystal size (mm)	0.496 × 0.192 × 0.149
Radiation type	Mo K\α
Wavelength (Å)	0.71073
θ range (°)	3.439–29.719
Index ranges	$-16 \leq h \leq 20$
	$-15 \leq k \leq 15$
	$-30 \leq l \leq 28$
Reflections collected	19513
Independent reflections	7966
R(int)	0.0218
Absorption correction	Gaussian
Data / restraints / parameters	7966 / 2 / 472
Goodness of fit, S	1.149
Final R indices [I>2σ(I)]	$R_1 = 0.0364$
	$wR_2 = 0.0952$
R indices (all data)	$R_1 = 0.0409$
	$wR_2 = 0.0988$
Max residual electron density (e^-Å^{-3})	0.229
Min residual electron density (e^-Å^{-3})	-0.159

Table S3. Crystal data and structure refinement for compound **3l’**.

**Compound**	**3l’**
Empirical formula	$C_{19} H_{26} N_2 O_4$ Si
M_r	398.53
Crystal system	Orthorhombic
Space group	$P2_12_12_1$
Temperature (K)	200
a, *b*, *c* (Å)	9.9467(4), 10.7574(6), 19.8365(9)
α, β, γ (°)	90, 90, 90
Volume, V (Å^3)	2122.52(17)
Z	4
Density, calc (g cm^{-3})	1.247
Absorption coefficient, μ (mm^{-1})	0.14
Crystal size (mm)	1.00 × 0.44 × 0.17
Radiation type	Mo K\α
Wavelength (Å)	0.71073
θ range (°)	3.439–29.719
Index ranges	$-12 \leq h \leq 13$
	$-13 \leq k \leq 12$
	$-27 \leq l \leq 25$
Reflections collected	9161
Independent reflections	4976
R(int)	0.015
Absorption correction	Gaussian
Data / restraints / parameters	4976 / 0 / 257
Goodness of fit, S	1.03
Final R indices [I>2σ(I)]	R_1 = 0.0418
	wR_2 = 0.0354
R indices (all data)	R_1 = 0.0858
	wR_2 = 0.0816
Max residual electron density (e^-Å^{-3})	0.22
Min residual electron density (e^-Å^{-3})	-0.19

Table S4. Crystal data and structure refinement for compound **1aa**·$B(C_6F_5)_3$.

**Compound**	**1aa**·$B(C_6F_5)_3$
Empirical formula	C_{31} H_{11} B F_{15} N O
M_r	709.22
Crystal system	Triclinic
Space group	P-1
Temperature (K)	200
a, *b*, *c* (Å)	9.0442(6), 9.8687(6), 16.2600(10)
α, β, γ (°)	83.784(5), 80.686(5), 75.180(5)
Volume, V (Å^3)	1381.10(16)
Z	2
Density, calc (g cm^{-3})	1.705
Absorption coefficient, μ (mm^{-1})	1.561
Crystal size (mm)	0.155 × 0.138 × 0.084
Radiation type	Cu K\α
Wavelength (Å)	1.54178
θ range (°)	4.646 – 72.719
Index ranges	-6 ≤ h ≤ 11
	-11 ≤ k ≤12
	-17 ≤ l ≤ 19
Reflections collected	9529
Independent reflections	5315
R(int)	0.0221
Absorption correction	Gaussian
Data / restraints / parameters	5315 / 0 / 442
Goodness of fit, S	1.057
Final R indices [I>2σ(I)]	R_1 = 0575
	wR_2 = 0.1472
R indices (all data)	R_1 = 0.0727
	wR_2 = 0.1609
Max residual electron density (e^-Å^{-3})	1.245
Min residual electron density (e^-Å^{-3})	-0.440

Table S5. Crystal data and structure refinement for compound **1ao**·$B(C_6F_5)_3$.

**Compound**	**1ao**·$B(C_6F_5)_3$
Empirical formula	C_{32} H_{13} B F_{15} N O
M_r	723.24
Crystal system	Monoclinic
Space group	P 2_1/c
Temperature (K)	200
a, *b*, *c* (Å)	18.4252(5), 8.90582(3), 20.1712(6)
α, β, γ (°)	90, 107.799(3), 90
Volume, V (Å^3)	2851.55(16)
Z	4
Density, calc (g cm^{-3})	1.685
Absorption coefficient, μ (mm^{-1})	1.525
Crystal size (mm)	0.380 × 0.224 × 0.064
Radiation type	Cu K\α
Wavelength (Å)	1.54178
θ range (°)	4.52–72.78
Index ranges	-22 ≤ h ≤ 13
	-9 ≤ k ≤ 9
	-21 ≤ l ≤ 24
Reflections collected	11158
Independent reflections	5518
R(int)	0.026
Absorption correction	Gaussian
Data / restraints / parameters	5518 / 0 / 452
Goodness of fit, S	1.149
Final R indices [I>2σ(I)]	R_1 = 0.0514
	wR_2 = 1287
R indices (all data)	R_1 = 0.0626
	wR_2 = 0.1353
Max residual electron density (e^-Å^{-3})	0.295
Min residual electron density (e^-Å^{-3})	-0.260

Table S6. Crystal data and structure refinement for compound **1ap**·$B(C_6F_5)_3$.

**Compound**	**1ap**·$B(C_6F_5)_3$
Empirical formula	C_{32} H_{13} B F_{15} N O
M_r	723.24
Crystal system	Monoclinic
Space group	P 2_1/c
Temperature (K)	200
a, *b*, *c* (Å)	13.0639(4), 11.4369(3), 19.0593(5)
α, β, γ (°)	90, 93.949(2), 90
Volume, V (Å^3)	2840.90(14)
Z	4
Density, calc (g cm^{-3})	1.691
Absorption coefficient, μ (mm^{-1})	1.531
Crystal size (mm)	0.249 × 0.211 × 0.154
Radiation type	Cu K\α
Wavelength (Å)	1.54178
θ range (°)	3.96–72.64
Index ranges	-15 ≤ h ≤ 10
	-14 ≤ k ≤ 12
	-23 ≤ l ≤ 20
Reflections collected	11862
Independent reflections	5511
R(int)	0.0240
Absorption correction	Gaussian
Data / restraints / parameters	5511 / 0 / 452
Goodness of fit, S	1.013
Final R indices [I>2σ(I)]	R_1 = 0.0458
	wR_2 = 0.1243
R indices (all data)	R_1 = 0.0587
	wR_2 = 0.1369
Max residual electron density (e^-Å^{-3})	0.596
Min residual electron density (e^-Å^{-3})	-0.265

9. Computational details

Gaussian 09, Revision D.01[18] was used to fully optimize all the structures at the BP86 level[19] of theory and the 6-31G* basis set[20] was chosen for all atoms. Frequency calculations were carried out at the same level of theory as those for the structural optimization. Transition structures were located using the Berny algorithm and intrinsic reaction coordinate (IRC) calculations[21] were employed to confirm the connectivity between transition structures and minima. To further refine the energies obtained from the BP86/6-31G* calculations, single-point energy calculations using the BP86-D3[22] functional method were carried out for all of the structures with a larger basis set def2-TZVP[23] and the SMD solvation model[24] in dichloromethane. All thermodynamic data were calculated in the standard state (298.15 K and 1 atm). An additional correction for compression of 1 mol of an ideal gas from 1 atm to the 1 M solution phase standard state (1.89 kcal/mol) was applied.[25] CYLview software was employed to present the 3D structures in the DFT section.[26]

Table S7. Cartesian coordinates and total energies for the calculated structures in dichloromethane.

2

E(BP86/6-31G*) = -732.3446035 au

H(BP86/6-31G*) = -732.126282 au

G(BP86/6-31G*) = -732.185268 au

E(SMD/BP86-D3/def2-TZVP//BP86/6-31G*) = -732.5891702 au

C	2.03759400	0.48163100	-0.17960600
C	3.28647700	-0.34628200	-0.13385000
C	-2.20301800	-1.08021200	-1.42623300
H	-1.58315300	-1.98946300	-1.37794600
H	-2.00510600	-0.57771300	-2.38990100
H	-3.26766600	-1.37961100	-1.41732800
C	-2.08739800	-0.73507500	1.70391500
H	-1.45496000	-1.63000600	1.81630500
H	-3.14454600	-1.03472000	1.82987600
H	-1.83993100	-0.03219000	2.51939200
O	-0.24975200	0.80865600	-0.12165700
Si	-1.83322000	0.07747600	0.01894900
C	-2.87564500	1.65076900	-0.11533700
H	-3.95153800	1.40316400	-0.05714200
H	-2.64860600	2.35801500	0.70134900
H	-2.70157200	2.17092000	-1.07332200
N	0.86331000	-0.10370100	-0.07312500
O	0.61282800	-1.32764900	0.06618700
H	2.02246100	1.56434800	-0.30408700
C	4.53282400	0.49889100	0.17694400
H	5.43798500	-0.13195200	0.18711800
H	4.68345600	1.28873900	-0.58112700
H	4.44864200	0.98890500	1.16299600
H	3.42333300	-0.87696600	-1.10029100

H	3.15350500	-1.14661100	0.61946400

$B(C_6F_5)_3$

E(BP86/6-31G*) = -2208.23786 au
H(BP86/6-31G*) = -2208.058736 au
G(BP86/6-31G*) = -2208.152212 au
E(SMD/BP86-D3/def2-TZVP//BP86/6-31G*) = -2209.258339 au

B	0.00014100	-0.00112900	0.00037600
C	-1.22473600	-0.98497900	-0.00063000
C	-2.40748300	-0.71772200	-0.72712400
C	-3.50519000	-1.58662400	-0.74900900
C	-3.45347000	-2.77354100	-0.00095500
C	-2.30590500	-3.08123300	0.74704100
C	-1.21986300	-2.19784600	0.72520200
C	-0.23973600	1.55178600	0.00071700
C	0.57859000	2.44256200	-0.73023900
C	0.37313700	3.82740900	-0.75265600
C	-0.67797000	4.37558100	-0.00039900
C	-1.51353700	3.53522100	0.75234900
C	-1.28941300	2.15328400	0.73126300
C	1.46509100	-0.56893200	0.00111800
C	2.51171700	0.04302300	0.72773200
C	3.82098500	-0.45289200	0.74882700
C	4.13069200	-1.59968500	0.00049300
C	3.12966400	-2.24004300	-0.74707900
C	1.82744900	-1.72599200	-0.72544100
F	-2.50722600	0.40164500	-1.47816600
F	-4.60152900	-1.29876900	-1.47337800
F	-4.49911000	-3.61264100	-0.00098500
F	-2.26229800	-4.21424900	1.47092300
F	-0.14829700	-2.53802900	1.47540900
F	1.59488400	1.96924500	-1.48528500
F	1.16658600	4.63314300	-1.48125300
F	-0.88350700	5.70045100	-0.00078100
F	-2.11460700	1.39465700	1.48658700
F	0.90935800	-2.37403900	-1.47631700
F	3.43039900	-3.33302100	-1.47129100
F	5.38126300	-2.08294200	0.00010900
F	4.77912300	0.15308500	1.47289500
F	2.26856600	1.13941500	1.47970600
F	-2.51369900	4.06309200	1.48081200

1aa

E(BP86/6-31G*) = -631.9147792 au
H(BP86/6-31G*) = -631.700809 au
G(BP86/6-31G*) = -631.750877 au
E(SMD/BP86-D3/def2-TZVP//BP86/6-31G*) = -632.1918333au

N	0.47564300	-0.47684000	-0.00000200
C	-0.48170100	0.45894300	0.00000200
C	1.90291200	-0.08868500	-0.00000200
C	2.35768900	1.24338500	-0.00001900
C	2.82173100	-1.15007200	0.00001700
C	3.73618500	1.49973400	-0.00001700
H	1.66992400	2.09284600	-0.00003600
C	4.19686600	-0.88049700	0.00001900
H	2.42485700	-2.16729100	0.00002900
C	4.66154100	0.44334100	0.00000200
H	4.08484100	2.53819800	-0.00003100
H	4.90721700	-1.71447300	0.00003400
H	5.73641000	0.65388400	0.00000400
O	0.24091000	-1.74090800	-0.00000600
C	-1.91007500	0.23674300	0.00000100
C	-2.73529400	1.39716600	0.00001600
C	-2.54169200	-1.03810000	-0.00001400
C	-4.12774700	1.29357500	0.00001600
H	-2.26409300	2.38811500	0.00002700
C	-3.93895100	-1.12796000	-0.00001300
H	-1.91418000	-1.92990900	-0.00002500
C	-4.73924000	0.02674600	0.00000100
H	-4.74052100	2.20205300	0.00002700
H	-4.40980600	-2.11771800	-0.00002500
H	-5.83174600	-0.05729000	0.00000100
H	-0.13302400	1.49081500	0.00000900

1ao
E(BP86/6-31G*) = -671.2300704 au
H(BP86/6-31G*) = -670.986337 au
G(BP86/6-31G*) = -671.042553 au
E(SMD/BP86-D3/def2-TZVP//BP86/6-31G*) = -671.5267724 au

N	-0.33053200	0.29985300	-0.33242900
C	0.61995800	-0.48519200	0.19000600
C	-1.73157800	-0.10977300	-0.17699100
C	-2.11582200	-1.37675400	-0.64097500
C	-2.65602200	0.79646400	0.39227400
C	-3.45534700	-1.77942600	-0.53795500
C	-3.99073100	0.35781200	0.48715300
C	-4.39434600	-0.90694000	0.02991300
H	-3.75928700	-2.76463900	-0.90727100
H	-4.72926800	1.03191400	0.93740000
H	-5.44436700	-1.20704100	0.11708600
O	-0.13813700	1.40041300	-0.97232000
C	2.05087600	-0.28086600	0.11123400
C	2.87857500	-1.24213700	0.75462900
C	2.67401900	0.80614100	-0.56125300

C	4.27064700	-1.12623900	0.72867000
H	2.41112700	-2.08599500	1.27732600
C	4.07027000	0.90978600	-0.58092700
H	2.04043200	1.54563700	-1.05375100
C	4.87548300	-0.04740500	0.05897600
H	4.88814900	-1.87916800	1.23141100
H	4.53623100	1.75268600	-1.10425000
H	5.96715200	0.04489300	0.03762100
H	0.23676100	-1.35331400	0.73151000
C	-2.25230400	2.16438500	0.89195200
H	-1.34716200	2.11666300	1.52292000
H	-2.00970100	2.83418900	0.05042000
H	-3.06672800	2.61217100	1.48609500
H	-1.36658600	-2.02940400	-1.10120000

1aa·B(C$_6$F$_5$)$_3$

E(BP86/6-31G*) = -2840.170441 au
H(BP86/6-31G*) = -2839.77412 au
G(BP86/6-31G*) = -2839.896323 au
E(SMD/BP86-D3/def2-TZVP//BP86/6-31G*) = -2841.492098 au

N	0.05456200	-1.69310500	1.63219300
C	0.98458800	-2.58163500	1.94730700
C	-1.35344300	-2.04082900	1.75849300
C	-2.21096100	-1.14092800	2.40909100
C	-1.80798600	-3.28668800	1.28943500
C	-3.55139000	-1.50690700	2.59470300
H	-1.82034500	-0.18950700	2.77487900
C	-3.15001000	-3.63557200	1.48775400
H	-1.13510400	-3.94869900	0.73547100
C	-4.02330900	-2.74859900	2.14023500
H	-4.22833000	-0.81311300	3.10358500
H	-3.51734400	-4.59557500	1.11073200
H	-5.07358500	-3.02258800	2.28398400
O	0.39903700	-0.41476900	1.37075000
B	-0.11162200	0.32644200	0.02409300
C	-0.84536200	-0.80137100	-0.90693700
C	-0.08927200	-1.85851700	-1.44978000
C	-2.21927500	-0.85800300	-1.20279300
C	-0.62301300	-2.88143600	-2.24389400
C	-2.79937200	-1.85556400	-2.00452700
C	-1.99604400	-2.87441900	-2.53048100
C	-1.01006400	1.63951800	0.44472600
C	-1.55473300	2.42718800	-0.58885600
C	-1.16109500	2.19311800	1.72628600
C	-2.24018700	3.63114700	-0.38639100
C	-1.84398500	3.39448500	1.98354800
C	-2.38375200	4.12423100	0.91870300

C	1.26800500	0.95845200	-0.63705700
C	1.57852600	0.97833900	-2.00992900
C	2.17228700	1.67875600	0.16406400
C	2.71784900	1.60748500	-2.54025900
C	3.33002400	2.30750900	-0.31311700
C	3.60286000	2.27759900	-1.68716900
F	-3.08672600	0.04382500	-0.68470400
F	-4.12579200	-1.85452100	-2.25046100
F	-2.53627300	-3.84625000	-3.29023000
F	0.16265500	-3.86533600	-2.73299900
F	1.24570000	-1.93533400	-1.19389100
F	0.77830800	0.37443800	-2.92218700
F	2.96257200	1.57520000	-3.86668200
F	4.70360100	2.87938900	-2.17791300
F	4.16803000	2.95004700	0.53027500
F	1.95708000	1.77942000	1.50957600
F	-1.42596500	2.01581800	-1.87741300
F	-0.66099800	1.57069100	2.83531300
F	-1.96797700	3.85455700	3.24750500
F	-3.03751400	5.28055100	1.14360800
F	-2.74868100	4.32337100	-1.42670500
C	2.42444800	-2.46392500	1.94773000
C	3.15084500	-1.24603400	1.87290900
C	3.13891800	-3.68799400	2.07973400
C	4.54873300	-1.27135400	1.90380900
H	2.61586600	-0.29856200	1.82730700
C	4.53462700	-3.70009800	2.09419000
H	2.58382900	-4.63068600	2.15466900
C	5.24407800	-2.48903700	2.00322400
H	5.10023300	-0.32688500	1.85156500
H	5.07150800	-4.65028800	2.18193000
H	6.33938400	-2.49479700	2.02094600
H	0.56038800	-3.53790000	2.26677500

1ao·B(C_6F_5)$_3$

E(BP86/6-31G*) = -2879.483037 au
H(BP86/6-31G*) = -2879.058231 au
G(BP86/6-31G*) = -2879.18356 au
E(SMD/BP86-D3/def2-TZVP//BP86/6-31G*) = -2880.826598 au

N	-0.02665200	2.27629600	0.04837800
C	-0.78190600	3.35876900	0.02794100
C	1.35683600	2.42004000	0.51437200
C	1.63692000	2.21133300	1.87111300
C	2.32938500	2.85350700	-0.41727300
C	2.94895600	2.39742500	2.32869800
H	0.83386000	1.91409100	2.54791800
C	3.63573200	3.02210200	0.07816400

C	3.94812400	2.79321700	1.42811700
H	3.18433000	2.22665800	3.38375000
H	4.42077200	3.34617400	-0.61456800
H	4.97731100	2.93109500	1.77620200
O	-0.49420000	1.11607900	-0.47379700
B	0.02594200	-0.34087700	-0.06415700
C	1.60764800	-0.43910900	-0.49003200
C	1.97865900	-0.24300200	-1.83398900
C	2.68846600	-0.69170800	0.37341900
C	3.29454400	-0.29593400	-2.30731300
C	4.02540900	-0.76486200	-0.05346600
C	4.33217400	-0.57082000	-1.40480800
C	-0.32995500	-0.73731900	1.49519700
C	-0.04117800	-2.05123800	1.91553300
C	-1.05356500	0.01412600	2.43041000
C	-0.39923500	-2.57534400	3.16380000
C	-1.43362900	-0.45973900	3.69700600
C	-1.10919600	-1.76973200	4.06641300
C	-0.98973900	-1.27976100	-0.97357500
C	-0.60135200	-2.35776500	-1.79293700
C	-2.38560900	-1.12883100	-0.85237900
C	-1.51022800	-3.17771800	-2.48324400
C	-3.32965200	-1.91378600	-1.52947400
C	-2.88610600	-2.95370200	-2.35593300
F	2.50360400	-0.84263200	1.70931200
F	5.01584400	-1.00461600	0.83183900
F	5.60899600	-0.62618500	-1.83117400
F	3.57323300	-0.08972000	-3.61274500
F	1.02298100	0.04019700	-2.76450300
F	0.70216500	-2.68588300	-1.96167700
F	-1.06563500	-4.18594900	-3.26161600
F	-3.76994900	-3.72772000	-3.01425800
F	-4.65320800	-1.68784200	-1.37698000
F	-2.90413700	-0.17406400	-0.02959200
F	0.62794000	-2.88637200	1.08008100
F	-1.41031300	1.30983800	2.16869700
F	-2.11232300	0.33608900	4.55242300
F	-1.46464900	-2.24771500	5.27464600
F	-0.07916000	-3.84129700	3.50170100
C	-2.12418200	3.56557700	-0.46940200
C	-2.88296400	2.62385400	-1.21264400
C	-2.68612700	4.84495100	-0.19915700
C	-4.16637000	2.96147000	-1.65426300
H	-2.45688500	1.64818300	-1.43482400
C	-3.97136700	5.16690900	-0.63831300
H	-2.10253200	5.58003600	0.36799200
C	-4.71622900	4.22272900	-1.36759000

H	-4.74573300	2.22796500	-2.22441800
H	-4.39375900	6.15226300	-0.41573100
H	-5.72481600	4.47226300	-1.71474600
H	-0.26703700	4.22533000	0.45412900
C	1.98700800	3.15624600	-1.85889400
H	1.42141200	4.10388900	-1.94790200
H	2.90483800	3.26462600	-2.45915800
H	1.37254400	2.36263800	-2.31449400

TS-3a

E(BP86/6-31G*) = -3572.500545 au

H(BP86/6-31G*) = -3571.88469 au

G(BP86/6-31G*) = -3572.04135 au

E(SMD/BP86-D3/def2-TZVP//BP86/6-31G*) = -3574.081601 au

N	-0.87948700	0.80061800	0.02719600
C	-2.08823900	0.09927500	-0.02775700
C	-0.94019400	2.22421700	0.19466500
C	-1.66371900	2.76795300	1.27939700
C	-0.31832600	3.07353700	-0.74172600
C	-1.77080500	4.15648400	1.41178000
H	-2.08903600	2.10903000	2.04224200
C	-0.43151100	4.46303300	-0.59139300
H	0.25209700	2.63650400	-1.56282900
C	-1.15887000	5.01041300	0.47699900
H	-2.31543900	4.57366100	2.26551000
H	0.05607300	5.11974400	-1.31985000
H	-1.23846900	6.09666400	0.59099600
O	0.17511900	0.30061900	-0.70552600
B	1.51002000	-0.08676800	0.06476200
C	2.49505000	1.23860700	0.21937700
C	2.75654700	2.03840300	-0.91002400
C	3.26885900	1.57744600	1.34537600
C	3.62526500	3.13750300	-0.92389500
C	4.16089100	2.66298000	1.38019800
C	4.34094700	3.45264600	0.23846900
C	1.06027200	-0.82134600	1.46961300
C	0.58528000	-0.08592900	2.57139500
C	1.02114700	-2.21563200	1.66168900
C	0.11528200	-0.65356700	3.76334700
C	0.57246200	-2.83376300	2.84105100
C	0.11468500	-2.04709600	3.90348300
C	2.34025800	-1.07010300	-0.97338800
C	3.56960200	-1.59513500	-0.52690500
C	2.04883300	-1.36588100	-2.31304900
C	4.43488000	-2.36393900	-1.31501600
C	2.88038800	-2.13544000	-3.14532000
C	4.08820100	-2.63406400	-2.64642400

F	3.20732900	0.85108000	2.48742900
F	4.85888300	2.94160100	2.50194200
F	5.19033800	4.49930500	0.25558000
F	3.79475100	3.87514200	-2.04444900
F	2.13856700	1.76717300	-2.10035000
F	3.96457500	-1.37158200	0.75390900
F	5.59288100	-2.84084100	-0.81100100
F	4.90150900	-3.37099600	-3.42973400
F	2.52438000	-2.38707600	-4.42532700
F	0.89305600	-0.94008000	-2.90722400
F	0.55526300	1.27540500	2.52569800
F	1.40035600	-3.07539600	0.68118600
F	0.56582700	-4.17987900	2.95157600
F	-0.33276900	-2.62055300	5.03881200
F	-0.33558500	0.12716800	4.77211200
C	-2.13895700	-1.39292200	0.05337800
C	-1.34205900	-2.26341900	-0.72399800
C	-3.09097900	-1.94563500	0.94175600
C	-1.48741200	-3.65101300	-0.59017900
H	-0.60869200	-1.85408900	-1.41948100
C	-3.22725600	-3.33359300	1.07448200
H	-3.71011100	-1.27852000	1.55342800
C	-2.42340800	-4.19052400	0.30658500
H	-0.85270600	-4.31460700	-1.18610000
H	-3.95523500	-3.74426400	1.78240200
H	-2.52365500	-5.27650600	0.40946100
H	-2.79675400	0.57057800	0.66795600
C	-2.94186200	0.65016400	-1.58769900
C	-2.24070700	0.14269500	-2.83671700
C	-7.57709800	0.78912500	-2.02429400
H	-7.28159400	-0.01105300	-2.72200100
H	-7.33986700	1.76261600	-2.48828400
H	-8.67409500	0.74117700	-1.89417700
C	-6.98288400	-1.07124400	0.45231600
H	-6.58877600	-1.88506800	-0.17686100
H	-8.06293700	-1.24819900	0.61134700
H	-6.48970300	-1.12295300	1.43870100
O	-4.99580200	0.97736500	-0.53647500
Si	-6.73326400	0.61290700	-0.34943400
C	-7.18290100	2.01957900	0.82014300
H	-8.25950500	1.97531700	1.06661200
H	-6.62145300	1.95806200	1.76847900
H	-6.98153700	3.00522400	0.36644700
N	-4.26989900	0.23478200	-1.47025600
O	-4.75831500	-0.80594000	-1.94069900
H	-2.89051700	1.73549300	-1.41316800
C	-2.75809600	0.82627200	-4.11700100

H	-2.19876000	0.45496300	-4.99243400
H	-3.82801000	0.61195800	-4.28391600
H	-2.62560300	1.92210100	-4.07351700
H	-2.37635800	-0.94920100	-2.90480400
H	-1.16173400	0.32560500	-2.71058500

TS-3a'

E(BP86/6-31G*) = -3572.492512 au

H(BP86/6-31G*) = -3571.876879 au

G(BP86/6-31G*) = -3572.032753 au

E(SMD/BP86-D3/def2-TZVP//BP86/6-31G*) = -3574.073643 au

N	0.96623700	-0.94749900	-0.87312000
C	1.39707100	-2.22412600	-0.56605400
C	1.51642200	-0.23988100	-1.98890400
C	1.60589400	-0.89410800	-3.23692200
C	1.92864600	1.09807900	-1.85970100
C	2.13127600	-0.21285800	-4.34151800
H	1.21695900	-1.91066100	-3.35345100
C	2.42906000	1.77531900	-2.97829400
H	1.87955700	1.56818000	-0.87794400
C	2.53829300	1.12581900	-4.22091900
H	2.18910500	-0.72248900	-5.30907200
H	2.74134600	2.82040100	-2.87537300
H	2.92804100	1.66334000	-5.09169700
O	0.39282900	-0.25061600	0.16379200
B	-1.04884900	0.39809000	0.07752900
C	-0.97097400	1.97664300	-0.43834900
C	0.00426300	2.84053300	0.09343900
C	-1.92213000	2.63041100	-1.24550000
C	0.11258100	4.20496900	-0.20357200
C	-1.86921000	3.99929800	-1.56112700
C	-0.84431800	4.79681100	-1.03800700
C	-2.00387200	-0.59468800	-0.82096300
C	-1.95389100	-0.59773100	-2.22772100
C	-2.88528000	-1.55138700	-0.28341400
C	-2.69251800	-1.46383200	-3.04609500
C	-3.65003300	-2.43838200	-1.05882400
C	-3.55511800	-2.39506500	-2.45411800
C	-1.50883800	0.54484100	1.66459600
C	-2.79449600	1.05920700	1.92833800
C	-0.73156000	0.34381600	2.81694900
C	-3.29203200	1.33487200	3.20802700
C	-1.18473600	0.60220300	4.12283100
C	-2.47267800	1.10929800	4.32298400
F	-2.97316900	1.96294900	-1.77779200
F	-2.80802800	4.55591500	-2.35532000
F	-0.77489900	6.10945900	-1.33619300

F	1.11642200	4.94862600	0.31744100
F	0.96387400	2.35054700	0.93894100
F	-3.63984700	1.30448700	0.89340900
F	-4.54127000	1.81696100	3.37753000
F	-2.92313300	1.36616800	5.56745100
F	-0.37931200	0.37116400	5.18440900
F	0.53921100	-0.16071100	2.75609200
F	-1.15316500	0.28393700	-2.88430000
F	-3.03982600	-1.69456800	1.05915600
F	-4.46964300	-3.33506500	-0.46841600
F	-4.27460400	-3.24225800	-3.21668600
F	-2.58498500	-1.40522200	-4.39221200
C	0.43689600	-3.23696600	-0.01851800
C	-0.30366700	-3.05455800	1.16752600
C	0.30677100	-4.44830400	-0.73518700
C	-1.17644000	-4.05921800	1.60902000
H	-0.18518400	-2.13513200	1.74374900
C	-0.57412700	-5.44476100	-0.29409700
H	0.88954400	-4.60349400	-1.65141700
C	-1.31832100	-5.25160800	0.88113500
H	-1.75594100	-3.90129100	2.52386800
H	-0.67753900	-6.37168100	-0.86854400
H	-2.00516800	-6.03011400	1.23024600
H	2.00578000	-2.61781900	-1.39088400
C	2.76971300	-2.16899900	0.83707900
C	3.13650500	-3.61306800	1.17065500
C	6.56887200	0.18714100	1.61894100
H	6.94172800	-0.77994100	1.24446700
H	6.18806000	0.04455900	2.64549900
H	7.42695000	0.88256600	1.67744800
C	5.80202700	1.04557000	-1.29833200
H	6.59020400	1.81641100	-1.38682200
H	4.96897200	1.33342700	-1.96161300
H	6.21293700	0.08791100	-1.65687300
O	3.76443300	-0.07131700	0.64513700
Si	5.24483500	0.92210700	0.49456300
C	4.56713900	2.53651100	1.18584900
H	5.26802900	3.36497600	0.97598300
H	3.58518100	2.80485600	0.76129400
H	4.44297400	2.46979800	2.28075100
N	3.86328200	-1.42977400	0.41239400
O	4.86196400	-1.88445300	-0.17895000
H	2.19673700	-1.59345500	1.57434800
C	4.16583000	-3.71834400	2.31166100
H	4.36212700	-4.77968600	2.54272000
H	5.12609000	-3.25213200	2.03385100
H	3.79378300	-3.23524500	3.23226100

H	3.52133500	-4.11477300	0.26461200
H	2.20775800	-4.12875500	1.45736900

TS-3o

E(BP86/6-31G*) = -3611.81304 au

H(BP86/6-31G*) = -3611.168266 au

G(BP86/6-31G*) = -3611.326785 au

E(SMD/BP86-D3/def2-TZVP//BP86/6-31G*) = -3613.412987 au

N	-0.92654400	0.63200500	0.08453800
C	-2.12604100	-0.07562500	0.01231300
C	-1.00631700	2.06541400	0.22018000
C	-1.61059000	2.65083900	1.36595000
C	-0.50557100	2.86416000	-0.82985700
C	-1.70148700	4.05606900	1.39412200
C	-0.61351100	4.25710800	-0.76487500
H	-0.02498700	2.37480000	-1.67885500
C	-1.21866400	4.85728500	0.35024600
H	-2.14531500	4.52897700	2.27846400
H	-0.22055400	4.86894400	-1.58362900
H	-1.30071600	5.94755700	0.41644900
O	0.12871000	0.12306200	-0.65115400
B	1.53922200	-0.08037200	0.04341400
C	2.47732900	1.28194600	-0.09720800
C	2.56218500	1.93640100	-1.34136800
C	3.38303500	1.77382900	0.86251300
C	3.37914900	3.04329300	-1.60631600
C	4.23001000	2.87397500	0.64403400
C	4.22838800	3.51823000	-0.59868800
C	1.25035600	-0.61453200	1.57324800
C	0.95450700	0.25776800	2.63628200
C	1.19709000	-1.97652200	1.92552100
C	0.65468300	-0.15667200	3.94196800
C	0.91045400	-2.44292700	3.21949100
C	0.63750500	-1.52496200	4.24025600
C	2.34432100	-1.17874000	-0.90150400
C	3.60267400	-1.62981200	-0.45437400
C	2.00295900	-1.63532700	-2.18341400
C	4.44629900	-2.47666700	-1.18453200
C	2.81051800	-2.48806100	-2.95583800
C	4.04736900	-2.90903600	-2.45702500
F	3.50179200	1.19683000	2.08295200
F	5.05849400	3.30525300	1.61907100
F	5.03234700	4.57657500	-0.82412400
F	3.37375600	3.63461500	-2.82240900
F	1.81147900	1.49985800	-2.39879900
F	4.05694800	-1.24169600	0.76599400
F	5.63465200	-2.87254600	-0.68118300

F	4.83873300	-3.72427300	-3.18299000
F	2.40273600	-2.89534500	-4.17896800
F	0.81428700	-1.30060500	-2.77041300
F	0.92964000	1.60381100	2.43561800
F	1.41124300	-2.94969500	1.00259300
F	0.88262800	-3.76697800	3.48284000
F	0.34735900	-1.95047700	5.48590200
F	0.36927700	0.74959700	4.90533500
C	-2.18652000	-1.56000900	0.17065900
C	-1.32028800	-2.47302400	-0.47112400
C	-3.21066700	-2.06267500	1.00876500
C	-1.46741500	-3.84976200	-0.25192800
H	-0.53947000	-2.10353200	-1.13560900
C	-3.35076400	-3.43936500	1.22463700
H	-3.88568500	-1.36287500	1.51584800
C	-2.47594700	-4.33789900	0.59368800
H	-0.77940700	-4.54480700	-0.74404400
H	-4.13841300	-3.80867900	1.89030600
H	-2.57954600	-5.41546800	0.76166800
H	-2.86833300	0.43364600	0.64122800
C	-2.93750300	0.38936900	-1.62857400
C	-2.21411300	-0.22566500	-2.81283900
C	-7.52377100	0.63086300	-2.23010100
H	-7.20148500	-0.19943800	-2.87911900
H	-7.25616000	1.58294000	-2.72105800
H	-8.62602200	0.59442300	-2.15113400
C	-7.09980300	-1.11448800	0.36129900
H	-8.18952400	-1.23595500	0.50599000
H	-6.62852400	-1.14244400	1.35941300
H	-6.73048100	-1.97289800	-0.22185800
O	-5.00633100	0.82379100	-0.64314300
Si	-6.75936800	0.51781400	-0.51232000
C	-7.21814300	1.99390700	0.56494500
H	-8.30580800	1.99260600	0.76181300
H	-6.70339500	1.96384800	1.54093500
H	-6.96585900	2.94986400	0.07465300
N	-4.26999000	0.00628200	-1.50805900
O	-4.76927900	-1.05501300	-1.91891100
H	-2.85989500	1.48108400	-1.51832300
C	-2.71454900	0.32997900	-4.16039200
H	-2.13696000	-0.11743300	-4.98703100
H	-3.77973500	0.09151800	-4.32378800
H	-2.59007100	1.42612500	-4.21846900
H	-2.33929100	-1.32071000	-2.77975200
H	-1.14023300	-0.02051800	-2.68530100
C	-2.11789900	1.85761100	2.55190100
H	-1.71043400	0.83569900	2.58153300

H	-1.82831200	2.35494700	3.49267500
H	-3.22331700	1.78555500	2.54757300

TS-3o'

E(BP86/6-31G*) = -3611.809276 au
H(BP86/6-31G*) = -3611.164697 au
G(BP86/6-31G*) = -3611.323401 au
E(SMD/BP86-D3/def2-TZVP//BP86/6-31G*) = -3613.410667 au

N	0.57172200	-1.38675300	-0.07634800
C	1.91530000	-1.25216600	0.23656600
C	0.07020900	-2.64644300	-0.56546800
C	0.14977200	-3.80383000	0.25345400
C	-0.48977400	-2.70013600	-1.85836600
C	-0.30798700	-5.01482400	-0.30360900
C	-0.94292200	-3.91812100	-2.37317000
C	-0.84298400	-5.08458200	-1.59627100
H	-0.26705500	-5.91915500	0.31542300
H	-1.37100100	-3.95460600	-3.38042900
H	-1.19858600	-6.04321900	-1.98910900
O	-0.02164400	-0.22644800	-0.52854100
B	-1.39693200	0.30235400	0.05000800
C	-2.68409800	-0.29823600	-0.80891800
C	-2.64357600	-0.29451900	-2.21645800
C	-3.94205300	-0.64659700	-0.28097500
C	-3.70153400	-0.68828900	-3.04617100
C	-5.03749000	-1.03944300	-1.06903200
C	-4.91922600	-1.06270100	-2.46350600
C	-1.40714300	-0.00791200	1.66692400
C	-1.73075400	-1.27759900	2.18015500
C	-1.03002600	0.92136800	2.65491000
C	-1.69474400	-1.61755500	3.53967100
C	-0.98753700	0.63420900	4.02932700
C	-1.32198800	-0.64822700	4.47908800
C	-1.40382300	1.91318300	-0.33973500
C	-2.48034100	2.69586300	0.12277800
C	-0.53533000	2.59458800	-1.20849500
C	-2.68410300	4.04027500	-0.21213600
C	-0.69521900	3.94325700	-1.57351600
C	-1.78109500	4.67246300	-1.07842900
F	-4.18049000	-0.61799200	1.05270400
F	-6.20964500	-1.37986500	-0.49203300
F	-5.95885500	-1.43985600	-3.23429700
F	-3.56603600	-0.68783300	-4.39156300
F	-1.50669700	0.10050900	-2.86815600
F	-3.39677400	2.14158700	0.95934900
F	-3.73662600	4.72670500	0.28152100
F	-1.95122900	5.96583900	-1.41987200

F	0.18966500	4.53636800	-2.40605100
F	0.56750200	1.99364600	-1.74438300
F	-2.10234100	-2.28289500	1.34183000
F	-0.65086900	2.18448500	2.32661600
F	-0.61330400	1.58001400	4.91776200
F	-1.27419100	-0.94872600	5.79196700
F	-2.00353000	-2.87098200	3.94574400
C	2.39611700	-0.26544600	1.24422400
C	2.03108700	1.09851300	1.26334800
C	3.27689800	-0.75640900	2.23742900
C	2.49967200	1.93399000	2.28808300
C	3.73927100	0.08315200	3.25930700
C	3.34504000	1.43182400	3.28889300
H	2.19076400	2.98368300	2.30285200
H	4.39427000	-0.31930500	4.04001900
H	3.69879800	2.08915800	4.09077700
H	2.36062100	-2.24651200	0.37273600
C	2.76146900	-0.80454000	-1.49265000
C	3.02363000	-2.12048600	-2.20911100
C	6.73632000	1.44624200	-2.44035700
H	5.95343400	2.22012700	-2.49261200
H	6.70518500	0.85390900	-3.37166800
H	7.71717200	1.95550500	-2.40200300
C	6.50167900	1.30423600	0.69980600
H	5.82938300	2.17500100	0.64738800
H	7.52108600	1.66075500	0.93848100
H	6.16178400	0.66610400	1.53352600
O	5.07257900	-0.65668900	-1.12385300
Si	6.53324400	0.35111000	-0.92068200
C	7.79950300	-1.04573300	-0.88249300
H	8.81822100	-0.63032300	-0.77615400
H	7.62573500	-1.72633000	-0.03097400
H	7.77528600	-1.64369300	-1.80973600
N	3.85808600	0.00958800	-1.27113000
O	3.81487900	1.23100100	-1.04825600
H	1.93324300	-0.17301500	-1.84678800
C	3.40810400	-1.92569500	-3.68924100
H	3.54442100	-2.90546800	-4.17908600
H	2.62132700	-1.37930200	-4.23819400
H	4.35151400	-1.36192700	-3.78862100
H	2.10119600	-2.71993900	-2.14874200
H	3.81278100	-2.67968900	-1.67819800
H	3.57250800	-1.81265800	2.22581300
H	1.39421400	1.49532700	0.47333900
C	0.63604200	-3.79093000	1.68840200
H	0.71223800	-2.77277000	2.09931900
H	-0.06789500	-4.35046200	2.32763400

H	1.62621600	-4.27561700	1.79045500
H	-0.55020800	-1.78115700	-2.44415200

10. References

1 Y. Soltani, L. C. Wilkins and R. L. Melen, *Angew. Chem. Int. Ed.*, 2017, **56**, 11995–11999.

2 Z. Zhou, G. Liu, Y. Chen and X. Lu, *Adv. Synth. Catal.*, 2015, **357**, 2944–2950.

3 M. M. C. Lo and G. C. Fu, *J. Am. Chem. Soc.*, 2002, **124**, 4572–4573.

4 A. Mukherjee, R. B. Dateer, R. Chaudhuri, S. Bhunia, S. N. Karad and R. S. Liu, *J. Am. Chem. Soc.*, 2011, **133**, 15372–15375.

5 N. Duguet, A. M. Z. Slawin and A. D. Smith, *Org. Lett.*, 2009, **11**, 3858–3861.

6 T. Liu, Z. Liu, Z. Liu, D. Hu and Y. Wang, *Synth.*, 2018, **50**, 1728–1736.

7 Z. Tian, J. Xu, B. Liu, Q. Tan and B. Xu, *Org. Lett.*, 2018, **20**, 2603–2606.

8 K. Stefkova, M. G. Guerzoni, Y. Van Ingen, E. Richards and R. L. Melen, *Org. Lett.*, 2023, **25**, 500–505.

9 D. Roca-López, T. Tejero and P. Merino, *J. Org. Chem.*, 2014, **79**, 8358–8365.

10 G. P. Marsh, P. J. Parsons, C. McCarthy and X. G. Cornique, *Org. Lett.*, 2007, **9**, 2613–2616.

11 S. Das, B. Mitschke, C. K. De, I. Harden, G. Bistoni and B. List, *Nat. Catal.*, 2021, **4**, 1043–1049.

12 X. Han, L. Dong, C. Geng and P. Jiao, *Org. Lett.*, 2015, **17**, 3194–3197.

13 S. L. Ioffe, V. M. Shitkin, B. N. Khasapov, M. V. Kashutina, V. A. Tartakovskii, M. Y. Myagi and E. T. Lippmaa, *Bull. Acad. Sci. USSR Div. Chem. Sci.*, 1973, **22**, 2100–2102.

14 D. W. Nelson, J. Owens and D. Hiraldo, *J. Org. Chem.*, 2001, **66**, 2572–2582.

15 M. Orlandi, F. Tosi, M. Bonsignore and M. Benaglia, *Org. Lett.*, 2015, **17**, 3941–3943.

16 SHELXL-2013, G.M. Sheldrick, University of Göttingen, Germany (2013).

17 CrysAlisPro, Agilent Technologies, Version 1.171.37.33 (release 27-03–2014 CrysAlis 171.NET)

18 Gaussian 09, Revision D.01, M. J. Frisch, G. W. Trucks, H. B. Schlegel, G. E. Scuseria, M. A. Robb, J. R. Cheeseman, G. Scalmani, V. Barone, B. Mennucci, G. A. Petersson, H. Nakatsuji, M. Caricato, X. Li, H. P. Hratchian, A. F. Izmaylov,

J. Bloino, G. Zheng, J. L. Sonnenberg, M. Hada, M. Ehara, K. Toyota, R. Fukuda, J. Hasegawa, M. Ishida, T. Nakajima, Y. Honda, O. Kitao, H. Nakai, T. Vreven, J. A. Montgomery, Jr., J. E. Peralta, F. Ogliaro, M. Bearpark, J. J. Heyd, E. Brothers, K. N. Kudin, V. N. Staroverov, T. Keith, R. Kobayashi, J. Normand, K. Raghavachari, A. Rendell, J. C. Burant, S. S. Iyengar, J. Tomasi, M. Cossi, N. Rega, J. M. Millam, M. Klene, J. E. Knox, J. B. Cross, V. Bakken, C. Adamo, J. Jaramillo, R. Gomperts, R. E. Stratmann, O. Yazyev, A. J. Austin, R. Cammi, C. Pomelli, J. W. Ochterski, R. L. Martin, K. Morokuma, V. G. Zakrzewski, G. A. Voth, P. Salvador, J. J. Dannenberg, S. Dapprich, A. D. Daniels, O. Farkas, J. B. Foresman, J. V. Ortiz, J. Cioslowski and D. J. Fox, Gaussian Inc., Wallingford CT, 2013.

19 (a) A. D. Becke, *Phys. Rev. A: At., Mol., Opt. Phys.,* 1988, **38**, 3098. (b) J. P. Perdew, *Phys. Rev. B: Condens. Matter Mater. Phys.,* 1986, **33**, 8822−8824.

20 P. C. Hariharan and J. A. Pople, *Theor. Chim. Acta.*, 1973, **28**, 213−222.

21 (a) K. Fukui, *J. Phys. Chem.*, 1970, **74**, 4161−4163. (b) K. Fukui, *Acc. Chem. Res.*, 1981, **14**, 363−368.

22 S. Grimme, J. Antony, S. Ehrlich and H. Krieg, *J. Chem. Phys.,* 2010, **132**, 154104−154119.

23 F. Weigend, F. Furche and R. Ahlrichs, *J. Chem. Phys.*, 2003, **119**, 12753–12762.

24 A. V. Marenich, C. J. Cramer and D. G. Truhlar, *J. Phys. Chem. B.*, 2009, **113**, 6378−6396.

25 J. W. Ochterski, Thermochemistry, Gaussian, Inc., Wallingford, CT, 2000.

26 C. Y. Legault, CYLview, 1.0b, Université de Sherbrooke, 2009 (http://www.cylview.org).
